# Clinical Background of Patients with Sperm in Their Urinary Sediment

**DOI:** 10.1371/journal.pone.0136844

**Published:** 2015-09-11

**Authors:** Masuomi Tomita, Eiji Kikuchi, Takahiro Maeda, Yusuke Kabeya, Takeshi Katsuki, Yoichi Oikawa, Kiyoe Kato, Masakazu Ohashi, So Nakamura, Mototsugu Oya, Akira Shimada

**Affiliations:** 1 Department of Internal Medicine, Tokyo Saiseikai Central Hospital, Tokyo, Japan; 2 Department of Urology, Keio University School of Medicine, Tokyo, Japan; 3 Department of Urology, Tokyo Saiseikai Central Hospital, Tokyo, Japan; 4 Department of Urology, Ogikubo Hospital, Tokyo, Japan; University of Glasgow, UNITED KINGDOM

## Abstract

**Introduction:**

The detection rate and associated factors of at least one sperm in urinary sediment is not well-known in real clinical practice.

**Aims:**

The aim of the present study was to evaluate the clinical features associated with the presence of sperm in urinary sediment in a large number of samples.

**Methods:**

We conducted a cross-sectional study at Tokyo Saiseikai Central Hospital. We identified 5,005 males who were aged ≥20 years in whom urinary sedimentation had been performed at least twice between May 2011 and June 2012. The sperm group included patients in whom at least one urinary sediment test performed under a microscope had detected at least one sperm. We evaluated the associations between the presence of at least one sperm in urinary sediment and clinical parameters such as various diseases and the use of particular oral medicines.

**Main Outcomes:**

In total, 1.6% (339/20,937) of urinary sediment samples contained at least one sperm. The sperm group consisted of 282 subjects (5.6%), and the no-sperm group included 4,723 subjects (94.3%).

**Results:**

Multivariate analysis demonstrated that younger age (<65) (odds ratio [OR]: 1.71, 95% confidence interval [CI]: 1.32–2.21), the total number of examinations (≥4) (OR: 1.46, 95%CI: 1.11–1.92), diabetes (OR: 1.72, 95%CI: 1.31–2.25), a history of pelvic surgery for colon cancer (OR: 4.89, 95%CI: 2.38–10.02), alpha-1 blocker use (OR: 1.55, 95%CI: 1.16–2.08), a history of trans-urethral resection of the prostate (OR: 2.77, 95%CI: 1.46–5.13), and selective serotonin reuptake inhibitor use (OR: 2.12, 95%CI: 1.07–4.19) were independent predictors of the presence of at least one sperm in urinary sediment.

**Conclusion:**

There is considerable overlap between the factors associated with the presence of at least one sperm in urinary sediment and those that are strongly associated with ejaculatory disorders.

## Introduction

Urinary sedimentation by centrifugal separation followed by a microscopic examination of the components of the sediment is routinely used to evaluate the general condition of urine and to detect kidney and urinary tract diseases in a timely and non-invasive manner. Most of the cellular components found in urinary sediment originate from the urinary tract, but sperm are occasionally detected. Sperm in urinary sediment are usually derived from the first post-ejaculatory voiding [1], and in older men sperm are sometimes found in urinary sediment due to reduced contraction of the internal urethral sphincter [2]. Furthermore, retrograde ejaculation (RE) causes a large number of sperm to be present in urinary sediment [2,3]. Although the only presence of sperm in urine does not imply RE [4], the presence of sperm in urinary sediment is an important factor in the diagnosis of RE [3]. However, there is no consensus as to defining of RE [5] and the rate of RE is subjectively evaluated by not-validated self-reported questionnaires in most of the studies [5–9]. Meanwhile, to the best of our knowledge there have not been any studies about the detection rate of at least one sperm in urinary sediment samples subjected to microscopic examinations, nor have any studies evaluated the associations between such a finding and clinical factors such as the presence of, or a history of, certain conditions or the use of particular medications. In fact, medical-staff often conduct routine urinary tests without paying particular attention to the presence/absence of sperm. Therefore, in the present study we evaluated 1) the detection rate of at least one sperm in urinary sediment in a large number of samples, 2) the associations between such a finding and clinical background factors, and 3) independent predictors for the presence of at least one sperm in urinary sediment.

## Materials and Methods

Urinalysis and urinary sediment were tested in 8,509 patients at Tokyo Saiseikai Central Hospital during the May 2011 to June 2012. We excluded the patients in whom urinalysis and urinary sedimentation tests had been performed only once (n = 3,504), which left 5,005 males aged ≥20 years (total number of measurements: 20,937) patients in whom the tests were performed at least twice. Among the 5,005 subjects, urinalysis and urinary sedimentation test were performed due to routine work-up for urological disease (N = 2,002), general check-ups for disorders of internal medicine (N = 2,600), health medical check-ups (N = 305), and unknown reasons (N = 98). The patients’ first urinary samples were discarded, and their second urinary samples were collected. The urine samples were submitted promptly after micturition and were analyzed using a fully automated urine element analyzer (UF-1000i, Sysmex Corporation, Kobe, Japan). If the analyzer detected the presence of a foreign body, trained medical technicians visually examined the sample under a microscope for the presence of at least one sperm under high magnification (400×, HPF). The sperm group included patients whose urine contained at least one sperm according to at least one urinary sediment test performed under a microscope, while the no-sperm group included patients in whom sperm was not detected in any urinary sediment test. We evaluated the associations between the presence of at least one sperm in urinary sediment and clinical background factors such as hypertension, dyslipidemia, diabetes, a history of pelvic surgery due to colorectal cancer, cardiovascular disease, prostatitis or transurethral resection of the prostate (TURP); or the use of selective serotonin reuptake inhibitors (SSRI), proton pump inhibitors (PPI), H2 blockers, or alpha-1 blockers by performing comparisons between the two groups **(Data in** S1 File **)**.

The Student’s unpaired *t* test was used to compare continuous variables, while the χ^2^ test was used for comparisons between categorical variables. Logistic regression analysis was performed to calculate odds ratios (OR) for the presence of at least one sperm in urine. First, univariate analysis was performed to identify variables that were significantly associated with the presence of at least one sperm in urinary sediment. Variables for which P <0.01 were then included in the multiple logistic regression analysis. The multivariate model was tested for goodness of fit using the Hosmer-Lemeshow test, which showed that the fit of the model was acceptable (P> 0.05). All analyses were performed using the STATA software program (version 10; StataCorp, Texas, USA). This study was a retrospective observation fashion and it was difficult to receive informed consents from the past participants. This study was approved as the following contents by the ethics committee of Saiseikai Central Hospital (No. 220). We displayed study contents inside the hospital and exclude the data from the study cohort if the patients hope to exclude it.

## Results

### Comparison of clinical features between the sperm group and no-sperm group

The study population consisted of 5,005 males (mean age: 66.0 ± 12.9 years) in whom urinary sedimentation was performed at least twice during the study period. The patients’ clinical background data are shown in Table 1. The mean number of examinations per patient was 4.2. In total, 1.6% (339/20,937) of the urinary sediment samples contained at least one sperm.

**Table 1:** Background characteristics of participants.

Characteristics	All subjects N = 5005	Presence of sperm		p value
		Yes N = 282(5.6%)	No N = 4723(94.3%)	
**Age(years)**	**66.0±12.9**	**63.8±11.5**	**66.1±12.9**	**0.003**
**The number of measurement**	**4.2±2.5**	**4.7±2.4**	**4.2±2.4**	**<0.001**
**Hypertension (%)**	**1953(39.0)**	**132(46.8)**	**1821(38.6)**	**0.006**
**Dyslipidemia (%)**	**1216(24.3)**	**86(30.5)**	**1130(23.9)**	**0.012**
**Diabetes (%)**	**1663(33.2)**	**135(47.8)**	**1528(32.3)**	**<0.001**
**Past history of cardio-vascular disease (%)**	**788(15.7)**	**55(19.5)**	**733(15.5)**	**0.074**
**The use of alpha-1 blocker agent (%)**	**1015(20.3)**	**72(25.5)**	**943(19.9)**	**0.024**
**Past history of intrapelvic surgery for colon cancer (%)**	**53(1.1)**	**10(3.5)**	**43(0.9)**	**<0.001**
**Past history of TUR-P (%)**	**113(2.3)**	**12(4.3)**	**101(2.1)**	**0.020**
**Prostatitis (%)**	**108(2.2)**	**5(1.8)**	**103(2.2)**	**0.640**
**The use of PPI or H2-bloker (%)**	**970(19.4)**	**59(20.9)**	**911(19.3)**	**0.500**
**The use of SSRI(%)**	**88(1.8)**	**10(3.6)**	**78(1.6)**	**0.020**

Data are n(%) or mean±SD, TUR-P:trans-urethral resection of the prostate, PPI: proton pump inhibitors, SSRI: selective serotonin reuptake Inhibitors

The study population consisted of 5,005 males and their clinical background data are shown in this table

The sperm group consisted of 282 people (5.6%), and the no-sperm group included 4,723 people (94.3%). Significant differences in age; the total number of urinary sediment examinations; and the frequencies of hypertension, dyslipidemia, diabetes, alpha-1 blocker use, SSRI use, TURP, and pelvic surgery due to colorectal cancer were observed between the sperm and no-sperm groups.

### Independent indicators for the presence of at least one sperm in urinary sediment

Table 2 shows the results of the uni- and multivariate analyses, which aimed to identify independent factors for the presence of at least one sperm in urinary sediment. In the univariate analysis, younger age (<65), the total number of urinary sediment examinations (≥4), hypertension, dyslipidemia, diabetes, a history of pelvic surgery for colon cancer, alpha-1 blocker use, a history of TURP, and SSRI use were significantly associated with the presence of at least one sperm in urinary sediment. In the multivariate analysis adjusted for these factors, younger age (<65), total number of urinary sediment examinations (≥4), diabetes, a history of pelvic surgery for colon cancer, alpha-1 blocker use, a history of TURP, and SSRI use were demonstrated to be independent indicators for the presence of at least one sperm in urinary sediment. The detection rate of at least one sperm in urinary sediment was 1.6% (11/678), 4.1% (72/1,737), 6.2% (109/1,755), 10.6% (81/763) and 12.9% (9/70) in patients who had no, one, two, three and four independent associated factors.

**Table 2:** Associated factors for at least one sperm in urinary sediment.

Variables		All subjects (N = 5005)		Univariate	Multivariate	
		No sperm group N = 4723	Sperm group N = 282	Pvalue	OR (95% CI)	Pvalue
**Age (years)**	**<65**	**1967**	**140**	**0.008**	**1.71 (1.32–2.21)**	**<0.001**
	**≥65**	**2756**	**142**		**1**	
**Total number of measurement of urinary sediment**	**2-<4**	**2196**	**92**	**<0.001**	**1**	**0.006**
	**≥4**	**2597**	**190**		**1.46 (1.11–1.92)**	
**Hypertension**	**NO**	**2902**	**150**	**0.009**		**0.390**
	**YES**	**1821**	**132**			
**Dyslipidemia**	**NO**	**3593**	**196**	**0.013**		**0.620**
	**YES**	**1130**	**86**			
**Diabetes mellitus**	**NO**	**3195**	**147**	**<0.001**	**1**	**<0.001**
	**YES**	**1528**	**135**		**1.72 (1.31–2.25)**	
**Past history of cardio-vascular disease**	**NO**	**733**	**55**	**0.070**		**0.350**
	**YES**	**3990**	**227**			
**Prostatitis**	**NO**	**4620**	**277**	**0.640**		**0.780**
	**YES**	**103**	**5**			
**The use of PPI or H2-bloker**	**NO**	**3812**	**223**	**0.500**		**0.860**
	**YES**	**911**	**59**			
**History of pelvic surgery for colon cancer**	**NO**	**4680**	**272**	**<0.001**	**1**	**<0.001**
	**YES**	**43**	**10**		**4.89 (2.38–10.02)**	
**The use of alpha- 1 blocker agent**	**NO**	**3780**	**210**	**0.024**	**1**	**0.003**
	**YES**	**943**	**72**		**1.55 (1.16–2.08)**	
**Past history of TURP**	**NO**	**4622**	**270**	**0.009**	**1**	**0.002**
	**YES**	**101**	**12**		**2.77 (1.46–5.13)**	
**The use of SSRI**	**NO**	**4645**	**272**	**0.022**	**1**	**0.030**
	**YES**	**78**	**10**		**2.12 (1.07–4.19)**	

TUR-P, Trans-urethral resection of the prostate; PPI, proton pump inhibitor; SSRI, selective serotonin reuptake inhibitors;

OR, odds ratio; CI, confidence interval

The results of the uni- and multivariate analyses identified independent factors of the presence of at least one sperm in urinary sediment.

### The association between types of alpha-1 blocker and the presence of at least one sperm in urinary sediment

Of the 1,015 patients treated with an alpha-1 blocker, 574 (56.6%), 278 (27.4%), 162 (16.0%), and 1 patient were treated with tamsulosin, silodosin, naftopidil, and urapidil, respectively. The detection rate of at least one sperm in the urinary sediment in patients treated with tamsulosin, silodosin, and naftopidil was 5.6% (32/574), 10.4% (29/278), and 6.8% (11/162), respectively. There were significant differences in the detection rate of at least one sperm in the urinary sediment between patients treated with and without silodosin (p = 0.011).

### The association between therapeutic type for diabetes mellitus and the presence of at least one sperm in urinary sediment

Among the diabetic patients, 1621 (97.5%) had type 2 diabetes and 42 (2.5%) type 1 diabetes. Their medical regimens were as follows: diet therapy alone, 35 (2.1%); oral hypoglycemic agent user, 1,007 (60.6%); and insulin user, 621 (37.3%).

The detection rate of at least one sperm in urinary sediment was 19.1% in type 1 diabetes patients, which was significantly higher than that in type 2 (7.8%, p<0.05). In addition the detection rate of at least one sperm in urinary sediment was 10.3% in patients treated with insulin, which was significantly higher than those treated with an oral hypoglycemic agent (6.3%, p = 0.001).

## Discussion

To the best of our knowledge, this is the first study to determine the incidence of at least one sperm in urinary sediment using a large number (more than 5,000) of patients and to evaluate the association between such a finding and clinical background factors. To date, the presence of sperm in urinary sediment has been treated as having little clinical significance and is usually seen in physiological conditions in which sperm has been incorporated into urine due to the mixing of semen components retained in the urethra following sexual activity or masturbation [2]. The present study demonstrated that 1.6% (339/20,937) of urinary sediment samples contained at least one sperm and that 5.6% (282/5,005) of general clinical practice patients who undergo urinary sediment examinations submitted samples that contain at least one sperm. Furthermore, the presence of at least one sperm in urinary sediment was found to be independently associated with diabetes, a history of pelvic surgery for colon cancer, a history of TURP, and alpha-1 blocker or SSRI use, which are known risk factors for ejaculation disorders [3,10]. We found that there is considerable overlap between the factors associated with the presence of at least one sperm in urinary sediment and those that are strongly associated with RE [11]. There have been many studies evaluating the medical factors associated with ejaculatory disorder such as RE. However, the problem is the definition of RE in the literature is not standardized and the rate of RE is subjectively evaluated by not-validated self-reported questionnaires in most of the studies [5,9]. For instance, in regard to the use of alpha-1 blockers, which is known to be a strong associated factor for RE, the incidence of RE has been reported to range widely from 0.9% to 28.1% due to different definitions for RE and the use of different methods for the evaluation of RE [6–8]. The primary goal of the present study was not to determine the incidence and risk factors of RE, but rather to identify what clinical background may have an association with “the presence of at least one sperm in urinary sediment” in real clinical practice. Interestingly, we found that associated factors in the case of “the presence of at least one sperm in urinary sediment” are similar to those related with so-called RE.

Accordingly, two hypotheses can be proposed for the close association between the presence of at least one sperm in urinary sediment and the abovementioned clinical factors. The first possibility is that chronic neurogenic impairments or direct damage to nerve paths results in the dysfunction of the internal sphincter of the urethra, leading to insufficient closure of the internal urethral orifice and the leakage of sperm into the posterior urethra [12,13]. In our study, the incidence of the presence of at least one sperm in urinary sediment was 8.1% (135/1,663) and 18.9% (10/53) in patients with diabetes and those who had undergone pelvic surgery for colorectal cancer, respectively. Diabetic autonomic neuropathy contributes to a wide spectrum of clinical disorders including ejaculation disorders [14–16] and is reported to affect about one-third of men with diabetes [15]. Furthermore, damage to the nerve paths involved in ejaculation is the main reason for ejaculation disorders in patients with a history of pelvic surgery for colorectal cancer [11]. Especially in diabetic patients, the type of treatment for diabetes is significantly associated with the detection rate of at least one sperm in urinary sediment in our present study. In fact, patients treated with insulin therapy had a significantly higher detection rate than those treated with an oral hypoglycemic agent. This might be explained in part by the speculation that the severity of diabetes could be associated with the detection rate of at least one sperm in urinary sediment. We plan to evaluate whether various factors, such as HbA1c level, duration of diabetes mellitus, and presence of diabetes-related complications could be associated with at least one sperm in urinary sediment in a future study.

The second explanation involves traumatic or drug-induced impairments that directly affect the closure of the internal urethral orifice of the bladder neck during ejaculation [10,11,17]. Our study demonstrated that the incidence rate of the presence of at least one sperm in urinary sediment was 7.1% (72/1,015), 11.4% (10/88), and 10.6% (12/113) in patients who used alpha-1 blockers, those who used SSRI, and those with a history of TURP, respectively. It has been reported that RE occurs in 0.9–28.1% of alpha-1 blocker users [6,7,18], and psychotropic drugs such as SSRI are associated with sexual dysfunction including erectile dysfunction, anorgasmia, and RE [19]. We further evaluated whether type of alpha-1 blocker could affect the incidence of at least one sperm in urinary sediment in our study population. We found that patients treated with silodosin had a significantly higher detection rate of at least one sperm in the urinary sediment, as compared to other types of alpha-1 blocker such as tamsulosin and naftopidil. Interestingly, the detection rate of at least one sperm in the urinary sediment in patients treated with silodosin was 10.4% in our study, which was lower than the incidence rate of RE (14.2%-28.1%) due to silodosin in previous reports [6–8]. Furthermore, RE is one of the main complications of TURP [11,20], and the incidence of RE after TURP varies from 36% to 100% depending on the degree of bladder neck resection [20,21].

The present study has several strengths. Firstly, it involved a large sample size (more than 5,000 patients), which reduced the risk of selection bias. Secondly, the sperm detection method used in this study was highly accurate; i.e., sperm was detected using a urinary element analyzer, and positive findings were confirmed by trained medical technicians who were unaware of the purpose of the study. Previously we compared the detection rate for the presence of sperm in urine examined using the automatic analyzer to that evaluated by a laboratory technician. With a sample size of 150 patients, 4 patients were found to have at least one sperm in urinary sediment by a laboratory technician and of these 4 the automatic analyzer could detect sperm in one patient, so the false negative rate by the analyzer was 75%. One hundred and forty-six patients were found to have no sperm in urinary sediment by the laboratory technician and the automatic analyzer could detect no sperm in these 146 patients, so the false positive rate by the analyzer was 0%. The overall concordance rate was 98%. However, the limitations of the present study should also be mentioned. Firstly, the total number of urinary sediment examinations differed among the subjects. Interestingly, among the patients in the sperm group, not all of their samples were found to contain sperm. Therefore, we only included patients who underwent urinalysis and urinary sedimentation testing at least twice during the observation period. The mean number of examinations per patient was 4.2, and 30.1±15.4% of the urinary sediment examinations included assessments of the presence/absence of sperm. Second, of seven independent indicators for the presence of at least one sperm in urinary sediment, four factors (younger age (<65), the total number of examinations (≥4), diabetes, and alpha-1 blocker use) were weak independent factors with odds ratio lower than 2. We cannot deny the possibility that these factors were extracted with some biases [22]. Thirdly, we do not have any data about the patients’ sexual activity such as whether the urine samples they provided were collected just after sexual intercourse or masturbation, which is strongly associated with the presence of sperm in urinary sediment. We routinely instructed the patients to discard their first urine samples and hand in their second urine samples because there is a high incidence of contamination due to debris normally present at the urethral opening in the first few drops. This could also minimize the chances of their samples being contaminated by sperm that had remained in the urethra.

## Conclusion

In conclusion, approximately 1.6% of all urinary sediment samples examined in daily clinical practice contain at least one sperm. There is considerable overlap between the factors associated with the presence of at least one sperm in urinary sediment and those that are strongly associated with ejaculatory disorders. These findings could help physicians to understand the clinical background of patients whose urinary sediment contains at least one sperm.

## Supporting Information

S1 File Background characteristics of participants. The patitens’ number (de-identified) and clinical parameters were described.
(PDF)
