# Supplementary material for: Clinical Background of Patients with Sperm in Their Urinary Sediment
Source: PLoS One. 2015 Sep 11;10(9):e0136844. doi: 10.1371/journal.pone.0136844 (PMC4567295; doi:10.1371/journal.pone.0136844)
Supplement: S1 File — The patitens’ number (de-identified) and clinical parameters were described. (PDF) [file pone.0136844.s001.pdf]

	times	freq		sperm	DM type	colon	HT	HL	anticoag
1	4		0	0	2	0	0	0	0
2	4		0	0	2	0	0	1	0
3	4		0	0	2	0	1	0	0
4	5		0	0	2	0	1	1	0
5	5		0	0	2	0	1	0	1
6	6		0	0	2	0	0	1	0
7	4		0	0	2	0	1	0	1
8	5		0	0	2	0	0	1	0
9	3		0	0	2	0	1	1	0
10	4		0	0	2	0	0	1	0
11	5		0	0	2	0	1	0	0
12	4		0	0	2	0	1	1	1
13	4		0	0	2	0	1	0	1
14	4		0	0	2	0	1	0	0
15	4		0	0	2	0	1	1	0
16	5		0	0	2	0	0	1	0
17	3		0	0	2	0	0	0	0
18	6		0	0	2	0	1	0	0
19	2		0	0	2	0	1	1	1
20	3		0	0	2	0	1	0	0
21	2		0	0	2	0	0	1	0
22	3		0	0	2	0	1	0	1
23	8		0	0	2	0	1	0	0
24	2		0	0	2	0	0	0	0
25	2		0	0	2	0	1	1	1
26	3		0	0	2	0	0	1	1
27	5	0.4	2	1	2	0	1	0	0
28	2	0.5	1	1	2	0	0	1	0
29	2	0.5	1	1	2	0	0	0	0
30	6	0.3333	2	1	2	1	0	0	0
31	8	1	8	1	2	0	0	0	0
32	3	0.3333	1	1	2	0	0	0	0
33	2	0.5	1	1	2	0	0	0	0
34	5	0.2	1	1	2	0	0	0	1
35	2	0.5	1	1	2	0	1	0	0
36	5	0.2	1	1	1	0	0	0	0
37	4		0	0	2	0	1	0	0
38	8		0	0	2	0	0	1	0
39	4		0	0	2	0	1	1	0
40	4		0	0	2	0	0	0	0
41	4		0	0	2	0	1	0	0

42	4	0	0	2	0	0	0	0
43	5	0	0	2	0	0	0	0
44	6	0	0	2	0	1	1	0
45	14	0	0	2	0	0	0	0
46	4	0	0	2	0	0	0	0
47	4	0	0	2	0	1	0	0
48	10	0	0	2	0	0	1	0
49	4	0	0	2	0	0	0	1
50	7	0	0	2	0	0	0	0
51	5	0	0	2	0	0	1	0
52	10	0	0	2	0	1	0	0
53	6	0	0	2	0	0	0	0
54	5	0	0	2	0	0	0	0
55	5	0	0	2	0	1	1	0
56	4	0	0	2	0	0	0	0
57	2	0	0	2	0	1	0	0
58	6	0	0	2	0	1	0	0
59	4	0	0	2	0	1	1	0
60	5	0	0	2	0	1	0	0
61	4	0	0	2	0	1	1	0
62	6	0	0	2	0	1	0	0
63	4	0	0	2	0	0	0	0
64	4	0	0	2	0	1	0	0
65	4	0	0	2	0	1	1	0
66	6	0	0	2	0	0	1	0
67	4	0	0	2	0	0	0	0
68	5	0	0	2	0	1	1	0
69	6	0	0	2	0	0	1	0
70	4	0	0	2	0	0	0	0
71	2	0	0	2	0	0	0	0
72	4	0	0	2	0	1	0	0
73	5	0	0	2	0	1	1	0
74	5	0	0	2	0	1	0	1
75	5	0	0	2	0	0	1	0
76	5	0	0	2	0	0	1	0
77	3	0	0	2	0	1	1	0
78	5	0	0	2	0	1	1	0
79	8	0	0	2	0	1	1	0
80	4	0	0	2	0	0	1	1
81	6	0	0	2	0	0	0	0
82	4	0	0	2	0	0	0	0
83	4	0	0	2	0	0	0	0
84	5	0	0	2	0	0	0	0

85	2	0	0	2	0	1	1	0
86	4	0	0	2	0	0	1	1
87	6	0	0	2	0	0	1	0
88	4	0	0	2	0	0	1	0
89	4	0	0	2	0	1	0	0
90	6	0	0	2	0	1	0	0
91	6	0	0	2	0	1	0	0
92	3	0	0	2	0	1	0	0
93	5	0	0	2	0	1	1	0
94	4	0	0	2	0	0	0	0
95	4	0	0	2	0	0	1	1
96	6	0	0	2	0	0	0	0
97	9	0	0	2	1	0	0	0
98	5	0	0	2	0	1	0	0
99	5	0	0	2	0	0	0	0
100	4	0	0	2	0	0	0	0
101	4	0	0	2	0	1	1	0
102	4	0	0	2	0	1	0	0
103	5	0	0	2	0	0	1	0
104	6	0	0	2	0	0	0	0
105	5	0	0	2	0	0	1	0
106	4	0	0	2	0	1	1	0
107	5	0	0	2	0	1	1	0
108	4	0	0	2	0	1	1	1
109	5	0	0	2	0	1	1	1
110	12	0	0	2	0	1	0	0
111	4	0	0	2	0	1	1	0
112	5	0	0	2	0	0	0	0
113	4	0	0	2	0	1	0	0
114	10	0	0	2	0	0	0	0
115	5	0	0	2	0	0	0	1
116	5	0	0	2	0	0	1	0
117	6	0	0	2	0	1	0	0
118	4	0	0	2	0	0	0	0
119	4	0	0	2	0	1	1	1
120	11	0	0	2	0	1	0	0
121	4	0	0	2	0	0	0	0
122	4	0	0	2	0	0	0	0
123	4	0	0	2	0	1	0	0
124	6	0	0	2	0	0	0	0
125	4	0	0	2	0	0	0	0
126	6	0	0	2	0	1	0	0
127	6	0	0	2	0	1	0	0

128	4	0	0	2	0	0	0	0
129	5	0	0	2	0	0	0	0
130	11	0	0	2	0	0	0	0
131	5	0	0	2	0	1	0	0
132	5	0	0	2	0	1	1	0
133	4	0	0	2	0	0	0	0
134	6	0	0	2	0	0	1	0
135	5	0	0	2	0	1	0	0
136	4	0	0	2	0	0	0	0
137	4	0	0	2	0	0	1	0
138	5	0	0	2	0	1	1	1
139	9	0	0	2	0	1	1	0
140	4	0	0	2	0	1	1	0
141	2	0	0	2	0	0	0	0
142	6	0	0	2	0	0	1	0
143	15	0	0	2	0	1	0	0
144	13	0	0	2	0	0	0	0
145	5	0	0	2	0	1	0	0
146	5	0	0	2	0	1	1	0
147	6	0	0	2	0	1	0	1
148	4	0	0	2	0	1	0	0
149	6	0	0	2	0	0	1	0
150	4	0	0	2	0	0	1	0
151	5	0	0	2	0	0	0	1
152	4	0	0	2	0	1	1	1
153	4	0	0	2	0	0	1	0
154	4	0	0	2	0	0	0	0
155	4	0	0	2	1	0	0	0
156	5	0	0	2	0	0	0	0
157	5	0	0	2	0	1	1	0
158	5	0	0	2	0	1	1	0
159	5	0	0	2	0	0	0	0
160	5	0	0	2	0	0	0	0
161	4	0	0	2	0	1	1	1
162	4	0	0	2	0	0	0	1
163	5	0	0	2	0	1	0	0
164	11	0	0	2	0	1	0	0
165	4	0	0	2	0	0	0	0
166	10	0	0	2	0	1	0	0
167	4	0	0	2	0	0	0	0
168	2	0	0	2	0	1	0	0
169	5	0	0	2	0	1	1	0

170	3	0	0	2	0	1	0	0
171	5	0	0	2	0	0	1	0
172	5	0	0	2	0	1	0	0
173	5	0	0	2	0	0	1	0
174	4	0	0	2	0	1	1	1
175	4	0	0	2	0	0	0	1
176	6	0	0	2	0	1	0	0
177	4	0	0	2	0	0	1	0
178	2	0	0	2	0	1	1	0
179	4	0	0	2	0	1	0	0
180	4	0	0	2	0	1	0	0
181	6	0	0	2	0	0	0	0
182	6	0	0	2	0	0	0	0
183	5	0	0	2	0	1	0	0
184	7	0	0	2	0	0	0	0
185	5	0	0	2	0	1	1	0
186	4	0	0	2	0	1	0	0
187	4	0	0	2	0	0	0	0
188	4	0	0	2	0	0	0	0
189	4	0	0	2	0	1	0	0
190	5	0	0	2	0	0	0	1
191	4	0	0	2	0	1	0	0
192	3	0	0	2	0	1	1	0
193	10	0	0	2	0	1	1	0
194	4	0	0	2	0	1	1	0
195	3	0	0	2	0	0	0	0
196	6	0	0	2	0	1	1	0
197	6	0	0	2	0	0	1	0
198	4	0	0	2	0	0	0	0
199	5	0	0	2	0	1	0	0
200	4	0	0	2	0	1	1	0
201	10	0	0	2	0	1	1	0
202	5	0	0	2	0	1	0	0
203	4	0	0	2	0	0	0	0
204	5	0	0	2	0	1	1	0
205	5	0	0	2	0	0	0	0
206	4	0	0	2	0	0	0	1
207	6	0	0	2	0	1	0	0
208	4	0	0	2	0	1	0	0
209	4	0	0	2	0	1	1	0
210	4	0	0	2	0	0	0	0
211	5	0	0	2	0	0	0	0

212	4	0	0	2	0	0	0	0
213	5	0	0	2	0	1	0	0
214	6	0	0	2	0	1	1	0
215	4	0	0	2	0	0	1	0
216	4	0	0	2	0	0	0	0
217	4	0	0	2	0	1	0	0
218	4	0	0	2	0	1	0	0
219	6	0	0	2	0	1	0	0
220	6	0	0	2	0	0	0	0
221	4	0	0	2	0	1	1	0
222	5	0	0	2	0	0	0	0
223	6	0	0	2	0	0	0	0
224	4	0	0	2	0	1	1	0
225	4	0	0	2	0	1	1	1
226	5	0	0	2	0	0	0	0
227	5	0	0	2	0	1	0	0
228	6	0	0	2	0	0	0	0
229	4	0	0	2	0	0	0	1
230	5	0	0	2	0	0	0	0
231	4	0	0	2	0	1	0	0
232	10	0	0	2	0	0	0	0
233	4	0	0	2	0	0	0	0
234	4	0	0	2	0	0	0	0
235	5	0	0	2	0	1	0	0
236	6	0	0	2	0	0	1	0
237	7	0	0	2	0	1	1	0
238	4	0	0	2	0	1	0	0
239	6	0	0	2	0	1	1	0
240	12	0	0	2	0	0	1	0
241	5	0	0	2	0	1	1	1
242	9	0	0	2	0	1	1	0
243	6	0	0	2	0	1	1	0
244	5	0	0	2	0	0	1	0
245	3	0	0	2	0	1	0	0
246	4	0	0	2	0	0	0	0
247	3	0	0	2	0	1	1	0
248	4	0	0	2	0	0	0	0
249	18	0	0	2	0	0	0	0
250	4	0	0	2	0	0	1	0
251	4	0	0	2	0	1	1	0
252	4	0	0	2	0	0	0	0
253	7	0	0	2	0	1	1	0

254	5	0	0	2	0	1	0	0
255	3	0	0	2	1	1	0	0
256	4	0	0	2	0	0	1	0
257	4	0	0	2	0	0	0	0
258	4	0	0	2	0	1	0	0
259	5	0	0	2	0	0	0	0
260	4	0	0	2	0	1	0	0
261	4	0	0	2	0	0	0	0
262	5	0	0	2	0	0	0	0
263	4	0	0	2	0	1	0	0
264	5	0	0	2	0	1	1	0
265	4	0	0	2	0	0	0	0
266	4	0	0	2	0	1	1	0
267	2	0	0	2	0	0	1	0
268	5	0	0	2	0	1	1	0
269	4	0	0	2	0	1	0	0
270	5	0	0	2	0	0	0	0
271	4	0	0	2	0	0	1	0
272	4	0	0	2	0	1	0	0
273	5	0	0	2	0	0	0	0
274	3	0	0	2	0	1	1	0
275	4	0	0	2	0	1	0	0
276	4	0	0	2	0	1	1	1
277	6	0	0	2	0	1	0	1
278	4	0	0	2	0	1	0	0
279	5	0	0	2	0	0	1	0
280	5	0	0	2	0	0	0	0
281	4	0	0	2	0	0	0	0
282	4	0	0	2	0	1	0	0
283	4	0	0	2	0	1	0	0
284	3	0	0	2	0	1	0	0
285	4	0	0	2	0	0	1	1
286	5	0	0	2	0	0	0	0
287	3	0	0	2	0	0	0	0
288	8	0	0	2	0	1	0	0
289	5	0	0	2	0	0	1	0
290	4	0	0	2	0	0	1	0
291	5	0	0	2	0	1	0	0
292	3	0	0	2	0	0	0	0
293	5	0	0	2	0	1	0	0
294	5	0	0	2	0	0	0	0
295	5	0	0	2	0	1	0	0

296	5	0	0	2	0	0	0	0
297	4	0	0	2	0	0	1	0
298	4	0	0	2	0	1	0	0
299	6	0	0	2	0	0	0	0
300	4	0	0	2	0	1	0	0
301	3	0	0	2	0	1	1	0
302	15	0	0	2	0	0	0	0
303	4	0	0	2	0	1	1	0
304	4	0	0	2	0	0	0	0
305	4	0	0	2	0	0	0	0
306	4	0	0	2	0	1	1	0
307	5	0	0	2	0	1	0	0
308	5	0	0	2	0	0	0	0
309	5	0	0	2	0	0	1	0
310	5	0	0	2	0	1	1	0
311	4	0	0	2	0	1	0	0
312	5	0	0	2	1	1	1	0
313	4	0	0	2	0	0	0	0
314	6	0	0	2	0	1	1	0
315	4	0	0	2	0	0	0	0
316	11	0	0	2	0	1	0	0
317	12	0	0	2	0	1	1	0
318	4	0	0	2	0	0	0	0
319	5	0	0	2	0	0	1	0
320	4	0	0	2	0	0	1	0
321	9	0	0	2	0	0	1	0
322	5	0	0	2	0	1	0	0
323	2	0	0	2	0	0	0	0
324	3	0	0	2	0	0	0	0
325	4	0	0	2	0	1	0	0
326	9	0	0	2	0	0	1	0
327	11	0	0	2	0	1	0	0
328	6	0	0	2	0	0	0	0
329	3	0	0	2	0	1	1	1
330	4	0	0	2	0	1	0	0
331	3	0	0	2	0	0	0	0
332	5	0	0	2	0	1	0	0
333	3	0	0	2	0	1	0	0
334	3	0	0	2	0	0	0	0
335	4	0	0	2	0	1	0	0
336	3	0	0	2	0	0	1	0
337	3	0	0	2	0	0	0	0

338	9	0	0	2	0	1	1	0
339	4	0	0	2	0	0	1	0
340	3	0	0	2	0	0	0	0
341	10	0	0	2	0	0	0	0
342	6	0	0	2	0	1	0	0
343	5	0	0	2	0	0	1	0
344	4	0	0	2	0	1	0	0
345	6	0	0	2	0	1	1	0
346	5	0	0	2	0	1	0	0
347	4	0	0	2	0	1	0	1
348	6	0	0	2	0	0	1	0
349	4	0	0	2	0	1	0	0
350	5	0	0	2	0	0	0	0
351	5	0	0	2	0	1	0	0
352	4	0	0	2	0	1	0	0
353	4	0	0	2	0	0	0	0
354	5	0	0	2	0	0	1	0
355	4	0	0	2	0	1	0	0
356	6	0	0	2	0	1	0	0
357	4	0	0	2	0	1	1	0
358	4	0	0	2	0	1	1	0
359	4	0	0	2	0	0	0	0
360	6	0	0	2	0	1	0	0
361	4	0	0	2	0	1	1	1
362	5	0	0	2	0	0	0	0
363	5	0	0	2	0	0	1	0
364	5	0	0	2	0	0	1	0
365	5	0	0	2	0	0	0	0
366	6	0	0	2	0	1	0	0
367	5	0	0	2	0	0	0	0
368	3	0	0	2	0	0	0	0
369	3	0	0	2	0	1	0	0
370	5	0	0	2	0	1	0	0
371	10	0	0	2	0	0	0	0
372	4	0	0	2	0	0	0	0
373	4	0	0	2	0	1	1	0
374	2	0	0	2	0	1	1	1
375	4	0	0	2	0	0	1	1
376	4	0	0	2	0	1	0	0
377	5	0	0	2	0	0	0	0
378	4	0	0	2	0	0	0	0
379	4	0	0	2	0	0	0	0

380	2	0	0	2	0	1	0	0
381	4	0	0	2	0	0	1	0
382	4	0	0	2	0	0	0	0
383	4	0	0	2	0	0	0	0
384	4	0	0	2	0	0	0	0
385	4	0	0	2	0	1	0	0
386	4	0	0	2	0	0	0	0
387	9	0	0	2	0	1	1	10
388	3	0	0	2	0	0	0	0
389	4	0	0	2	0	1	1	0
390	3	0	0	2	0	0	0	1
391	3	0	0	2	0	0	0	0
392	9	0	0	2	0	0	0	0
393	6	0	0	2	0	1	0	0
394	6	0	0	2	0	0	0	0
395	3	0	0	2	0	0	1	0
396	5	0	0	2	0	1	0	0
397	4	0	0	2	0	1	1	0
398	4	0	0	2	0	0	0	0
399	4	0	0	2	0	0	0	0
400	6	0	0	2	0	0	1	0
401	7	0	0	2	0	0	0	0
402	4	0	0	2	0	0	0	0
403	4	0	0	2	0	1	0	0
404	3	0	0	2	0	1	1	0
405	4	0	0	2	0	0	0	0
406	3	0	0	2	0	0	1	0
407	6	0	0	2	0	1	1	0
408	3	0	0	2	0	1	1	0
409	4	0	0	2	0	0	0	0
410	4	0	0	2	0	0	0	0
411	3	0	0	2	0	1	1	0
412	3	0	0	2	0	1	0	0
413	4	0	0	2	0	0	1	0
414	4	0	0	2	0	1	0	0
415	4	0	0	2	0	0	1	0
416	2	0	0	2	0	1	0	0
417	5	0	0	2	0	1	1	0
418	5	0	0	2	0	0	0	0
419	4	0	0	2	0	1	1	0
420	3	0	0	2	0	0	0	0
421	5	0	0	2	0	1	0	0
422	4	0	0	2	0	1	1	0

423	4	0	0	2	0	1	0	0
424	4	0	0	2	0	1	1	0
425	3	0	0	2	0	0	1	0
426	5	0	0	2	0	0	0	0
427	2	0	0	2	0	1	0	0
428	4	0	0	2	0	0	1	0
429	4	0	0	2	0	1	1	1
430	8	0	0	2	0	0	1	0
431	4	0	0	2	0	0	0	0
432	5	0	0	2	0	1	0	0
433	4	0	0	2	0	0	0	1
434	5	0	0	2	0	1	1	0
435	4	0	0	2	0	1	1	0
436	5	0	0	2	0	1	0	0
437	5	0	0	2	0	1	1	0
438	4	0	0	2	0	1	1	0
439	4	0	0	2	0	0	1	0
440	6	0	0	2	0	0	1	0
441	5	0	0	2	0	1	1	0
442	4	0	0	2	0	1	0	1
443	2	0	0	2	0	0	0	0
444	6	0	0	2	0	1	0	0
445	6	0	0	2	0	1	0	0
446	4	0	0	2	0	1	0	0
447	9	0	0	2	0	1	1	0
448	4	0	0	2	0	1	0	0
449	5	0	0	2	0	1	1	0
450	3	0	0	2	0	0	0	0
451	4	0	0	2	0	1	0	0
452	6	0	0	2	0	1	1	0
453	3	0	0	2	0	1	1	0
454	5	0	0	2	0	0	0	0
455	5	0	0	2	0	1	0	0
456	5	0	0	2	0	0	1	0
457	4	0	0	2	0	0	1	0
458	5	0	0	2	0	0	1	0
459	4	0	0	2	0	1	0	0
460	4	0	0	2	0	1	1	0
461	3	0	0	2	0	1	1	0
462	3	0	0	2	0	0	0	0
463	5	0	0	2	0	0	1	0
464	6	0	0	2	0	0	1	0

465	4	0	0	2	0	0	0	0
466	4	0	0	2	0	0	0	0
467	4	0	0	2	0	1	1	0
468	4	0	0	2	0	0	0	0
469	3	0	0	2	0	0	0	0
470	4	0	0	2	0	0	1	1
471	5	0	0	2	0	1	0	0
472	6	0	0	2	0	0	0	0
473	5	0	0	2	1	1	1	0
474	4	0	0	2	0	0	1	1
475	6	0	0	2	0	0	0	0
476	5	0	0	2	0	1	0	0
477	3	0	0	2	0	0	1	0
478	4	0	0	2	0	0	0	0
479	2	0	0	2	0	0	0	0
480	2	0	0	2	0	0	0	0
481	4	0	0	2	0	0	0	0
482	5	0	0	2	0	0	1	0
483	3	0	0	2	0	0	0	0
484	5	0	0	2	0	0	1	0
485	4	0	0	2	0	0	1	0
486	2	0	0	2	0	1	0	0
487	5	0	0	2	0	0	0	0
488	4	0	0	2	0	1	0	0
489	6	0	0	2	0	0	0	0
490	2	0	0	2	0	0	0	0
491	3	0	0	2	0	0	0	0
492	3	0	0	2	0	1	1	0
493	3	0	0	2	0	1	0	0
494	2	0	0	2	0	1	0	0
495	4	0	0	2	0	1	0	0
496	4	0	0	2	0	0	0	0
497	10	0	0	2	0	0	0	0
498	6	0	0	2	0	0	0	0
499	6	0	0	2	0	1	0	0
500	11	0	0	2	0	0	0	0
501	3	0	0	2	0	1	0	0
502	3	0	0	2	0	1	0	0
503	2	0	0	2	0	0	0	0
504	3	0	0	2	0	1	1	0
505	3	0	0	2	0	1	0	0
506	4	0	0	2	0	1	1	0

507	4	0	0	2	0	1	0	0
508	3	0	0	2	0	1	0	0
509	3	0	0	2	0	1	0	0
510	4	0	0	2	0	0	1	0
511	3	0	0	2	0	1	0	0
512	3	0	0	2	0	1	0	0
513	3	0	0	2	0	0	0	0
514	5	0	0	2	0	1	1	0
515	3	0	0	2	0	1	0	1
516	6	0	0	2	0	0	0	0
517	5	0	0	2	0	0	0	0
518	5	0	0	2	0	0	1	0
519	6	0	0	2	0	1	0	0
520	4	0	0	2	0	0	0	0
521	5	0	0	2	0	1	0	0
522	7	0	0	2	0	0	1	0
523	6	0	0	2	0	0	0	0
524	4	0	0	2	0	0	0	0
525	4	0	0	2	0	1	0	0
526	2	0	0	2	0	1	0	0
527	6	0	0	2	0	0	0	0
528	5	0	0	2	0	1	1	0
529	2	0	0	2	0	0	0	0
530	2	0	0	2	0	1	0	0
531	6	0	0	2	0	0	1	0
532	7	0	0	2	0	0	0	0
533	5	0	0	2	0	0	0	0
534	12	0	0	2	0	1	0	0
535	10	0	0	2	0	0	1	0
536	3	0	0	2	0	0	0	0
537	4	0	0	2	0	1	1	0
538	4	0	0	2	0	1	0	0
539	6	0	0	2	0	1	1	1
540	6	0	0	2	0	1	1	1
541	6	0	0	2	0	1	1	1
542	5	0	0	2	0	0	1	1
543	4	0	0	2	0	1	1	1
544	4	0	0	2	0	0	0	1
545	10	0	0	2	0	1	1	1
546	5	0	0	2	0	1	0	1
547	6	0	0	2	0	1	0	1

548	5	0	0	2	0	1	1	1
549	2	0	0	2	0	1	1	1
550	5	0	0	2	0	0	0	1
551	5	0	0	2	0	1	1	1
552	2	0	0	2	0	1	0	1
553	6	0	0	2	0	0	1	1
554	5	0	0	2	0	0	0	1
555	4	0	0	2	0	1	1	1
556	5	0	0	2	0	0	1	1
557	3	0	0	2	0	1	1	1
558	2	0	0	2	0	1	1	1
559	5	0	0	2	0	1	1	1
560	9	0	0	2	0	0	1	1
561	4	0	0	2	0	0	0	1
562	5	0	0	2	0	1	1	1
563	5	0	0	2	0	1	1	1
564	6	0	0	2	0	1	1	1
565	4	0	0	2	0	1	1	1
566	5	0	0	2	0	1	1	1
567	4	0	0	2	0	1	1	1
568	4	0	0	2	0	0	0	1
569	7	0	0	2	0	1	0	1
570	13	0	0	2	0	1	0	1
571	5	0	0	2	0	1	1	1
572	6	0	0	2	0	1	1	1
573	4	0	0	2	0	1	1	1
574	4	0	0	2	0	1	1	1
575	9	0	0	2	0	0	0	1
576	5	0	0	2	0	1	0	1
577	4	0	0	2	0	1	1	1
578	5	0	0	2	0	1	1	1
579	4	0	0	2	0	1	0	1
580	6	0	0	2	0	1	1	1
581	5	0	0	2	0	1	0	1
582	4	0	0	2	0	1	1	1
583	6	0	0	2	0	1	1	1
584	5	0	0	2	0	1	1	1
585	10	0	0	2	0	1	0	1
586	13	0	0	2	0	0	1	1
587	5	0	0	2	0	1	1	1
588	10	0	0	2	0	1	0	1
589	6	0	0	2	0	1	1	1

590	4	0	0	2	0	1	1	1
591	4	0	0	2	0	1	1	1
592	6	0	0	2	0	1	1	1
593	5	0	0	2	0	1	1	1
594	6	0	0	2	0	1	1	1
595	2	0	0	2	1	1	0	1
596	2	0	0	2	0	1	0	1
597	4	0	0	2	0	1	1	1
598	9	0	0	2	0	0	0	1
599	4	0	0	2	0	1	1	1
600	4	0	0	2	0	1	1	1
601	6	0	0	2	0	1	1	1
602	5	0	0	2	0	0	0	1
603	6	0	0	2	0	1	1	1
604	4	0	0	2	0	0	1	1
605	5	0	0	2	0	0	0	1
606	3	0	0	2	0	1	1	1
607	5	0	0	2	0	0	1	1
608	11	0	0	2	0	1	1	1
609	7	0	0	2	0	1	1	1
610	4	0	0	2	0	1	0	1
611	5	0	0	2	0	1	1	1
612	5	0	0	2	0	1	1	1
613	5	0	0	2	0	0	1	1
614	5	0	0	2	0	1	1	1
615	5	0	0	2	0	0	1	1
616	5	0	0	2	0	1	1	1
617	4	0	0	2	0	0	0	1
618	5	0	0	2	0	0	1	1
619	4	0	0	2	0	1	0	1
620	5	0	0	2	0	1	1	1
621	5	0	0	2	0	1	0	1
622	4	0	0	2	0	1	0	1
623	3	0	0	2	0	1	0	1
624	5	0	0	2	0	0	1	1
625	5	0	0	2	0	1	1	1
626	3	0	0	2	0	1	1	1
627	3	0	0	2	0	1	1	0
628	4	0	0	2	0	0	1	1
629	4	0	0	2	0	1	1	1
630	5	0	0	2	0	1	1	1
631	5	0	0	2	0	0	0	1
632	3	0	0	2	0	0	1	0

633	7	0	0	2	0	1	1	0
634	5	0	0	2	0	1	1	0
635	7	0	0	2	0	1	0	0
636	8	0	0	2	0	1	0	0
637	5	0	0	2	0	1	1	0
638	5	0	0	2	0	0	0	0
639	6	0	0	2	0	1	1	1
640	4	0	0	2	0	0	0	1
641	5	0	0	2	0	0	1	0
642	4	0	0	2	0	1	1	0
643	5	0	0	2	0	1	1	1
644	3	0	0	2	0	1	0	0
645	4	0	0	2	0	1	1	0
646	5	0	0	2	0	1	0	0
647	5	0	0	2	0	1	1	0
648	5	0	0	2	0	0	0	0
649	4	0	0	2	0	1	1	0
650	5	0	0	2	0	1	0	0
651	4	0	0	2	0	1	1	0
652	4	0	0	2	0	1	1	1
653	4	0	0	2	0	1	1	0
654	5	0	0	2	0	1	1	1
655	9	0	0	2	0	0	0	0
656	4	0	0	2	0	1	0	1
657	14	0	0	2	0	0	1	0
658	4	0	0	2	0	1	1	1
659	6	0	0	2	0	1	1	0
660	8	0	0	2	0	1	1	0
661	2	0	0	2	0	1	0	0
662	5	0	0	2	0	0	0	0
663	4	0	0	2	0	0	0	1
664	6	0	0	2	0	0	0	0
665	6	0	0	2	0	1	0	1
666	6	0	0	2	0	0	1	0
667	4	0	0	2	0	0	0	0
668	4	0	0	2	0	1	0	0
669	6	0	0	2	0	1	0	0
670	5	0	0	2	0	1	0	0
671	5	0	0	2	0	1	0	0
672	5	0	0	2	0	1	1	0
673	4	0	0	2	0	1	0	0
674	4	0	0	2	0	1	0	0

675	4	0	0	2	0	1	0	0
676	4	0	0	2	0	1	0	1
677	5	0	0	2	0	0	1	0
678	4	0	0	2	0	0	1	0
679	4	0	0	2	0	1	1	0
680	6	0	0	2	0	0	0	0
681	3	0	0	2	0	1	1	0
682	3	0	0	2	0	0	0	0
683	5	0	0	2	0	0	1	0
684	9	0	0	2	0	1	0	0
685	3	0	0	2	0	1	0	0
686	4	0	0	2	0	1	0	0
687	5	0	0	2	0	0	0	0
688	8	0	0	2	0	1	0	0
689	4	0	0	2	0	0	1	0
690	3	0	0	2	0	0	1	0
691	6	0	0	2	0	1	1	0
692	6	0	0	2	0	1	0	0
693	6	0	0	2	0	0	0	0
694	9	0	0	2	0	1	0	0
695	5	0	0	2	0	0	0	0
696	4	0	0	2	0	0	0	0
697	4	0	0	2	0	1	0	0
698	5	0	0	2	0	1	0	0
699	2	0	0	2	0	1	1	1
700	3	0	0	2	0	0	1	0
701	4	0	0	2	0	0	0	0
702	8	0	0	2	0	1	0	0
703	3	0	0	2	0	1	0	0
704	4	0	0	2	0	1	0	0
705	3	0	0	2	0	0	0	0
706	4	0	0	2	0	1	0	0
707	4	0	0	2	0	1	1	0
708	6	0	0	2	0	1	0	0
709	3	0	0	2	0	0	1	0
710	2	0	0	2	0	1	1	1
711	4	0	0	2	0	0	0	0
712	4	0	0	2	0	1	0	0
713	5	0	0	2	0	0	0	0
714	5	0	0	2	0	1	1	1
715	4	0	0	2	0	1	1	0
716	4	0	0	2	0	1	0	0

717	2	0	0	2	0	1	0	0
718	5	0	0	2	0	1	0	0
719	3	0	0	2	0	0	0	0
720	6	0	0	2	0	0	0	0
721	5	0	0	2	0	1	0	0
722	4	0	0	2	0	1	0	0
723	7	0	0	2	0	0	0	0
724	10	0	0	2	0	1	0	0
725	3	0	0	2	0	1	1	0
726	5	0	0	2	0	1	0	0
727	3	0	0	2	0	0	1	0
728	6	0	0	2	0	0	0	0
729	5	0	0	2	0	0	0	0
730	7	0	0	2	0	1	1	0
731	9	0	0	2	0	0	0	0
732	4	0	0	2	0	1	1	0
733	10	0	0	2	0	1	1	1
734	5	0	0	2	0	1	1	1
735	17	0	0	2	0	1	0	1
736	10	0	0	2	0	1	0	1
737	5	0	0	2	0	1	0	1
738	2	0	0	2	0	1	1	1
739	5	0	0	2	0	1	0	1
740	3	0	0	2	0	1	1	1
741	5	0	0	2	0	0	0	1
742	7	0	0	2	0	1	1	1
743	6	0	0	2	0	1	1	1
744	4	0	0	2	0	1	1	1
745	6	0	0	2	0	1	1	1
746	5	0	0	2	0	0	0	1
747	4	0	0	2	0	0	0	1
748	5	0	0	2	0	1	1	1
749	11	0	0	2	0	1	1	1
750	9	0	0	2	0	1	1	1
751	16	0	0	2	0	0	0	1
752	6	0	0	2	0	1	0	1
753	4	0	0	2	0	1	0	1
754	11	0	0	2	0	0	1	1
755	6	0	0	2	0	1	1	1
756	11	0	0	2	0	1	0	0
757	9	0	0	2	0	1	1	1
758	6	0	0	2	0	1	1	1

759	10	0	0	2	0	1	0	1
760	6	0	0	2	0	1	1	1
761	5	0	0	2	0	1	0	1
762	4	0	0	2	0	1	1	1
763	5	0	0	2	0	1	0	1
764	5	0	0	2	0	1	0	1
765	5	0	0	2	0	0	1	1
766	3	0	0	2	0	1	1	1
767	6	0	0	2	0	1	1	1
768	6	0	0	2	0	1	1	1
769	5	0	0	2	0	1	1	1
770	4	0	0	2	0	1	0	1
771	6	0	0	2	0	1	1	1
772	2	0	0	2	0	0	1	1
773	2	0	0	2	0	1	1	1
774	4	0	0	2	0	1	1	1
775	4	0	0	2	0	1	0	1
776	10	0	0	2	0	1	1	1
777	8	0	0	2	0	1	1	1
778	3	0	0	2	0	1	0	1
779	4	0	0	2	0	1	0	1
780	6	0	0	2	0	1	1	1
781	2	0	0	2	0	1	0	1
782	7	0	0	2	0	1	0	1
783	4	0	0	2	0	1	1	1
784	6	0	0	2	0	1	1	1
785	2	0	0	2	0	1	1	1
786	6	0	0	2	0	1	0	1
787	5	0	0	2	0	1	0	1
788	5	0	0	2	0	1	0	1
789	3	0	0	2	0	0	0	0
790	3	0	0	2	0	1	1	0
791	3	0	0	2	0	0	0	0
792	6	0	0	2	0	1	1	1
793	13	0	0	2	0	1	1	1
794	2	0	0	2	0	1	0	1
795	2	0	0	2	0	0	0	0
796	8	0	0	2	0	1	1	0
797	2	0	0	2	0	0	0	0
798	7	0	0	2	0	0	1	0
799	2	0	0	2	0	1	0	0
800	3	0	0	2	0	1	0	1

801	7	0	0	2	0	0	1	0
802	2	0	0	2	0	1	1	1
803	8	0	0	2	0	1	0	0
804	3	0	0	2	0	0	0	0
805	3	0	0	2	0	0	0	0
806	8	0	0	2	0	1	0	0
807	8	0	0	2	0	1	1	0
808	2	0	0	2	0	0	1	0
809	2	0	0	2	0	1	0	0
810	3	0	0	2	0	0	0	1
811	3	0	0	2	0	0	0	0
812	3	0	0	2	0	1	0	1
813	2	0	0	2	0	1	1	0
814	3	0	0	2	0	0	0	0
815	2	0	0	2	0	0	0	0
816	2	0	0	2	0	1	0	0
817	8	0	0	2	0	0	1	0
818	3	0	0	2	0	0	0	0
819	2	0	0	2	0	1	0	0
820	3	0	0	2	0	0	0	0
821	8	0	0	2	0	1	0	0
822	3	0	0	2	0	0	1	0
823	3	0	0	2	0	1	0	0
824	2	0	0	2	0	1	1	0
825	8	0	0	2	0	1	0	0
826	2	0	0	2	0	0	0	0
827	3	0	0	2	0	0	0	0
828	8	0	0	2	0	1	0	0
829	2	0	0	2	0	0	0	0
830	2	0	0	2	0	0	0	0
831	3	0	0	2	0	1	0	0
832	3	0	0	2	0	1	0	0
833	3	0	0	2	0	0	0	0
834	7	0	0	2	0	0	0	0
835	3	0	0	2	0	1	1	0
836	2	0	0	2	0	1	0	0
837	3	0	0	2	0	1	0	0
838	7	0	0	2	0	1	0	0
839	3	0	0	2	0	1	1	0
840	8	0	0	2	0	0	1	0
841	2	0	0	2	0	1	1	0

842	2	0	0	2	0	0	0	0
843	2	0	0	2	0	1	1	1
844	7	0	0	2	0	0	0	0
845	7	0	0	2	0	1	1	0
846	2	0	0	2	0	0	0	0
847	2	0	0	2	0	0	0	0
848	2	0	0	2	0	0	0	0
849	2	0	0	2	0	1	0	0
850	2	0	0	2	0	0	1	0
851	2	0	0	2	1	1	1	0
852	2	0	0	2	0	1	1	0
853	2	0	0	2	0	1	1	0
854	2	0	0	2	0	0	1	0
855	8	0	0	2	0	1	1	0
856	2	0	0	2	0	1	0	0
857	2	0	0	2	0	1	0	0
858	2	0	0	2	0	0	0	0
859	7	0	0	2	0	0	1	0
860	2	0	0	2	0	1	0	0
861	2	0	0	2	0	1	1	0
862	2	0	0	2	0	0	0	0
863	2	0	0	2	0	0	0	0
864	2	0	0	2	0	0	0	0
865	7	0	0	2	0	0	0	0
866	2	0	0	2	0	0	0	0
867	2	0	0	2	0	1	1	0
868	2	0	0	2	0	0	0	0
869	2	0	0	2	0	1	1	0
870	2	0	0	2	0	1	1	0
871	2	0	0	2	0	0	0	0
872	2	0	0	2	0	0	0	0
873	2	0	0	2	0	0	0	0
874	2	0	0	2	0	0	1	0
875	2	0	0	2	0	1	0	0
876	7	0	0	2	0	0	0	0
877	8	0	0	2	0	1	1	0
878	2	0	0	2	0	1	0	0
879	7	0	0	2	0	1	1	0
880	2	0	0	2	0	1	0	0
881	7	0	0	2	0	0	0	0
882	7	0	0	2	0	1	1	0
883	2	0	0	2	0	1	0	0

884	2	0	0	2	0	0	1	1
885	7	0	0	2	0	1	0	0
886	7	0	0	2	0	0	1	0
887	8	0	0	2	0	0	0	0
888	2	0	0	2	0	1	1	0
889	2	0	0	2	0	1	1	0
890	2	0	0	2	0	1	0	0
891	2	0	0	2	0	0	0	0
892	2	0	0	2	0	0	0	0
893	2	0	0	2	0	1	1	0
894	2	0	0	2	0	1	0	0
895	2	0	0	2	0	0	1	0
896	7	0	0	2	0	1	0	0
897	2	0	0	2	0	0	1	0
898	2	0	0	2	0	0	0	0
899	2	0	0	2	0	0	0	0
900	8	0	0	2	0	0	0	0
901	2	0	0	2	0	1	0	0
902	2	0	0	2	0	1	1	0
903	7	0	0	2	0	0	0	0
904	2	0	0	2	0	1	0	1
905	3	0	0	2	0	1	1	1
906	3	0	0	2	0	1	1	1
907	8	0	0	2	0	1	1	1
908	3	0	0	2	0	1	1	1
909	3	0	0	2	0	0	0	1
910	2	0	0	2	0	1	0	1
911	2	0	0	2	0	0	1	1
912	7	0	0	2	0	1	1	1
913	2	0	0	2	0	1	0	1
914	7	0	0	2	0	1	1	1
915	7	0	0	2	0	1	1	1
916	2	0	0	2	0	1	0	1
917	7	0	0	2	0	1	1	1
918	7	0	0	2	0	0	0	1
919	7	0	0	2	0	1	1	1
920	2	0	0	2	0	0	0	1
921	2	0	0	2	0	1	1	1
922	2	0	0	2	0	1	0	1
923	2	0	0	2	0	1	1	1
924	3	0	0	2	0	0	1	1
925	7	0	0	2	0	1	1	1

926	7	0	0	2	0	1	0	0
927	3	0	0	2	0	1	0	0
928	2	0	0	2	0	0	0	0
929	7	0	0	2	0	1	1	0
930	2	0	0	2	0	1	1	0
931	2	0	0	2	0	1	1	0
932	7	0	0	2	0	0	0	0
933	3	0	0	2	0	1	1	0
934	3	0	0	2	0	1	0	0
935	3	0	0	2	0	1	1	0
936	3	0	0	2	0	0	0	0
937	3	0	0	2	0	0	1	0
938	8	0	0	2	0	1	0	0
939	2	0	0	2	0	1	1	0
940	2	0	0	2	0	1	0	0
941	7	0	0	2	0	0	0	0
942	2	0	0	2	0	1	1	0
943	2	0	0	2	0	0	0	0
944	7	0	0	2	0	1	0	0
945	2	0	0	2	0	1	0	0
946	8	0	0	2	0	1	0	0
947	7	0	0	2	0	0	1	0
948	2	0	0	2	0	1	0	0
949	2	0	0	2	0	1	1	0
950	8	0	0	2	0	1	0	1
951	3	0	0	2	0	1	1	1
952	7	0	0	2	0	0	0	1
953	3	0	0	2	0	0	1	1
954	3	0	0	2	0	1	1	1
955	7	0	0	2	0	1	1	1
956	2	0	0	2	0	1	1	1
957	2	0	0	2	0	1	1	1
958	2	0	0	2	0	1	1	1
959	3	0	0	2	0	0	1	1
960	8	0	0	2	0	1	1	1
961	7	0	0	2	0	1	0	1
962	8	0	0	2	0	1	0	1
963	2	0	0	2	0	0	1	1
964	2	0	0	2	0	1	1	1
965	2	0	0	2	0	1	1	1
966	2	0	0	2	0	1	1	1

967	8		0	0	2	0	1	0	1
968	8		0	0	2	0	1	1	1
969	7		0	0	2	0	1	0	1
970	8		0	0	2	0	0	0	1
971	2		0	0	2	0	1	0	1
972	8		0	0	2	0	1	1	1
973	2		0	0	2	0	1	1	1
974	2	0.5	1	1	2	0	1	1	0
975	7	0.1429	1	1	2	0	0	0	0
976	7	0.1429	1	1	2	0	0	0	0
977	5	0.2	1	1	2	0	0	1	0
978	5	0.2	1	1	2	0	0	1	0
979	5	0.2	1	1	2	0	1	0	0
980	5	0.2	1	1	2	0	0	0	0
981	7	0.1429	1	1	2	0	0	0	0
982	4	0.5	2	1	2	0	0	1	0
983	7	0.1429	1	1	2	0	0	0	0
984	4	0.25	1	1	2	0	1	0	0
985	12	0.1667	2	1	2	0	0	0	0
986	4	0.25	1	1	2	0	0	0	0
987	5	0.2	1	1	2	0	1	0	0
988	8	0.125	1	1	2	0	0	0	0
989	4	0.25	1	1	2	0	1	0	0
990	5	0.2	1	1	2	0	0	1	0
991	4	0.25	1	1	2	0	1	0	0
992	6	0.1667	1	1	2	0	0	1	0
993	6	0.1667	1	1	2	0	1	0	0
994	3	0.3333	1	1	2	0	1	1	1
995	5	0.2	1	1	2	0	1	0	0
996	8	0.125	1	1	2	0	0	0	0
997	5	0.2	1	1	2	0	1	0	0
998	5	0.2	1	1	2	0	1	0	0
999	2	1	2	1	2	0	1	0	0
1000	6	0.1667	1	1	2	0	0	1	0
1001	6	0.1667	1	1	2	0	1	0	0
1002	2	0.5	1	1	2	0	1	1	0
1003	5	0.2	1	1	2	0	1	0	0
1004	3	0.3333	1	1	2	0	0	0	0
1005	4	0.25	1	1	2	0	1	1	0
1006	4	0.25	1	1	2	0	1	0	0
1007	2	0.5	1	1	2	0	0	0	0

1008	2	0.5	1	1	2	0	0	0	0
1009	3	0.3333	1	1	2	0	1	1	1
1010	8	0.125	1	1	2	0	1	1	0
1011	3	0.3333	1	1	2	0	1	1	0
1012	3	0.3333	1	1	2	0	1	0	1
1013	7	0.1429	1	1	2	0	1	0	1
1014	6	0.3333	2	1	2	0	1	0	1
1015	5	0.2	1	1	2	0	1	1	1
1016	5	0.2	1	1	2	0	1	0	1
1017	5	0.2	1	1	2	0	0	1	1
1018	3	0.3333	1	1	2	0	1	1	0
1019	10	0.1	1	1	2	0	1	1	0
1020	2	0.5	1	1	2	0	1	0	0
1021	4	0.25	1	1	2	0	0	0	0
1022	4	0.25	1	1	2	0	0	0	0
1023	5	0.2	1	1	2	0	1	1	0
1024	6	0.3333	2	1	2	0	1	0	0
1025	8	0.25	2	1	2	1	1	0	0
1026	4	0.25	1	1	2	0	0	1	0
1027	3	0.3333	1	1	2	0	1	0	0
1028	11	0.3636	4	1	2	0	1	0	0
1029	4	0.25	1	1	2	0	1	1	1
1030	3	0.3333	1	1	2	0	1	1	1
1031	10	0.1	1	1	2	0	1	1	1
1032	4	0.25	1	1	2	0	1	1	1
1033	8	0.375	3	1	2	0	1	0	1
1034	4	0.25	1	1	2	0	1	1	1
1035	4		0	0	2	0	1	0	0
1036	4		0	0	2	0	0	0	0
1037	4		0	0	2	0	0	0	1
1038	4		0	0	2	0	1	1	0
1039	5		0	0	2	0	0	0	1
1040	6		0	0	2	0	1	0	0
1041	2		0	0	1	0	1	1	0
1042	6		0	0	1	0	0	0	0
1043	6		0	0	1	0	0	1	0
1044	5		0	0	1	0	0	0	0
1045	3		0	0	1	0	0	0	0
1046	3		0	0	1	0	1	1	0
1047	5		0	0	1	0	1	0	0
1048	6		0	0	1	0	0	0	0
1049	6		0	0	1	0	1	0	0

1050	6		0	0	1	0	0	0	0
1051	5		0	0	1	0	1	1	0
1052	6		0	0	1	0	0	0	0
1053	6		0	0	1	0	0	1	0
1054	6		0	0	1	0	0	0	0
1055	6		0	0	1	0	0	0	0
1056	6		0	0	1	0	1	0	0
1057	6		0	0	1	0	0	1	0
1058	6		0	0	1	0	0	0	0
1059	2		0	0	1	0	0	0	0
1060	5		0	0	1	0	0	0	0
1061	2		0	0	1	0	1	1	1
1062	4		0	0	1	0	0	1	1
1063	6		0	0	1	0	0	0	1
1064	6		0	0	1	0	1	0	1
1065	3		0	0	1	0	1	1	1
1066	2		0	0	1	0	0	1	1
1067	5		0	0	1	0	0	0	1
1068	5		0	0	1	0	1	1	0
1069	7		0	0	1	0	0	0	0
1070	3		0	0	1	1	1	1	0
1071	4		0	0	1	0	1	1	0
1072	4		0	0	1	0	1	1	1
1073	2		0	0	1	0	1	0	1
1074	3	0.3333	1	1	1	0	1	0	0
1075	4	0.25	1	1	1	0	0	0	0
1076	5	0.2	1	1	1	0	1	1	0
1077	5	0.4	2	1	1	0	1	0	0
1078	5	0.2	1	1	1	0	0	0	0
1079	6	0.1667	1	1	1	0	1	1	1
1080	5	0.2	1	1	1	0	1	0	0
1081	5		0	0	1	0	0	1	0
1082	6		0	0	2	0	0	0	0
1083	4		0	0	2	0	1	1	0
1084	6		0	0	2	0	1	0	1
1085	5		0	0	2	0	1	0	1
1086	4		0	0	2	0	1	0	0
1087	4		0	0	2	0	0	0	1
1088	6		0	0	2	0	0	0	1
1089	5		0	0	2	0	1	1	0
1090	4		0	0	2	0	1	0	0
1091	5		0	0	2	0	1	1	0

1092	6	0	0	2	0	1	1	0
1093	5	0	0	2	0	0	0	0
1094	6	0	0	2	0	0	0	0
1095	2	0	0	2	0	0	0	0
1096	4	0	0	2	0	0	0	0
1097	5	0	0	2	0	1	1	0
1098	4	0	0	2	0	0	0	0
1099	5	0	0	2	0	1	0	0
1100	5	0	0	2	0	0	0	0
1101	5	0	0	2	0	1	0	0
1102	5	0	0	2	0	1	0	1
1103	6	0	0	2	0	1	0	0
1104	4	0	0	2	0	1	1	1
1105	6	0	0	2	0	1	0	0
1106	4	0	0	2	0	1	1	1
1107	5	0	0	2	0	1	0	1
1108	5	0	0	2	0	0	1	0
1109	4	0	0	2	0	0	1	0
1110	5	0	0	2	0	1	0	0
1111	5	0	0	2	0	1	0	0
1112	6	0	0	2	0	1	0	0
1113	6	0	0	2	0	1	0	1
1114	4	0	0	2	0	1	0	0
1115	6	0	0	2	0	1	0	0
1116	4	0	0	2	0	0	0	0
1117	4	0	0	2	0	0	0	0
1118	5	0	0	2	0	0	1	0
1119	5	0	0	2	0	1	1	1
1120	4	0	0	2	0	0	0	0
1121	6	0	0	2	0	1	1	0
1122	5	0	0	2	0	0	0	0
1123	4	0	0	2	0	1	0	0
1124	5	0	0	2	0	1	0	0
1125	5	0	0	2	0	1	0	0
1126	6	0	0	2	0	0	0	0
1127	4	0	0	2	0	0	0	0
1128	4	0	0	2	0	1	0	0
1129	6	0	0	2	0	1	0	0
1130	4	0	0	2	0	1	0	0
1131	15	0	0	2	0	1	0	0
1132	9	0	0	2	0	1	0	0
1133	4	0	0	2	0	0	1	1
1134	6	0	0	2	0	0	0	0

1135	4	0	0	2	0	0	1	0
1136	5	0	0	2	1	1	1	0
1137	4	0	0	2	0	0	1	0
1138	4	0	0	2	0	1	0	0
1139	5	0	0	2	0	1	0	0
1140	5	0	0	2	1	0	0	0
1141	6	0	0	2	0	1	0	0
1142	4	0	0	2	0	0	0	0
1143	6	0	0	2	0	1	0	0
1144	3	0	0	2	0	1	1	0
1145	4	0	0	2	0	1	1	0
1146	6	0	0	2	1	0	0	0
1147	4	0	0	2	0	1	0	0
1148	5	0	0	2	0	1	1	0
1149	3	0	0	2	0	1	0	0
1150	4	0	0	2	0	0	0	0
1151	7	0	0	2	0	0	0	0
1152	4	0	0	2	0	1	0	0
1153	2	0	0	2	0	1	0	1
1154	4	0	0	2	0	0	1	0
1155	6	0	0	2	0	1	0	0
1156	5	0	0	2	0	1	0	1
1157	2	0	0	2	0	0	0	0
1158	4	0	0	2	0	1	0	0
1159	10	0	0	2	0	1	1	0
1160	4	0	0	2	0	1	0	0
1161	5	0	0	2	0	1	0	1
1162	5	0	0	2	0	1	1	0
1163	6	0	0	2	0	1	0	0
1164	4	0	0	2	0	0	0	0
1165	6	0	0	2	0	0	0	0
1166	14	0	0	2	0	0	0	0
1167	6	0	0	2	0	1	1	0
1168	4	0	0	2	0	0	0	0
1169	4	0	0	2	0	0	0	0
1170	4	0	0	2	0	1	0	0
1171	7	0	0	2	0	1	0	0
1172	4	0	0	2	0	1	0	1
1173	5	0	0	2	0	0	1	0
1174	4	0	0	2	0	1	0	0
1175	4	0	0	2	0	1	0	0
1176	4	0	0	2	0	0	0	0

1177	4	0	0	2	0	0	1	0
1178	4	0	0	2	0	0	1	0
1179	5	0	0	2	0	0	1	0
1180	11	0	0	2	0	1	0	0
1181	6	0	0	2	0	1	1	0
1182	4	0	0	2	0	1	0	0
1183	3	0	0	2	0	0	0	0
1184	10	0	0	2	0	0	0	0
1185	6	0	0	2	0	0	0	0
1186	5	0	0	2	0	0	0	0
1187	2	0	0	2	0	1	0	0
1188	4	0	0	2	0	0	1	0
1189	6	0	0	2	0	1	0	0
1190	6	0	0	2	0	1	1	0
1191	5	0	0	2	0	1	0	0
1192	6	0	0	2	0	0	0	0
1193	4	0	0	2	0	1	1	1
1194	4	0	0	2	0	1	0	0
1195	4	0	0	2	0	1	0	0
1196	3	0	0	2	0	1	0	0
1197	4	0	0	2	0	1	0	0
1198	5	0	0	2	0	0	0	0
1199	5	0	0	2	0	1	1	0
1200	3	0	0	2	0	1	1	0
1201	6	0	0	2	0	0	1	0
1202	6	0	0	2	0	0	0	0
1203	5	0	0	2	0	0	1	0
1204	6	0	0	2	0	1	1	
1205	5	0	0	2	0	1	1	0
1206	3	0	0	2	0	1	0	0
1207	6	0	0	2	0	1	0	0
1208	2	0	0	2	0	0	0	0
1209	6	0	0	2	0	1	1	0
1210	4	0	0	2	0	1	1	0
1211	6	0	0	2	0	1	1	0
1212	3	0	0	2	0	1	0	0
1213	5	0	0	2	0	1	1	0
1214	5	0	0	2	0	0	0	0
1215	3	0	0	2	0	1	1	1
1216	4	0	0	2	0	1	0	0
1217	4	0	0	2	1	1	0	0
1218	4	0	0	2	0	0	0	0

1219	10	0	0	2	0	1	1	1
1220	5	0	0	2	0	0	1	0
1221	6	0	0	2	0	1	0	0
1222	6	0	0	2	0	1	1	0
1223	11	0	0	2	0	0	0	0
1224	4	0	0	2	0	1	0	0
1225	4	0	0	2	0	0	0	0
1226	4	0	0	2	0	0	0	0
1227	6	0	0	2	0	1	0	0
1228	6	0	0	2	0	1	1	0
1229	3	0	0	2	1	1	0	0
1230	3	0	0	2	0	1	0	0
1231	3	0	0	2	0	0	0	0
1232	3	0	0	2	0	0	0	0
1233	4	0	0	2	0	0	0	0
1234	6	0	0	2	0	1	0	0
1235	5	0	0	2	0	1	1	0
1236	3	0	0	2	0	0	0	0
1237	11	0	0	2	0	1	1	0
1238	5	0	0	2	0	1	0	0
1239	3	0	0	2	0	1	1	0
1240	5	0	0	2	0	0	0	0
1241	4	0	0	2	0	1	0	0
1242	3	0	0	2	0	1	1	0
1243	5	0	0	2	0	0	1	0
1244	4	0	0	2	0	1	0	1
1245	3	0	0	2	0	1	1	0
1246	5	0	0	2	0	0	1	0
1247	5	0	0	2	0	1	1	0
1248	4	0	0	2	0	0	0	0
1249	4	0	0	2	0	1	0	0
1250	4	0	0	2	0	1	0	0
1251	6	0	0	2	0	1	1	0
1252	3	0	0	2	0	0	0	0
1253	3	0	0	2	0	0	0	0
1254	6	0	0	2	0	1	1	0
1255	4	0	0	2	0	0	1	0
1256	5	0	0	2	0	1	1	0
1257	3	0	0	2	0	0	0	0
1258	4	0	0	2	0	1	0	0
1259	3	0	0	2	0	0	0	0
1260	3	0	0	2	0	1	0	0

1261	4	0	0	2	0	0	1	0
1262	3	0	0	2	0	1	1	0
1263	4	0	0	2	0	0	1	0
1264	3	0	0	2	0	1	0	0
1265	6	0	0	2	0	1	1	0
1266	4	0	0	2	0	1	0	0
1267	3	0	0	2	0	0	0	0
1268	3	0	0	2	0	0	1	0
1269	4	0	0	2	0	1	0	0
1270	5	0	0	2	0	0	1	0
1271	4	0	0	2	0	0	0	0
1272	3	0	0	2	0	1	0	0
1273	6	0	0	2	0	0	1	0
1274	3	0	0	2	0	0	1	0
1275	5	0	0	2	0	1	1	0
1276	4	0	0	2	0	1	0	1
1277	4	0	0	2	0	0	0	0
1278	4	0	0	2	0	0	0	0
1279	6	0	0	2	0	1	1	0
1280	5	0	0	2	0	0	0	0
1281	3	0	0	2	0	1	0	0
1282	3	0	0	2	0	0	0	0
1283	4	0	0	2	0	1	1	0
1284	4	0	0	2	0	0	0	0
1285	4	0	0	2	0	0	0	0
1286	4	0	0	2	0	0	0	0
1287	10	0	0	2	0	1	1	0
1288	6	0	0	2	0	1	1	0
1289	3	0	0	2	0	1	0	0
1290	5	0	0	2	0	0	0	0
1291	4	0	0	2	0	0	0	0
1292	4	0	0	2	0	0	0	1
1293	4	0	0	2	0	1	0	0
1294	4	0	0	2	0	1	0	0
1295	3	0	0	2	0	0	0	0
1296	3	0	0	2	0	1	0	0
1297	3	0	0	2	0	0	0	0
1298	9	0	0	2	0	1	0	0
1299	22	0	0	2	0	1	0	0
1300	2	0	0	2	0	1	0	0
1301	2	0	0	2	0	0	0	0
1302	3	0	0	2	0	0	0	0

1303	3	0	0	2	0	1	0	0
1304	6	0	0	2	0	0	0	0
1305	4	0	0	2	0	1	0	0
1306	3	0	0	2	0	1	0	0
1307	3	0	0	2	0	1	0	0
1308	5	0	0	2	0	1	1	0
1309	5	0	0	2	0	1	0	0
1310	6	0	0	2	0	0	0	0
1311	5	0	0	2	0	0	0	1
1312	6	0	0	2	0	1	1	1
1313	10	0	0	2	0	1	0	1
1314	11	0	0	2	0	1	1	1
1315	11	0	0	2	0	1	1	1
1316	4	0	0	2	0	1	0	0
1317	4	0	0	2	0	1	0	1
1318	6	0	0	2	0	1	1	1
1319	4	0	0	2	0	0	0	1
1320	12	0	0	2	0	1	1	1
1321	4	0	0	2	0	0	1	1
1322	6	0	0	2	0	1	0	1
1323	5	0	0	2	0	1	1	1
1324	5	0	0	2	0	1	0	1
1325	10	0	0	2	0	1	1	1
1326	5	0	0	2	0	1	0	1
1327	5	0	0	2	0	1	1	1
1328	6	0	0	2	0	1	0	1
1329	6	0	0	2	0	1	1	1
1330	4	0	0	2	0	1	1	1
1331	9	0	0	2	0	1	0	1
1332	4	0	0	2	0	1	0	1
1333	10	0	0	2	0	1	1	1
1334	11	0	0	2	0	1	0	1
1335	6	0	0	2	0	1	1	1
1336	6	0	0	2	0	1	1	1
1337	6	0	0	2	0	1	1	1
1338	6	0	0	2	0	1	1	1
1339	5	0	0	2	0	1	1	0
1340	3	0	0	2	0	1	1	1
1341	3	0	0	2	0	1	1	1
1342	5	0	0	2	0	1	1	0
1343	4	0	0	2	0	1	1	1
1344	5	0	0	2	0	1	1	1

1345	5	0	0	2	0	1	0	1
1346	5	0	0	2	0	1	0	1
1347	4	0	0	2	0	1	1	1
1348	6	0	0	2	0	1	1	1
1349	5	0	0	2	0	1	1	1
1350	3	0	0	2	0	1	1	1
1351	5	0	0	2	0	1	1	1
1352	5	0	0	2	0	1	1	1
1353	2	0	0	2	0	1	0	1
1354	5	0	0	2	0	1	1	1
1355	6	0	0	2	0	0	1	1
1356	6	0	0	2	0	1	1	1
1357	5	0	0	2	0	1	1	1
1358	3	0	0	2	0	1	0	1
1359	4	0	0	2	0	1	1	1
1360	5	0	0	2	0	1	1	1
1361	4	0	0	2	0	1	0	1
1362	5	0	0	2	0	1	0	1
1363	5	0	0	2	0	1	1	1
1364	6	0	0	2	0	1	1	1
1365	5	0	0	2	0	1	0	1
1366	2	0	0	2	0	1	1	1
1367	5	0	0	2	0	1	1	1
1368	10	0	0	2	0	1	1	0
1369	16	0	0	2	0	0	1	0
1370	4	0	0	2	0	1	1	1
1371	5	0	0	2	0	1	0	0
1372	19	0	0	2	0	0	0	0
1373	4	0	0	2	0	0	1	0
1374	4	0	0	2	0	1	1	0
1375	5	0	0	2	0	1	1	0
1376	19	0	0	2	0	1	1	0
1377	5	0	0	2	0	0	0	0
1378	4	0	0	2	0	1	0	0
1379	4	0	0	2	0	0	0	0
1380	2	0	0	2	0	0	1	0
1381	10	0	0	2	0	1	1	0
1382	16	0	0	2	0	0	0	0
1383	6	0	0	2	0	1	1	0
1384	4	0	0	2	0	1	0	0
1385	5	0	0	2	0	0	0	0
1386	5	0	0	2	0	1	0	0

1387	6	0	0	2	0	1	0	0
1388	5	0	0	2	0	1	0	0
1389	6	0	0	2	0	1	1	0
1390	5	0	0	2	0	0	0	0
1391	12	0	0	2	0	1	0	0
1392	4	0	0	2	0	1	1	0
1393	5	0	0	2	0	1	0	0
1394	7	0	0	2	0	1	0	0
1395	5	0	0	2	0	1	0	0
1396	5	0	0	2	0	1	1	0
1397	4	0	0	2	0	0	1	1
1398	5	0	0	2	0	0	0	0
1399	5	0	0	2	0	1	1	0
1400	4	0	0	2	0	1	0	0
1401	5	0	0	2	0	1	0	0
1402	4	0	0	2	0	1	1	0
1403	5	0	0	2	0	1	1	0
1404	2	0	0	2	0	1	0	1
1405	4	0	0	2	0	0	0	0
1406	4	0	0	2	0	1	0	0
1407	6	0	0	2	0	1	1	0
1408	5	0	0	2	0	1	0	0
1409	4	0	0	2	0	1	1	0
1410	2	0	0	2	1	0	0	0
1411	4	0	0	2	0	0	0	1
1412	2	0	0	2	0	0	0	0
1413	9	0	0	2	0	1	1	0
1414	6	0	0	2	0	1	1	0
1415	3	0	0	2	0	0	0	0
1416	3	0	0	2	0	1	1	1
1417	5	0	0	2	0	0	0	0
1418	2	0	0	2	0	1	1	1
1419	5	0	0	2	0	1	1	1
1420	6	0	0	2	0	1	1	1
1421	5	0	0	2	0	1	1	1
1422	4	0	0	2	0	1	1	1
1423	6	0	0	2	0	1	1	1
1424	5	0	0	2	0	1	1	1
1425	6	0	0	2	0	1	0	1
1426	9	0	0	2	0	1	1	1
1427	16	0	0	2	0	1	0	1
1428	12	0	0	2	0	1	1	1

1429	7	0	0	2	0	0	0	1
1430	7	0	0	2	0	1	1	1
1431	9	0	0	2	0	1	0	1
1432	5	0	0	2	0	1	1	1
1433	4	0	0	2	0	0	1	1
1434	4	0	0	2	0	0	0	1
1435	5	0	0	2	0	1	0	1
1436	5	0	0	2	0	1	0	1
1437	16	0	0	2	0	0	0	1
1438	13	0	0	2	0	1	1	1
1439	11	0	0	2	0	0	1	1
1440	3	0	0	2	0	1	1	1
1441	2	0	0	2	0	1	1	1
1442	5	0	0	2	0	1	1	1
1443	6	0	0	2	0	1	1	1
1444	5	0	0	2	0	1	1	1
1445	6	0	0	2	0	1	1	1
1446	4	0	0	2	0	1	1	1
1447	10	0	0	2	0	1	0	1
1448	3	0	0	2	0	1	1	1
1449	4	0	0	2	0	0	1	1
1450	10	0	0	2	0	0	1	1
1451	4	0	0	2	0	1	1	1
1452	2	0	0	2	0	0	0	0
1453	3	0	0	2	0	0	0	0
1454	2	0	0	2	0	0	0	0
1455	7	0	0	2	0	0	0	0
1456	2	0	0	2	0	1	0	0
1457	2	0	0	2	0	0	0	0
1458	7	0	0	2	0	1	1	0
1459	2	0	0	2	0	0	0	0
1460	7	0	0	2	0	0	1	0
1461	7	0	0	2	0	1	1	0
1462	7	0	0	2	0	0	1	0
1463	3	0	0	2	0	0	0	0
1464	3	0	0	2	0	1	1	0
1465	3	0	0	2	0	1	0	0
1466	7	0	0	2	0	0	0	0
1467	2	0	0	2	0	0	0	0
1468	3	0	0	2	0	0	0	0
1469	3	0	0	2	0	1	0	0
1470	8	0	0	2	0	0	0	0

1471	2	0	0	2	0	0	0	0
1472	2	0	0	2	0	0	1	0
1473	2	0	0	2	0	1	1	0
1474	3	0	0	2	0	0	0	0
1475	2	0	0	2	0	0	0	0
1476	3	0	0	2	0	1	0	0
1477	9	0	0	2	0	0	0	0
1478	3	0	0	2	0	0	1	0
1479	8	0	0	2	0	1	0	0
1480	8	0	0	2	0	0	0	0
1481	7	0	0	2	0	0	0	0
1482	2	0	0	2	0	0	0	0
1483	8	0	0	2	0	1	1	0
1484	3	0	0	2	0	0	0	0
1485	2	0	0	2	0	1	1	0
1486	2	0	0	2	0	0	0	0
1487	2	0	0	2	0	0	0	0
1488	8	0	0	2	0	1	0	0
1489	8	0	0	2	0	1	1	0
1490	3	0	0	2	0	1	0	0
1491	3	0	0	2	0	1	0	0
1492	3	0	0	2	0	0	0	0
1493	3	0	0	2	0	1	0	0
1494	7	0	0	2	0	1	0	0
1495	7	0	0	2	0	0	1	0
1496	2	0	0	2	0	0	0	1
1497	8	0	0	2	0	0	0	0
1498	3	0	0	2	0	1	0	0
1499	2	0	0	2	0	0	0	0
1500	2	0	0	2	0	0	1	0
1501	3	0	0	2	0	1	0	0
1502	2	0	0	2	0	0	0	0
1503	2	0	0	2	0	0	0	0
1504	8	0	0	2	0	0	0	0
1505	3	0	0	2	0	1	0	0
1506	7	0	0	2	0	1	0	0
1507	3	0	0	2	0	0	0	0
1508	3	0	0	2	0	0	0	0
1509	3	0	0	2	0	0	0	0
1510	7	0	0	2	0	0	0	0
1511	7	0	0	2	0	1	1	0
1512	2	0	0	2	0	0	0	0

1513	8	0	0	2	0	1	0	0
1514	2	0	0	2	0	1	0	0
1515	7	0	0	2	0	0	0	0
1516	7	0	0	2	0	1	0	0
1517	2	0	0	2	0	0	0	0
1518	2	0	0	2	0	1	1	0
1519	2	0	0	2	0	1	0	0
1520	7	0	0	2	0	0	0	0
1521	7	0	0	2	0	0	1	0
1522	7	0	0	2	0	1	1	0
1523	7	0	0	2	0	0	0	0
1524	7	0	0	2	0	1	0	0
1525	2	0	0	2	0	0	0	0
1526	7	0	0	2	0	1	1	0
1527	8	0	0	2	0	0	1	0
1528	7	0	0	2	0	1	0	0
1529	2	0	0	2	0	0	1	1
1530	8	0	0	2	0	1	0	0
1531	7	0	0	2	0	1	1	0
1532	2	0	0	2	0	0	0	0
1533	7	0	0	2	0	1	0	0
1534	2	0	0	2	0	0	1	0
1535	2	0	0	2	0	1	1	0
1536	7	0	0	2	0	0	0	0
1537	2	0	0	2	0	0	0	0
1538	7	0	0	2	0	1	1	0
1539	7	0	0	2	0	0	1	0
1540	2	0	0	2	0	0	1	0
1541	2	0	0	2	0	1	1	0
1542	2	0	0	2	0	1	0	0
1543	2	0	0	2	0	0	0	0
1544	2	0	0	2	0	0	0	0
1545	2	0	0	2	0	0	0	0
1546	2	0	0	2	0	1	0	0
1547	2	0	0	2	0	1	0	0
1548	2	0	0	2	0	1	1	0
1549	7	0	0	2	0	0	0	0
1550	7	0	0	2	0	1	1	1
1551	2	0	0	2	0	1	1	1
1552	3	0	0	2	0	0	0	1
1553	7	0	0	2	0	1	1	1
1554	7	0	0	2	0	1	1	1

1555	2	0	0	2	0	1	1	1
1556	7	0	0	2	0	1	1	1
1557	3	0	0	2	0	0	0	1
1558	7	0	0	2	0	1	1	1
1559	3	0	0	2	0	1	1	1
1560	8	0	0	2	0	1	0	1
1561	3	0	0	2	0	1	0	1
1562	3	0	0	2	0	1	1	1
1563	3	0	0	2	0	1	1	1
1564	3	0	0	2	0	1	1	1
1565	2	0	0	2	0	1	1	1
1566	8	0	0	2	0	0	0	1
1567	2	0	0	2	0	1	1	1
1568	2	0	0	2	0	1	1	1
1569	2	0	0	2	0	1	1	1
1570	2	0	0	2	0	1	0	0
1571	8	0	0	2	0	1	1	1
1572	8	0	0	2	0	1	1	1
1573	8	0	0	2	0	1	1	1
1574	2	0	0	2	0	1	1	1
1575	2	0	0	2	0	1	1	1
1576	2	0	0	2	0	0	0	0
1577	2	0	0	2	0	1	0	0
1578	7	0	0	2	0	1	1	0
1579	3	0	0	2	0	1	0	0
1580	8	0	0	2	0	0	0	0
1581	2	0	0	2	0	1	0	0
1582	3	0	0	2	1	1	1	0
1583	2	0	0	2	0	1	0	0
1584	2	0	0	2	0	1	1	0
1585	2	0	0	2	0	1	0	1
1586	2	0	0	2	0	0	1	0
1587	7	0	0	2	0	1	1	0
1588	7	0	0	2	0	0	0	0
1589	8	0	0	2	0	0	0	0
1590	2	0	0	2	0	0	0	0
1591	2	0	0	2	0	1	1	0
1592	2	0	0	2	0	0	0	0
1593	8	0	0	2	0	1	0	0
1594	3	0	0	2	0	1	0	1
1595	7	0	0	2	0	1	0	1
1596	7	0	0	2	0	1	0	1

1597	2		0	0	2	0	1	1	1
1598	3		0	0	2	0	1	1	1
1599	3		0	0	2	0	1	1	1
1600	8		0	0	2	0	1	1	1
1601	3		0	0	2	0	1	1	1
1602	2		0	0	2	0	1	1	1
1603	3		0	0	2	0	1	1	1
1604	7		0	0	2	0	1	0	1
1605	7		0	0	2	0	1	1	1
1606	7		0	0	2	0	1	1	1
1607	4	0.25	1	1	2	0	0	0	0
1608	3	0.3333	1	1	2	0	0	1	0
1609	4	0.25	1	1	2	0	1	1	0
1610	5	0.2	1	1	2	1	1	1	0
1611	5	0.2	1	1	2	0	1	0	0
1612	6	0.1667	1	1	2	0	0	0	0
1613	5	0.2	1	1	2	0	1	1	0
1614	4	0.5	2	1	2	0	0	0	0
1615	7	0.1429	1	1	2	0	1	1	0
1616	4	0.5	2	1	2	0	1	0	0
1617	6	0.1667	1	1	2	0	1	0	0
1618	2	0.5	1	1	2	0	1	1	0
1619	6	0.1667	1	1	2	0	1	1	0
1620	5	0.4	2	1	2	0	1	0	0
1621	6	0.1667	1	1	2	0	0	0	0
1622	4	0.25	1	1	2	0	0	1	0
1623	3	0.3333	1	1	2	0	0	0	0
1624	5	0.2	1	1	2	0	1	0	0
1625	5	0.4	2	1	2	0	1	1	1
1626	4	0.25	1	1	2	0	0	0	0
1627	4	0.25	1	1	2	0	0	0	0
1628	2	1	2	1	2	0	1	0	0
1629	5	0.2	1	1	2	0	0	1	0
1630	2	0.5	1	1	2	0	0	1	0
1631	4	0.25	1	1	2	0	0	0	0
1632	5	0.2	1	1	2	0	0	0	0
1633	10	0.1	1	1	2	0	0	1	0
1634	4	0.25	1	1	2	0	0	0	0
1635	2	0.5	1	1	2	0	0	0	0
1636	5	0.2	1	1	2	0	1	0	0
1637	2	0.5	1	1	2	0	1	1	1

1638	7	0.2857	2	1	2	0	1	0	1
1639	11	0.0909	1	1	2	1	1	0	1
1640	3	0.3333	1	1	2	0	1	1	1
1641	4	0.25	1	1	2	0	1	1	1
1642	3	0.3333	1	1	2	0	0	1	1
1643	12	0.25	3	1	2	0	1	1	1
1644	8	0.125	1	1	2	0	0	0	1
1645	2	0.5	1	1	2	0	1	1	1
1646	5	0.2	1	1	2	0	1	1	0
1647	8	0.25	2	1	2	0	1	1	1
1648	8	0.125	1	1	2	0	1	0	0
1649	5	0.2	1	1	2	0	1	1	1
1650	3	0.3333	1	1	2	0	1	1	0
1651	6	0.3333	2	1	2	0	1	1	0
1652	5	0.2	1	1	2	0	1	0	1
1653	5	0.2	1	1	2	0	1	1	0
1654	3	0.6667	2	1	2	0	1	1	1
1655	9	0.2222	2	1	2	0	1	1	1
1656	6	0.1667	1	1	2	0	0	1	1
1657	10	0.1	1	1	2	0	1	1	1
1658	2	0.5	1	1	2	0	1	1	1
1659	3	0.3333	1	1	2	0	1	1	1
1660	8	0.125	1	1	2	0	1	1	1
1661	4	0.25	1	1	2	0	0	0	1
1662	8	0.25	2	1	2	0	0	0	1
1663	4	0.25	1	1	2	0	1	1	0
1664	5		0	0		0	0	0	0
1665	2		0	0		0	0	0	0
1666	3		0	0		0	0	0	0
1667	2		0	0		0	0	0	0
1668	3		0	0		0	0	0	0
1669	2		0	0		0	0	0	0
1670	6		0	0		0	0	0	0
1671	2		0	0		0	0	0	0
1672	2		0	0		0	0	0	0
1673	2		0	0		0	0	0	0
1674	2		0	0		0	0	0	0
1675	2		0	0		0	0	0	0
1676	2		0	0		0	0	0	0
1677	2		0	0		0	1	0	0
1678	2		0	0		0	0	0	0

1679	2	0	0	0	0	0	0
1680	2	0	0	0	0	0	0
1681	2	0	0	0	0	0	0
1682	3	0	0	0	1	0	0
1683	3	0	0	0	0	0	0
1684	3	0	0	0	1	0	0
1685	3	0	0	0	0	0	0
1686	3	0	0	0	0	0	0
1687	2	0	0	0	0	0	0
1688	4	0	0	0	0	0	1
1689	6	0	0	0	0	0	0
1690	4	0	0	0	0	0	0
1691	6	0	0	0	0	0	0
1692	12	0	0	0	0	0	0
1693	2	0	0	0	0	0	0
1694	2	0	0	0	0	0	0
1695	8	0	0	0	1	0	0
1696	2	0	0	0	0	0	0
1697	4	0	0	0	0	0	0
1698	4	0	0	0	1	0	0
1699	5	0	0	0	0	0	0
1700	4	0	0	0	1	0	0
1701	3	0	0	0	0	0	0
1702	4	0	0	0	0	0	0
1703	3	0	0	0	0	0	0
1704	3	0	0	0	0	0	0
1705	8	0	0	0	0	0	0
1706	3	0	0	0	0	0	0
1707	4	0	0	0	1	0	0
1708	6	0	0	0	0	1	0
1709	2	0	0	0	0	0	0
1710	3	0	0	0	1	0	0
1711	3	0	0	0	0	0	0
1712	4	0	0	0	0	0	0
1713	4	0	0	0	0	0	0
1714	3	0	0	0	0	0	0
1715	2	0	0	0	0	0	0
1716	4	0	0	0	0	1	0
1717	3	0	0	0	0	0	0
1718	3	0	0	0	1	0	0
1719	2	0	0	0	0	0	0
1720	4	0	0	0	1	0	0

1721	6	0	0	0	1	0	0
1722	3	0	0	0	0	0	0
1723	2	0	0	0	0	0	0
1724	5	0	0	0	1	0	0
1725	2	0	0	0	0	0	0
1726	4	0	0	0	1	1	0
1727	12	0	0	0	0	0	0
1728	6	0	0	0	0	0	0
1729	3	0	0	0	0	0	0
1730	3	0	0	0	0	0	0
1731	2	0	0	0	0	0	0
1732	4	0	0	0	0	0	0
1733	7	0	0	0	1	1	0
1734	2	0	0	0	0	0	0
1735	3	0	0	0	0	0	0
1736	2	0	0	0	1	0	0
1737	2	0	0	0	0	0	0
1738	2	0	0	0	0	0	0
1739	2	0	0	0	0	0	0
1740	2	0	0	0	0	0	0
1741	4	0	0	0	1	0	0
1742	3	0	0	0	0	0	0
1743	2	0	0	0	0	0	0
1744	2	0	0	0	1	1	0
1745	3	0	0	0	1	1	1
1746	4	0	0	0	0	1	0
1747	4	0	0	0	0	0	0
1748	3	0	0	0	0	0	0
1749	3	0	0	0	1	0	0
1750	3	0	0	0	0	0	0
1751	3	0	0	0	0	0	0
1752	2	0	0	0	0	0	0
1753	2	0	0	0	0	0	0
1754	3	0	0	0	0	0	0
1755	23	0	0	0	1	0	0
1756	3	0	0	0	1	0	0
1757	2	0	0	0	0	0	0
1758	4	0	0	0	1	0	0
1759	2	0	0	0	0	0	0
1760	2	0	0	0	0	0	0
1761	4	0	0	0	0	0	0
1762	3	0	0	0	0	0	0

1763	4	0	0	0	1	1	0
1764	2	0	0	0	0	0	0
1765	4	0	0	0	0	0	0
1766	2	0	0	0	0	0	0
1767	3	0	0	0	0	0	0
1768	4	0	0	0	0	0	0
1769	6	0	0	0	0	0	0
1770	4	0	0	0	0	0	0
1771	3	0	0	0	0	0	0
1772	2	0	0	0	0	0	0
1773	3	0	0	0	0	0	0
1774	2	0	0	0	0	0	0
1775	3	0	0	0	1	0	0
1776	2	0	0	0	0	0	0
1777	5	0	0	0	1	0	0
1778	2	0	0	0	0	0	0
1779	2	0	0	0	0	0	0
1780	2	0	0	0	0	0	0
1781	2	0	0	0	0	0	0
1782	2	0	0	0	0	0	0
1783	3	0	0	0	0	0	0
1784	2	0	0	0	0	0	0
1785	3	0	0	0	0	0	0
1786	3	0	0	0	0	0	0
1787	3	0	0	0	0	0	0
1788	2	0	0	0	0	0	0
1789	3	0	0	0	0	0	0
1790	2	0	0	0	0	0	0
1791	4	0	0	0	0	0	0
1792	2	0	0	0	0	0	0
1793	3	0	0	0	1	0	0
1794	2	0	0	1	0	0	0
1795	4	0	0	0	1	0	1
1796	5	0	0	0	0	1	0
1797	2	0	0	0	0	0	0
1798	7	0	0	0	0	0	0
1799	3	0	0	0	0	0	0
1800	2	0	0	0	0	0	0
1801	4	0	0	0	0	0	0
1802	2	0	0	0	0	0	0
1803	2	0	0	0	0	0	0

1804	2	0	0	0	0	0	0
1805	4	0	0	0	0	0	0
1806	4	0	0	1	0	0	0
1807	4	0	0	0	0	0	0
1808	2	0	0	0	1	0	0
1809	2	0	0	0	0	0	0
1810	3	0	0	0	0	0	0
1811	4	0	0	0	1	0	0
1812	2	0	0	0	0	0	0
1813	3	0	0	0	0	0	0
1814	2	0	0	0	0	0	0
1815	5	0	0	0	0	1	0
1816	7	0	0	0	0	0	0
1817	3	0	0	0	0	1	0
1818	2	0	0	0	0	0	0
1819	4	0	0	0	0	0	0
1820	3	0	0	0	0	0	0
1821	2	0	0	0	0	0	0
1822	3	0	0	0	0	0	0
1823	3	0	0	0	0	0	0
1824	2	0	0	0	0	0	0
1825	4	0	0	0	0	1	0
1826	3	0	0	0	0	0	0
1827	2	0	0	0	0	1	0
1828	3	0	0	0	0	0	0
1829	3	0	0	0	0	0	0
1830	3	0	0	0	0	0	0
1831	3	0	0	0	0	0	0
1832	2	0	0	0	1	0	0
1833	3	0	0	0	0	0	0
1834	4	0	0	0	0	0	0
1835	4	0	0	0	0	0	1
1836	3	0	0	0	0	0	0
1837	4	0	0	0	0	0	0
1838	3	0	0	0	0	0	0
1839	4	0	0	0	1	0	0
1840	2	0	0	0	0	0	0
1841	2	0	0	0	0	0	0
1842	3	0	0	0	0	0	0
1843	3	0	0	0	1	0	0
1844	2	0	0	0	0	0	0

1845	3	0	0	0	0	0	0
1846	12	0	0	0	1	1	1
1847	3	0	0	0	0	0	0
1848	4	0	0	0	0	0	0
1849	2	0	0	0	0	0	0
1850	4	0	0	0	0	0	0
1851	2	0	0	0	0	0	0
1852	3	0	0	0	0	0	0
1853	4	0	0	0	0	1	0
1854	4	0	0	0	0	1	0
1855	4	0	0	0	1	1	0
1856	7	0	0	0	0	0	0
1857	2	0	0	0	0	0	0
1858	8	0	0	0	0	0	0
1859	3	0	0	0	1	0	0
1860	4	0	0	0	0	0	0
1861	4	0	0	0	1	1	0
1862	2	0	0	0	0	0	0
1863	5	0	0	0	1	0	0
1864	2	0	0	0	0	0	0
1865	2	0	0	0	0	0	0
1866	2	0	0	0	0	0	0
1867	2	0	0	0	0	0	0
1868	2	0	0	0	0	0	0
1869	4	0	0	0	1	0	0
1870	2	0	0	0	1	0	0
1871	4	0	0	0	0	0	0
1872	2	0	0	0	0	0	0
1873	2	0	0	0	0	0	0
1874	3	0	0	0	0	1	0
1875	2	0	0	0	0	0	0
1876	4	0	0	0	0	1	0
1877	2	0	0	0	1	1	0
1878	3	0	0	0	1	0	1
1879	2	0	0	0	0	0	0
1880	2	0	0	0	0	0	0
1881	6	0	0	0	0	0	0
1882	2	0	0	0	0	0	0
1883	2	0	0	0	0	0	0
1884	2	0	0	0	0	0	0
1885	2	0	0	0	0	0	0

1886	5	0	0	0	0	0	0
1887	2	0	0	0	0	1	0
1888	4	0	0	0	1	1	0
1889	2	0	0	0	1	0	0
1890	2	0	0	0	1	0	0
1891	3	0	0	0	0	0	0
1892	3	0	0	0	0	0	0
1893	4	0	0	0	0	0	0
1894	2	0	0	0	0	0	0
1895	2	0	0	0	1	0	0
1896	3	0	0	0	0	0	0
1897	4	0	0	0	0	0	0
1898	3	0	0	0	0	0	0
1899	3	0	0	0	0	0	0
1900	3	0	0	0	0	0	0
1901	4	0	0	0	0	0	0
1902	4	0	0	0	0	0	0
1903	8	0	0	0	0	0	0
1904	4	0	0	0	0	0	0
1905	4	0	0	0	0	0	0
1906	2	0	0	0	0	0	0
1907	3	0	0	0	0	0	0
1908	3	0	0	1	0	0	0
1909	3	0	0	0	0	0	0
1910	4	0	0	0	0	0	0
1911	2	0	0	0	0	0	0
1912	12	0	0	0	0	0	0
1913	3	0	0	0	0	0	0
1914	4	0	0	0	0	0	0
1915	5	0	0	0	0	0	0
1916	5	0	0	0	1	0	0
1917	5	0	0	0	0	0	0
1918	3	0	0	0	0	0	0
1919	2	0	0	0	0	0	0
1920	2	0	0	0	0	0	0
1921	4	0	0	0	1	1	0
1922	3	0	0	0	0	0	0
1923	3	0	0	0	0	0	0
1924	3	0	0	0	0	0	0
1925	15	0	0	0	0	0	0
1926	3	0	0	0	0	0	0
1927	4	0	0	0	1	1	0

1928	4	0	0	0	0	1	0
1929	3	0	0	0	0	0	0
1930	3	0	0	0	0	0	0
1931	3	0	0	0	0	0	0
1932	4	0	0	0	1	0	0
1933	4	0	0	0	0	0	0
1934	2	0	0	0	1	0	0
1935	2	0	0	0	1	1	1
1936	3	0	0	0	1	1	0
1937	3	0	0	0	0	0	0
1938	5	0	0	0	0	0	0
1939	6	0	0	0	0	0	0
1940	3	0	0	0	0	0	0
1941	2	0	0	0	1	0	1
1942	3	0	0	0	0	0	0
1943	3	0	0	0	0	0	0
1944	2	0	0	0	0	0	0
1945	3	0	0	0	0	0	0
1946	3	0	0	0	0	0	0
1947	3	0	0	1	0	0	0
1948	3	0	0	0	0	0	0
1949	3	0	0	0	0	0	0
1950	2	0	0	0	0	0	0
1951	5	0	0	0	0	0	0
1952	2	0	0	0	0	0	0
1953	3	0	0	0	0	0	0
1954	3	0	0	0	0	0	0
1955	5	0	0	0	0	0	0
1956	2	0	0	0	0	0	0
1957	2	0	0	0	0	0	0
1958	4	0	0	0	0	0	0
1959	5	0	0	0	0	0	0
1960	5	0	0	0	0	0	0
1961	2	0	0	0	0	0	0
1962	5	0	0	0	1	1	0
1963	2	0	0	0	0	0	0
1964	2	0	0	0	0	0	0
1965	2	0	0	0	0	0	0
1966	2	0	0	0	0	0	0
1967	4	0	0	0	0	0	0
1968	2	0	0	0	0	0	0

1969	2	0	0	0	0	0	0
1970	2	0	0	0	0	0	0
1971	2	0	0	0	0	0	0
1972	2	0	0	0	0	0	0
1973	3	0	0	0	0	0	0
1974	2	0	0	0	0	0	0
1975	2	0	0	0	0	0	0
1976	2	0	0	0	0	0	0
1977	8	0	0	0	0	0	0
1978	2	0	0	0	0	0	0
1979	2	0	0	0	0	0	0
1980	2	0	0	0	0	0	0
1981	2	0	0	0	0	0	0
1982	2	0	0	0	1	0	0
1983	2	0	0	0	0	0	0
1984	2	0	0	0	0	0	0
1985	2	0	0	0	0	0	0
1986	2	0	0	0	0	0	0
1987	2	0	0	0	0	0	0
1988	4	0	0	0	0	0	0
1989	2	0	0	0	0	0	0
1990	2	0	0	0	0	0	0
1991	2	0	0	0	0	0	0
1992	2	0	0	0	0	0	0
1993	2	0	0	0	0	0	0
1994	2	0	0	0	0	0	0
1995	2	0	0	0	0	0	0
1996	2	0	0	0	0	0	0
1997	2	0	0	0	0	0	0
1998	2	0	0	0	0	0	0
1999	2	0	0	0	0	0	0
2000	2	0	0	0	0	0	0
2001	2	0	0	0	0	0	0
2002	2	0	0	0	0	0	0
2003	2	0	0	0	0	0	0
2004	2	0	0	0	0	0	0
2005	2	0	0	0	0	0	0
2006	2	0	0	0	0	0	0
2007	4	0	0	0	0	0	0

2008	9	0	0	0	0	1	0
2009	4	0	0	0	1	0	0
2010	2	0	0	0	0	0	0
2011	5	0	0	0	0	0	0
2012	4	0	0	0	1	0	0
2013	2	0	0	0	0	0	0
2014	4	0	0	0	0	0	0
2015	6	0	0	0	0	0	0
2016	4	0	0	0	0	0	0
2017	2	0	0	0	0	0	0
2018	2	0	0	0	0	0	0
2019	2	0	0	0	0	0	0
2020	2	0	0	0	0	0	0
2021	2	0	0	0	0	0	0
2022	2	0	0	0	0	0	0
2023	2	0	0	0	1	1	0
2024	5	0	0	0	1	1	0
2025	3	0	0	0	0	0	0
2026	5	0	0	0	0	0	0
2027	6	0	0	0	0	1	0
2028	5	0	0	0	0	0	0
2029	2	0	0	0	0	0	0
2030	5	0	0	0	0	0	0
2031	2	0	0	0	0	0	0
2032	2	0	0	0	1	1	1
2033	4	0	0	0	1	0	1
2034	3	0	0	0	0	0	1
2035	7	0	0	0	1	0	1
2036	4	0	0	0	0	0	1
2037	3	0	0	0	1	1	1
2038	2	0	0	0	1	1	1
2039	2	0	0	0	1	1	1
2040	2	0	0	0	0	0	1
2041	3	0	0	0	1	0	1
2042	18	0	0	0	1	1	1
2043	2	0	0	0	0	0	1
2044	4	0	0	0	1	1	1
2045	6	0	0	0	1	1	1
2046	4	0	0	0	1	1	1
2047	5	0	0	0	0	0	1
2048	2	0	0	0	1	0	1
2049	2	0	0	0	1	0	1

2050	4	0	0	0	0	1	1
2051	2	0	0	0	1	1	0
2052	7	0	0	0	1	0	1
2053	2	0	0	0	1	0	0
2054	2	0	0	0	1	1	1
2055	5	0	0	0	0	0	0
2056	8	0	0	0	1	1	1
2057	10	0	0	0	1	0	1
2058	5	0	0	0	1	0	1
2059	4	0	0	0	0	0	1
2060	5	0	0	0	1	1	1
2061	4	0	0	0	0	0	1
2062	2	0	0	0	1	1	1
2063	3	0	0	0	0	0	0
2064	3	0	0	0	0	0	0
2065	2	0	0	0	0	0	0
2066	3	0	0	0	1	0	1
2067	4	0	0	0	1	0	0
2068	2	0	0	0	0	0	0
2069	4	0	0	0	1	1	1
2070	6	0	0	0	1	0	1
2071	5	0	0	0	0	0	0
2072	2	0	0	0	1	1	0
2073	2	0	0	0	0	0	0
2074	2	0	0	0	0	0	0
2075	5	0	0	0	0	0	0
2076	2	0	0	0	0	0	0
2077	2	0	0	0	0	0	0
2078	4	0	0	0	0	0	0
2079	8	0	0	0	1	1	0
2080	3	0	0	0	1	0	0
2081	2	0	0	0	1	1	1
2082	8	0	0	0	0	1	0
2083	5	0	0	0	0	0	0
2084	2	0	0	0	0	0	0
2085	4	0	0	0	0	0	0
2086	6	0	0	0	0	0	0
2087	2	0	0	0	0	0	0
2088	2	0	0	0	0	0	0
2089	2	0	0	0	0	0	0
2090	2	0	0	0	0	0	0

2091	3	0	0	0	0	1	0
2092	4	0	0	0	1	0	0
2093	4	0	0	0	0	1	0
2094	4	0	0	0	0	0	0
2095	4	0	0	0	1	1	0
2096	6	0	0	0	1	1	0
2097	4	0	0	0	0	0	0
2098	6	0	0	0	0	1	0
2099	16	0	0	0	0	0	0
2100	2	0	0	0	1	1	0
2101	2	0	0	0	0	0	0
2102	4	0	0	0	0	0	0
2103	7	0	0	0	1	1	0
2104	5	0	0	0	0	1	0
2105	9	0	0	1	0	0	0
2106	4	0	0	0	0	0	1
2107	2	0	0	0	1	0	0
2108	2	0	0	0	1	1	0
2109	2	0	0	0	0	0	0
2110	2	0	0	0	1	0	1
2111	4	0	0	0	0	1	1
2112	2	0	0	0	1	0	1
2113	3	0	0	0	0	0	1
2114	5	0	0	0	1	0	1
2115	3	0	0	0	0	0	1
2116	5	0	0	0	1	1	1
2117	2	0	0	0	1	1	1
2118	4	0	0	0	1	1	1
2119	2	0	0	0	0	0	1
2120	3	0	0	0	1	1	1
2121	11	0	0	0	0	1	1
2122	5	0	0	0	1	1	1
2123	4	0	0	0	1	0	1
2124	4	0	0	0	1	1	1
2125	2	0	0	0	1	1	1
2126	3	0	0	0	1	0	1
2127	2	0	0	0	1	1	1
2128	5	0	0	0	0	0	1
2129	2	0	0	0	1	0	1
2130	2	0	0	0	0	1	1
2131	3	0	0	0	1	1	1
2132	2	0	0	0	1	1	1

2133	2	0	0	0	1	1	1
2134	5	0	0	0	0	1	1
2135	3	0	0	0	0	0	0
2136	5	0	0	0	0	0	0
2137	2	0	0	0	0	1	0
2138	9	0	0	0	1	1	0
2139	2	0	0	0	0	0	0
2140	6	0	0	0	1	0	0
2141	2	0	0	0	0	0	0
2142	3	0	0	0	0	0	0
2143	10	0	0	0	1	1	0
2144	3	0	0	0	0	0	0
2145	6	0	0	0	1	0	0
2146	2	0	0	0	0	0	0
2147	2	0	0	0	0	0	0
2148	11	0	0	0	0	0	0
2149	4	0	0	0	1	0	0
2150	7	0	0	0	0	0	0
2151	2	0	0	0	0	1	0
2152	2	0	0	0	0	0	0
2153	8	0	0	0	0	0	0
2154	2	0	0	0	0	0	0
2155	2	0	0	0	0	0	0
2156	4	0	0	0	0	0	0
2157	6	0	0	0	0	0	0
2158	3	0	0	0	0	0	0
2159	2	0	0	0	0	0	0
2160	2	0	0	0	0	1	1
2161	9	0	0	0	0	0	0
2162	2	0	0	0	0	0	0
2163	2	0	0	0	0	0	0
2164	5	0	0	0	0	0	0
2165	4	0	0	0	0	0	0
2166	2	0	0	0	0	0	0
2167	4	0	0	0	0	1	0
2168	4	0	0	0	0	0	0
2169	6	0	0	0	0	0	0
2170	3	0	0	1	0	0	0
2171	6	0	0	0	0	0	0
2172	3	0	0	0	0	0	0
2173	2	0	0	0	0	0	0
2174	4	0	0	0	0	0	0

2175	2	0	0	0	0	0	0
2176	3	0	0	0	0	0	0
2177	4	0	0	0	0	0	0
2178	2	0	0	0	0	0	0
2179	3	0	0	0	0	0	0
2180	2	0	0	0	0	0	0
2181	2	0	0	0	0	0	0
2182	3	0	0	0	0	0	0
2183	2	0	0	0	0	0	0
2184	3	0	0	0	0	0	0
2185	2	0	0	0	0	0	0
2186	4	0	0	0	0	0	0
2187	3	0	0	0	0	0	0
2188	2	0	0	0	1	0	0
2189	7	0	0	0	0	0	0
2190	2	0	0	0	1	0	1
2191	4	0	0	0	0	0	0
2192	4	0	0	0	0	0	0
2193	2	0	0	0	0	0	0
2194	2	0	0	0	0	0	0
2195	2	0	0	0	0	0	0
2196	3	0	0	0	0	0	0
2197	3	0	0	0	1	1	0
2198	4	0	0	0	0	0	0
2199	4	0	0	0	1	0	0
2200	4	0	0	0	0	0	0
2201	6	0	0	0	0	0	0
2202	4	0	0	0	0	0	0
2203	3	0	0	0	0	0	0
2204	4	0	0	0	1	1	0
2205	5	0	0	0	0	0	0
2206	5	0	0	0	0	0	0
2207	3	0	0	0	0	0	0
2208	3	0	0	0	0	0	0
2209	4	0	0	0	0	1	1
2210	3	0	0	0	0	0	0
2211	4	0	0	0	0	0	0
2212	4	0	0	0	0	0	0
2213	3	0	0	0	1	0	0
2214	3	0	0	0	0	0	0

2215	2	0	0	0	0	0	0
2216	8	0	0	0	1	0	0
2217	3	0	0	0	0	0	0
2218	5	0	0	0	0	1	1
2219	5	0	0	0	0	0	0
2220	5	0	0	0	0	0	0
2221	6	0	0	0	0	0	0
2222	2	0	0	0	0	0	0
2223	4	0	0	0	0	0	0
2224	2	0	0	0	0	0	0
2225	2	0	0	0	0	0	0
2226	2	0	0	0	0	0	0
2227	2	0	0	0	0	1	0
2228	5	0	0	0	0	1	1
2229	7	0	0	0	1	0	0
2230	5	0	0	0	0	0	0
2231	2	0	0	0	1	0	0
2232	3	0	0	0	0	0	0
2233	5	0	0	0	0	0	0
2234	3	0	0	0	1	1	0
2235	6	0	0	0	0	0	0
2236	2	0	0	0	0	0	0
2237	4	0	0	0	0	0	0
2238	3	0	0	0	0	0	0
2239	2	0	0	0	1	0	0
2240	4	0	0	0	1	0	1
2241	4	0	0	0	0	0	0
2242	3	0	0	0	1	0	0
2243	2	0	0	0	0	1	0
2244	12	0	0	0	0	0	0
2245	2	0	0	0	1	0	0
2246	2	0	0	0	1	0	0
2247	4	0	0	0	1	1	1
2248	4	0	0	0	0	0	0
2249	6	0	0	0	1	1	0
2250	2	0	0	0	0	0	0
2251	4	0	0	0	0	0	0
2252	2	0	0	0	1	0	0
2253	4	0	0	0	0	0	0
2254	3	0	0	0	0	0	0
2255	2	0	0	0	0	0	0
2256	2	0	0	0	1	0	0
2257	2	0	0	0	0	0	0

2258	2	0	0	0	0	0	0
2259	4	0	0	0	0	0	0
2260	4	0	0	0	0	0	0
2261	4	0	0	0	0	0	0
2262	2	0	0	1	0	0	0
2263	4	0	0	0	1	0	1
2264	4	0	0	0	0	0	0
2265	7	0	0	0	0	0	0
2266	3	0	0	1	1	0	0
2267	5	0	0	0	0	0	0
2268	3	0	0	0	1	0	0
2269	5	0	0	0	1	1	0
2270	6	0	0	0	1	1	1
2271	4	0	0	0	0	0	0
2272	4	0	0	0	1	1	1
2273	2	0	0	0	0	0	0
2274	3	0	0	0	0	0	0
2275	4	0	0	0	0	1	0
2276	4	0	0	0	0	0	0
2277	4	0	0	0	0	0	0
2278	4	0	0	0	1	0	0
2279	6	0	0	0	0	0	0
2280	5	0	0	0	0	0	0
2281	3	0	0	0	0	0	0
2282	5	0	0	0	1	1	0
2283	3	0	0	0	0	0	0
2284	5	0	0	0	0	1	1
2285	7	0	0	0	0	0	0
2286	3	0	0	0	0	0	0
2287	4	0	0	0	0	0	0
2288	4	0	0	0	0	0	0
2289	4	0	0	0	0	0	0
2290	4	0	0	0	0	0	1
2291	5	0	0	0	1	1	0
2292	4	0	0	0	0	0	0
2293	2	0	0	0	0	0	0
2294	4	0	0	0	0	0	0
2295	3	0	0	0	0	0	0
2296	4	0	0	0	0	1	0
2297	3	0	0	0	0	0	0
2298	2	0	0	0	0	1	0
2299	4	0	0	0	1	0	0
2300	4	0	0	0	0	0	0

2301	3	0	0	0	1	0	0
2302	4	0	0	0	0	0	0
2303	2	0	0	0	0	0	0
2304	15	0	0	0	0	0	0
2305	4	0	0	0	1	0	0
2306	4	0	0	0	1	0	0
2307	4	0	0	0	0	0	0
2308	4	0	0	0	0	0	0
2309	3	0	0	0	0	1	0
2310	2	0	0	0	0	0	0
2311	4	0	0	0	1	0	0
2312	3	0	0	0	0	1	0
2313	3	0	0	0	1	0	0
2314	2	0	0	0	0	0	0
2315	3	0	0	0	0	0	0
2316	4	0	0	0	0	0	0
2317	4	0	0	0	1	0	1
2318	3	0	0	0	1	0	0
2319	3	0	0	0	0	1	1
2320	2	0	0	0	1	0	0
2321	6	0	0	0	0	0	0
2322	2	0	0	0	1	1	0
2323	10	0	0	0	1	0	0
2324	4	0	0	0	0	0	0
2325	6	0	0	0	1	0	0
2326	4	0	0	0	0	0	0
2327	3	0	0	0	0	0	0
2328	3	0	0	0	1	1	0
2329	3	0	0	0	0	0	0
2330	4	0	0	0	1	0	0
2331	4	0	0	0	0	0	0
2332	2	0	0	0	0	0	0
2333	4	0	0	0	1	0	0
2334	4	0	0	0	0	0	0
2335	4	0	0	0	1	0	1
2336	4	0	0	0	1	1	1
2337	5	0	0	0	0	0	0
2338	2	0	0	0	0	0	0
2339	2	0	0	0	1	0	0
2340	3	0	0	0	0	0	0
2341	8	0	0	0	1	0	0
2342	4	0	0	0	0	1	1

2343	3	0	0	1	0	0	0
2344	6	0	0	0	1	0	0
2345	2	0	0	0	0	0	0
2346	2	0	0	0	0	0	0
2347	3	0	0	0	0	0	0
2348	2	0	0	0	1	0	0
2349	5	0	0	0	1	0	0
2350	5	0	0	0	0	0	0
2351	5	0	0	0	0	0	0
2352	4	0	0	0	1	0	1
2353	3	0	0	0	0	0	0
2354	4	0	0	0	0	0	0
2355	3	0	0	0	0	0	0
2356	5	0	0	0	0	0	0
2357	3	0	0	0	0	0	0
2358	2	0	0	0	1	0	0
2359	10	0	0	0	0	0	0
2360	4	0	0	0	0	0	0
2361	4	0	0	0	1	1	1
2362	4	0	0	0	0	0	0
2363	6	0	0	0	1	0	0
2364	10	0	0	0	1	0	0
2365	4	0	0	0	0	1	0
2366	4	0	0	0	0	1	0
2367	2	0	0	0	1	0	0
2368	4	0	0	0	0	0	0
2369	2	0	0	0	1	0	0
2370	4	0	0	0	0	0	1
2371	6	0	0	0	0	1	0
2372	2	0	0	0	0	0	0
2373	9	0	0	0	0	0	0
2374	4	0	0	0	1	1	0
2375	6	0	0	0	0	0	0
2376	4	0	0	0	0	1	0
2377	4	0	0	0	0	0	0
2378	4	0	0	0	1	1	0
2379	4	0	0	0	1	1	0
2380	3	0	0	0	0	0	0
2381	3	0	0	0	0	0	0
2382	4	0	0	0	1	1	0
2383	4	0	0	0	0	0	0
2384	3	0	0	0	0	0	0

2385	4	0	0	0	0	0	0
2386	2	0	0	0	0	0	0
2387	8	0	0	0	0	0	0
2388	2	0	0	0	0	0	0
2389	2	0	0	0	0	0	0
2390	7	0	0	0	0	0	0
2391	9	0	0	0	0	0	0
2392	3	0	0	0	0	0	0
2393	4	0	0	0	1	1	1
2394	4	0	0	0	0	0	0
2395	2	0	0	0	0	0	0
2396	2	0	0	0	0	0	0
2397	5	0	0	0	0	0	0
2398	4	0	0	0	0	0	0
2399	3	0	0	0	1	0	0
2400	5	0	0	0	1	1	1
2401	3	0	0	0	0	0	0
2402	4	0	0	0	0	0	0
2403	4	0	0	0	0	0	1
2404	14	0	0	0	0	0	0
2405	3	0	0	0	1	0	0
2406	5	0	0	0	0	1	0
2407	2	0	0	0	0	0	0
2408	3	0	0	0	0	0	0
2409	4	0	0	0	0	0	0
2410	2	0	0	0	1	1	0
2411	4	0	0	0	0	0	0
2412	3	0	0	0	1	0	0
2413	2	0	0	0	0	0	0
2414	4	0	0	0	0	0	0
2415	2	0	0	0	0	0	0
2416	5	0	0	0	0	0	0
2417	4	0	0	0	0	0	0
2418	4	0	0	0	0	0	1
2419	3	0	0	0	1	0	0
2420	6	0	0	0	1	0	0
2421	7	0	0	0	1	1	0
2422	2	0	0	0	0	0	0
2423	12	0	0	0	0	0	0
2424	2	0	0	0	0	0	0
2425	4	0	0	0	0	0	0
2426	4	0	0	0	1	1	0

2427	2	0	0	0	0	0	0
2428	4	0	0	0	0	0	0
2429	3	0	0	0	0	1	0
2430	2	0	0	0	1	0	0
2431	4	0	0	0	0	0	0
2432	2	0	0	0	1	0	0
2433	2	0	0	0	0	0	0
2434	13	0	0	0	0	0	0
2435	2	0	0	0	0	0	0
2436	2	0	0	0	1	1	0
2437	3	0	0	0	0	0	0
2438	2	0	0	0	1	0	0
2439	4	0	0	0	1	0	0
2440	2	0	0	0	0	0	0
2441	2	0	0	0	0	0	0
2442	3	0	0	0	0	0	0
2443	5	0	0	0	1	0	0
2444	2	0	0	0	0	0	0
2445	2	0	0	0	0	0	0
2446	2	0	0	0	0	1	0
2447	2	0	0	0	0	0	0
2448	5	0	0	0	1	0	0
2449	3	0	0	0	0	0	0
2450	12	0	0	0	0	0	0
2451	5	0	0	0	0	0	0
2452	4	0	0	0	1	0	0
2453	5	0	0	0	1	0	1
2454	3	0	0	0	0	0	0
2455	6	0	0	0	0	1	0
2456	2	0	0	0	0	0	0
2457	3	0	0	0	0	0	0
2458	2	0	0	0	0	0	0
2459	4	0	0	0	0	0	0
2460	2	0	0	0	0	0	0
2461	3	0	0	0	0	0	0
2462	2	0	0	0	1	0	0
2463	4	0	0	0	0	0	0
2464	4	0	0	0	1	1	0
2465	5	0	0	0	0	0	0
2466	6	0	0	0	0	0	0
2467	4	0	0	0	0	0	0
2468	5	0	0	0	1	1	0

2469	4	0	0	0	0	0	0
2470	5	0	0	0	1	0	0
2471	8	0	0	0	1	0	0
2472	5	0	0	0	0	0	0
2473	3	0	0	0	1	0	0
2474	3	0	0	0	1	0	0
2475	2	0	0	0	1	0	0
2476	5	0	0	0	0	0	0
2477	2	0	0	0	0	0	0
2478	3	0	0	0	0	0	0
2479	4	0	0	0	0	0	0
2480	5	0	0	0	0	0	0
2481	3	0	0	0	1	0	0
2482	4	0	0	0	1	1	0
2483	2	0	0	0	0	0	0
2484	4	0	0	0	0	0	0
2485	4	0	0	0	1	1	0
2486	4	0	0	0	0	0	0
2487	2	0	0	0	0	0	0
2488	2	0	0	0	0	0	0
2489	2	0	0	0	0	0	0
2490	4	0	0	0	0	0	0
2491	2	0	0	0	0	1	0
2492	2	0	0	0	0	0	0
2493	4	0	0	0	1	1	0
2494	6	0	0	0	0	1	1
2495	3	0	0	0	0	0	0
2496	4	0	0	0	0	0	0
2497	7	0	0	0	1	0	0
2498	4	0	0	0	0	0	0
2499	3	0	0	0	0	0	0
2500	4	0	0	0	0	0	0
2501	4	0	0	0	0	0	0
2502	3	0	0	0	1	0	0
2503	4	0	0	0	1	0	0
2504	4	0	0	0	0	1	0
2505	5	0	0	0	1	0	0
2506	4	0	0	0	0	0	0
2507	3	0	0	0	1	0	0
2508	4	0	0	0	0	0	0
2509	5	0	0	0	0	0	0
2510	11	0	0	0	1	0	0

2511	4	0	0	0	0	0	0
2512	2	0	0	0	0	0	0
2513	4	0	0	0	0	0	0
2514	6	0	0	0	0	0	0
2515	4	0	0	0	0	0	0
2516	2	0	0	0	0	0	0
2517	4	0	0	0	0	0	0
2518	2	0	0	0	0	0	0
2519	2	0	0	0	0	0	0
2520	4	0	0	0	1	0	0
2521	4	0	0	0	0	0	0
2522	3	0	0	0	0	0	0
2523	3	0	0	0	1	0	0
2524	2	0	0	0	0	0	0
2525	6	0	0	0	0	0	0
2526	3	0	0	0	0	0	0
2527	4	0	0	0	0	0	0
2528	2	0	0	0	0	0	0
2529	3	0	0	0	1	0	0
2530	7	0	0	0	1	0	0
2531	3	0	0	0	0	0	0
2532	4	0	0	0	1	0	0
2533	4	0	0	0	0	0	0
2534	4	0	0	0	0	1	0
2535	12	0	0	0	0	0	0
2536	4	0	0	0	1	0	0
2537	4	0	0	0	0	0	0
2538	3	0	0	0	0	0	0
2539	3	0	0	0	0	0	0
2540	4	0	0	0	1	1	1
2541	2	0	0	0	1	1	0
2542	2	0	0	0	0	0	0
2543	2	0	0	0	1	0	0
2544	6	0	0	0	1	0	0
2545	4	0	0	0	0	0	0
2546	5	0	0	0	0	0	0
2547	3	0	0	0	1	0	0
2548	8	0	0	0	1	0	0
2549	2	0	0	0	0	0	0
2550	8	0	0	0	1	0	0
2551	3	0	0	0	0	0	0

2552	3	0	0	0	0	0	0
2553	4	0	0	0	0	0	0
2554	4	0	0	0	0	1	0
2555	3	0	0	0	1	0	0
2556	4	0	0	0	0	0	0
2557	4	0	0	0	0	1	0
2558	4	0	0	0	0	0	0
2559	2	0	0	0	1	0	0
2560	5	0	0	0	0	1	0
2561	7	0	0	0	0	0	0
2562	4	0	0	0	1	1	1
2563	5	0	0	0	1	0	0
2564	4	0	0	0	0	0	0
2565	2	0	0	0	0	0	0
2566	6	0	0	0	1	0	0
2567	2	0	0	0	0	0	0
2568	3	0	0	0	1	1	0
2569	8	0	0	0	0	1	0
2570	2	0	0	0	0	1	0
2571	2	0	0	0	0	0	0
2572	4	0	0	0	0	1	1
2573	5	0	0	0	0	0	0
2574	4	0	0	0	1	0	0
2575	2	0	0	0	0	0	0
2576	4	0	0	0	0	0	0
2577	6	0	0	0	0	0	0
2578	2	0	0	0	0	0	0
2579	2	0	0	0	0	0	0
2580	4	0	0	0	0	0	0
2581	4	0	0	0	0	0	0
2582	4	0	0	0	0	0	0
2583	5	0	0	0	1	0	0
2584	4	0	0	0	0	0	0
2585	4	0	0	0	0	0	0
2586	6	0	0	0	1	0	0
2587	3	0	0	0	1	0	0
2588	4	0	0	0	0	0	0
2589	2	0	0	0	0	0	0
2590	2	0	0	0	1	0	0
2591	3	0	0	0	0	0	0
2592	2	0	0	0	0	0	0

2593	5	0	0	0	0	0	1
2594	3	0	0	0	0	0	0
2595	2	0	0	0	1	0	0
2596	3	0	0	0	0	0	0
2597	4	0	0	0	0	0	0
2598	7	0	0	0	1	0	0
2599	4	0	0	0	0	0	0
2600	2	0	0	0	0	0	0
2601	4	0	0	0	0	0	0
2602	4	0	0	0	0	0	0
2603	6	0	0	0	0	0	0
2604	5	0	0	0	0	0	0
2605	3	0	0	0	1	0	0
2606	4	0	0	0	0	1	1
2607	7	0	0	0	0	0	0
2608	3	0	0	0	0	0	0
2609	4	0	0	0	0	0	0
2610	4	0	0	0	1	1	0
2611	5	0	0	0	1	0	0
2612	4	0	0	0	1	0	0
2613	4	0	0	0	0	0	0
2614	2	0	0	0	0	0	0
2615	2	0	0	0	1	1	0
2616	4	0	0	0	0	0	0
2617	2	0	0	0	0	0	0
2618	4	0	0	0	0	1	0
2619	2	0	0	0	0	0	0
2620	5	0	0	0	1	0	0
2621	2	0	0	0	0	0	0
2622	2	0	0	0	0	0	0
2623	2	0	0	0	0	0	0
2624	2	0	0	0	0	1	0
2625	5	0	0	0	0	0	0
2626	3	0	0	0	1	0	0
2627	4	0	0	0	0	0	0
2628	4	0	0	0	0	0	0
2629	5	0	0	0	0	0	1
2630	6	0	0	0	0	0	0
2631	4	0	0	0	0	0	0
2632	2	0	0	0	1	1	0
2633	5	0	0	0	0	0	0

2634	3	0	0	0	0	0	0
2635	3	0	0	0	0	0	0
2636	2	0	0	0	1	0	0
2637	5	0	0	0	0	0	0
2638	4	0	0	0	0	1	0
2639	2	0	0	0	0	0	0
2640	4	0	0	0	0	0	0
2641	3	0	0	0	0	0	0
2642	4	0	0	0	1	1	0
2643	4	0	0	0	1	1	0
2644	3	0	0	0	0	0	0
2645	2	0	0	0	0	0	0
2646	6	0	0	0	0	0	0
2647	4	0	0	0	1	0	0
2648	2	0	0	1	0	0	0
2649	9	0	0	0	1	1	0
2650	4	0	0	0	0	0	0
2651	4	0	0	0	0	0	0
2652	4	0	0	0	0	0	0
2653	3	0	0	0	0	0	0
2654	2	0	0	0	1	1	0
2655	4	0	0	0	0	0	0
2656	11	0	0	0	0	0	0
2657	2	0	0	0	0	0	0
2658	3	0	0	0	0	0	0
2659	2	0	0	0	0	0	0
2660	4	0	0	0	1	1	0
2661	11	0	0	0	1	0	0
2662	14	0	0	0	0	0	0
2663	4	0	0	0	1	0	0
2664	2	0	0	0	0	0	0
2665	3	0	0	0	0	0	0
2666	2	0	0	1	0	0	0
2667	3	0	0	0	1	0	0
2668	7	0	0	0	1	1	0
2669	5	0	0	0	0	0	0
2670	5	0	0	0	0	0	0
2671	4	0	0	0	1	0	0
2672	4	0	0	0	0	0	0
2673	3	0	0	0	0	0	0
2674	3	0	0	0	1	0	0

2675	4	0	0	0	0	0	0
2676	7	0	0	0	0	0	0
2677	2	0	0	0	0	0	0
2678	14	0	0	0	1	0	0
2679	2	0	0	0	0	0	0
2680	5	0	0	0	0	0	0
2681	2	0	0	0	1	0	0
2682	4	0	0	0	1	0	1
2683	13	0	0	0	0	0	0
2684	4	0	0	0	1	0	0
2685	4	0	0	0	0	0	0
2686	3	0	0	0	1	0	0
2687	4	0	0	0	0	0	1
2688	3	0	0	0	0	0	0
2689	3	0	0	0	1	0	0
2690	4	0	0	0	1	1	0
2691	3	0	0	0	0	0	0
2692	2	0	0	0	0	0	0
2693	6	0	0	0	1	0	0
2694	4	0	0	0	0	0	0
2695	2	0	0	0	0	0	0
2696	2	0	0	0	0	0	0
2697	3	0	0	0	1	0	0
2698	4	0	0	0	0	0	0
2699	4	0	0	0	0	0	0
2700	3	0	0	0	1	1	1
2701	2	0	0	0	0	1	0
2702	3	0	0	0	0	0	0
2703	4	0	0	0	0	0	0
2704	4	0	0	0	1	0	0
2705	5	0	0	0	1	0	0
2706	2	0	0	0	0	0	0
2707	11	0	0	0	0	0	0
2708	2	0	0	0	0	0	0
2709	4	0	0	0	0	0	0
2710	3	0	0	0	0	0	0
2711	5	0	0	0	0	1	0
2712	4	0	0	0	0	0	0
2713	2	0	0	0	0	0	0
2714	5	0	0	0	0	0	0
2715	4	0	0	0	0	0	0

2716	2	0	0	0	0	0	0
2717	4	0	0	0	0	0	0
2718	4	0	0	0	1	0	0
2719	4	0	0	0	0	0	0
2720	2	0	0	0	0	0	0
2721	4	0	0	0	0	0	0
2722	19	0	0	0	1	0	0
2723	3	0	0	0	0	0	0
2724	2	0	0	0	1	1	0
2725	4	0	0	0	0	0	0
2726	2	0	0	0	0	0	0
2727	4	0	0	0	0	0	0
2728	3	0	0	0	0	0	0
2729	3	0	0	0	1	0	0
2730	6	0	0	0	1	0	0
2731	2	0	0	0	1	0	0
2732	4	0	0	0	0	0	0
2733	4	0	0	0	1	0	0
2734	5	0	0	0	0	0	0
2735	4	0	0	0	0	0	0
2736	2	0	0	0	0	0	0
2737	4	0	0	0	1	1	0
2738	2	0	0	0	0	1	0
2739	3	0	0	0	1	0	0
2740	3	0	0	0	0	0	0
2741	6	0	0	0	0	0	0
2742	3	0	0	0	0	0	0
2743	3	0	0	0	0	0	0
2744	3	0	0	0	1	0	0
2745	2	0	0	0	0	0	0
2746	4	0	0	0	0	0	0
2747	13	0	0	0	1	0	0
2748	6	0	0	0	0	0	0
2749	7	0	0	0	0	1	0
2750	6	0	0	0	0	0	0
2751	10	0	0	0	0	0	0
2752	2	0	0	0	0	0	0
2753	2	0	0	0	1	0	0
2754	2	0	0	0	0	0	0
2755	5	0	0	0	0	0	0
2756	2	0	0	0	0	0	0

2757	2	0	0	0	0	0	0
2758	4	0	0	0	0	0	0
2759	2	0	0	0	0	1	0
2760	5	0	0	0	0	0	0
2761	4	0	0	0	1	1	0
2762	2	0	0	0	0	1	0
2763	2	0	0	0	0	0	0
2764	5	0	0	0	0	0	0
2765	3	0	0	0	0	1	0
2766	2	0	0	0	0	0	0
2767	5	0	0	0	0	0	0
2768	2	0	0	0	0	0	0
2769	2	0	0	0	1	0	0
2770	5	0	0	0	0	0	0
2771	5	0	0	0	0	0	0
2772	2	0	0	0	0	0	0
2773	4	0	0	0	0	0	0
2774	4	0	0	0	0	0	0
2775	5	0	0	0	0	0	0
2776	4	0	0	0	0	0	0
2777	3	0	0	0	0	0	0
2778	7	0	0	0	0	0	0
2779	4	0	0	0	0	0	0
2780	4	0	0	0	0	0	0
2781	2	0	0	0	1	0	0
2782	2	0	0	0	0	0	0
2783	4	0	0	0	0	0	0
2784	2	0	0	0	0	0	0
2785	3	0	0	0	1	1	0
2786	3	0	0	0	1	1	1
2787	2	0	0	0	0	0	0
2788	3	0	0	0	1	0	0
2789	4	0	0	0	0	0	0
2790	3	0	0	0	1	0	0
2791	6	0	0	0	0	0	0
2792	3	0	0	0	0	0	0
2793	4	0	0	0	0	0	0
2794	2	0	0	0	0	0	0
2795	2	0	0	0	1	0	0
2796	2	0	0	0	0	0	0
2797	2	0	0	0	1	0	0

2798	4	0	0	0	0	0	0
2799	8	0	0	0	1	0	0
2800	4	0	0	0	0	0	0
2801	4	0	0	0	0	0	0
2802	5	0	0	0	0	0	0
2803	2	0	0	0	1	0	0
2804	12	0	0	0	0	0	0
2805	4	0	0	0	0	0	0
2806	3	0	0	0	1	1	0
2807	5	0	0	0	0	0	0
2808	4	0	0	0	0	0	0
2809	5	0	0	0	0	0	0
2810	4	0	0	0	0	0	0
2811	2	0	0	0	0	0	0
2812	3	0	0	0	0	0	0
2813	3	0	0	0	0	1	0
2814	2	0	0	0	1	0	0
2815	2	0	0	0	0	0	0
2816	4	0	0	0	1	0	0
2817	4	0	0	0	0	0	0
2818	2	0	0	0	0	0	0
2819	4	0	0	0	1	1	1
2820	3	0	0	0	0	0	0
2821	5	0	0	0	0	1	0
2822	12	0	0	0	0	0	0
2823	4	0	0	0	0	0	0
2824	4	0	0	0	1	1	1
2825	2	0	0	0	0	0	0
2826	8	0	0	0	1	0	0
2827	6	0	0	0	0	0	0
2828	4	0	0	0	0	0	0
2829	6	0	0	0	0	0	0
2830	4	0	0	0	1	0	0
2831	4	0	0	0	0	0	0
2832	6	0	0	0	0	0	0
2833	4	0	0	0	1	1	1
2834	3	0	0	0	0	0	0
2835	8	0	0	0	0	0	0
2836	3	0	0	0	1	0	0
2837	2	0	0	0	1	1	0
2838	4	0	0	0	0	0	0

2839	4	0	0	0	0	1	0
2840	4	0	0	0	0	0	0
2841	4	0	0	0	0	0	0
2842	4	0	0	0	1	0	0
2843	2	0	0	0	0	0	0
2844	4	0	0	0	0	0	0
2845	4	0	0	0	1	1	0
2846	3	0	0	0	0	0	0
2847	2	0	0	0	0	1	0
2848	5	0	0	0	0	0	0
2849	2	0	0	0	0	0	0
2850	4	0	0	0	1	1	0
2851	2	0	0	0	0	0	0
2852	3	0	0	0	0	0	0
2853	2	0	0	0	1	0	0
2854	3	0	0	0	0	0	0
2855	5	0	0	0	1	0	0
2856	3	0	0	0	0	0	0
2857	4	0	0	0	0	0	0
2858	4	0	0	0	0	0	0
2859	6	0	0	0	0	0	0
2860	8	0	0	0	0	0	0
2861	2	0	0	0	0	0	0
2862	3	0	0	0	0	1	0
2863	5	0	0	0	0	0	0
2864	2	0	0	0	0	0	0
2865	2	0	0	0	1	1	0
2866	3	0	0	0	0	0	0
2867	3	0	0	0	0	0	0
2868	4	0	0	0	0	0	0
2869	5	0	0	0	0	0	0
2870	5	0	0	0	1	0	0
2871	3	0	0	0	1	0	1
2872	3	0	0	0	0	0	0
2873	2	0	0	0	0	0	0
2874	10	0	0	0	1	0	0
2875	2	0	0	0	1	0	0
2876	4	0	0	0	0	0	0
2877	2	0	0	1	1	1	0
2878	8	0	0	0	0	0	0
2879	2	0	0	0	1	0	0
2880	3	0	0	0	0	0	0

2881	6	0	0	0	0	0	0
2882	2	0	0	0	0	1	0
2883	4	0	0	0	0	0	0
2884	3	0	0	0	0	0	0
2885	2	0	0	0	0	0	0
2886	3	0	0	0	0	0	0
2887	3	0	0	0	0	1	0
2888	4	0	0	0	1	0	0
2889	2	0	0	0	0	0	0
2890	3	0	0	0	0	0	0
2891	6	0	0	0	1	1	0
2892	2	0	0	0	0	0	0
2893	2	0	0	0	0	0	0
2894	2	0	0	0	0	0	0
2895	4	0	0	0	1	1	0
2896	2	0	0	0	0	0	0
2897	3	0	0	0	1	1	0
2898	2	0	0	0	1	1	0
2899	6	0	0	0	0	0	0
2900	3	0	0	0	0	0	0
2901	7	0	0	0	1	0	0
2902	3	0	0	0	0	0	0
2903	2	0	0	0	0	0	0
2904	2	0	0	0	0	0	0
2905	3	0	0	0	1	1	0
2906	3	0	0	0	1	0	0
2907	5	0	0	0	1	1	0
2908	4	0	0	0	0	1	0
2909	2	0	0	0	0	0	0
2910	3	0	0	0	1	0	0
2911	4	0	0	0	0	1	0
2912	3	0	0	0	0	0	0
2913	4	0	0	0	0	0	0
2914	6	0	0	0	0	0	0
2915	6	0	0	0	0	0	0
2916	5	0	0	0	0	0	0
2917	4	0	0	0	0	0	0
2918	2	0	0	0	1	0	0
2919	5	0	0	0	0	0	0
2920	4	0	0	0	1	1	0
2921	2	0	0	0	0	0	0

2922	6	0	0	0	0	0	0
2923	4	0	0	0	0	0	0
2924	4	0	0	0	1	0	0
2925	3	0	0	0	0	0	0
2926	3	0	0	0	0	0	0
2927	10	0	0	0	1	0	0
2928	3	0	0	0	1	1	0
2929	4	0	0	0	0	0	0
2930	2	0	0	0	0	0	0
2931	6	0	0	0	0	0	0
2932	3	0	0	0	1	1	0
2933	4	0	0	0	0	0	0
2934	2	0	0	0	0	0	0
2935	4	0	0	0	0	0	0
2936	3	0	0	0	0	1	0
2937	2	0	0	0	0	0	0
2938	5	0	0	0	0	0	0
2939	3	0	0	0	0	0	0
2940	7	0	0	0	0	0	0
2941	4	0	0	0	0	0	0
2942	2	0	0	0	0	0	0
2943	4	0	0	0	0	0	0
2944	4	0	0	0	1	1	0
2945	2	0	0	0	0	0	0
2946	3	0	0	0	1	0	1
2947	2	0	0	0	0	0	0
2948	4	0	0	0	0	0	0
2949	6	0	0	0	0	0	0
2950	3	0	0	0	0	0	0
2951	4	0	0	0	0	0	0
2952	2	0	0	0	0	0	0
2953	4	0	0	0	0	1	0
2954	4	0	0	0	0	0	0
2955	17	0	0	0	0	0	0
2956	5	0	0	0	0	0	0
2957	5	0	0	0	0	0	0
2958	2	0	0	0	0	0	0
2959	4	0	0	0	1	0	1
2960	2	0	0	0	0	0	0
2961	2	0	0	0	0	0	0
2962	5	0	0	0	0	0	0

2963	6	0	0	0	0	0	0
2964	2	0	0	0	0	0	0
2965	3	0	0	0	0	0	0
2966	4	0	0	0	0	0	0
2967	6	0	0	0	0	0	0
2968	5	0	0	0	1	0	0
2969	4	0	0	0	1	0	0
2970	2	0	0	0	1	0	0
2971	6	0	0	0	0	0	0
2972	3	0	0	0	0	0	0
2973	3	0	0	0	0	0	0
2974	8	0	0	0	0	0	0
2975	6	0	0	0	0	0	0
2976	6	0	0	0	0	0	0
2977	4	0	0	0	0	0	0
2978	2	0	0	0	0	1	0
2979	2	0	0	0	0	0	0
2980	6	0	0	0	0	0	0
2981	2	0	0	0	0	0	0
2982	7	0	0	0	0	0	0
2983	3	0	0	0	0	0	0
2984	4	0	0	0	0	0	0
2985	5	0	0	0	0	0	0
2986	5	0	0	0	0	0	0
2987	7	0	0	0	0	0	0
2988	4	0	0	0	0	0	0
2989	12	0	0	0	0	0	0
2990	5	0	0	0	0	0	0
2991	4	0	0	0	0	0	0
2992	3	0	0	0	0	0	0
2993	3	0	0	0	0	0	0
2994	2	0	0	0	0	0	0
2995	2	0	0	0	0	0	0
2996	3	0	0	0	0	0	0
2997	4	0	0	0	0	0	0
2998	2	0	0	0	0	1	0
2999	4	0	0	0	0	0	0
3000	4	0	0	0	0	0	0
3001	2	0	0	0	0	0	0
3002	5	0	0	0	0	0	0
3003	3	0	0	0	0	0	0

3004	2	0	0	0	0	0	0
3005	19	0	0	0	1	0	0
3006	2	0	0	0	0	0	0
3007	4	0	0	0	0	0	0
3008	3	0	0	0	0	0	0
3009	4	0	0	0	0	0	0
3010	4	0	0	0	0	0	0
3011	3	0	0	0	0	0	0
3012	3	0	0	0	1	0	0
3013	12	0	0	0	0	0	0
3014	4	0	0	0	0	0	0
3015	7	0	0	0	0	0	0
3016	4	0	0	0	0	0	0
3017	2	0	0	0	0	0	0
3018	4	0	0	0	0	0	0
3019	3	0	0	0	1	0	0
3020	3	0	0	0	0	0	0
3021	2	0	0	0	0	0	0
3022	4	0	0	0	0	0	0
3023	3	0	0	0	1	1	0
3024	2	0	0	0	1	0	0
3025	2	0	0	0	0	0	0
3026	2	0	0	0	0	0	0
3027	2	0	0	0	1	0	0
3028	2	0	0	0	1	0	0
3029	3	0	0	0	0	0	0
3030	2	0	0	0	0	0	0
3031	2	0	0	0	0	0	0
3032	3	0	0	0	0	0	0
3033	2	0	0	0	0	0	0
3034	3	0	0	0	0	0	0
3035	5	0	0	0	0	0	0
3036	3	0	0	0	0	0	0
3037	4	0	0	0	0	0	0
3038	5	0	0	0	0	0	0
3039	4	0	0	0	0	0	0
3040	2	0	0	0	0	0	0
3041	4	0	0	0	0	0	0
3042	2	0	0	0	0	0	0
3043	2	0	0	0	1	1	1
3044	2	0	0	0	1	0	0
3045	4	0	0	0	0	0	0

3046	2	0	0	0	0	0	0
3047	4	0	0	0	0	0	0
3048	2	0	0	0	0	0	0
3049	13	0	0	0	0	0	0
3050	3	0	0	0	0	0	0
3051	2	0	0	0	0	0	0
3052	4	0	0	0	0	0	0
3053	6	0	0	0	0	0	0
3054	4	0	0	0	1	1	0
3055	2	0	0	0	0	0	0
3056	2	0	0	0	0	0	0
3057	2	0	0	0	0	0	0
3058	12	0	0	0	0	0	0
3059	3	0	0	0	1	0	0
3060	2	0	0	0	0	0	0
3061	2	0	0	0	0	0	0
3062	6	0	0	0	1	0	1
3063	6	0	0	0	1	0	0
3064	2	0	0	0	0	0	0
3065	2	0	0	0	0	0	0
3066	4	0	0	0	0	0	0
3067	5	0	0	0	0	0	0
3068	4	0	0	0	0	0	0
3069	6	0	0	0	0	0	0
3070	2	0	0	0	0	0	0
3071	3	0	0	0	0	0	1
3072	6	0	0	0	1	1	0
3073	5	0	0	0	0	0	0
3074	2	0	0	0	0	0	0
3075	4	0	0	0	0	1	0
3076	7	0	0	0	0	1	0
3077	5	0	0	0	0	0	0
3078	3	0	0	0	0	0	0
3079	4	0	0	0	1	0	0
3080	4	0	0	0	0	0	0
3081	4	0	0	0	1	0	0
3082	2	0	0	0	0	0	0
3083	2	0	0	0	1	0	0
3084	2	0	0	0	0	0	0
3085	3	0	0	0	0	0	0
3086	4	0	0	0	0	0	0
3087	2	0	0	0	1	0	0

3088	3	0	0	0	0	0	0
3089	3	0	0	0	0	0	0
3090	2	0	0	0	0	0	0
3091	6	0	0	0	0	0	0
3092	2	0	0	0	0	0	0
3093	2	0	0	0	0	0	0
3094	2	0	0	0	0	0	0
3095	3	0	0	0	1	0	0
3096	2	0	0	0	0	0	0
3097	5	0	0	0	0	0	0
3098	4	0	0	0	0	0	0
3099	4	0	0	0	0	1	1
3100	2	0	0	0	0	0	0
3101	3	0	0	0	1	1	0
3102	5	0	0	0	0	0	0
3103	2	0	0	0	1	0	0
3104	6	0	0	0	0	0	0
3105	2	0	0	0	0	0	0
3106	4	0	0	0	0	0	0
3107	3	0	0	0	0	0	0
3108	12	0	0	0	0	0	0
3109	2	0	0	0	0	0	0
3110	2	0	0	0	0	0	0
3111	2	0	0	0	0	0	0
3112	5	0	0	0	0	0	0
3113	3	0	0	0	0	0	0
3114	2	0	0	0	0	0	0
3115	2	0	0	0	0	0	0
3116	2	0	0	0	0	0	0
3117	4	0	0	0	1	0	0
3118	3	0	0	0	0	1	0
3119	4	0	0	0	0	0	0
3120	5	0	0	0	1	0	0
3121	3	0	0	0	0	0	0
3122	4	0	0	0	0	0	0
3123	7	0	0	0	1	0	0
3124	4	0	0	0	0	0	0
3125	4	0	0	0	0	0	0
3126	4	0	0	0	1	0	0
3127	4	0	0	0	0	0	0
3128	14	0	0	0	1	0	0
3129	2	0	0	0	0	0	0

3130	7	0	0	0	0	0	0
3131	4	0	0	0	0	0	0
3132	4	0	0	0	0	0	0
3133	2	0	0	0	0	0	0
3134	2	0	0	0	0	0	0
3135	3	0	0	0	0	0	0
3136	6	0	0	1	1	0	0
3137	4	0	0	0	1	0	0
3138	8	0	0	0	1	0	0
3139	2	0	0	0	0	0	0
3140	3	0	0	0	0	0	0
3141	4	0	0	0	1	0	0
3142	12	0	0	0	1	1	0
3143	4	0	0	0	0	0	0
3144	5	0	0	0	0	0	0
3145	2	0	0	0	0	0	0
3146	3	0	0	0	0	0	0
3147	4	0	0	0	0	0	0
3148	2	0	0	0	0	0	0
3149	5	0	0	0	0	0	0
3150	3	0	0	0	0	0	0
3151	2	0	0	0	0	0	0
3152	3	0	0	0	0	0	0
3153	5	0	0	0	1	0	0
3154	4	0	0	0	1	1	1
3155	4	0	0	0	0	0	0
3156	2	0	0	0	0	0	0
3157	3	0	0	0	0	0	0
3158	5	0	0	0	0	0	0
3159	2	0	0	0	0	0	0
3160	2	0	0	0	0	0	0
3161	4	0	0	0	0	0	0
3162	4	0	0	0	0	0	0
3163	3	0	0	0	0	0	0
3164	5	0	0	0	0	0	0
3165	4	0	0	0	0	0	0
3166	4	0	0	0	1	0	0
3167	4	0	0	0	0	0	0
3168	3	0	0	0	0	0	0
3169	8	0	0	0	0	0	0
3170	3	0	0	0	0	0	0
3171	6	0	0	0	0	0	0

3172	2	0	0	0	1	0	0
3173	2	0	0	0	0	0	0
3174	18	0	0	0	0	0	0
3175	6	0	0	0	0	0	0
3176	4	0	0	0	0	0	0
3177	2	0	0	0	0	0	0
3178	5	0	0	0	0	0	0
3179	4	0	0	0	0	0	0
3180	2	0	0	0	1	0	0
3181	2	0	0	0	0	0	0
3182	2	0	0	0	0	0	0
3183	9	0	0	0	0	0	0
3184	4	0	0	0	1	0	0
3185	2	0	0	0	0	0	0
3186	2	0	0	0	0	0	0
3187	3	0	0	0	0	0	0
3188	2	0	0	0	0	0	0
3189	2	0	0	0	0	0	0
3190	3	0	0	0	0	0	0
3191	2	0	0	0	0	0	0
3192	2	0	0	0	0	0	0
3193	5	0	0	0	0	0	0
3194	2	0	0	0	0	0	0
3195	2	0	0	0	0	0	0
3196	2	0	0	0	0	0	0
3197	2	0	0	0	0	0	0
3198	3	0	0	0	0	1	0
3199	3	0	0	0	0	0	0
3200	3	0	0	0	0	0	0
3201	2	0	0	0	0	0	0
3202	2	0	0	0	0	0	0
3203	4	0	0	0	0	0	0
3204	4	0	0	0	0	0	0
3205	4	0	0	0	0	0	0
3206	4	0	0	0	0	0	0
3207	2	0	0	0	0	0	0
3208	10	0	0	0	0	1	0
3209	2	0	0	0	0	0	0
3210	3	0	0	0	0	0	0
3211	2	0	0	0	0	0	0
3212	2	0	0	0	0	0	0
3213	4	0	0	0	0	0	0

3214	4	0	0	0	0	0	0
3215	9	0	0	0	0	0	0
3216	3	0	0	0	0	0	0
3217	5	0	0	0	0	0	0
3218	2	0	0	0	0	0	1
3219	4	0	0	0	0	0	0
3220	3	0	0	0	0	0	0
3221	3	0	0	0	0	0	0
3222	3	0	0	0	0	0	0
3223	2	0	0	0	0	0	0
3224	4	0	0	0	0	0	0
3225	3	0	0	0	1	1	0
3226	2	0	0	0	0	0	0
3227	3	0	0	0	0	0	0
3228	3	0	0	0	0	1	0
3229	4	0	0	0	1	1	0
3230	4	0	0	0	0	0	0
3231	4	0	0	0	1	0	0
3232	5	0	0	0	0	0	0
3233	4	0	0	0	1	0	0
3234	2	0	0	0	0	0	0
3235	2	0	0	0	1	1	0
3236	4	0	0	0	1	0	0
3237	6	0	0	0	0	0	0
3238	2	0	0	0	0	0	0
3239	2	0	0	0	0	0	0
3240	13	0	0	0	0	0	0
3241	3	0	0	0	1	1	0
3242	4	0	0	0	0	0	0
3243	4	0	0	0	0	0	0
3244	3	0	0	0	0	0	0
3245	2	0	0	0	0	0	0
3246	5	0	0	0	1	0	0
3247	2	0	0	0	0	0	0
3248	2	0	0	0	0	0	0
3249	4	0	0	0	0	0	0
3250	3	0	0	0	0	0	0
3251	5	0	0	0	0	0	0
3252	5	0	0	1	0	0	0
3253	3	0	0	0	0	0	0
3254	5	0	0	0	0	0	0
3255	2	0	0	0	0	0	0

3256	3	0	0	0	0	0	0
3257	4	0	0	0	0	0	0
3258	7	0	0	0	0	0	0
3259	2	0	0	0	0	0	0
3260	2	0	0	0	0	0	0
3261	4	0	0	0	0	0	0
3262	6	0	0	0	0	0	0
3263	2	0	0	0	0	0	0
3264	4	0	0	0	0	0	0
3265	2	0	0	0	1	1	0
3266	2	0	0	0	0	0	0
3267	4	0	0	0	0	0	0
3268	2	0	0	0	1	0	0
3269	5	0	0	0	0	0	0
3270	6	0	0	0	1	0	0
3271	4	0	0	0	0	0	0
3272	2	0	0	0	1	0	0
3273	4	0	0	0	0	0	0
3274	4	0	0	0	1	0	0
3275	4	0	0	0	0	0	0
3276	2	0	0	0	0	0	0
3277	2	0	0	0	1	0	0
3278	3	0	0	0	0	0	0
3279	2	0	0	0	0	0	0
3280	6	0	0	0	0	0	0
3281	4	0	0	0	1	1	0
3282	2	0	0	0	0	0	0
3283	6	0	0	0	0	0	0
3284	4	0	0	0	0	0	0
3285	3	0	0	0	0	0	0
3286	7	0	0	0	0	0	0
3287	3	0	0	0	0	0	0
3288	3	0	0	0	0	0	0
3289	2	0	0	0	0	0	0
3290	5	0	0	0	0	0	0
3291	5	0	0	0	0	0	0
3292	9	0	0	0	0	0	0
3293	3	0	0	0	0	0	0
3294	4	0	0	0	0	0	0
3295	2	0	0	0	1	0	0
3296	2	0	0	0	0	0	0
3297	3	0	0	0	0	0	0

3298	2	0	0	0	0	0	0
3299	3	0	0	0	0	0	0
3300	2	0	0	0	0	0	0
3301	4	0	0	0	0	0	0
3302	4	0	0	0	0	0	1
3303	4	0	0	0	0	0	0
3304	3	0	0	0	0	0	0
3305	3	0	0	0	0	1	0
3306	2	0	0	0	0	0	0
3307	3	0	0	0	0	0	0
3308	4	0	0	0	0	0	0
3309	5	0	0	0	0	0	0
3310	7	0	0	0	0	0	0
3311	2	0	0	0	0	0	0
3312	4	0	0	0	1	0	0
3313	2	0	0	0	0	0	0
3314	2	0	0	0	0	0	0
3315	4	0	0	0	0	0	0
3316	2	0	0	0	0	0	0
3317	2	0	0	0	0	0	0
3318	4	0	0	0	0	0	0
3319	8	0	0	0	0	0	0
3320	4	0	0	0	0	0	0
3321	8	0	0	0	1	0	0
3322	4	0	0	0	0	0	0
3323	3	0	0	0	0	0	0
3324	4	0	0	0	0	0	0
3325	7	0	0	0	0	0	0
3326	3	0	0	0	0	0	0
3327	2	0	0	0	0	0	0
3328	5	0	0	0	0	0	0
3329	2	0	0	0	0	0	0
3330	2	0	0	0	0	0	0
3331	11	0	0	0	1	1	0
3332	6	0	0	0	0	0	0
3333	3	0	0	0	0	0	0
3334	2	0	0	0	0	0	0
3335	4	0	0	0	0	0	0
3336	11	0	0	0	0	0	0
3337	2	0	0	0	0	0	1
3338	5	0	0	0	1	1	0
3339	2	0	0	0	0	1	0

3340	3	0	0	0	1	0	0
3341	6	0	0	0	0	0	0
3342	3	0	0	0	0	0	0
3343	3	0	0	0	0	0	0
3344	2	0	0	0	0	0	0
3345	4	0	0	0	0	0	0
3346	6	0	0	0	1	0	0
3347	2	0	0	0	0	0	0
3348	7	0	0	0	0	0	0
3349	3	0	0	0	1	1	0
3350	6	0	0	0	0	0	0
3351	2	0	0	0	0	1	0
3352	2	0	0	0	0	0	0
3353	2	0	0	0	0	0	0
3354	4	0	0	0	0	0	0
3355	4	0	0	0	0	0	0
3356	13	0	0	0	0	0	1
3357	4	0	0	0	0	0	0
3358	4	0	0	0	1	0	0
3359	2	0	0	0	0	0	0
3360	7	0	0	0	1	0	0
3361	2	0	0	0	0	0	0
3362	2	0	0	0	1	0	0
3363	4	0	0	0	0	0	0
3364	4	0	0	0	0	0	0
3365	9	0	0	0	0	0	0
3366	2	0	0	0	1	0	0
3367	2	0	0	0	0	0	0
3368	4	0	0	0	0	0	0
3369	4	0	0	0	0	0	0
3370	6	0	0	0	0	0	0
3371	5	0	0	0	0	0	0
3372	2	0	0	0	0	0	0
3373	3	0	0	0	1	0	0
3374	2	0	0	0	0	0	0
3375	2	0	0	0	0	0	0
3376	2	0	0	0	0	0	0
3377	3	0	0	0	1	0	0
3378	3	0	0	0	0	0	0
3379	6	0	0	0	0	0	0
3380	4	0	0	0	0	0	0
3381	3	0	0	0	0	0	0

3382	6	0	0	0	0	0	0
3383	3	0	0	0	0	0	0
3384	2	0	0	0	0	0	0
3385	4	0	0	0	0	0	0
3386	2	0	0	0	1	0	0
3387	5	0	0	0	0	0	0
3388	5	0	0	0	1	1	0
3389	4	0	0	0	0	0	0
3390	5	0	0	0	0	0	0
3391	3	0	0	0	1	0	0
3392	2	0	0	0	0	0	0
3393	3	0	0	0	0	0	0
3394	2	0	0	0	0	0	0
3395	2	0	0	0	0	0	0
3396	3	0	0	0	1	0	1
3397	5	0	0	0	0	0	0
3398	2	0	0	0	1	0	0
3399	2	0	0	0	0	0	0
3400	3	0	0	0	0	0	0
3401	2	0	0	0	0	0	0
3402	5	0	0	0	0	0	0
3403	4	0	0	0	0	0	0
3404	3	0	0	0	0	0	0
3405	3	0	0	0	0	0	0
3406	2	0	0	0	0	0	0
3407	4	0	0	0	0	0	0
3408	3	0	0	0	0	0	0
3409	5	0	0	0	0	0	0
3410	4	0	0	0	0	0	0
3411	7	0	0	0	0	0	0
3412	2	0	0	0	1	0	1
3413	4	0	0	0	1	1	0
3414	5	0	0	0	0	0	0
3415	5	0	0	0	0	0	0
3416	3	0	0	0	0	0	0
3417	2	0	0	0	0	0	0
3418	5	0	0	0	1	0	0
3419	2	0	0	0	0	0	0
3420	3	0	0	0	0	0	0
3421	2	0	0	0	0	0	0
3422	3	0	0	0	0	0	0
3423	4	0	0	0	0	0	0

3424	4	0	0	0	0	0	0
3425	3	0	0	0	0	0	0
3426	2	0	0	0	0	0	0
3427	5	0	0	0	0	0	0
3428	6	0	0	0	0	0	0
3429	2	0	0	0	0	0	0
3430	2	0	0	0	0	0	0
3431	2	0	0	0	0	0	0
3432	2	0	0	0	0	0	0
3433	2	0	0	0	0	0	0
3434	2	0	0	0	0	0	0
3435	5	0	0	0	0	0	0
3436	3	0	0	0	0	0	0
3437	2	0	0	0	0	0	0
3438	2	0	0	0	0	0	0
3439	3	0	0	0	0	0	0
3440	2	0	0	0	0	0	0
3441	4	0	0	0	0	0	1
3442	5	0	0	0	1	0	0
3443	4	0	0	0	0	0	1
3444	4	0	0	0	0	0	0
3445	2	0	0	0	0	0	0
3446	2	0	0	0	0	0	0
3447	2	0	0	0	0	0	0
3448	2	0	0	0	0	0	0
3449	2	0	0	0	0	0	0
3450	2	0	0	0	0	0	0
3451	2	0	0	0	0	0	0
3452	2	0	0	0	0	0	0
3453	2	0	0	0	0	0	0
3454	2	0	0	0	0	0	0
3455	2	0	0	0	0	0	0
3456	2	0	0	0	1	1	0
3457	2	0	0	0	0	0	0
3458	2	0	0	0	0	0	0
3459	2	0	0	0	0	0	0
3460	2	0	0	0	0	0	0
3461	2	0	0	0	0	0	0
3462	3	0	0	0	0	0	0
3463	3	0	0	0	0	0	0
3464	2	0	0	0	0	0	0
3465	2	0	0	0	0	0	0

3466	5	0	0	0	0	0	0
3467	3	0	0	0	0	0	0
3468	3	0	0	0	1	0	0
3469	2	0	0	0	0	0	0
3470	2	0	0	0	0	0	0
3471	3	0	0	0	0	0	0
3472	3	0	0	0	0	0	0
3473	4	0	0	0	0	0	0
3474	3	0	0	0	0	0	0
3475	4	0	0	0	0	0	0
3476	4	0	0	0	0	0	0
3477	2	0	0	0	0	1	0
3478	3	0	0	0	1	0	0
3479	2	0	0	0	0	0	0
3480	4	0	0	0	1	0	0
3481	4	0	0	0	0	0	0
3482	7	0	0	0	0	0	0
3483	4	0	0	0	0	0	0
3484	2	0	0	0	0	0	0
3485	2	0	0	0	1	0	0
3486	2	0	0	0	0	0	0
3487	2	0	0	0	0	0	0
3488	3	0	0	0	0	0	0
3489	5	0	0	0	1	0	0
3490	4	0	0	0	1	0	0
3491	2	0	0	0	0	0	0
3492	4	0	0	0	1	1	0
3493	2	0	0	0	0	0	0
3494	2	0	0	0	0	0	0
3495	2	0	0	0	0	0	0
3496	2	0	0	0	0	0	0
3497	2	0	0	0	0	0	0
3498	6	0	0	0	0	0	0
3499	2	0	0	0	0	0	0
3500	2	0	0	0	0	0	0
3501	2	0	0	0	0	0	0
3502	4	0	0	0	0	0	0
3503	2	0	0	0	1	0	0
3504	3	0	0	0	0	0	0
3505	2	0	0	0	0	0	0
3506	7	0	0	1	0	0	0
3507	2	0	0	0	0	0	0

3508	4	0	0	0	1	0	0
3509	2	0	0	0	0	0	0
3510	3	0	0	0	0	0	0
3511	4	0	0	0	0	0	0
3512	2	0	0	0	0	0	0
3513	2	0	0	0	0	0	0
3514	5	0	0	0	1	0	0
3515	2	0	0	0	0	0	0
3516	3	0	0	0	0	0	0
3517	2	0	0	0	0	0	0
3518	2	0	0	0	0	0	0
3519	2	0	0	0	0	0	0
3520	2	0	0	0	0	0	0
3521	2	0	0	0	0	0	0
3522	2	0	0	0	0	0	0
3523	5	0	0	0	0	0	0
3524	5	0	0	0	0	0	0
3525	4	0	0	0	0	0	0
3526	2	0	0	0	0	0	0
3527	2	0	0	0	0	0	0
3528	2	0	0	0	0	0	0
3529	2	0	0	0	0	0	0
3530	2	0	0	0	0	0	0
3531	2	0	0	0	0	0	0
3532	4	0	0	0	0	0	0
3533	2	0	0	0	0	0	0
3534	2	0	0	0	0	0	0
3535	2	0	0	0	0	0	0
3536	2	0	0	0	0	0	0
3537	6	0	0	0	0	0	0
3538	2	0	0	0	0	0	0
3539	4	0	0	0	0	0	0
3540	3	0	0	0	0	1	0
3541	3	0	0	0	0	0	0
3542	2	0	0	0	0	0	0
3543	6	0	0	0	0	0	0
3544	4	0	0	0	0	0	0
3545	3	0	0	0	1	1	0
3546	5	0	0	0	0	0	0
3547	17	0	0	0	0	0	0
3548	2	0	0	0	0	0	0

3549	5	0	0	0	0	0	0
3550	2	0	0	0	0	0	0
3551	3	0	0	0	0	1	0
3552	2	0	0	0	0	0	0
3553	2	0	0	0	0	0	0
3554	2	0	0	0	1	1	0
3555	2	0	0	0	0	0	0
3556	2	0	0	0	0	0	0
3557	4	0	0	0	0	0	0
3558	2	0	0	0	0	0	0
3559	4	0	0	0	0	0	0
3560	4	0	0	0	0	0	0
3561	4	0	0	0	1	0	0
3562	4	0	0	0	0	0	0
3563	4	0	0	0	0	0	0
3564	4	0	0	0	1	0	0
3565	4	0	0	0	0	0	0
3566	3	0	0	0	0	0	0
3567	4	0	0	0	0	0	0
3568	3	0	0	0	0	0	0
3569	2	0	0	0	0	0	0
3570	2	0	0	0	0	0	0
3571	2	0	0	0	0	0	0
3572	5	0	0	0	0	0	0
3573	3	0	0	0	0	0	0
3574	3	0	0	0	0	0	0
3575	6	0	0	0	1	1	0
3576	2	0	0	0	0	0	0
3577	5	0	0	0	0	0	0
3578	3	0	0	0	0	0	0
3579	3	0	0	0	0	0	0
3580	3	0	0	0	0	0	0
3581	8	0	0	0	0	0	0
3582	2	0	0	0	0	0	0
3583	2	0	0	0	0	0	0
3584	2	0	0	0	0	0	0
3585	3	0	0	0	0	0	0
3586	2	0	0	0	0	0	0
3587	2	0	0	0	0	0	0
3588	2	0	0	0	0	0	0
3589	2	0	0	0	0	0	0
3590	2	0	0	0	0	0	0

3591	3	0	0	0	1	0	1
3592	2	0	0	0	0	0	0
3593	5	0	0	0	0	0	0
3594	2	0	0	0	0	0	0
3595	2	0	0	0	0	0	0
3596	2	0	0	0	0	0	0
3597	4	0	0	0	0	0	0
3598	2	0	0	0	0	0	0
3599	3	0	0	0	0	0	0
3600	2	0	0	0	1	0	0
3601	3	0	0	0	0	0	0
3602	4	0	0	0	0	0	0
3603	4	0	0	0	0	0	1
3604	4	0	0	0	1	0	0
3605	3	0	0	0	0	0	1
3606	3	0	0	0	0	0	0
3607	6	0	0	0	0	0	0
3608	6	0	0	0	0	0	0
3609	4	0	0	0	0	0	0
3610	2	0	0	0	0	0	0
3611	2	0	0	0	0	0	0
3612	4	0	0	0	0	0	0
3613	4	0	0	0	0	0	0
3614	4	0	0	0	0	0	0
3615	2	0	0	0	1	0	0
3616	4	0	0	0	0	0	0
3617	3	0	0	0	0	0	0
3618	2	0	0	0	0	0	0
3619	12	0	0	0	0	0	0
3620	2	0	0	0	0	0	0
3621	2	0	0	0	0	0	0
3622	2	0	0	0	0	0	0
3623	2	0	0	0	0	0	0
3624	6	0	0	0	0	0	0
3625	2	0	0	0	0	0	0
3626	2	0	0	0	0	0	0
3627	2	0	0	0	0	0	0
3628	2	0	0	0	0	0	0
3629	2	0	0	0	0	0	0
3630	3	0	0	0	1	0	0
3631	4	0	0	0	1	0	0
3632	4	0	0	0	0	0	0

3633	4	0	0	0	1	0	0
3634	3	0	0	0	1	0	0
3635	5	0	0	0	0	1	0
3636	8	0	0	0	0	0	0
3637	8	0	0	0	1	1	0
3638	4	0	0	0	0	0	0
3639	4	0	0	0	0	0	0
3640	3	0	0	0	0	0	0
3641	4	0	0	0	0	0	0
3642	5	0	0	0	0	0	0
3643	4	0	0	0	0	0	0
3644	9	0	0	1	0	0	0
3645	2	0	0	0	0	1	1
3646	2	0	0	0	0	0	1
3647	4	0	0	0	1	1	0
3648	4	0	0	0	0	0	0
3649	5	0	0	0	0	0	0
3650	3	0	0	0	0	0	0
3651	4	0	0	0	0	0	0
3652	4	0	0	0	0	0	0
3653	4	0	0	0	0	0	0
3654	2	0	0	0	0	0	1
3655	6	0	0	0	0	0	0
3656	5	0	0	0	0	0	0
3657	4	0	0	0	0	0	0
3658	7	0	0	0	1	0	0
3659	2	0	0	0	0	0	0
3660	4	0	0	0	0	0	0
3661	2	0	0	0	0	0	0
3662	3	0	0	0	0	0	0
3663	2	0	0	0	0	0	0
3664	2	0	0	0	0	0	0
3665	3	0	0	0	1	0	0
3666	2	0	0	0	0	0	0
3667	19	0	0	0	0	0	0
3668	2	0	0	0	0	0	0
3669	2	0	0	0	1	1	0
3670	2	0	0	0	0	0	0
3671	2	0	0	0	0	0	0
3672	3	0	0	0	1	0	0
3673	4	0	0	0	0	0	0
3674	3	0	0	0	1	0	0

3675	4	0	0	0	0	0	0
3676	2	0	0	0	1	1	0
3677	2	0	0	0	0	0	0
3678	4	0	0	0	0	0	0
3679	2	0	0	0	0	0	0
3680	13	0	0	0	0	0	0
3681	9	0	0	0	1	0	0
3682	3	0	0	0	0	0	0
3683	6	0	0	0	0	0	0
3684	2	0	0	0	0	0	0
3685	2	0	0	0	1	0	0
3686	2	0	0	0	1	1	0
3687	6	0	0	0	0	0	0
3688	2	0	0	0	0	0	0
3689	4	0	0	0	0	0	0
3690	4	0	0	0	0	0	0
3691	4	0	0	0	0	0	0
3692	2	0	0	0	0	0	0
3693	4	0	0	0	1	0	0
3694	4	0	0	0	0	0	0
3695	2	0	0	0	0	0	0
3696	2	0	0	0	0	0	0
3697	2	0	0	0	0	0	0
3698	2	0	0	0	0	0	0
3699	2	0	0	0	0	0	0
3700	5	0	0	0	0	0	0
3701	2	0	0	0	0	0	0
3702	5	0	0	0	0	0	0
3703	2	0	0	0	0	0	0
3704	7	0	0	0	0	0	0
3705	2	0	0	0	0	0	0
3706	2	0	0	0	0	0	0
3707	11	0	0	0	0	0	0
3708	2	0	0	0	0	0	0
3709	2	0	0	0	0	0	0
3710	3	0	0	0	0	0	0
3711	2	0	0	0	0	0	0
3712	5	0	0	0	0	0	0
3713	4	0	0	0	0	0	0
3714	2	0	0	0	0	0	0
3715	3	0	0	0	1	0	0
3716	4	0	0	0	0	0	0

3717	4	0	0	0	0	0	0
3718	5	0	0	0	0	0	0
3719	4	0	0	0	0	0	0
3720	5	0	0	0	0	0	0
3721	2	0	0	0	0	0	0
3722	4	0	0	0	0	0	0
3723	3	0	0	0	0	0	0
3724	2	0	0	0	1	0	0
3725	3	0	0	0	0	0	0
3726	2	0	0	0	0	0	0
3727	4	0	0	0	0	0	0
3728	3	0	0	0	0	0	0
3729	3	0	0	0	1	0	0
3730	6	0	0	0	0	0	0
3731	3	0	0	0	0	1	0
3732	4	0	0	0	0	0	0
3733	4	0	0	0	0	0	0
3734	2	0	0	0	0	0	0
3735	4	0	0	0	0	0	0
3736	2	0	0	0	0	0	0
3737	6	0	0	0	0	0	0
3738	2	0	0	0	0	0	0
3739	2	0	0	0	0	0	0
3740	3	0	0	0	0	0	0
3741	5	0	0	0	0	0	0
3742	5	0	0	0	0	0	0
3743	2	0	0	0	0	0	0
3744	3	0	0	0	0	0	0
3745	12	0	0	0	0	0	0
3746	3	0	0	0	0	0	0
3747	2	0	0	0	0	0	0
3748	4	0	0	0	0	0	0
3749	2	0	0	0	0	0	0
3750	2	0	0	0	0	0	0
3751	2	0	0	0	0	0	0
3752	24	0	0	0	1	0	0
3753	2	0	0	0	0	0	0
3754	4	0	0	0	1	0	0
3755	2	0	0	0	0	0	0
3756	2	0	0	0	0	0	0
3757	7	0	0	1	0	0	0
3758	2	0	0	0	0	0	0

3759	2	0	0	1	0	0	0
3760	2	0	0	0	0	0	0
3761	2	0	0	0	0	0	0
3762	2	0	0	0	0	0	0
3763	4	0	0	0	0	0	0
3764	2	0	0	0	0	0	0
3765	3	0	0	0	0	0	0
3766	4	0	0	0	0	0	0
3767	3	0	0	0	0	0	0
3768	2	0	0	0	0	0	0
3769	2	0	0	0	0	0	0
3770	2	0	0	0	0	0	0
3771	5	0	0	0	0	0	0
3772	10	0	0	0	1	0	0
3773	6	0	0	0	0	0	0
3774	2	0	0	0	0	0	0
3775	2	0	0	0	0	0	0
3776	3	0	0	0	0	0	0
3777	2	0	0	0	0	0	0
3778	2	0	0	0	0	0	0
3779	2	0	0	0	0	0	0
3780	2	0	0	0	0	0	0
3781	2	0	0	0	0	0	0
3782	2	0	0	0	0	0	0
3783	2	0	0	0	0	0	0
3784	2	0	0	0	0	0	0
3785	6	0	0	0	0	0	0
3786	5	0	0	0	0	0	0
3787	5	0	0	0	0	0	0
3788	3	0	0	0	0	0	0
3789	5	0	0	0	0	0	0
3790	3	0	0	0	0	0	0
3791	2	0	0	0	0	0	0
3792	2	0	0	0	0	1	0
3793	3	0	0	0	0	0	0
3794	4	0	0	0	0	0	0
3795	3	0	0	0	0	0	0
3796	4	0	0	0	0	0	0
3797	2	0	0	0	0	0	0
3798	2	0	0	0	0	0	0
3799	2	0	0	0	0	0	0
3800	8	0	0	1	0	0	0
3801	2	0	0	0	0	0	0

3802	5	0	0	0	0	0	0
3803	2	0	0	0	0	0	0
3804	2	0	0	0	0	0	0
3805	2	0	0	0	1	0	0
3806	3	0	0	0	0	0	0
3807	2	0	0	0	0	0	0
3808	2	0	0	0	0	0	0
3809	2	0	0	0	0	0	0
3810	2	0	0	0	0	0	0
3811	2	0	0	0	0	0	0
3812	2	0	0	0	0	0	0
3813	4	0	0	0	0	0	0
3814	3	0	0	0	0	0	0
3815	2	0	0	0	0	0	0
3816	2	0	0	0	1	0	0
3817	4	0	0	0	0	0	0
3818	2	0	0	0	0	1	0
3819	4	0	0	0	0	0	0
3820	4	0	0	0	1	0	1
3821	3	0	0	0	0	0	0
3822	2	0	0	0	0	0	0
3823	3	0	0	0	0	0	0
3824	5	0	0	0	0	0	0
3825	3	0	0	0	0	0	0
3826	2	0	0	1	1	0	0
3827	2	0	0	0	1	0	0
3828	2	0	0	0	0	0	0
3829	5	0	0	0	0	0	1
3830	2	0	0	0	0	0	0
3831	2	0	0	0	0	1	0
3832	4	0	0	0	0	0	0
3833	2	0	0	0	0	0	0
3834	7	0	0	0	0	0	0
3835	3	0	0	0	1	0	0
3836	4	0	0	0	0	0	0
3837	2	0	0	0	0	0	0
3838	2	0	0	0	0	0	0
3839	10	0	0	0	0	0	0
3840	3	0	0	0	0	0	0
3841	2	0	0	0	0	0	0
3842	2	0	0	0	0	0	0
3843	4	0	0	0	0	0	0

3844	2	0	0	0	0	0	0
3845	3	0	0	0	1	0	0
3846	5	0	0	0	0	0	0
3847	3	0	0	0	0	0	0
3848	3	0	0	0	0	0	0
3849	4	0	0	0	0	0	0
3850	3	0	0	0	0	0	0
3851	2	0	0	0	0	0	0
3852	2	0	0	0	0	0	0
3853	4	0	0	0	0	0	0
3854	4	0	0	0	0	0	0
3855	2	0	0	0	0	0	0
3856	2	0	0	0	0	0	0
3857	4	0	0	0	0	0	0
3858	6	0	0	0	0	0	0
3859	2	0	0	0	0	0	0
3860	3	0	0	0	0	0	0
3861	2	0	0	0	0	0	0
3862	3	0	0	0	0	0	0
3863	5	0	0	0	0	0	0
3864	4	0	0	0	0	0	0
3865	2	0	0	0	0	0	0
3866	2	0	0	0	0	0	0
3867	3	0	0	0	1	0	0
3868	4	0	0	0	0	0	0
3869	3	0	0	0	0	0	0
3870	2	0	0	0	0	0	0
3871	5	0	0	0	0	0	0
3872	4	0	0	0	0	0	0
3873	2	0	0	0	1	0	0
3874	2	0	0	0	0	0	0
3875	4	0	0	0	0	0	0
3876	4	0	0	0	0	0	0
3877	2	0	0	0	0	0	0
3878	2	0	0	0	0	0	0
3879	5	0	0	0	0	0	0
3880	4	0	0	0	0	1	0
3881	8	0	0	0	1	0	0
3882	4	0	0	0	0	0	0
3883	5	0	0	0	0	0	0

3884	2	0	0	0	0	0	0
3885	4	0	0	0	0	0	0
3886	4	0	0	0	0	0	0
3887	3	0	0	0	0	0	0
3888	2	0	0	0	1	0	0
3889	2	0	0	0	0	0	0
3890	3	0	0	0	0	0	0
3891	2	0	0	0	0	0	0
3892	2	0	0	0	0	0	0
3893	3	0	0	0	0	0	0
3894	4	0	0	0	1	0	0
3895	2	0	0	0	0	0	0
3896	5	0	0	0	1	0	0
3897	3	0	0	0	0	0	0
3898	2	0	0	0	1	0	0
3899	3	0	0	0	0	0	0
3900	2	0	0	0	0	0	0
3901	4	0	0	0	0	0	0
3902	4	0	0	0	0	0	0
3903	2	0	0	0	0	0	0
3904	2	0	0	0	0	0	0
3905	2	0	0	0	0	0	0
3906	4	0	0	0	0	0	0
3907	3	0	0	0	0	0	0
3908	2	0	0	0	0	0	0
3909	2	0	0	0	0	0	0
3910	2	0	0	0	1	0	0
3911	3	0	0	0	0	0	0
3912	2	0	0	0	0	0	0
3913	3	0	0	0	0	1	1
3914	5	0	0	0	0	0	0
3915	2	0	0	0	1	1	0
3916	2	0	0	0	0	1	0
3917	2	0	0	0	0	0	0
3918	2	0	0	0	0	0	0
3919	3	0	0	0	0	0	0
3920	2	0	0	0	0	0	0
3921	2	0	0	0	0	0	0

3922	3	0	0	0	0	0	0
3923	3	0	0	0	0	0	0
3924	5	0	0	0	0	0	0
3925	2	0	0	0	0	0	0
3926	4	0	0	0	0	1	0
3927	2	0	0	0	0	0	0
3928	2	0	0	0	0	0	0
3929	3	0	0	0	0	0	0
3930	2	0	0	0	0	0	0
3931	3	0	0	0	0	0	0
3932	3	0	0	0	0	0	0
3933	2	0	0	0	0	0	1
3934	5	0	0	0	0	0	0
3935	7	0	0	1	0	0	0
3936	5	0	0	0	0	0	0
3937	3	0	0	0	1	0	0
3938	3	0	0	0	0	0	0
3939	5	0	0	0	1	0	0
3940	4	0	0	0	0	0	0
3941	2	0	0	0	0	0	0
3942	2	0	0	0	1	0	0
3943	2	0	0	0	0	0	0
3944	5	0	0	0	1	0	0
3945	2	0	0	0	1	0	0
3946	4	0	0	0	0	0	0
3947	4	0	0	0	0	0	0
3948	2	0	0	0	0	0	0
3949	2	0	0	0	0	0	0
3950	3	0	0	0	0	0	0
3951	3	0	0	0	0	0	0
3952	4	0	0	0	0	0	0
3953	4	0	0	0	1	0	0
3954	3	0	0	0	0	0	0
3955	3	0	0	0	1	1	0
3956	2	0	0	0	0	0	0
3957	4	0	0	0	1	0	0
3958	2	0	0	0	1	0	0
3959	3	0	0	0	0	0	0
3960	3	0	0	0	0	0	0
3961	3	0	0	0	0	0	0
3962	3	0	0	0	0	0	0

3963	2	0	0	0	0	0	0
3964	2	0	0	0	0	0	0
3965	4	0	0	0	0	0	0
3966	2	0	0	0	1	0	0
3967	2	0	0	0	0	0	0
3968	3	0	0	0	0	0	0
3969	2	0	0	0	0	0	0
3970	4	0	0	0	1	1	0
3971	2	0	0	0	0	0	0
3972	5	0	0	0	0	0	0
3973	4	0	0	0	1	0	0
3974	2	0	0	0	1	0	0
3975	2	0	0	0	0	0	0
3976	8	0	0	0	0	0	0
3977	2	0	0	0	0	0	0
3978	2	0	0	0	0	0	0
3979	4	0	0	0	1	0	0
3980	4	0	0	0	0	0	0
3981	4	0	0	0	0	1	0
3982	4	0	0	0	0	0	0
3983	2	0	0	0	0	0	0
3984	2	0	0	0	0	0	0
3985	4	0	0	0	0	0	0
3986	6	0	0	0	0	0	0
3987	2	0	0	0	1	0	0
3988	4	0	0	0	0	1	0
3989	2	0	0	0	0	0	0
3990	2	0	0	0	0	0	0
3991	2	0	0	0	0	0	0
3992	2	0	0	0	1	0	0
3993	4	0	0	0	0	0	0
3994	3	0	0	0	0	1	0
3995	3	0	0	0	0	0	0
3996	2	0	0	0	0	0	0
3997	4	0	0	0	0	0	0
3998	3	0	0	0	0	0	0
3999	6	0	0	0	0	0	0
4000	2	0	0	0	0	0	0
4001	2	0	0	0	0	0	0
4002	2	0	0	0	0	0	0
4003	2	0	0	0	0	0	0
4004	4	0	0	0	0	0	0

4005	5	0	0	0	1	0	0
4006	2	0	0	0	0	0	0
4007	4	0	0	0	0	0	0
4008	6	0	0	0	0	0	0
4009	3	0	0	0	0	0	0
4010	2	0	0	0	0	0	0
4011	3	0	0	0	0	0	0
4012	3	0	0	0	0	0	0
4013	2	0	0	0	0	0	0
4014	3	0	0	0	0	0	0
4015	3	0	0	0	0	0	0
4016	2	0	0	0	0	0	0
4017	2	0	0	0	0	0	0
4018	3	0	0	0	0	0	0
4019	3	0	0	0	1	0	0
4020	5	0	0	0	1	0	0
4021	5	0	0	0	0	0	0
4022	2	0	0	0	0	0	0
4023	2	0	0	0	0	0	0
4024	4	0	0	0	0	0	0
4025	2	0	0	0	0	0	0
4026	3	0	0	0	0	0	0
4027	6	0	0	0	0	0	0
4028	2	0	0	0	0	0	0
4029	2	0	0	0	0	0	0
4030	4	0	0	0	0	0	0
4031	2	0	0	0	0	0	0
4032	5	0	0	0	0	0	0
4033	3	0	0	0	0	0	0
4034	4	0	0	0	1	1	0
4035	3	0	0	0	0	0	0
4036	4	0	0	0	0	0	0
4037	5	0	0	0	1	0	0
4038	3	0	0	0	0	0	0
4039	5	0	0	0	1	1	0
4040	7	0	0	0	1	0	0
4041	4	0	0	0	1	0	0
4042	5	0	0	0	1	0	0
4043	2	0	0	0	1	0	0
4044	3	0	0	0	0	0	0
4045	2	0	0	0	1	0	0

4046	2	0	0	0	0	0	0
4047	11	0	0	0	0	0	0
4048	2	0	0	0	0	0	0
4049	8	0	0	0	0	0	0
4050	3	0	0	0	1	0	0
4051	3	0	0	0	0	0	0
4052	2	0	0	0	0	0	0
4053	2	0	0	0	1	0	0
4054	3	0	0	0	0	0	1
4055	4	0	0	0	0	0	0
4056	3	0	0	0	1	1	1
4057	4	0	0	0	0	0	0
4058	3	0	0	0	0	0	0
4059	2	0	0	0	0	0	0
4060	2	0	0	0	0	0	0
4061	18	0	0	0	0	0	0
4062	3	0	0	0	0	0	0
4063	2	0	0	0	0	0	0
4064	4	0	0	0	0	0	0
4065	2	0	0	0	0	0	0
4066	2	0	0	0	0	0	0
4067	2	0	0	0	0	0	0
4068	2	0	0	0	1	0	0
4069	5	0	0	0	0	0	0
4070	2	0	0	0	0	0	0
4071	2	0	0	0	1	0	0
4072	5	0	0	0	0	0	0
4073	2	0	0	0	1	0	0
4074	6	0	0	0	0	0	0
4075	2	0	0	0	1	0	0
4076	2	0	0	0	0	0	0
4077	2	0	0	0	0	0	0
4078	3	0	0	0	0	0	0
4079	3	0	0	0	0	0	0
4080	3	0	0	0	0	0	0
4081	3	0	0	0	0	0	0
4082	2	0	0	0	0	0	0
4083	6	0	0	0	0	0	0
4084	3	0	0	0	0	0	0
4085	3	0	0	0	0	0	0
4086	2	0	0	0	0	0	0

4087	2	0	0	0	0	0	0
4088	2	0	0	0	1	0	0
4089	2	0	0	0	0	0	0
4090	2	0	0	0	0	1	0
4091	2	0	0	0	1	0	0
4092	2	0	0	0	0	0	0
4093	4	0	0	0	0	0	0
4094	2	0	0	0	0	0	0
4095	4	0	0	0	1	0	0
4096	2	0	0	0	0	0	0
4097	2	0	0	0	0	0	0
4098	2	0	0	0	0	0	0
4099	6	0	0	0	0	0	0
4100	2	0	0	0	0	0	0
4101	8	0	0	0	0	1	0
4102	3	0	0	0	0	0	0
4103	2	0	0	0	0	0	0
4104	4	0	0	0	0	0	0
4105	4	0	0	0	1	0	0
4106	8	0	0	0	0	0	0
4107	17	0	0	0	0	0	0
4108	3	0	0	0	0	0	0
4109	10	0	0	0	1	0	0
4110	3	0	0	0	0	0	0
4111	7	0	0	0	0	0	0
4112	7	0	0	0	0	0	0
4113	7	0	0	0	0	0	0
4114	4	0	0	0	0	0	0
4115	5	0	0	0	0	0	0
4116	10	0	0	0	0	0	0
4117	9	0	0	0	0	0	0
4118	5	0	0	0	0	0	0
4119	2	0	0	0	0	0	0
4120	3	0	0	0	0	0	0
4121	7	0	0	0	0	0	0
4122	3	0	0	0	1	0	0
4123	3	0	0	0	0	0	0
4124	4	0	0	0	0	0	0
4125	4	0	0	0	0	0	0
4126	3	0	0	0	0	0	0
4127	6	0	0	0	0	0	0
4128	3	0	0	0	0	0	0

4129	9	0	0	0	0	0	0
4130	6	0	0	0	0	0	0
4131	8	0	0	0	0	0	0
4132	6	0	0	0	0	0	0
4133	3	0	0	0	0	0	0
4134	2	0	0	0	0	0	0
4135	2	0	0	0	0	0	0
4136	9	0	0	0	0	0	0
4137	2	0	0	0	0	0	0
4138	3	0	0	0	0	0	0
4139	6	0	0	0	0	0	0
4140	6	0	0	0	0	0	0
4141	2	0	0	0	0	0	0
4142	2	0	0	0	0	0	0
4143	4	0	0	0	0	0	0
4144	5	0	0	0	0	0	0
4145	2	0	0	0	0	0	0
4146	2	0	0	0	0	0	0
4147	8	0	0	0	0	0	0
4148	4	0	0	0	0	0	0
4149	2	0	0	0	0	0	0
4150	5	0	0	0	0	0	0
4151	4	0	0	0	0	0	0
4152	2	0	0	0	0	0	0
4153	4	0	0	0	0	0	0
4154	3	0	0	0	0	0	0
4155	3	0	0	0	0	0	0
4156	4	0	0	0	0	0	0
4157	5	0	0	0	0	0	0
4158	4	0	0	0	1	1	1
4159	4	0	0	0	0	0	1
4160	3	0	0	0	1	1	1
4161	4	0	0	0	1	1	1
4162	4	0	0	0	1	1	1
4163	7	0	0	0	1	1	1
4164	2	0	0	0	1	1	1
4165	2	0	0	0	0	0	1
4166	7	0	0	0	1	1	1
4167	3	0	0	0	1	1	1
4168	2	0	0	0	0	0	1
4169	7	0	0	0	0	0	1

4170	13	0	0	0	1	0	1
4171	2	0	0	0	1	1	1
4172	5	0	0	0	1	1	1
4173	2	0	0	0	1	1	1
4174	9	0	0	0	1	1	1
4175	3	0	0	0	1	0	1
4176	5	0	0	0	1	0	1
4177	5	0	0	0	1	1	1
4178	3	0	0	0	1	1	1
4179	4	0	0	0	1	1	1
4180	6	0	0	0	1	1	1
4181	2	0	0	0	1	1	1
4182	13	0	0	0	1	0	1
4183	5	0	0	0	1	1	1
4184	2	0	0	0	1	0	1
4185	7	0	0	0	1	0	1
4186	5	0	0	0	0	0	0
4187	4	0	0	0	1	1	1
4188	2	0	0	0	1	1	1
4189	10	0	0	0	1	1	1
4190	7	0	0	0	1	1	1
4191	2	0	0	0	1	1	1
4192	2	0	0	0	1	1	1
4193	3	0	0	0	1	1	1
4194	5	0	0	0	1	0	1
4195	2	0	0	0	1	1	1
4196	5	0	0	0	1	1	1
4197	4	0	0	0	0	1	1
4198	4	0	0	0	0	1	1
4199	5	0	0	0	1	1	1
4200	2	0	0	0	1	1	1
4201	4	0	0	0	1	0	1
4202	4	0	0	0	1	0	1
4203	3	0	0	0	1	1	1
4204	4	0	0	0	0	0	0
4205	3	0	0	0	1	1	1
4206	3	0	0	0	1	1	1
4207	4	0	0	0	0	1	1
4208	2	0	0	0	1	0	1
4209	2	0	0	0	1	0	1
4210	4	0	0	0	0	0	1
4211	4	0	0	0	1	1	1

4212	4	0	0	0	1	0	1
4213	8	0	0	0	1	0	1
4214	4	0	0	0	1	0	0
4215	4	0	0	0	0	0	0
4216	3	0	0	0	1	0	1
4217	2	0	0	0	1	0	1
4218	4	0	0	0	1	1	1
4219	9	0	0	0	1	0	1
4220	4	0	0	0	1	0	1
4221	5	0	0	0	1	0	0
4222	4	0	0	0	1	0	0
4223	4	0	0	0	1	0	1
4224	6	0	0	0	1	0	1
4225	7	0	0	0	1	0	1
4226	4	0	0	0	0	0	0
4227	5	0	0	0	1	0	1
4228	4	0	0	0	0	0	1
4229	2	0	0	0	1	0	1
4230	4	0	0	0	1	1	1
4231	21	0	0	0	0	0	1
4232	5	0	0	0	0	0	1
4233	3	0	0	0	1	0	1
4234	2	0	0	0	1	1	1
4235	4	0	0	0	1	0	1
4236	7	0	0	0	1	1	1
4237	11	0	0	0	1	1	1
4238	12	0	0	0	1	0	1
4239	4	0	0	0	0	0	1
4240	2	0	0	0	1	1	1
4241	4	0	0	0	1	1	1
4242	2	0	0	0	1	1	1
4243	4	0	0	0	1	0	1
4244	13	0	0	0	1	1	1
4245	5	0	0	0	1	1	1
4246	11	0	0	0	1	0	1
4247	4	0	0	0	0	0	1
4248	5	0	0	0	1	0	1
4249	5	0	0	0	1	1	1
4250	12	0	0	0	1	0	1
4251	5	0	0	0	1	0	1
4252	2	0	0	0	1	0	1

4253	4	0	0	0	1	0	1
4254	3	0	0	0	1	1	1
4255	2	0	0	0	1	1	1
4256	5	0	0	0	0	0	1
4257	8	0	0	0	1	0	1
4258	8	0	0	0	1	0	1
4259	6	0	0	0	0	1	1
4260	2	0	0	0	1	1	1
4261	3	0	0	0	1	0	1
4262	4	0	0	0	1	1	1
4263	4	0	0	0	1	1	1
4264	2	0	0	0	0	1	1
4265	5	0	0	0	1	0	1
4266	4	0	0	0	1	1	1
4267	2	0	0	0	1	0	1
4268	6	0	0	0	1	1	1
4269	3	0	0	0	1	1	1
4270	5	0	0	0	1	0	1
4271	2	0	0	0	1	1	1
4272	4	0	0	0	0	1	1
4273	5	0	0	0	1	0	0
4274	9	0	0	0	1	0	1
4275	9	0	0	0	1	1	1
4276	4	0	0	0	1	1	1
4277	4	0	0	0	1	1	1
4278	2	0	0	0	1	1	1
4279	6	0	0	0	1	1	1
4280	2	0	0	0	0	0	0
4281	2	0	0	0	0	0	1
4282	4	0	0	0	1	0	0
4283	6	0	0	0	1	1	1
4284	3	0	0	0	1	1	1
4285	4	0	0	0	0	0	1
4286	4	0	0	0	1	1	1
4287	4	0	0	0	1	1	1
4288	3	0	0	0	1	0	1
4289	2	0	0	0	1	1	0
4290	2	0	0	0	1	1	1
4291	2	0	0	0	0	1	1
4292	4	0	0	0	1	1	1
4293	4	0	0	0	1	1	1
4294	3	0	0	1	1	1	1

4295	5	0	0	0	1	1	1
4296	5	0	0	0	1	1	1
4297	2	0	0	0	1	1	1
4298	2	0	0	0	1	1	1
4299	2	0	0	0	0	0	1
4300	2	0	0	0	1	1	1
4301	2	0	0	0	1	0	0
4302	5	0	0	0	1	1	1
4303	8	0	0	0	1	0	1
4304	6	0	0	0	1	0	1
4305	2	0	0	0	1	0	1
4306	5	0	0	0	0	0	1
4307	6	0	0	0	1	1	1
4308	4	0	0	0	0	0	1
4309	9	0	0	0	1	0	1
4310	5	0	0	0	1	1	1
4311	3	0	0	0	0	0	1
4312	4	0	0	0	0	0	1
4313	5	0	0	0	1	1	1
4314	7	0	0	0	0	0	1
4315	4	0	0	0	0	1	1
4316	3	0	0	0	1	1	1
4317	9	0	0	0	1	1	1
4318	6	0	0	0	0	0	1
4319	8	0	0	0	1	0	1
4320	2	0	0	0	1	0	1
4321	15	0	0	0	1	0	1
4322	3	0	0	0	1	1	1
4323	5	0	0	0	1	0	1
4324	10	0	0	0	1	1	1
4325	5	0	0	0	1	1	0
4326	5	0	0	0	1	1	1
4327	2	0	0	0	1	0	1
4328	2	0	0	0	1	1	1
4329	4	0	0	0	1	1	1
4330	7	0	0	0	0	0	1
4331	5	0	0	0	0	1	1
4332	2	0	0	0	0	0	1
4333	4	0	0	0	0	1	1
4334	2	0	0	0	1	1	1
4335	2	0	0	0	1	0	1
4336	2	0	0	0	1	1	1

4337	2	0	0	0	0	0	1
4338	2	0	0	0	1	1	1
4339	4	0	0	0	1	1	1
4340	2	0	0	0	0	0	1
4341	2	0	0	0	0	0	1
4342	4	0	0	0	1	0	1
4343	2	0	0	0	1	0	1
4344	2	0	0	0	1	0	1
4345	3	0	0	0	0	0	1
4346	15	0	0	1	0	0	1
4347	4	0	0	0	0	0	1
4348	4	0	0	0	0	0	1
4349	9	0	0	0	1	0	1
4350	4	0	0	0	1	1	1
4351	4	0	0	0	0	0	1
4352	9	0	0	0	1	0	1
4353	2	0	0	0	0	0	0
4354	8	0	0	0	1	0	0
4355	5	0	0	0	0	0	0
4356	2	0	0	0	0	0	0
4357	5	0	0	0	0	1	0
4358	5	0	0	0	0	0	1
4359	2	0	0	0	1	0	0
4360	3	0	0	0	1	0	0
4361	6	0	0	0	0	0	0
4362	11	0	0	0	0	0	0
4363	2	0	0	0	0	0	0
4364	4	0	0	0	1	1	0
4365	2	0	0	0	0	0	0
4366	11	0	0	0	0	1	0
4367	6	0	0	0	0	0	0
4368	2	0	0	0	0	0	0
4369	4	0	0	0	0	0	0
4370	4	0	0	0	0	0	0
4371	4	0	0	0	0	0	0
4372	6	0	0	0	1	0	0
4373	3	0	0	0	0	0	0
4374	2	0	0	0	1	0	0
4375	2	0	0	0	1	1	0
4376	3	0	0	0	1	0	0
4377	4	0	0	0	1	0	0

4378	8	0	0	0	1	0	0
4379	7	0	0	0	0	0	0
4380	2	0	0	0	0	0	0
4381	4	0	0	0	1	0	1
4382	4	0	0	0	1	0	0
4383	3	0	0	0	1	1	0
4384	5	0	0	0	0	0	0
4385	3	0	0	0	0	0	1
4386	6	0	0	0	0	0	0
4387	2	0	0	0	1	0	1
4388	3	0	0	0	0	0	0
4389	7	0	0	0	1	1	0
4390	4	0	0	0	1	0	0
4391	5	0	0	0	0	0	0
4392	4	0	0	0	0	0	0
4393	3	0	0	0	0	1	0
4394	2	0	0	0	0	0	0
4395	4	0	0	0	0	0	0
4396	4	0	0	0	0	0	0
4397	3	0	0	0	1	0	0
4398	2	0	0	0	0	0	0
4399	7	0	0	0	1	0	0
4400	3	0	0	0	0	0	0
4401	8	0	0	0	0	0	0
4402	2	0	0	0	1	1	0
4403	3	0	0	0	0	0	0
4404	2	0	0	0	0	0	0
4405	2	0	0	0	0	0	0
4406	4	0	0	0	0	0	0
4407	3	0	0	0	0	0	0
4408	2	0	0	0	0	0	0
4409	2	0	0	0	1	0	0
4410	10	0	0	0	1	1	0
4411	3	0	0	0	1	0	0
4412	17	0	0	0	0	0	0
4413	4	0	0	0	0	0	0
4414	4	0	0	0	0	0	0
4415	4	0	0	0	1	0	0
4416	4	0	0	0	0	0	0
4417	2	0	0	0	0	0	0
4418	11	0	0	0	0	0	0
4419	12	0	0	0	0	0	0

4420	4	0	0	0	0	0	0
4421	3	0	0	0	1	1	0
4422	12	0	0	0	0	0	0
4423	3	0	0	0	0	0	0
4424	3	0	0	0	1	1	0
4425	13	0	0	0	0	0	0
4426	2	0	0	0	1	0	0
4427	3	0	0	0	0	1	0
4428	4	0	0	0	1	1	1
4429	4	0	0	0	0	0	0
4430	4	0	0	0	1	0	0
4431	2	0	0	0	1	0	0
4432	4	0	0	0	1	1	1
4433	2	0	0	0	1	0	0
4434	4	0	0	0	1	0	0
4435	2	0	0	0	0	0	0
4436	10	0	0	0	1	0	0
4437	3	0	0	0	0	1	0
4438	5	0	0	0	1	0	0
4439	2	0	0	0	0	0	0
4440	7	0	0	0	0	0	0
4441	5	0	0	0	0	0	0
4442	22	0	0	0	1	1	0
4443	4	0	0	0	0	0	0
4444	2	0	0	0	0	0	0
4445	3	0	0	0	0	1	0
4446	11	0	0	0	0	0	0
4447	10	0	0	0	1	1	0
4448	2	0	0	0	0	0	0
4449	2	0	0	0	1	0	0
4450	3	0	0	0	0	0	0
4451	6	0	0	0	1	0	0
4452	4	0	0	0	0	0	0
4453	2	0	0	0	1	0	0
4454	7	0	0	0	0	0	0
4455	2	0	0	0	0	0	0
4456	2	0	0	0	0	0	1
4457	8	0	0	0	1	0	0
4458	4	0	0	0	1	1	0
4459	4	0	0	0	0	0	1
4460	4	0	0	0	0	0	0

4461	4	0	0	0	1	0	0
4462	2	0	0	0	0	0	0
4463	6	0	0	0	1	0	0
4464	4	0	0	0	0	0	0
4465	2	0	0	0	0	0	0
4466	2	0	0	0	1	0	0
4467	4	0	0	0	0	0	0
4468	12	0	0	0	0	0	0
4469	2	0	0	0	1	0	0
4470	3	0	0	0	1	1	0
4471	4	0	0	0	0	0	1
4472	2	0	0	0	0	0	0
4473	4	0	0	0	0	0	0
4474	4	0	0	0	0	0	0
4475	3	0	0	0	1	0	0
4476	2	0	0	0	0	0	0
4477	3	0	0	0	1	1	0
4478	3	0	0	0	1	0	0
4479	3	0	0	0	0	0	0
4480	3	0	0	0	0	1	0
4481	4	0	0	0	0	0	0
4482	4	0	0	0	0	0	1
4483	2	0	0	0	0	1	0
4484	2	0	0	0	1	0	0
4485	6	0	0	0	1	0	0
4486	5	0	0	0	1	0	0
4487	5	0	0	0	0	0	0
4488	2	0	0	0	1	0	0
4489	17	0	0	0	0	0	0
4490	2	0	0	0	0	1	0
4491	5	0	0	0	1	0	0
4492	2	0	0	0	0	0	0
4493	6	0	0	0	0	0	0
4494	9	0	0	0	0	0	0
4495	4	0	0	0	0	0	1
4496	5	0	0	0	0	0	0
4497	4	0	0	0	1	0	0
4498	3	0	0	0	0	0	0
4499	7	0	0	0	1	1	0
4500	2	0	0	0	0	0	0
4501	2	0	0	0	0	0	0

4502	2	0	0	0	0	0	0
4503	2	0	0	0	1	0	0
4504	6	0	0	0	0	0	0
4505	4	0	0	0	0	0	0
4506	2	0	0	0	0	0	0
4507	4	0	0	0	0	1	0
4508	2	0	0	0	0	0	0
4509	2	0	0	0	1	0	0
4510	3	0	0	0	0	0	0
4511	5	0	0	0	0	0	0
4512	2	0	0	0	1	0	0
4513	3	0	0	0	0	0	1
4514	11	0	0	0	0	0	0
4515	3	0	0	0	0	0	0
4516	4	0	0	0	0	0	0
4517	8	0	0	0	0	0	0
4518	4	0	0	0	0	1	0
4519	4	0	0	0	1	1	0
4520	5	0	0	0	0	0	0
4521	5	0	0	0	0	0	0
4522	7	0	0	0	0	0	0
4523	4	0	0	0	0	0	0
4524	2	0	0	0	0	0	0
4525	4	0	0	0	0	0	0
4526	8	0	0	0	0	0	0
4527	2	0	0	0	1	0	1
4528	2	0	0	0	0	0	0
4529	2	0	0	0	0	0	0
4530	3	0	0	0	1	0	0
4531	3	0	0	0	1	0	0
4532	3	0	0	0	1	0	0
4533	2	0	0	0	0	0	0
4534	2	0	0	0	1	0	1
4535	2	0	0	0	0	0	0
4536	3	0	0	0	1	1	0
4537	5	0	0	0	1	0	0
4538	2	0	0	0	1	0	0
4539	2	0	0	0	0	0	0
4540	7	0	0	0	0	0	0
4541	2	0	0	0	0	0	0
4542	5	0	0	0	0	0	0
4543	2	0	0	0	0	0	0

4544	8	0	0	0	0	0	0
4545	3	0	0	0	0	0	0
4546	6	0	0	0	1	0	0
4547	2	0	0	0	0	0	0
4548	2	0	0	0	1	0	0
4549	2	0	0	0	0	0	0
4550	3	0	0	0	0	0	0
4551	4	0	0	0	1	1	0
4552	2	0	0	0	1	0	0
4553	4	0	0	0	1	0	0
4554	2	0	0	0	0	0	1
4555	2	0	0	0	0	0	0
4556	9	0	0	1	0	1	0
4557	5	0	0	0	1	0	0
4558	2	0	0	0	0	0	0
4559	7	0	0	0	0	0	0
4560	8	0	0	0	0	0	0
4561	4	0	0	0	1	0	1
4562	4	0	0	0	0	0	0
4563	2	0	0	0	0	0	0
4564	2	0	0	0	0	0	0
4565	27	0	0	0	1	0	0
4566	5	0	0	0	0	0	0
4567	2	0	0	0	0	0	0
4568	2	0	0	0	1	0	0
4569	5	0	0	0	0	0	0
4570	2	0	0	1	0	0	1
4571	7	0	0	0	0	0	0
4572	5	0	0	0	0	0	0
4573	2	0	0	0	0	0	0
4574	6	0	0	0	1	0	0
4575	3	0	0	0	0	0	0
4576	2	0	0	0	0	0	0
4577	2	0	0	0	1	0	0
4578	6	0	0	0	0	0	0
4579	2	0	0	0	0	0	0
4580	2	0	0	0	0	1	0
4581	2	0	0	0	0	0	0
4582	14	0	0	0	0	0	0
4583	4	0	0	0	0	0	0
4584	4	0	0	0	0	0	0
4585	2	0	0	0	1	0	0

4586	2	0	0	0	1	0	0
4587	5	0	0	0	1	0	0
4588	7	0	0	0	1	0	0
4589	6	0	0	1	0	0	0
4590	2	0	0	0	0	0	0
4591	4	0	0	0	0	0	0
4592	7	0	0	0	0	0	0
4593	4	0	0	0	1	0	0
4594	6	0	0	0	1	0	0
4595	2	0	0	0	0	0	0
4596	7	0	0	0	0	0	0
4597	3	0	0	0	1	1	0
4598	2	0	0	0	0	0	0
4599	6	0	0	0	1	1	0
4600	2	0	0	0	0	0	0
4601	2	0	0	0	1	0	0
4602	13	0	0	0	1	0	0
4603	2	0	0	0	0	0	0
4604	2	0	0	0	0	0	0
4605	4	0	0	0	0	0	0
4606	3	0	0	0	0	0	0
4607	4	0	0	0	0	0	0
4608	3	0	0	0	0	0	0
4609	5	0	0	0	0	0	0
4610	2	0	0	0	1	0	0
4611	3	0	0	0	0	0	0
4612	2	0	0	0	1	1	0
4613	4	0	0	0	1	0	0
4614	5	0	0	0	0	0	0
4615	2	0	0	0	0	0	0
4616	2	0	0	0	0	0	0
4617	3	0	0	0	0	0	0
4618	4	0	0	0	1	1	0
4619	2	0	0	0	1	0	0
4620	2	0	0	0	0	1	0
4621	2	0	0	0	0	0	0
4622	9	0	0	0	1	0	0
4623	10	0	0	0	0	1	1
4624	3	0	0	0	0	0	0
4625	3	0	0	0	1	1	1
4626	2	0	0	0	1	0	0
4627	2	0	0	0	0	0	0

4628	2	0	0	0	1	1	1
4629	2	0	0	0	1	0	0
4630	2	0	0	1	0	0	0
4631	8	0	0	0	1	0	0
4632	10	0	0	0	0	1	1
4633	3	0	0	0	1	0	0
4634	4	0	0	0	1	0	0
4635	2	0	0	0	0	0	0
4636	4	0	0	0	0	0	1
4637	3	0	0	0	0	0	0
4638	2	0	0	0	0	0	0
4639	17	0	0	0	0	0	0
4640	2	0	0	0	1	0	0
4641	2	0	0	0	0	0	0
4642	5	0	0	0	0	0	0
4643	2	0	0	0	0	0	0
4644	5	0	0	0	0	0	1
4645	6	0	0	0	0	0	0
4646	2	0	0	0	1	1	0
4647	5	0	0	0	0	0	0
4648	4	0	0	0	0	0	0
4649	3	0	0	0	0	0	0
4650	2	0	0	0	1	0	0
4651	2	0	0	0	1	0	0
4652	2	0	0	0	0	0	0
4653	10	0	0	0	0	0	0
4654	3	0	0	0	0	0	0
4655	2	0	0	0	0	0	0
4656	9	0	0	0	0	0	0
4657	2	0	0	0	0	0	0
4658	5	0	0	0	0	0	0
4659	4	0	0	0	0	0	0
4660	3	0	0	0	0	0	0
4661	2	0	0	0	1	0	0
4662	3	0	0	0	1	0	0
4663	7	0	0	0	0	0	0
4664	2	0	0	0	0	0	1
4665	2	0	0	0	0	0	0
4666	2	0	0	0	0	0	0
4667	2	0	0	0	1	0	0
4668	5	0	0	0	0	0	0

4669	7	0	0	0	1	0	0
4670	4	0	0	0	0	1	0
4671	2	0	0	0	1	0	0
4672	9	0	0	0	0	0	0
4673	5	0	0	0	1	0	0
4674	5	0	0	0	0	0	0
4675	4	0	0	0	0	0	0
4676	8	0	0	0	1	0	0
4677	2	0	0	0	0	0	0
4678	2	0	0	0	0	0	0
4679	2	0	0	0	1	0	0
4680	2	0	0	0	0	1	0
4681	6	0	0	0	1	0	0
4682	4	0	0	0	1	0	0
4683	3	0	0	0	0	0	1
4684	2	0	0	0	1	0	0
4685	2	0	0	0	0	0	0
4686	5	0	0	0	1	0	0
4687	2	0	0	0	0	0	0
4688	10	0	0	0	1	0	0
4689	3	0	0	0	1	0	1
4690	2	0	0	0	1	0	0
4691	4	0	0	0	0	0	0
4692	2	0	0	0	0	0	0
4693	3	0	0	0	0	0	0
4694	3	0	0	0	0	0	0
4695	2	0	0	0	0	0	0
4696	17	0	0	0	0	0	0
4697	4	0	0	0	0	0	0
4698	3	0	0	0	1	1	1
4699	2	0	0	1	0	0	0
4700	3	0	0	0	0	0	1
4701	2	0	0	0	1	0	1
4702	2	0	0	0	0	0	0
4703	7	0	0	0	1	0	0
4704	2	0	0	0	1	0	0
4705	3	0	0	0	0	0	0
4706	2	0	0	0	0	0	0
4707	2	0	0	0	0	0	0
4708	6	0	0	0	0	0	0
4709	5	0	0	0	0	0	0

4710	2	0	0	0	0	0	0
4711	2	0	0	0	0	0	0
4712	2	0	0	0	0	0	0
4713	6	0	0	0	1	0	0
4714	16	0	0	0	1	1	0
4715	5	0	0	0	1	0	0
4716	2	0	0	0	0	0	0
4717	3	0	0	0	1	0	0
4718	7	0	0	0	0	1	0
4719	3	0	0	0	1	0	0
4720	2	0	0	0	1	0	0
4721	3	0	0	0	1	0	1
4722	5	0	0	0	1	1	1
4723	5	0	0	0	1	1	1
4724	2	0	0	0	1	0	1
4725	6	0	0	0	1	0	1
4726	2	0	0	0	1	0	1
4727	4	0	0	0	1	0	1
4728	12	0	0	0	1	1	1
4729	4	0	0	0	1	1	1
4730	4	0	0	0	1	1	1
4731	10	0	0	0	1	0	1
4732	2	0	0	0	1	0	1
4733	5	0	0	0	1	1	1
4734	8	0	0	0	1	0	1
4735	2	0	0	0	1	1	1
4736	15	0	0	0	1	0	1
4737	5	0	0	0	1	1	1
4738	3	0	0	0	1	0	1
4739	3	0	0	0	1	0	1
4740	4	0	0	0	0	0	1
4741	4	0	0	0	1	1	1
4742	5	0	0	0	1	1	1
4743	5	0	0	0	1	1	1
4744	7	0	0	0	1	1	1
4745	4	0	0	0	1	0	1
4746	4	0	0	0	1	0	0
4747	6	0	0	0	1	1	1
4748	5	0	0	0	1	1	1
4749	5	0	0	0	1	1	1
4750	4	0	0	0	1	1	1

4751	2	0	0	0	1	1	1
4752	3	0	0	0	1	1	1
4753	15	0	0	0	1	1	1
4754	3	0	0	0	1	1	1
4755	5	0	0	0	1	0	1
4756	3	0	0	0	1	1	1
4757	2	0	0	0	1	1	1
4758	5	0	0	0	1	1	1
4759	8	0	0	0	1	0	1
4760	5	0	0	0	1	0	1
4761	8	0	0	0	1	1	1
4762	7	0	0	0	1	1	1
4763	2	0	0	0	1	0	1
4764	4	0	0	0	0	0	1
4765	6	0	0	0	0	1	1
4766	4	0	0	0	1	1	1
4767	5	0	0	0	1	0	1
4768	14	0	0	0	0	0	1
4769	2	0	0	0	1	1	1
4770	2	0	0	0	1	1	1
4771	4	0	0	0	0	0	1
4772	6	0	0	0	1	0	1
4773	3	0	0	0	1	1	1
4774	5	0	0	0	1	1	1
4775	2	0	0	0	1	0	1
4776	8	0	0	0	1	0	1
4777	8	0	0	0	1	0	1
4778	4	0	0	0	0	1	1
4779	6	0	0	0	1	0	1
4780	5	0	0	0	1	0	1
4781	6	0	0	0	1	0	1
4782	5	0	0	0	0	0	1
4783	3	0	0	0	1	1	1
4784	2	0	0	0	1	1	1
4785	12	0	0	0	1	0	1
4786	3	0	0	0	1	1	1
4787	4	0	0	0	0	0	1
4788	2	0	0	0	1	1	1
4789	8	0	0	0	0	0	1
4790	4	0	0	0	1	0	1
4791	2	0	0	0	1	0	0

4792	9	0	0	0	1	1	1
4793	5	0	0	0	1	1	1
4794	3	0	0	0	1	1	0
4795	4	0	0	0	1	0	1
4796	3	0	0	0	0	1	1
4797	2	0	0	0	0	0	1
4798	2	0	0	0	1	0	1
4799	2	0	0	0	1	1	1
4800	12	0	0	0	1	1	1
4801	5	0	0	0	1	0	1
4802	2	0	0	0	1	0	1
4803	13	0	0	0	1	0	1
4804	3	0	0	0	0	1	1
4805	7	0	0	0	1	1	1
4806	5	0	0	0	1	1	1
4807	7	0	0	0	1	1	1
4808	2	0	0	0	1	0	0
4809	2	0	0	0	0	0	1
4810	6	0	0	0	1	0	1
4811	4	0	0	0	1	1	1
4812	10	0	0	0	1	1	1
4813	16	0	0	0	1	0	1
4814	5	0	0	0	1	0	1
4815	2	0	0	0	1	0	1
4816	2	0	0	0	1	0	0
4817	2	0	0	0	1	1	1
4818	2	0	0	0	0	0	1
4819	3	0	0	0	1	1	1
4820	2	0	0	0	1	0	1
4821	3	0	0	0	1	1	1
4822	3	0	0	0	1	1	1
4823	2	0	0	0	0	0	1
4824	2	0	0	0	1	1	1
4825	5	0	0	0	1	0	1
4826	2	0	0	0	1	1	1
4827	5	0	0	0	1	0	1
4828	3	0	0	0	0	0	1
4829	3	0	0	0	1	1	1
4830	12	0	0	0	1	1	1
4831	4	0	0	0	1	1	1
4832	3	0	0	0	0	0	1
4833	5	0	0	0	0	0	0

4834	6		0	0	0	1	1	1
4835	2		0	0	0	1	1	1
4836	6		0	0	0	1	0	1
4837	7		0	0	0	1	0	1
4838	3		0	0	0	0	0	1
4839	4		0	0	0	1	0	1
4840	2		0	0	0	0	0	1
4841	3		0	0	0	0	1	1
4842	5		0	0	0	1	1	1
4843	4		0	0	0	0	0	1
4844	2		0	0	0	0	0	1
4845	3		0	0	0	1	1	1
4846	4		0	0	0	0	0	1
4847	2		0	0	0	1	1	1
4848	2		0	0	0	1	1	1
4849	2		0	0	0	1	0	1
4850	4		0	0	0	1	1	1
4851	8		0	0	0	1	1	1
4852	4		0	0	0	1	0	1
4853	3		0	0	0	0	0	1
4854	8		0	0	0	1	0	1
4855	6		0	0	0	1	1	1
4856	5		0	0	0	1	0	1
4857	5		0	0	0	1	0	1
4858	5		0	0	0	0	0	1
4859	3	0.3333	1	1	0	0	0	0
4860	10	0.1	1	1	0	1	0	0
4861	4	0.25	1	1	0	0	1	0
4862	4	0.25	1	1	0	1	1	1
4863	5	0.2	1	1	0	0	1	0
4864	6	0.1667	1	1	0	0	0	0
4865	3	0.3333	1	1	0	0	0	0
4866	7	0.1429	1	1	0	0	0	0
4867	5	0.2	1	1	0	0	0	0
4868	2	0.5	1	1	0	0	0	0
4869	11	0.1818	2	1	0	1	0	0
4870	4	0.5	2	1	0	1	1	0
4871	4	0.5	2	1	0	1	0	0
4872	2	0.5	1	1	1	1	0	0
4873	2	0.5	1	1	0	0	0	0
4874	2	0.5	1	1	0	0	0	0

4875	5	0.2	1	1	0	1	0	0
4876	3	0.3333	1	1	0	0	0	0
4877	3	0.3333	1	1	0	1	0	0
4878	2	0.5	1	1	0	0	0	0
4879	3	0.3333	1	1	0	1	0	0
4880	4	0.25	1	1	0	1	1	0
4881	4	0.25	1	1	0	0	1	1
4882	5	0.2	1	1	0	1	0	0
4883	2	0.5	1	1	0	1	1	1
4884	9	0.1111	1	1	0	0	0	1
4885	6	0.1667	1	1	0	1	1	1
4886	10	0.4	4	1	0	1	1	1
4887	4	0.25	1	1	0	0	0	0
4888	6	0.1667	1	1	0	0	0	0
4889	2	0.5	1	1	0	0	0	0
4890	3	0.3333	1	1	0	0	0	0
4891	4	0.25	1	1	0	0	0	0
4892	3	0.3333	1	1	0	0	0	0
4893	6	0.1667	1	1	0	0	0	1
4894	7	0.4286	3	1	0	1	1	1
4895	5	0.2	1	1	0	0	0	0
4896	10	0.1	1	1	0	0	0	0
4897	5	0.2	1	1	0	0	0	0
4898	4	0.25	1	1	0	1	0	0
4899	5	0.2	1	1	0	0	0	0
4900	2	0.5	1	1	0	0	0	0
4901	4	0.25	1	1	0	0	0	0
4902	6	0.1667	1	1	0	0	0	0
4903	3	0.3333	1	1	0	0	0	0
4904	5	0.2	1	1	0	0	0	0
4905	3	0.3333	1	1	0	1	0	1
4906	4	0.25	1	1	0	0	0	0
4907	2	0.5	1	1	0	0	0	0
4908	6	0.3333	2	1	0	0	0	0
4909	5	0.2	1	1	0	0	0	0
4910	3	0.6667	2	1	1	0	0	0
4911	4	0.25	1	1	0	0	1	0
4912	6	0.1667	1	1	0	1	0	0
4913	4	0.5	2	1	0	1	0	0
4914	6	0.3333	2	1	0	0	0	0
4915	4	0.25	1	1	0	0	0	0

4916	4	0.25	1	1	0	0	0	0
4917	5	0.2	1	1	0	0	0	0
4918	7	0.1429	1	1	0	1	0	0
4919	2	0.5	1	1	0	0	0	0
4920	7	0.1429	1	1	0	1	1	0
4921	3	0.3333	1	1	0	0	0	0
4922	7	0.1429	1	1	0	1	0	1
4923	7	0.1429	1	1	0	0	0	0
4924	4	0.25	1	1	0	1	0	0
4925	3	0.3333	1	1	0	1	1	0
4926	4	0.25	1	1	0	0	0	0
4927	2	0.5	1	1	0	0	0	0
4928	2	0.5	1	1	0	0	0	0
4929	7	0.1429	1	1	0	1	0	0
4930	2	0.5	1	1	0	0	0	0
4931	3	0.3333	1	1	0	0	0	0
4932	4	0.25	1	1	0	0	0	0
4933	4	0.25	1	1	0	0	0	0
4934	2	0.5	1	1	0	0	0	0
4935	3	0.3333	1	1	0	0	0	0
4936	2	0.5	1	1	0	0	0	0
4937	8	0.125	1	1	0	0	0	0
4938	2	0.5	1	1	0	0	0	0
4939	2	0.5	1	1	0	0	0	0
4940	3	0.3333	1	1	0	0	0	0
4941	4	0.25	1	1	0	0	0	0
4942	2	0.5	1	1	0	0	0	0
4943	5	0.2	1	1	0	0	0	0
4944	2	0.5	1	1	0	0	0	0
4945	2	0.5	1	1	0	1	1	0
4946	4	0.25	1	1	0	1	0	1
4947	7	0.1429	1	1	1	0	0	0
4948	5	0.2	1	1	0	0	0	0
4949	2	0.5	1	1	0	0	0	0
4950	4	0.25	1	1	0	1	0	0
4951	2	0.5	1	1	0	0	0	0
4952	6	0.3333	2	1	0	1	1	0
4953	2	0.5	1	1	0	0	0	0
4954	4	0.25	1	1	0	0	0	0
4955	4	0.25	1	1	0	0	0	0
4956	11	0.0909	1	1	0	0	0	0

4957	4	0.25	1	1	0	1	1	0
4958	4	0.25	1	1	0	0	0	0
4959	3	0.3333	1	1	0	0	0	0
4960	4	0.25	1	1	0	0	0	1
4961	3	0.3333	1	1	0	1	0	0
4962	3	0.3333	1	1	0	1	1	1
4963	5	0.2	1	1	0	0	0	0
4964	2	0.5	1	1	0	0	0	0
4965	7	0.1429	1	1	0	1	0	0
4966	2	0.5	1	1	0	0	0	0
4967	2	0.5	1	1	0	0	0	0
4968	3	0.3333	1	1	0	1	0	0
4969	2	0.5	1	1	0	1	1	0
4970	3	0.3333	1	1	0	0	0	0
4971	10	0.3	3	1	0	0	0	0
4972	2	1	2	1	0	0	0	0
4973	3	0.3333	1	1	0	0	0	0
4974	4	0.25	1	1	0	0	0	0
4975	12	0.1667	2	1	1	0	0	0
4976	4	0.25	1	1	0	1	0	1
4977	5	0.2	1	1	0	0	0	0
4978	6	0.1667	1	1	0	1	1	1
4979	4	0.25	1	1	0	0	1	1
4980	6	0.1667	1	1	0	1	0	1
4981	4	0.25	1	1	0	1	1	1
4982	5	0.2	1	1	0	0	0	1
4983	5	0.2	1	1	0	1	1	1
4984	20	0.05	1	1	0	0	0	1
4985	2	0.5	1	1	0	1	0	1
4986	8	0.25	2	1	0	1	1	1
4987	6	0.1667	1	1	0	0	0	0
4988	4	0.25	1	1	0	0	0	0
4989	3	0.6667	2	1	1	1	0	0
4990	4	0.25	1	1	0	0	0	0
4991	4	0.25	1	1	0	0	0	0
4992	6	0.1667	1	1	0	1	1	1
4993	5	0.2	1	1	0	0	0	0
4994	2	0.5	1	1	0	1	1	0
4995	2	0.5	1	1	0	1	0	0
4996	10	0.3	3	1	0	0	0	0
4997	4	0.25	1	1	0	0	0	0

4998	2	0.5	1	1		1	0	0	0
4999	3	0.3333	1	1		0	0	0	0
5000	5	0.2	1	1		0	0	0	0
5001	9	0.1111	1	1		0	1	0	1
5002	7	0.4286	3	1		0	1	0	1
5003	3	0.3333	1	1		0	0	1	1
5004	2	0.5	1	1		0	0	1	1
5005	3	0.3333	1	1		0	1	0	1
	times	freq		sperm	DM type	colon	HT	HL	anticoag

coronary	DM	PPI	α－bloc	SSRI	treat	DPP4	urethra	Height	Weight
0	1	0	0	0	0		0		
0	1	0	0	0	0		0	183.0	78.0
0	1	0	0	0	0		0		
0	1	0	0	0	0		0	173.0	68.5
0	1	0	0	0	0		0	####	63.2
0	1	0	0	0	0		0		
0	1	0	0	0	0		0		
0	1	0	0	1	0		0	161.0	65.5
0	1	0	0	0	0		0		
0	1	0	0	0	0		0	174.0	83.0
0	1	0	0	1	0		0	170.0	58.0
1	1	0	0	0	0		0		
1	1	0	0	1	0		0	170.0	73.0
0	1	1	0	0	0		0		
0	1	1	0	0	0		0		
0	1	1	0	0	0		0	158.0	50.0
0	1	1	0	0	0		0		
0	1	1	0	0	0		0	147.0	40.0
1	1	1	0	0	0		0	159.0	55.7
0	1	0	0	0	0		0		
0	1	0	0	0	0		0		
0	1	0	0	0	0		0		
0	1	0	0	0	0		0		
0	1	0	0	0	0		0		
1	1	0	0	0	0		0		
1	1	1	1	0	0		0		
0	1	0	0	0	0		0	165.0	63.0
0	1	0	0	0	0		0	####	57.1
0	1	0	0	0	0		0	####	46.0
0	1	0	1	0	0		0	174.0	73.0
0	1	0	0	0	0		0	170.0	
0	1	0	0	0	0		0	####	85.5
0	1	0	0	0	0		0		47.9
1	1	1	0	0	0		0	172.0	57.0
0	2	0	0	0	0		0	170.0	70.0
0	1	1	1	0	1	1	0	####	56.7
0	1	0	0	1	1		0		
0	1	0	1	0	1		0		
0	1	0	1	0	1		0		
0	1	0	0	0	1		0	170.0	66.0
0	1	0	0	0	1		0		

0	1	0	0	0	1		0		
0	1	0	0	0	1		0	168.0	72.0
0	1	0	0	0	1		0	177.0	86.0
0	1	0	1	0	1		0		
0	1	0	1	0	1		0		
0	1	0	0	0	1		0		
0	1	0	0	0	1		0	157.0	58.0
0	1	0	0	0	1		0		
0	1	0	0	0	1		0		
0	1	0	0	0	1		0	178.0	61.6
0	1	0	1	0	1		0	169.0	66.0
0	1	0	0	0	1		0		
0	1	0	0	0	1		0	####	47.4
0	1	0	0	0	1		0	162.0	60.0
0	1	0	0	0	1		0		
0	1	0	0	0	1		0		
0	1	0	0	0	1		0		
0	1	0	0	0	1		0		
0	1	0	0	0	1		0		
0	1	0	0	0	1		0		
0	1	0	0	0	1		0	162.0	64.0
0	1	0	0	0	1		0		
0	1	0	0	0	1		0		
0	1	0	0	0	1		0		
0	1	0	0	0	1		0		
0	1	0	0	0	1		0		
0	1	0	0	0	1		0	168.5	60.0
0	1	0	0	0	1		0	162.0	131.0
0	1	0	0	0	1		0		
0	1	0	0	0	1		0	158.0	55.0
0	1	0	0	0	1		0		
0	1	0	0	0	1		0	166.0	61.0
0	1	0	0	0	1		0	158.0	69.0
0	1	0	0	0	1		0	168.0	73.0
0	1	0	0	0	1		0	161.0	73.0
0	1	0	1	0	1		0	165.0	56.0
0	1	0	0	0	1	1	0	155.0	80.0
0	1	0	1	0	1		0		
0	1	0	1	0	1		0		
0	1	0	0	0	1		0	171.0	66.0
0	1	0	1	0	1		0		
0	1	0	0	0	1		0		
0	1	0	0	0	1		0		

0	1	0	0	0	1	0		
0	1	0	1	0	1	0	160.0	67.0
0	1	0	0	0	1	0		
0	1	0	0	0	1	0		
0	1	0	0	0	1	0	156.0	67.0
0	1	0	0	0	1	0		
0	1	0	0	0	1	0	162.0	68.0
0	1	0	0	0	1	0		
0	1	0	0	0	1	0		
0	1	0	0	0	1	0		
0	1	0	0	0	1	0		
0	1	0	1	0	1	0	175.0	75.0
0	1	0	1	0	1	0	170.0	56.0
0	1	0	0	0	1	0	165.0	68.0
0	1	0	0	0	1	0		
0	1	0	0	1	1	0		
0	1	0	0	0	1	0		
0	1	0	0	0	1	0	165.0	85.0
0	1	0	0	0	1	0	153.0	51.0
0	1	0	0	0	1	0		
0	1	0	0	0	1	0	168.7	48.8
0	1	0	0	0	1	0		
0	1	0	0	0	1	0	166.0	73.0
0	1	0	0	0	1	0	163.0	68.0
0	1	0	1	0	1	0		
0	1	0	0	0	1	0	167.0	70.0
0	1	0	0	0	1	0		
0	1	0	0	0	1	0	166.0	60.0
0	1	0	0	0	1	0		
0	1	0	1	0	1	0		
0	1	0	0	0	1	0	165.0	61.0
0	1	0	0	0	1	0	170.0	71.0
0	1	0	0	0	1	0		
0	1	0	1	0	1	0	159.0	44.0
0	1	0	0	0	1	0		
0	1	0	0	0	1	0	177.0	87.0
0	1	0	1	0	1	0	165.0	54.0
0	1	0	0	0	1	0	160.0	69.0
0	1	0	0	0	1	0		
0	1	0	0	0	1	0		
0	1	0	0	0	1	0		
0	1	0	0	0	1	0		
0	1	0	0	0	1	0		

0	1	0	0	0	1		0		
0	1	0	0	0	1		0	161.0	51.0
0	1	0	0	0	1		0		
0	1	0	0	0	1		0		
0	1	0	0	0	1		0	172.0	65.0
0	1	0	0	0	1		0		
0	1	0	0	0	1		0		
0	1	0	0	0	1		0	175.0	81.0
0	1	0	0	0	1		0		
0	1	0	0	0	1		0		
0	1	0	0	0	1		0	170.0	80.0
0	1	0	0	0	1		0	166.0	68.0
0	1	0	0	0	1		0		
0	1	0	1	0	1		0	####	74.0
0	1	0	0	0	1		0		
0	1	0	0	0	1		0		
0	1	0	1	0	1		0	175.0	53.0
0	1	0	0	0	1		0	163.0	77.7
0	1	0	0	0	1		0	157.0	90.0
0	1	0	0	0	1		0	170.0	72.0
0	1	0	0	0	1		0	165.0	56.0
0	1	0	0	0	1		0		
0	1	0	0	0	1		0		
0	1	0	0	0	1	1	0	166.0	65.0
0	1	0	0	0	1		0		
0	1	0	0	0	1		0	167.0	53.0
0	1	0	0	0	1		0		
0	1	0	0	0	1		0	163.0	49.0
0	1	0	0	0	1		0	174.0	60.0
0	1	0	0	0	1		0	162.0	73.6
0	1	0	0	0	1	1	0	155.1	65.0
0	1	0	0	0	1		0		
0	1	0	0	0	1		0		
0	1	0	0	0	1		0		
0	1	0	0	0	1		0	179.0	83.0
0	1	0	0	0	1		0		
0	1	0	1	0	1		0		
0	1	0	0	0	1		0		
0	1	0	0	0	1		0		
0	1	0	0	0	1		0		
0	1	0	0	0	1		0		
0	1	0	0	0	1		0	####	65.0

0	1	0	0	0	1	0	####	85.0
0	1	0	0	0	1	0	170.0	60.6
0	1	0	0	0	1	0	####	67.0
0	1	0	1	0	1	0	165.0	65.0
0	1	0	0	0	1	0		
0	1	0	0	0	1	0		
0	1	0	1	0	1	0	171.0	82.0
0	1	0	0	0	1	0		
0	1	0	0	0	1	0	161.0	56.0
0	1	0	0	0	1	0		
0	1	0	0	0	1	0		
0	1	0	1	0	1	0	168.0	76.0
0	1	0	0	0	1	0	171.0	57.0
0	1	0	1	1	1	0	167.0	70.0
0	1	0	1	0	1	0	170.0	70.0
0	1	0	1	0	1	0	####	53.0
0	1	0	0	0	1	0		
0	1	0	0	0	1	0	162.0	61.0
0	1	0	0	0	1	0		
0	1	0	0	0	1	0		
0	1	0	0	0	1	0	161.0	50.0
0	1	0	1	0	1	0	170.0	56.0
0	1	0	0	0	1	0	163.0	54.0
0	1	0	0	0	1	0	175.0	85.0
0	1	0	0	0	1	0		
0	1	0	0	0	1	0	####	73.0
0	1	0	1	0	1	0	164.0	68.0
0	1	0	0	0	1	0		
0	1	0	0	0	1	0		
0	1	0	0	0	1	0	####	68.0
0	1	0	0	0	1	0	162.0	62.0
0	1	0	0	0	1	0		
0	1	0	0	0	1	0	####	79.0
0	1	0	1	0	1	0	164.0	75.0
0	1	0	0	0	1	0	165.0	77.0
0	1	0	0	0	1	0	158.0	56.0
0	1	0	0	0	1	0		
0	1	0	0	0	1	0		
0	1	0	0	0	1	0		
0	1	0	0	0	1	0		
0	1	0	0	0	1	0	177.3	67.0
0	1	0	0	0	1	0	165.0	60.0

0	1	0	0	0	1	0		
0	1	0	0	0	1	0	167.0	64.0
0	1	0	0	0	1	0	172.0	75.0
0	1	0	0	0	1	0	167.0	67.0
0	1	0	0	0	1	0		
0	1	0	0	0	1	0		
0	1	0	0	0	1	0		
0	1	0	0	0	1	0	170.0	67.0
0	1	0	0	0	1	0		
0	1	0	0	0	1	0		
0	1	0	0	0	1	0		
0	1	0	0	0	1	0	####	74.0
0	1	0	0	0	1	0	170.0	70.0
0	1	0	0	0	1	0		
0	1	0	0	0	1	0	167.0	71.0
0	1	0	0	0	1	0		
0	1	0	0	0	1	0		
0	1	0	0	0	1	0		
0	1	0	1	0	1	0	158.0	72.0
0	1	0	0	0	1	0		
0	1	0	0	0	1	0		
0	1	0	0	0	1	0		
0	1	0	0	0	1	0		
0	1	0	0	0	1	0		
0	1	0	0	0	1	0		
0	1	0	1	0	1	0	167.0	67.0
0	1	0	0	0	1	0		
0	1	0	0	0	1	0		
0	1	0	0	0	1	0		
0	1	0	0	0	1	0		
0	1	0	0	0	1	0	178.0	96.0
0	1	0	0	0	1	0	163.0	58.0
0	1	0	1	0	1	0	####	53.0
0	1	0	0	0	1	0		
0	1	0	0	0	1	0		
0	1	0	0	0	1	0		
0	1	0	0	0	1	0		
0	1	0	0	0	1	0	165.0	60.0
0	1	0	0	0	1	0		
0	1	0	0	0	1	0		
0	1	0	0	0	1	0		
0	1	0	0	0	1	0	162.0	70.0

0	1	0	0	0	1	0	165.0	72.0
0	1	0	0	0	1	0		
0	1	0	0	0	1	0		
0	1	0	1	0	1	0	####	58.0
0	1	0	1	0	1	0	####	80.0
0	1	0	0	0	1	0	170.0	55.0
0	1	0	0	0	1	0	161.0	59.0
0	1	0	0	0	1	0		
0	1	0	0	0	1	0	171.0	55.0
0	1	0	0	0	1	0	161.0	64.0
0	1	0	0	0	1	0	160.0	83.0
0	1	0	0	0	1	0		
0	1	0	0	0	1	0		
0	1	0	0	0	1	0		
0	1	0	0	0	1	0	169.0	68.0
0	1	0	0	0	1	0		
0	1	0	0	0	1	0	176.0	76.0
0	1	0	1	0	1	0	156.0	52.0
0	1	0	0	0	1	0	170.7	69.0
0	1	0	1	0	1	0	177.0	73.7
0	1	0	0	0	1	0		
0	1	0	0	0	1	0		
0	1	0	0	0	1	0		
0	1	0	0	0	1	0	175.0	88.0
0	1	0	0	0	1	0		
0	1	0	0	0	1	0	166.0	54.6
0	1	0	0	0	1	0	167.0	59.7
0	1	0	0	0	1	0		
0	1	0	0	0	1	0		
0	1	0	0	0	1	0		
0	1	0	0	0	1	0		
0	1	0	0	0	1	0	164.0	50.0
0	1	0	0	0	1	0	166.0	47.0
0	1	0	0	0	1	0		
0	1	0	0	0	1	0		
0	1	0	0	0	1	0	166.0	57.0
0	1	0	0	0	1	0		
0	1	0	0	0	1	0		
0	1	0	0	0	1	0		
0	1	0	1	0	1	0		
0	1	0	1	0	1	0	165.0	67.0
0	1	0	0	0	1	0	161.0	61.0

0	1	0	0	0	1	0	161.0	53.0
0	1	0	0	0	1	0		
0	1	0	0	0	1	0	173.0	72.0
0	1	0	1	0	1	0	130.0	62.0
0	1	0	0	0	1	0		
0	1	0	0	0	1	0		
0	1	0	0	0	1	0		
0	1	0	0	0	1	0		
0	1	0	1	0	1	0		
0	1	0	0	0	1	0		
0	1	0	1	0	1	0		
0	1	0	0	0	1	0	####	71.5
0	1	0	0	0	1	0	167.0	57.0
0	1	0	0	0	1	0		
0	1	0	0	0	1	0	169.5	61.0
0	1	0	0	0	1	0		
0	1	0	0	0	1	0	163.0	48.0
0	1	0	0	0	1	0		
0	1	0	0	0	1	0	177.0	108.0
0	1	0	0	0	1	0	153.0	70.0
0	1	0	0	0	1	0		
0	1	0	0	0	1	0		
0	1	0	0	0	1	0		
0	1	0	0	0	1	0	162.2	56.0
0	1	0	0	0	1	0		
0	1	0	0	0	1	0		
0	1	0	0	0	1	0	164.0	68.0
0	1	0	0	0	1	0	180.0	78.0
0	1	0	0	0	1	0		
0	1	0	0	0	1	0		
0	1	0	0	0	1	0		
0	1	0	0	0	1	0	####	63.0
0	1	0	0	0	1	0		
0	1	0	0	0	1	0		
0	1	0	0	0	1	0		
0	1	0	0	0	1	0		
0	1	0	0	0	1	0	173.0	124.0
0	1	0	0	0	1	0		
0	1	0	0	0	1	0	172.0	74.0
0	1	0	0	0	1	0		
0	1	0	0	0	1	0		
0	1	0	0	0	1	0		

0	1	0	0	0	1	0	167.0	94.0
0	1	0	0	0	1	0		
0	1	0	0	0	1	0		
0	1	0	1	0	1	0		
0	1	0	0	0	1	0		
0	1	0	0	0	1	0	####	92.0
0	1	0	0	0	1	0	173.0	89.0
0	1	0	0	0	1	0		
0	1	0	0	0	1	0	170.0	74.0
0	1	0	0	0	1	0		
0	1	0	0	0	1	0	160.0	56.7
0	1	0	0	0	1	0		
0	1	0	0	0	1	0	175.0	77.0
0	1	0	0	0	1	0	177.0	88.0
0	1	0	0	0	1	0		
0	1	0	0	0	1	0	161.0	51.0
0	1	0	1	0	1	0	172.0	72.0
0	1	0	0	0	1	0		
0	1	0	0	0	1	0	167.7	74.5
0	1	0	0	0	1	0		
0	1	0	1	0	1	0		
0	1	0	0	0	1	0	175.0	75.0
0	1	0	0	0	1	0		
0	1	0	0	0	1	0		
0	1	0	1	0	1	0	173.0	73.0
0	1	0	0	0	1	0	170.0	76.0
0	1	0	0	0	1	0		
0	1	0	1	0	1	0	162.0	64.4
0	1	0	0	0	1	0	189.0	124.0
0	1	0	0	0	1	0		
0	1	0	0	0	1	0		
0	1	0	0	0	1	0		
0	1	0	0	0	1	0	179.0	80.0
0	1	0	0	0	1	0		
0	1	0	1	0	1	0		
0	1	0	0	0	1	0		
0	1	0	0	0	1	0		
0	1	0	0	0	1	0		
0	1	0	0	0	1	0		
0	1	0	0	0	1	0		
0	1	0	1	0	1	0		
0	1	0	0	0	1	0		

0	1	0	1	0	1	0		
0	1	0	0	0	1	0		
0	1	0	0	0	1	0		
0	1	0	1	0	1	0	169.0	77.0
0	1	0	0	0	1	0		
0	1	0	0	0	1	0		
0	1	0	0	0	1	0		
0	1	0	0	0	1	0		
0	1	0	0	0	1	0		
0	1	0	0	0	1	0		
0	1	0	0	0	1	0		
0	1	0	0	0	1	0		
0	1	0	0	0	1	0		
0	1	0	0	0	1	0		
0	1	0	0	0	1	0		
0	1	0	0	0	1	0		
0	1	0	0	0	1	0	164.0	66.0
0	1	0	0	0	1	0		
0	1	0	0	0	1	0		
0	1	0	0	0	1	0	167.0	55.4
0	1	0	0	0	1	0	165.0	73.0
0	1	0	0	0	1	0		
0	1	0	0	0	1	0		
0	1	0	1	0	1	0		
0	1	0	0	0	1	0		
0	1	0	0	0	1	0		
0	1	0	0	0	1	0		
0	1	0	1	0	1	0		
0	1	0	0	0	1	0	169.0	68.0
0	1	0	1	0	1	0		
0	1	0	0	0	1	0		
0	1	0	0	0	1	0	163.6	44.6
0	1	0	0	0	1	0		
0	1	0	0	0	1	0	163.0	55.0
0	1	0	0	0	1	0		
0	1	0	0	0	1	0		
0	1	0	0	0	1	0		
0	1	0	0	0	1	0	172.0	97.0
0	1	0	0	0	1	0	160.0	42.0
0	1	0	0	0	1	0		
0	1	0	0	0	1	0		
0	1	0	0	0	1	0		
0	1	0	0	0	1	0		

0	1	0	0	0	1	0		
0	1	0	0	0	1	0	166.0	63.0
0	1	0	0	0	1	0		
0	1	0	0	0	1	0		
0	1	0	1	0	1	0		
0	1	0	1	0	1	0	####	76.0
0	1	0	0	0	1	0		
0	1	0	1	0	1	0	173.0	66.0
0	1	0	0	0	1	0		
0	1	0	0	0	1	0		
0	1	0	0	0	1	0		
0	1	0	0	0	1	0	171.0	93.0
0	1	0	0	0	1	0		
0	1	0	0	0	1	0	172.0	74.0
0	1	0	0	0	1	0		
0	1	0	0	0	1	0		
0	1	0	0	0	1	0		
0	1	0	0	0	1	0	165.0	79.0
0	1	0	0	0	1	0	161.0	63.0
0	1	0	0	0	1	0		
0	1	0	0	0	1	0		
0	1	0	0	0	1	0		
0	1	0	1	0	1	0	####	73.0
0	1	0	1	0	1	0		
0	1	0	1	0	1	0	158.0	62.0
0	1	0	0	0	1	0		
0	1	0	1	0	1	0	170.0	77.0
0	1	0	1	0	1	0	####	61.0
0	1	0	0	0	1	0		
0	1	0	0	0	1	0	169.0	65.0
0	1	0	0	0	1	0	####	70.0
0	1	0	0	0	1	0	163.0	46.0
0	1	0	0	0	1	0	150.0	46.0
0	1	0	0	1	1	0	170.0	75.0
0	1	0	0	0	1	0		
0	1	0	0	0	1	0		
0	1	0	0	0	1	0		55.5
0	1	0	0	0	1	0	172.0	106.0
0	1	0	0	0	1	0		
0	1	0	0	0	1	0		
0	1	0	0	0	1	0		
0	1	0	0	0	1	0	174.0	75.0

0	1	0	0	0	1		0		
0	1	0	1	0	1		0		
0	1	0	0	0	1		0		
0	1	0	0	0	1		0		
0	1	0	1	0	1		0	171.0	67.0
0	1	0	0	0	1		0	178.5	72.1
0	1	0	1	0	1		0	173.0	74.0
0	1	0	0	0	1	0	0	173.0	71.0
0	1	0	0	0	1		0	164.0	70.0
0	1	0	0	0	1		0		
0	1	0	0	0	1		0	175.0	90.0
0	1	0	0	0	1		0		
0	1	0	0	0	1		0	164.0	53.0
0	1	0	0	0	1		0		
0	1	0	0	0	1		0	160.0	53.3
0	1	0	0	0	1		0	176.6	72.2
0	1	0	0	0	1		0	168.0	52.0
0	1	0	0	0	1		0	167.0	
0	1	0	0	0	1		0		
0	1	0	0	0	1		0		
0	1	0	0	0	1		0		
0	1	0	0	0	1		0		
0	1	0	0	0	1		0		
0	1	0	0	0	1		0	164.0	74.6
0	1	0	0	0	1		0	173.0	67.0
0	1	0	0	0	1		0	167.0	60.0
0	1	0	0	0	1		0		
0	1	0	1	0	1		0		
0	1	0	0	0	1		0		
0	1	0	0	0	1		0		
0	1	0	0	0	1		0	182.7	106.0
0	1	0	1	0	1		0		
0	1	0	0	0	1		0		
0	1	0	0	0	1		0	179.0	130.0
0	1	0	0	0	1		0	174.0	77.0
0	1	0	0	0	1		0	175.0	56.0
0	1	0	0	0	1		0		
0	1	0	1	0	1		0	158.0	54.0
0	1	0	0	0	1		0	####	92.5
0	1	0	0	0	1		0		
0	1	0	0	0	1		0	175.0	106.0
0	1	0	1	0	1		0		

0	1	0	0	0	1		0	####	54.2
0	1	0	1	0	1		0		
0	1	0	1	0	1		0		
0	1	0	0	0	1		0	####	58.0
0	1	0	0	0	1		0	####	83.8
0	1	0	0	0	1		0	####	90.0
0	1	0	1	0	1		0	####	65.0
0	1	0	0	0	1		0	####	55.0
0	1	0	1	0	1		0		
0	1	0	0	0	1		0	170.0	58.0
0	1	0	0	0	1		0		
0	1	0	0	0	1		0	171.0	100.0
0	1	0	0	0	1		0	163.5	68.0
0	1	0	0	0	1		0		
0	1	0	0	0	1		0	167.5	67.0
0	1	0	1	0	1		0		
0	1	0	0	0	1	0	0	174.0	82.0
0	1	0	0	0	1		0		
0	1	0	0	0	1		0		
0	1	0	0	0	1		0		
0	1	0	1	0	1		0		
0	1	0	0	0	1		0	168.0	69.0
0	1	0	0	0	1		0		
0	1	0	0	0	1		1	####	64.3
0	1	0	0	0	1		1		
0	1	0	0	0	1		1	171.0	64.0
0	1	0	0	0	1		1	164.0	60.0
0	1	0	0	0	1		1	167.0	65.0
0	1	0	0	0	1		1		
0	1	0	0	0	1		1		
0	1	0	0	0	1		1	####	66.0
0	1	0	1	0	1		1		
1	1	0	0	0	1		0		
1	1	0	0	0	1		0	160.0	55.0
1	1	0	0	0	1		0	####	63.0
1	1	0	0	0	1		0	####	52.0
1	1	0	0	0	1		0	164.0	56.0
1	1	0	0	0	1		0	169.0	50.0
1	1	0	1	0	1		0		
1	1	0	1	0	1		0	161.0	62.0
1	1	0	0	0	1		0	167.0	66.0

1	1	0	0	0	1	0	176.0	80.0
1	1	0	0	0	1	0		
1	1	0	0	0	1	0	167.0	51.0
1	1	0	0	0	1	0	163.0	57.0
1	1	0	0	0	1	0		
1	1	0	0	0	1	0	176.0	86.0
1	1	0	1	0	1	0	171.0	56.0
1	1	0	0	0	1	0		
1	1	0	0	0	1	0	174.0	58.0
1	1	0	1	0	1	0	155.0	52.0
1	1	0	0	0	1	0	172.0	94.0
1	1	0	0	0	1	0	160.0	53.0
1	1	0	1	0	1	0	166.0	66.0
1	1	0	0	0	1	0	####	64.0
1	1	0	0	0	1	0	####	74.0
1	1	0	0	0	1	0	172.0	56.8
1	1	0	0	0	1	0	161.0	78.0
1	1	0	1	0	1	0	####	78.0
1	1	0	0	0	1	0	170.0	74.0
1	1	0	0	0	1	0	168.0	65.0
1	1	0	0	0	1	0	160.0	55.0
1	1	0	0	0	1	0	164.0	45.0
1	1	0	0	0	1	0		
1	1	0	0	0	1	0	168.0	69.0
1	1	0	0	0	1	0		
1	1	0	0	0	1	0	170.0	63.0
1	1	0	0	0	1	0		
1	1	0	0	0	1	0	171.0	56.0
1	1	0	0	0	1	0		
1	1	0	0	0	1	0	163.0	66.0
1	1	0	0	0	1	0	167.0	60.0
1	1	0	1	1	1	0		
1	1	0	1	0	1	0	####	61.0
1	1	0	0	0	1	0	166.0	72.0
1	1	0	0	0	1	0	####	80.0
1	1	0	1	0	1	0	161.0	64.0
1	1	0	0	0	1	0	165.0	74.0
1	1	0	1	0	1	0	171.0	74.0
1	1	0	1	0	1	0		
1	1	0	0	0	1	0		
1	1	0	1	0	1	0	172.0	75.0
1	1	0	1	0	1	0	165.5	70.0

1	1	0	0	0	1	0	163.0	53.5
1	1	0	0	0	1	0	172.5	76.6
1	1	0	0	0	1	0		
1	1	0	0	0	1	0		
1	1	0	1	0	1	0		
1	1	0	0	0	1	0	####	72.8
1	1	0	0	0	1	0	171.0	63.0
1	1	0	0	0	1	0	163.0	55.0
1	1	0	1	0	1	0		
1	1	0	0	0	1	0	169.0	63.0
1	1	0	0	0	1	0		
1	1	0	1	0	1	0		
1	1	0	0	0	1	0	173.0	71.3
1	1	0	0	0	1	0	165.0	78.0
1	1	0	0	0	1	0	165.0	63.0
1	1	0	0	0	1	0	155.0	62.0
1	1	0	0	0	1	0	165.0	79.0
1	1	0	0	0	1	0	166.0	67.0
1	1	0	1	0	1	0		
1	1	0	0	0	1	0		
1	1	0	0	0	1	0	166.7	51.0
1	1	0	0	0	1	0	168.0	65.0
1	1	0	0	0	1	0	172.0	77.0
1	1	0	0	0	1	0	166.0	81.1
1	1	0	0	0	1	0		
1	1	0	0	0	1	0	162.0	76.0
1	1	0	0	0	1	0	167.0	68.0
1	1	0	0	0	1	0		
1	1	0	0	0	1	0		
1	1	0	0	0	1	0	176.0	73.0
1	1	0	0	0	1	0	165.0	71.0
1	1	0	0	0	1	0	170.0	64.3
1	1	0	0	0	1	0		
1	1	0	1	0	1	0	153.0	42.0
1	1	0	0	0	1	0	157.0	55.0
1	1	0	0	0	1	0		
1	1	0	0	0	1	0	163.0	78.0
1	1	0	0	0	1	0	174.0	82.5
1	1	0	0	0	1	0	176.0	73.0
1	1	0	0	0	1	0		
1	1	0	0	0	1	1		
1	1	0	0	0	1	1	168.0	71.0
0	1	1	0	0	1	0		

0	1	1	1	0	1	0		
0	1	1	0	0	1	0		
0	1	1	0	0	1	0		
0	1	1	1	0	1	0	151.0	50.0
0	1	1	0	0	1	0		
0	1	1	0	0	1	0		
0	1	1	1	0	1	0	####	50.0
0	1	1	1	0	1	0	174.0	76.0
0	1	1	0	0	1	0	159.3	57.9
0	1	1	0	0	1	0	164.5	71.0
0	1	1	0	0	1	0	162.0	56.0
0	1	1	0	0	1	0	166.0	66.0
0	1	1	1	0	1	0	173.0	67.0
0	1	1	0	0	1	0	158.0	61.0
0	1	1	0	0	1	0	168.0	66.0
0	1	1	0	0	1	0	181.0	74.6
0	1	1	1	0	1	0		
0	1	1	1	0	1	0	164.0	55.0
0	1	1	0	0	1	0	163.0	73.0
0	1	1	0	0	1	0		
0	1	1	0	0	1	0		
0	1	1	0	0	1	0	173.0	89.0
0	1	1	0	0	1	0		
0	1	1	0	0	1	0	####	62.0
0	1	1	1	0	1	0	160.0	73.0
0	1	1	0	0	1	0		
0	1	1	0	0	1	0	161.0	70.0
0	1	1	1	0	1	0	172.0	72.0
0	1	1	0	0	1	0	163.0	74.0
0	1	1	0	1	1	0	164.0	63.0
0	1	1	0	0	1	0		
0	1	1	0	0	1	0		
0	1	1	1	0	1	0	161.0	67.0
0	1	1	1	0	1	0	162.0	54.0
0	1	1	0	0	1	0		
0	1	1	0	0	1	0	169.0	54.0
0	1	1	0	0	1	0	163.0	67.0
0	1	1	0	0	1	0		
0	1	1	0	0	1	0		
0	1	1	1	0	1	0		
0	1	1	0	0	1	0	####	65.0
0	1	1	0	0	1	0		

0	1	1	0	0	1	0		
0	1	1	0	0	1	0	173.0	72.0
0	1	1	0	0	1	0		
0	1	1	0	0	1	0	167.0	57.0
0	1	1	0	0	1	0	162.0	62.0
0	1	1	0	0	1	0		
0	1	1	0	0	1	0		
0	1	1	0	0	1	0	166.0	53.0
0	1	1	0	0	1	0	160.0	60.0
0	1	1	1	0	1	0	159.0	59.0
0	1	1	1	0	1	0	####	64.0
0	1	1	0	0	1	0		
0	1	1	0	0	1	0	166.8	69.0
0	1	1	0	0	1	0		
0	1	1	0	0	1	0		
0	1	1	0	0	1	0	167.0	58.0
0	1	1	0	0	1	0	177.0	63.0
0	1	1	0	0	1	0	168.0	61.0
0	1	1	0	0	1	0		
0	1	1	0	0	1	0	170.0	83.0
0	1	1	0	0	1	0		
0	1	1	0	0	1	0		
0	1	1	0	0	1	0		
0	1	1	1	0	1	0		
0	1	1	0	0	1	0		
0	1	1	0	0	1	0		
0	1	1	0	0	1	0		
0	1	1	1	0	1	0	169.0	65.5
0	1	1	0	0	1	0	####	51.0
0	1	1	0	0	1	0		
0	1	1	1	0	1	0	162.0	50.7
0	1	1	0	0	1	0		
0	1	1	0	0	1	0	154.0	52.1
0	1	1	0	0	1	0	163.0	64.0
0	1	1	0	0	1	0		
0	1	1	0	0	1	0		
0	1	1	0	0	1	0		
0	1	1	0	0	1	0		
0	1	1	0	0	1	0		
0	1	1	0	0	1	0	####	61.0
0	1	1	1	0	1	0	159.0	66.0
0	1	1	0	0	1	0		

0	1	1	0	0	1		0		
0	1	1	0	1	1		0		
0	1	1	0	0	1		0	166.0	56.0
0	1	1	0	0	1		0	167.0	65.0
0	1	1	0	0	1		0	165.0	52.2
0	1	1	0	0	1		0		
0	1	1	0	0	1		0		
0	1	1	0	0	1		0	161.0	64.0
0	1	1	1	0	1		0		
0	1	1	0	0	1		0	158.0	55.0
0	1	1	0	0	1		0	162.0	72.0
0	1	1	0	0	1	1	0	160.0	55.0
0	1	1	0	1	1		0	163.0	76.0
0	1	1	0	0	1		0		85.0
0	1	1	1	0	1		1	####	64.0
0	1	1	1	0	1		1		
1	1	1	0	0	1		0		
1	1	1	1	0	1		0	167.0	62.0
1	1	1	1	0	1		0	165.0	60.0
1	1	1	1	0	1		0	####	81.0
1	1	1	0	0	1		0	166.0	72.0
1	1	1	0	0	1		0	160.0	52.0
1	1	1	0	0	1		0	165.0	54.0
1	1	1	0	0	1		0		
1	1	1	0	0	1		0	175.0	54.0
1	1	1	1	0	1		0	162.0	56.0
1	1	1	1	0	1		0	161.0	47.0
1	1	1	0	0	1		0	166.0	52.0
1	1	1	0	0	1		0		
1	1	1	1	0	1		0	159.0	65.0
1	1	1	1	0	1		0		58.0
1	1	1	0	0	1		0	158.0	78.0
1	1	1	1	0	1		0	167.0	56.0
1	1	1	1	0	1		0		
1	1	1	1	0	1		0	155.0	57.0
1	1	1	0	0	1		0	170.0	78.0
1	1	1	1	0	1		0		
1	1	1	0	0	1		0		
1	1	1	0	0	1		0	162.0	72.0
1	1	1	1	0	1		0	170.0	77.0
1	1	1	0	0	1		0	168.0	72.0
1	1	1	0	0	1		0	167.0	67.0

1	1	1	0	0	1	0	163.0	59.0
1	1	1	0	0	1	0		
1	1	1	0	0	1	0	173.0	77.7
1	1	1	0	0	1	0		
1	1	1	0	0	1	0	156.0	58.0
1	1	1	1	0	1	0	167.0	59.0
1	1	1	0	0	1	0		
1	1	1	0	0	1	0	175.0	77.0
1	1	1	0	0	1	0	162.0	62.0
1	1	1	0	0	1	0	####	61.3
1	1	1	1	0	1	0	159.0	52.0
1	1	1	0	0	1	0		
1	1	1	0	0	1	0	168.0	66.0
1	1	1	1	0	1	0	162.0	59.0
1	1	1	0	0	1	0		
1	1	1	1	0	1	0	165.0	54.4
1	1	1	0	0	1	0	166.0	72.0
1	1	1	0	0	1	0		
1	1	1	0	0	1	0		
1	1	1	0	0	1	0	180.0	99.5
1	1	1	0	0	1	0		88.0
1	1	1	0	0	1	0		
1	1	1	0	0	1	0	160.0	53.0
1	1	1	0	0	1	0		
1	1	1	0	0	1	0		
1	1	1	0	0	1	0	157.0	65.0
1	1	1	0	0	1	0	167.0	72.0
1	1	1	0	0	1	0		
1	1	1	1	0	1	0		
1	1	1	0	1	1	0	167.0	63.0
1	1	1	0	0	1	0		
1	1	1	0	0	1	0	####	82.5
1	1	1	1	0	1	0		
1	1	1	0	0	1	0	173.0	83.0
1	1	1	0	0	1	1	162.0	54.0
0	1	0	1	0	1	0		
0	1	0	0	0	1	0	####	88.0
0	1	0	1	0	1	0	165.0	56.0
0	1	0	0	0	1	0		
0	1	0	0	1	1	0		
0	1	0	0	0	1	0		
0	1	0	0	0	1	0	150.0	55.0

0	1	0	0	0	1	0	144.0	44.6
0	1	0	0	0	1	0	169.0	57.0
0	1	0	1	0	1	0		
0	1	0	0	0	1	0		
0	1	0	0	0	1	0		
0	1	0	0	0	1	0		
0	1	0	0	0	1	0		
0	1	0	0	0	1	0		
0	1	0	0	1	1	0	####	65.0
0	1	0	1	0	1	0	####	65.0
0	1	0	0	0	1	0	160.0	57.0
0	1	0	1	0	1	0	165.0	56.0
0	1	0	0	0	1	0		
0	1	0	0	0	1	0		
0	1	0	0	0	1	0		
0	1	0	0	0	1	0		
0	1	0	0	0	1	0		
0	1	0	0	0	1	0		
0	1	0	0	0	1	0		
0	1	0	0	0	1	0		
0	1	0	0	0	1	0		
0	1	0	0	0	1	0		
0	1	0	0	0	1	0		
0	1	0	0	0	1	0		
0	1	0	0	0	1	0		
0	1	0	0	0	1	0		
0	1	0	0	0	1	0		
0	1	0	0	0	1	0		
0	1	0	0	0	1	0		
0	1	0	0	0	1	0		
0	1	0	1	0	1	0		
0	1	0	0	0	1	0	167.0	91.0
0	1	0	0	0	1	0		
0	1	0	0	1	1	0		
0	1	0	0	0	1	0		
0	1	0	0	0	1	0		
0	1	0	0	0	1	0		
0	1	0	0	0	1	0		
0	1	0	0	0	1	0		
0	1	0	1	0	1	0	157.0	45.0
0	1	0	1	0	1	0		

0	1	0	0	0	1	0		
0	1	0	1	0	1	0	####	65.0
0	1	0	0	0	1	0	####	50.0
0	1	0	0	0	1	0		
0	1	0	0	0	1	0	####	55.0
0	1	0	0	0	1	0		
0	1	0	0	0	1	0		
0	1	0	0	0	1	0		
0	1	0	0	0	1	0	174.0	62.0
0	1	0	0	0	1	0		
0	1	0	0	0	1	0		
0	1	0	0	0	1	0	166.0	86.0
0	1	0	0	0	1	0	166.0	52.0
0	1	0	0	0	1	0		
0	1	0	0	0	1	0	173.0	69.0
0	1	0	0	0	1	0		
0	1	0	0	0	1	0		
0	1	0	1	0	1	0		
0	1	0	0	0	1	0		
0	1	0	0	0	1	0		
0	1	0	0	0	1	0		
0	1	0	0	0	1	0		
0	1	0	0	0	1	0		
0	1	0	0	0	1	0		
0	1	0	0	0	1	0		
0	1	0	0	0	1	0	165.6	59.1
0	1	0	0	0	1	0		
0	1	0	0	0	1	0	171.0	64.0
0	1	0	0	0	1	0		
0	1	0	0	0	1	0		
0	1	0	1	0	1	0		
0	1	0	0	0	1	0		
0	1	0	0	0	1	0		
0	1	0	0	0	1	0	178.0	76.0
0	1	0	1	0	1	0		
0	1	0	0	0	1	0	168.0	65.0
0	1	0	1	0	1	0	161.0	63.0
0	1	0	0	0	1	0		
0	1	0	0	0	1	0		
0	1	0	1	0	1	0	172.0	67.0
0	1	0	0	0	1	0		
0	1	0	0	0	1	0		

0	1	0	1	0	1	0	170.0	70.0
0	1	0	0	0	1	0	####	48.0
0	1	0	0	0	1	0		
0	1	0	0	1	1	0		
0	1	0	0	0	1	0	182.0	73.4
0	1	0	0	0	1	0	170.0	70.0
0	1	0	0	0	1	0	175.0	82.0
0	1	0	0	0	1	0		
0	1	0	0	0	1	0		
0	1	0	0	0	1	0		
0	1	0	0	0	1	0	172.0	69.5
0	1	0	0	0	1	0	####	75.0
0	1	0	0	0	1	0		
0	1	0	0	0	1	0		
0	1	0	0	0	1	0		
0	1	0	0	0	1	0		
0	1	0	0	1	1	0	162.0	98.0
0	1	0	0	0	1	0		
0	1	0	0	0	1	0		63.0
0	1	0	0	0	1	1	161.0	58.0
1	1	0	1	0	1	0		
1	1	0	1	0	1	0		
1	1	0	0	0	1	0		
1	1	0	1	0	1	0		
1	1	0	0	0	1	0	163.0	66.0
1	1	0	0	0	1	0		
1	1	0	0	0	1	0	167.0	60.0
1	1	0	0	1	1	0		
1	1	0	0	0	1	0	165.0	70.0
1	1	0	0	0	1	0	168.0	67.0
1	1	0	0	0	1	0		
1	1	0	0	0	1	0	161.0	62.0
1	1	0	0	0	1	0	166.0	76.0
1	1	0	0	0	1	0	166.0	53.0
1	1	0	1	0	1	0		
1	1	0	0	0	1	0		
1	1	0	0	0	1	0	####	47.6
1	1	0	0	0	1	0	171.0	66.0
1	1	0	0	0	1	0		
1	1	0	0	0	1	0	165.0	80.0
1	1	0	0	0	1	1	171.0	70.0
1	1	0	0	0	1	1	158.0	69.0

0	1	1	1	0	1	0	176.0	71.0
0	1	1	0	0	1	0	160.0	66.0
0	1	1	0	0	1	0		
0	1	1	1	0	1	0		
0	1	1	0	0	1	0	166.0	68.0
0	1	1	0	0	1	0		
0	1	1	1	0	1	0	159.0	65.0
0	1	1	0	0	1	0		
0	1	1	0	0	1	0	178.0	60.0
0	1	1	0	0	1	0		
0	1	1	0	0	1	0		
0	1	1	0	0	1	0		
0	1	1	0	0	1	0	172.0	90.0
0	1	1	0	0	1	0		
0	1	1	0	0	1	0		
0	1	1	0	0	1	0	170.0	70.0
0	1	1	0	0	1	0	####	62.0
0	1	1	0	0	1	0		
0	1	1	1	0	1	0		
0	1	1	0	0	1	0		
0	1	1	0	0	1	0	175.0	65.0
0	1	1	0	0	1	0		
0	1	1	0	0	1	0	####	####
0	1	1	0	0	1	0		
1	1	1	0	0	1	0		
1	1	1	0	0	1	0	170.0	79.0
1	1	1	0	0	1	0	154.0	50.0
1	1	1	0	0	1	0		
1	1	1	1	0	1	0	168.0	90.0
1	1	1	0	0	1	0	158.0	65.0
1	1	1	1	0	1	0	173.0	69.0
1	1	1	0	0	1	0		
1	1	1	0	0	1	0		
1	1	1	0	0	1	0		
1	1	1	1	0	1	0		
1	1	1	0	0	1	0		
1	1	1	0	0	1	0	164.0	62.0
1	1	1	0	0	1	0	####	59.0
1	1	1	0	0	1	0	170.0	48.0
1	1	1	0	0	1	0	163.0	61.0
1	1	1	0	0	1	0	158.0	62.3

1	1	1	0	0	1		0	####	66.0
1	1	1	1	0	1		0	####	48.6
1	1	1	0	0	1		0		
1	1	1	0	0	1		0	168.0	57.0
1	1	1	0	0	1		0	####	54.0
1	1	1	1	0	1		0		
1	1	1	0	0	1		0	175.0	64.0
0	1	0	0	1	1	0	0	173.0	87.0
0	1	0	0	0	1	1	0		
0	1	0	0	0	1	1	0		
0	1	0	0	0	1	0	0	184.0	76.0
0	1	0	0	0	1	0	0	177.0	82.0
0	1	0	0	0	1		0	169.0	61.0
0	1	0	0	0	1	1	0	170.0	63.0
0	1	0	0	0	1	1	0	####	54.0
0	1	0	1	0	1		0	####	72.0
0	1	0	0	0	1		0	173.5	70.0
0	1	0	0	0	1		0	####	70.0
0	1	0	1	0	1	0	0	####	65.0
0	1	0	0	0	1		0		
0	1	0	0	0	1	1	0	156.5	65.0
0	1	0	0	0	1	0	0	171.0	67.0
0	1	0	0	0	1		0	####	56.0
0	1	0	0	0	1	1	0	####	74.0
0	1	0	0	0	1	0	0	165.0	84.0
0	1	0	0	0	1		0	####	61.0
0	1	0	0	0	1	1	0	173.0	57.0
0	1	0	0	0	1		0	168.0	68.0
0	1	0	0	0	1	1	0	158.7	60.0
0	1	0	0	0	1	1	0	165.0	61.0
0	1	0	0	0	1		0	####	####
0	1	0	0	0	1	0	0	170.0	74.0
0	1	0	0	0	1	1	0		
0	1	0	0	0	1	0	0	172.0	57.0
0	1	0	0	0	1	0	0	177.0	72.0
0	1	0	0	0	1	1	0	169.0	92.0
0	1	0	0	0	1	1	0		
0	1	0	0	0	1		0		
0	1	0	0	0	1	1	0		71.7
0	1	0	1	0	1	1	0		
0	1	0	0	0	1	1	0		

0	1	0	0	0	1		0		
0	1	0	0	0	1	0	0		
0	1	0	0	1	1	0	0	177.5	81.0
0	1	0	0	0	1		0	169.0	53.0
1	1	0	0	0	1	0	0	170.0	73.2
1	1	0	0	0	1	1	0	####	83.0
1	1	0	0	0	1		0	176.0	64.0
1	1	0	1	0	1		0	175.0	76.0
1	1	0	0	0	1	0	0		
1	1	0	0	0	1	1	0	164.0	73.0
0	1	1	0	0	1	0	0	170.0	60.0
0	1	1	0	1	1	0	0	163.0	
0	1	1	1	0	1	0	0	161.0	53.0
0	1	1	0	0	1	0	0	####	56.0
0	1	1	1	0	1	1	0	163.0	50.0
0	1	1	0	0	1	1	0	####	69.5
0	1	1	0	1	1	0	0	166.0	51.0
0	1	1	1	0	1	0	0	####	58.0
0	1	1	0	0	1		0	####	80.0
0	1	1	1	0	1		0	162.0	65.0
0	1	1	0	0	1	0	0		58.0
1	1	1	0	0	1		0	165.0	57.0
1	1	1	1	0	1	1	0	155.0	50.0
1	1	1	0	0	1		0	####	59.0
1	1	1	0	1	1	1	0	167.0	67.0
1	1	1	0	0	1	1	0	168.0	79.0
1	1	1	0	1	1	1	0	166.0	50.0
0	1	0	0	0	1		0		
0	1	0	1	0	1		0		
0	1	0	1	0	1		0		
0	1	0	1	0	1		0	####	67.0
0	1	0	0	0	1		0	####	58.0
0	1	0	0	0	1		0	152.0	42.0
0	1	0	0	0	2		0		
0	1	0	0	0	2		0	179.0	66.0
0	1	0	1	0	2		0		
0	1	0	0	0	2	0	0	174.0	60.0
0	1	0	1	0	2		0	163.0	63.0
0	1	0	0	0	2		0		
0	1	0	0	0	2		0	185.0	72.3
0	1	0	0	0	2		0	167.0	50.0
0	1	0	0	0	2		0	167.5	98.8

0	1	0	0	0	2		0	167.0	61.0
0	1	0	0	0	2		0	177.0	78.0
0	1	0	0	0	2		0	####	84.0
0	1	0	0	0	2		0	177.0	65.0
0	1	0	0	0	2		0	174.0	60.0
0	1	0	0	0	2		0	171.0	62.0
0	1	0	0	0	2		0	155.0	40.0
0	1	0	0	0	2		0	169.0	61.6
0	1	0	0	0	2		0	168.0	60.0
0	1	0	0	0	2		0	170.0	77.0
0	1	0	0	0	2	0	0	160.0	55.0
1	1	0	0	0	2		0	164.0	65.0
1	1	0	0	0	2		0	####	58.0
1	1	0	0	0	2		0	159.0	37.0
1	1	0	0	0	2		0	176.0	72.0
1	1	0	1	0	2		0	####	85.0
1	1	0	0	0	2		0	173.0	69.0
1	1	0	0	0	2		0	182.0	65.0
0	1	1	0	0	2		0	####	55.0
0	1	1	0	1	2		0	164.0	54.0
0	1	1	0	0	2		0	156.0	50.0
0	1	1	0	0	2		0	168.0	66.6
1	1	1	0	0	2		0	175.0	69.0
1	1	1	0	0	2		0	174.0	96.0
0	1	0	0	0	2		0		
0	1	0	0	0	2		0	164.0	56.0
0	1	0	0	0	2	0	0	170.0	65.0
0	1	0	0	0	2		0	180.0	67.0
0	1	0	0	0	2		0	173.0	66.0
1	1	0	0	0	2		0	163.0	57.0
0	1	0	0	0	2	1	0	163.0	61.2
0	1	0	0	0	2		0	160.0	44.0
0	1	0	0	0	2		0	167.0	47.0
0	1	0	0	0	2		0		
0	1	0	0	0	2		0	160.0	53.4
0	1	0	0	0	2		0	176.0	75.0
0	1	0	0	0	2		0		
0	1	0	0	0	2		0		
0	1	0	0	0	2		0		
0	1	0	0	0	2		0	168.0	57.0
0	1	0	0	0	2		0		61.0
0	1	0	0	0	2		0	167.0	62.0

0	1	0	0	0	2	0		
0	1	0	0	0	2	0	173.5	44.0
0	1	0	0	0	2	0		
0	1	0	1	0	2	0	####	61.0
0	1	0	0	0	2	0	163.0	76.0
0	1	0	0	0	2	0	169.0	72.0
0	1	0	0	0	2	0		
0	1	0	1	0	2	0	168.0	62.0
0	1	0	0	0	2	0	161.0	72.0
0	1	0	0	0	2	0	158.0	66.0
0	1	0	0	0	2	0	175.0	90.0
0	1	0	0	0	2	0	170.0	86.0
0	1	0	0	0	2	0	158.0	63.0
0	1	0	0	0	2	0		
0	1	0	0	0	2	0	165.0	59.0
0	1	0	0	0	2	0	169.0	78.0
0	1	0	0	0	2	0	173.0	95.0
0	1	0	0	0	2	0	169.0	68.0
0	1	0	0	0	2	0	170.0	85.0
0	1	0	0	0	2	0	170.0	73.0
0	1	0	0	0	2	0	167.0	53.0
0	1	0	0	0	2	0		
0	1	0	1	0	2	0	159.0	55.0
0	1	0	0	0	2	0	166.0	62.0
0	1	0	0	0	2	0		
0	1	0	0	0	2	0		
0	1	0	0	0	2	0	172.0	60.0
0	1	0	0	0	2	0	168.0	82.0
0	1	0	0	0	2	0		
0	1	0	0	0	2	0	171.0	67.0
0	1	0	0	0	2	0	167.0	86.5
0	1	0	0	0	2	0	158.0	54.0
0	1	0	1	0	2	0	170.0	56.0
0	1	0	0	0	2	0	165.0	70.0
0	1	0	0	0	2	0		
0	1	0	0	0	2	0		
0	1	0	0	0	2	0		
0	1	0	0	0	2	0	163.0	66.0
0	1	0	0	0	2	0		
0	1	0	0	0	2	0		
0	1	0	1	0	2	0	163.0	65.0
0	1	0	0	0	2	0		
0	1	0	0	0	2	0		

0	1	0	0	0	2	0		
0	1	0	0	0	2	0	155.0	52.0
0	1	0	0	0	2	0	170.0	63.0
0	1	0	0	0	2	0		
0	1	0	1	0	2	0	####	55.0
0	1	0	0	0	2	0	170.0	63.0
0	1	0	0	0	2	0	164.0	55.0
0	1	0	0	0	2	0		
0	1	0	0	0	2	0	159.0	68.0
0	1	0	0	0	2	0		
0	1	0	1	0	2	0		
0	1	0	0	0	2	0		
0	1	0	0	0	2	0		
0	1	0	0	0	2	0	161.0	50.0
0	1	0	0	0	2	0	####	54.0
0	1	0	0	0	2	0		
0	1	0	1	0	2	0	187.0	110.0
0	1	0	1	0	2	0		
0	1	0	0	0	2	0		
0	1	0	0	0	2	0	170.0	66.0
0	1	0	0	0	2	0	158.0	65.0
0	1	0	0	0	2	0	162.0	52.0
0	1	0	0	0	2	0	####	38.0
0	1	0	0	0	2	0		
0	1	0	0	1	2	0		
0	1	0	0	0	2	0		
0	1	0	0	0	2	0	166.0	75.0
0	1	0	0	0	2	0		
0	1	0	0	0	2	0	171.0	119.0
0	1	0	1	0	2	0	164.0	54.0
0	1	0	0	0	2	0	161.0	60.0
0	1	0	0	1	2	0	167.0	73.0
0	1	0	0	0	2	0	168.5	60.0
0	1	0	0	0	2	0	172.0	69.0
0	1	0	0	0	2	0		
0	1	0	0	0	2	0		
0	1	0	0	0	2	0		
0	1	0	0	0	2	0	166.0	58.0
0	1	0	0	0	2	0	165.0	48.0
0	1	0	0	0	2	0		
0	1	0	0	0	2	0	168.0	53.0
0	1	0	0	0	2	0		

0	1	0	0	0	2	0		
0	1	0	0	0	2	0		
0	1	0	0	0	2	0	####	67.7
0	1	0	0	0	2	0		
0	1	0	1	0	2	0	175.0	74.0
0	1	0	0	0	2	0		
0	1	0	0	0	2	0		
0	1	0	0	0	2	0	159.0	46.0
0	1	0	0	0	2	0	165.0	62.0
0	1	0	0	0	2	0	####	67.0
0	1	0	0	0	2	0		53.0
0	1	0	1	0	2	0		
0	1	0	0	0	2	0	165.0	79.0
0	1	0	0	0	2	0	162.0	67.0
0	1	0	0	0	2	0	169.0	106.0
0	1	0	0	0	2	0	172.0	96.0
0	1	0	0	0	2	0	####	65.0
0	1	0	1	0	2	0		
0	1	0	0	0	2	0		
0	1	0	0	0	2	0		
0	1	0	0	0	2	0	172.0	67.6
0	1	0	0	0	2	0		
0	1	0	0	0	2	0		
0	1	0	0	0	2	0	166.0	68.0
0	1	0	0	0	2	0	188.0	76.0
0	1	0	0	0	2	0		
0	1	0	0	0	2	0	172.0	75.0
0	1	0	0	0	2	0	170.0	92.0
0	1	0	0	0	2	0		
0	1	0	0	0	2	0		
0	1	0	0	0	2	0	164.0	72.0
0	1	0	0	0	2	0	180.0	76.0
0	1	0	0	0	2	0	167.0	64.0
0	1	0	0	0	2	0	169.0	91.0
0	1	0	0	0	2	0	179.0	105.0
0	1	0	0	0	2	0		
0	1	0	0	0	2	0	167.5	71.1
0	1	0	0	0	2	0	165.0	74.0
0	1	0	0	0	2	0	160.0	68.0
0	1	0	0	0	2	0		
0	1	0	0	0	2	0	165.0	55.0
0	1	0	0	0	2	0	173.0	64.0

0	1	0	1	0	2	0	165.0	71.0
0	1	0	0	0	2	0		
0	1	0	1	0	2	0	170.0	66.0
0	1	0	0	0	2	0	164.0	66.0
0	1	0	0	0	2	0	155.0	57.0
0	1	0	0	0	2	0		
0	1	0	1	0	2	0	164.0	56.0
0	1	0	0	0	2	0		
0	1	0	0	0	2	0	173.0	72.0
0	1	0	0	0	2	0		
0	1	0	0	0	2	0	172.0	84.5
0	1	0	1	0	2	0		
0	1	0	0	0	2	0	####	70.0
0	1	0	0	0	2	0		
0	1	0	0	0	2	0		
0	1	0	0	0	2	0	175.0	101.0
0	1	0	0	0	2	0	164.0	100.0
0	1	0	0	0	2	0		
0	1	0	0	0	2	0	172.0	77.0
0	1	0	0	0	2	0	174.0	98.0
0	1	0	0	0	2	0	164.0	79.0
0	1	0	0	0	2	0		
0	1	0	0	0	2	0		
0	1	0	1	0	2	0	160.0	67.0
0	1	0	0	0	2	0		
0	1	0	0	0	2	0	164.2	49.0
0	1	0	0	0	2	0		
0	1	0	0	1	2	0	167.0	54.0
0	1	0	0	0	2	0	162.5	74.4
0	1	0	0	0	2	0	162.0	56.0
0	1	0	0	0	2	0		
0	1	0	0	0	2	0	162.0	85.0
0	1	0	0	0	2	0	170.0	80.0
0	1	0	0	0	2	0		
0	1	0	0	1	2	0		
0	1	0	0	0	2	0	166.0	67.0
0	1	0	0	0	2	0		
0	1	0	0	0	2	0	171.0	75.0
0	1	0	0	0	2	0		
0	1	0	0	0	2	0	174.0	83.0
0	1	0	0	0	2	0		
0	1	0	0	0	2	0		

0	1	0	0	0	2		0		
0	1	0	1	0	2		0	167.0	73.3
0	1	0	0	0	2		0	####	46.0
0	1	0	0	0	2		0		
0	1	0	0	0	2		0	157.0	66.0
0	1	0	0	0	2		0	173.0	75.0
0	1	0	0	0	2		0	153.0	64.0
0	1	0	0	0	2		0		
0	1	0	0	0	2		0		
0	1	0	0	0	2		0		
0	1	0	0	0	2		0		
0	1	0	0	0	2		0	63.0	38.0
0	1	0	0	0	2		0	167.6	56.1
0	1	0	0	0	2		0		
0	1	0	0	0	2		0	164.0	73.0
0	1	0	0	0	2		0		
0	1	0	0	0	2		0		
0	1	0	0	0	2		0		
0	1	0	0	0	2		0	168.5	89.0
0	1	0	0	0	2		0	161.0	55.5
0	1	0	0	0	2		0		
0	1	0	1	0	2		0	173.0	74.0
0	1	0	0	0	2		0	####	43.0
0	1	0	1	0	2		0	161.0	66.0
0	1	0	0	0	2		0		
0	1	0	0	0	2		0	167.0	75.0
0	1	0	1	0	2		0		
0	1	0	0	0	2		0	178.0	105.0
0	1	0	0	0	2		0	####	51.0
0	1	0	0	0	2		0		
0	1	0	1	0	2		0	168.0	65.0
0	1	0	0	0	2		0	161.0	70.0
0	1	0	0	1	2		0		
0	1	0	0	0	2		0	173.0	70.0
0	1	0	0	0	2		0		
0	1	0	0	0	2		0		
0	1	0	1	0	2		0	170.0	72.0
0	1	0	0	0	2		0	169.0	70.0
0	1	0	0	0	2	1	0	173.0	72.0
0	1	0	0	0	2		0	171.0	84.8
0	1	0	0	0	2		0	180.0	105.0
0	1	0	0	0	2		0	####	68.0

0	1	0	0	0	2	0	####	91.0
0	1	0	0	0	2	0	170.0	69.0
0	1	0	0	0	2	0		
0	1	0	0	0	2	0		
0	1	0	0	1	2	0	157.0	58.0
0	1	0	0	0	2	0	172.0	83.0
0	1	0	1	0	2	1		
0	1	0	0	0	2	1		
1	1	0	1	0	2	0	####	59.0
1	1	0	1	0	2	0	164.0	54.0
1	1	0	0	0	2	0	175.0	80.0
1	1	0	0	0	2	0	182.0	80.0
1	1	0	0	0	2	0		
1	1	0	0	0	2	0		
1	1	0	1	0	2	0	156.0	56.0
1	1	0	0	0	2	0	167.0	37.0
1	1	0	0	0	2	0	166.0	58.0
1	1	0	0	0	2	0	155.0	63.0
1	1	0	0	0	2	0	158.0	63.0
1	1	0	0	0	2	0	162.0	68.0
1	1	0	0	0	2	0	157.0	63.0
1	1	0	1	0	2	0	153.0	47.0
1	1	0	0	0	2	0	169.0	65.0
1	1	0	0	0	2	0		
1	1	0	0	0	2	0	165.0	80.0
1	1	0	0	0	2	0		
1	1	0	0	0	2	0	168.0	65.0
1	1	0	0	0	2	0	167.0	82.3
1	1	0	0	1	2	0	167.0	75.0
1	1	0	0	0	2	0	172.0	87.0
1	1	0	0	0	2	0	168.0	119.0
1	1	0	0	0	2	0		
1	1	0	0	0	2	0	####	64.0
1	1	0	0	0	2	0		
1	1	0	0	0	2	0	172.0	75.0
1	1	0	0	0	2	0	168.0	75.0
1	1	0	0	0	2	0	169.0	55.6
1	1	0	0	0	2	0	179.0	62.5
1	1	0	0	0	2	0		
1	1	0	0	0	2	0	170.0	73.0
1	1	0	0	0	2	0	167.5	58.0
1	1	0	0	0	2	0	172.0	69.0

1	1	0	0	0	2	0	156.7	53.0
1	1	0	0	0	2	0		
1	1	0	0	0	2	0	167.0	60.0
1	1	0	0	0	2	0	176.0	77.0
1	1	0	0	0	2	0	161.0	65.0
1	1	0	0	0	2	0		
1	1	0	0	0	2	0	158.0	53.0
1	1	0	1	0	2	0	159.0	56.0
1	1	0	0	0	2	0	####	71.0
1	1	0	0	0	2	0	167.0	68.0
1	1	0	0	0	2	0		
1	1	0	0	0	2	0	169.0	74.0
1	1	0	1	0	2	0	####	67.0
1	1	0	1	0	2	0	####	66.6
1	1	0	1	0	2	0	170.0	79.0
1	1	0	0	0	2	0		
1	1	0	0	0	2	0	160.0	64.0
1	1	0	0	0	2	0	167.0	65.0
1	1	0	0	0	2	0	160.0	60.0
1	1	0	0	0	2	0	158.0	59.0
1	1	0	0	0	2	0	157.0	48.4
1	1	0	0	0	2	0	164.0	62.0
1	1	0	0	0	2	0		
0	1	1	1	0	2	0		
0	1	1	1	0	2	0	170.0	60.0
0	1	1	0	0	2	0	170.0	74.0
0	1	1	0	0	2	0	####	66.0
0	1	1	0	0	2	0	####	57.0
0	1	1	1	0	2	0	174.0	60.0
0	1	1	0	0	2	0	170.0	100.0
0	1	1	1	0	2	0	168.0	59.0
0	1	1	1	0	2	0	160.0	48.0
0	1	1	0	0	2	0	167.0	65.0
0	1	1	0	0	2	0	171.0	101.0
0	1	1	0	0	2	0		
0	1	1	0	0	2	0		
0	1	1	0	0	2	0	160.0	97.0
0	1	1	1	0	2	0	162.0	77.0
0	1	1	0	0	2	0	170.0	66.0
0	1	1	0	0	2	0	165.0	61.0
0	1	1	0	0	2	0	162.0	76.0
0	1	1	0	0	2	0	163.0	54.0

0	1	1	0	0	2	0	169.0	75.0
0	1	1	0	0	2	0	161.0	58.0
0	1	1	0	0	2	0	163.0	70.0
0	1	1	1	0	2	0	####	62.0
0	1	1	0	0	2	0		
0	1	1	0	0	2	0	168.0	86.0
0	1	1	0	0	2	0	166.0	71.0
0	1	1	0	0	2	0	####	54.0
0	1	1	0	0	2	0	175.0	65.0
0	1	1	0	0	2	0		
0	1	1	0	0	2	0	177.0	54.0
0	1	1	0	0	2	0	169.0	63.0
0	1	1	0	0	2	0	172.0	65.0
0	1	1	0	0	2	0		
0	1	1	0	0	2	0	162.0	63.0
0	1	1	1	0	2	0		
0	1	1	0	0	2	0	171.0	70.0
0	1	1	0	0	2	0	175.0	158.1
0	1	1	0	0	2	0		
0	1	1	1	0	2	0		
0	1	1	0	0	2	0	168.0	65.0
0	1	1	0	0	2	0	159.0	63.0
0	1	1	0	0	2	0	149.0	58.0
0	1	1	0	0	2	0		
0	1	1	0	0	2	0		
0	1	1	0	0	2	0		
0	1	1	0	0	2	0	166.0	90.0
0	1	1	0	0	2	0	165.0	68.6
0	1	1	0	0	2	0	170.0	69.0
0	1	1	0	0	2	0	163.0	62.5
0	1	1	0	0	2	0	158.0	59.0
1	1	1	0	0	2	0		
1	1	1	0	0	2	0	168.0	65.0
1	1	1	0	0	2	0	168.0	60.0
1	1	1	0	0	2	0	167.0	73.0
1	1	1	0	0	2	0		
1	1	1	0	0	2	0	168.0	69.0
1	1	1	0	0	2	0	170.0	68.0
1	1	1	0	0	2	0		
1	1	1	1	0	2	0		
1	1	1	0	0	2	0	172.0	78.0
1	1	1	1	0	2	0	167.0	68.0

1	1	1	1	0	2		0	####	62.0
1	1	1	1	0	2		0	167.0	67.0
1	1	1	0	0	2		0	175.0	110.0
1	1	1	0	0	2		0	168.0	54.0
1	1	1	0	0	2		0	162.0	60.0
1	1	1	0	0	2		0	157.0	57.0
1	1	1	1	0	2		0		
1	1	1	0	0	2		0	167.0	71.0
1	1	1	0	0	2		0	175.0	69.0
1	1	1	0	0	2		0	157.0	75.0
1	1	1	1	0	2		0		
1	1	1	0	0	2		0	####	75.7
1	1	1	0	0	2		0		
1	1	1	0	0	2		0	173.0	76.0
1	1	1	1	0	2		0	162.0	63.0
1	1	1	0	0	2		0	####	63.0
1	1	1	0	0	2		0	165.0	64.0
1	1	1	0	0	2		0	160.0	83.0
1	1	1	0	0	2		0		
1	1	1	0	0	2		0	167.0	56.0
1	1	1	0	0	2		0	156.7	62.4
1	1	1	0	0	2		1	163.0	81.0
1	1	1	0	0	2		1	162.0	63.0
0	1	0	0	0	2		0		
0	1	0	0	0	2		0		
0	1	0	0	0	2		0		
0	1	0	0	0	2		0		57.0
0	1	0	0	0	2		0	####	64.0
0	1	0	1	0	2		0	####	67.0
0	1	0	0	0	2		0	168.0	70.0
0	1	0	0	0	2		0	166.0	60.0
0	1	0	0	0	2		0	182.0	70.0
0	1	0	0	0	2		0	167.0	59.0
0	1	0	0	0	2		0		
0	1	0	0	0	2		0		
0	1	0	0	0	2		0		
0	1	0	0	0	2		0		
0	1	0	1	0	2		0		
0	1	0	0	0	2		0	167.0	54.0
0	1	0	0	0	2		0		
0	1	0	1	0	2		0		
0	1	0	0	0	2		0	158.0	44.0

0	1	0	0	0	2	0		
0	1	0	0	0	2	0	####	60.0
0	1	0	0	0	2	0	167.0	63.0
0	1	0	0	0	2	0	166.0	57.0
0	1	0	0	0	2	0		
0	1	0	0	0	2	0		
0	1	0	0	0	2	0	159.0	71.0
0	1	0	0	0	2	0	173.0	73.0
0	1	0	0	0	2	0		
0	1	0	0	0	2	0		
0	1	0	0	0	2	0		
0	1	0	0	0	2	0	161.0	49.0
0	1	0	1	0	2	0	160.0	52.0
0	1	0	1	0	2	0	165.0	60.0
0	1	0	0	0	2	0	165.0	65.0
0	1	0	0	0	2	0	####	61.0
0	1	0	0	0	2	0		
0	1	0	1	0	2	0		
0	1	0	0	0	2	0		
0	1	0	0	0	2	0		
0	1	0	0	0	2	0	163.0	65.0
0	1	0	0	0	2	0		
0	1	0	0	0	2	0		
0	1	0	0	0	2	0		
0	1	0	0	0	2	0	168.0	68.0
0	1	0	1	0	2	0	####	65.0
0	1	0	0	0	2	0	169.0	58.0
0	1	0	0	0	2	0	####	62.0
0	1	0	0	0	2	0	165.0	85.0
0	1	0	0	0	2	0	175.0	63.0
0	1	0	1	0	2	0	157.0	51.0
0	1	0	0	0	2	0		
0	1	0	1	0	2	0		
0	1	0	0	0	2	0		
0	1	0	0	0	2	0	175.0	63.0
0	1	0	0	0	2	0		
0	1	0	0	0	2	0		
0	1	0	0	0	2	0		
0	1	0	0	0	2	0		
0	1	0	1	0	2	0	####	67.0
0	1	0	0	0	2	0	165.0	65.0
0	1	0	0	0	2	0	170.5	57.7

0	1	0	0	0	2	0	####	56.0
0	1	0	0	0	2	0		
0	1	0	0	0	2	0		
0	1	0	1	0	2	0		
0	1	0	0	0	2	0		
0	1	0	0	0	2	0		
0	1	0	1	0	2	0		
0	1	0	0	0	2	0	####	45.0
0	1	0	0	0	2	0		
0	1	0	0	0	2	0	171.0	83.0
0	1	0	0	0	2	0		
0	1	0	0	0	2	0		
0	1	0	0	0	2	0		
0	1	0	0	0	2	0	170.0	67.0
0	1	0	0	0	2	0	169.0	57.0
0	1	0	0	0	1	0		
0	1	0	0	0	1	0	150.0	72.0
0	1	0	0	0	2	0		
0	1	0	0	0	2	0		
0	1	0	0	0	2	0		
0	1	0	0	0	2	0		
0	1	0	0	0	2	0		
0	1	0	0	0	2	0	164.0	59.0
0	1	0	0	0	2	0		
0	1	0	0	0	2	0		
0	1	0	0	0	2	0	157.0	54.0
0	1	0	0	0	2	0		
0	1	0	0	0	2	0	182.3	97.8
0	1	0	0	0	2	0		
0	1	0	0	0	2	0	####	77.8
0	1	0	0	0	2	0		
0	1	0	0	0	2	0		
0	1	0	0	0	2	0	167.0	52.0
0	1	0	0	0	2	0		
0	1	0	0	0	2	0	####	90.7
0	1	0	0	0	2	0		
0	1	0	0	0	2	1		
1	1	0	0	0	2	0		90.6
1	1	0	0	0	2	0	175.0	68.0
1	1	0	0	0	2	0	165.0	56.0
1	1	0	0	0	2	0	178.0	89.0
1	1	0	0	0	2	0	163.0	63.0

1	1	0	0	0	2	0		
1	1	0	0	0	2	0	166.0	61.0
1	1	0	0	0	2	0		
1	1	0	0	0	2	0		
1	1	0	0	0	2	0	157.0	65.0
1	1	0	1	0	2	0	173.0	83.0
1	1	0	0	0	2	0		
1	1	0	0	0	2	0	164.0	62.0
1	1	0	0	0	2	0	163.0	48.0
1	1	0	0	0	2	0		
1	1	0	0	0	2	0		
1	1	0	1	0	2	0	169.0	55.0
1	1	0	0	0	2	0	172.0	69.0
1	1	0	0	0	2	0	161.0	49.0
1	1	0	1	0	2	0		
1	1	0	0	0	2	0		
1	1	0	1	0	2	0	172.0	68.0
1	1	0	0	0	2	0	174.0	108.0
1	1	0	0	0	2	0	160.0	62.0
1	1	0	0	0	2	1	175.0	72.0
1	1	0	0	0	2	1	161.0	62.0
0	1	1	0	0	2	0	164.0	49.0
0	1	1	0	0	2	0		
0	1	1	0	0	2	0	170.0	65.3
0	1	1	0	0	2	0		
0	1	1	0	0	2	0		
0	1	1	0	0	2	0		
0	1	1	0	0	2	0	####	81.0
0	1	1	0	0	2	0	173.0	129.0
0	1	1	0	0	2	0	####	58.2
0	1	1	0	0	2	0	154.0	44.0
0	1	1	0	0	2	0		
0	1	1	0	0	2	0	168.0	80.0
0	1	1	0	0	2	0		88.0
0	1	1	0	0	2	0	173.0	84.0
0	1	1	0	0	2	0		
0	1	1	0	0	2	0	172.0	75.0
0	1	1	0	1	2	0		
0	1	1	0	0	2	1	172.0	50.0
1	1	1	0	0	2	0		
1	1	1	0	0	2	0		57.0
1	1	1	0	0	2	0		

1	1	1	0	0	2		0	165.0	58.0
1	1	1	0	0	2		0	165.0	62.0
1	1	1	0	0	2		0	174.0	64.0
1	1	1	0	0	2		0	164.0	53.0
1	1	1	0	0	2		0	####	60.0
1	1	1	0	0	2		0		
1	1	1	0	0	2		0	160.0	56.0
1	1	1	0	0	2		0	171.0	91.0
1	1	1	0	0	2		0	167.0	71.0
1	1	1	0	0	2		0	172.0	94.0
0	1	0	0	0	2		0	170.0	91.0
0	1	0	0	0	2		0	163.0	63.0
0	1	0	0	0	2	0	0	####	64.0
0	1	0	0	0	2		0	####	58.5
0	1	0	0	0	2		0	169.0	100.0
0	1	0	0	0	2		0	####	48.0
0	1	0	0	0	2		0	176.0	108.0
0	1	0	0	0	2		0	172.0	76.0
0	1	0	0	0	2		0		
0	1	0	1	0	2		0	170.0	60.0
0	1	0	0	0	2		0	####	67.0
0	1	0	0	0	2	0	0	173.0	69.0
0	1	0	0	0	2		0	####	####
0	1	0	0	0	2		0	173.0	65.0
0	1	0	0	0	2		0	167.0	70.0
0	1	0	0	0	2	0	0	####	87.0
0	1	0	0	0	2		0	####	72.0
0	1	0	0	0	2		0	####	76.0
0	1	0	0	0	2	1	0	168.0	76.0
0	1	0	1	0	2		0	####	76.0
0	1	0	0	0	2		0	####	80.0
0	1	0	0	0	2		0	168.0	71.0
0	1	0	0	0	2		0	170.0	83.0
0	1	0	0	0	2	0	0		
0	1	0	0	0	2	0	0	170.0	67.0
0	1	0	0	0	2		0		
0	1	0	0	0	2		0		
0	1	0	1	0	2		0		
0	1	0	0	0	2		0	####	85.0
0	1	0	0	0	2		0		
1	1	0	1	0	2		0	164.0	73.0

1	1	0	0	0	2		0	####	87.0
1	1	0	1	0	2	0	0	159.0	46.0
1	1	0	0	0	2		0	172.0	67.0
1	1	0	0	0	2	0	0		
1	1	0	0	0	2	0	0	170.0	74.0
1	1	0	0	0	2		0	170.0	97.0
1	1	0	0	0	2	0	0	####	53.0
1	1	0	0	0	2		0	####	55.0
1	1	0	0	0	2	0	0	####	71.0
1	1	0	0	0	2		1	165.0	72.0
0	1	1	0	0	2		0	####	56.0
0	1	1	0	0	2		0	160.0	52.0
0	1	1	0	0	2		0	168.0	72.0
0	1	1	1	0	2		0	####	74.0
0	1	1	0	0	2	0	0	163.0	50.0
0	1	1	0	0	2		0	####	####
1	1	1	0	0	2		0	168.0	67.0
1	1	1	0	0	2		0	####	####
1	1	1	0	0	2		0	####	75.0
1	1	1	1	0	2		0	168.0	88.0
1	1	1	0	0	2		0	193.0	113.0
1	1	1	1	0	2		0	160.0	69.0
1	1	1	1	0	2		0	162.0	73.0
1	1	1	0	0	2		0	168.0	61.0
1	1	1	0	0	2	0	0	167.0	62.0
0	1	1	0	0	2		0	164.0	64.0
0	0	0	1	0			0	163.0	50.0
0	0	0	0	0			0		
0	0	0	0	0			0		
0	0	0	0	0			0		
0	0	0	0	0			0		
0	0	0	0	0			0		
0	0	0	0	0			0		
0	0	0	0	0			0		
0	0	0	0	0			0		
0	0	0	0	0			0		
0	0	0	0	0			0		
0	0	0	0	0			0		
0	0	0	0	0			0		
0	0	0	0	0			0		
0	0	0	0	0			0		

0	0	0	0	0	0		
0	0	0	0	0	0		
0	0	0	0	0	0		
0	0	0	1	0	0		
0	0	0	1	0	0		
0	0	0	0	0	0		
0	0	0	0	0	0	165.0	49.0
0	0	0	1	0	0		
0	0	0	0	0	0	170.0	46.0
0	0	0	1	0	0		
0	0	0	0	0	0		
0	0	0	1	0	0	153.0	57.0
0	0	0	1	0	0	168.0	74.0
0	0	0	1	0	0		
0	0	0	0	0	0		
0	0	0	0	0	0		
0	0	0	0	0	0		
0	0	0	0	0	0		
0	0	0	0	0	0		
0	0	0	0	0	0	166.7	63.4
0	0	0	1	0	0	166.0	42.0
0	0	0	0	0	0		
0	0	0	0	0	0	158.0	56.0
0	0	0	0	0	0	174.0	47.0
0	0	0	1	0	0		
0	0	0	0	0	0		
0	0	0	1	0	0		
0	0	0	0	0	0		
0	0	0	0	0	0		
0	0	0	0	0	0		
0	0	0	0	0	0		
0	0	0	0	0	0		
0	0	0	0	0	0		
0	0	0	0	0	0		
0	0	0	1	0	0	156.0	55.0
0	0	0	0	0	0	160.0	84.0
0	0	0	0	0	0		
0	0	0	0	0	0		
0	0	0	0	0	0		
0	0	0	0	0	0		
0	0	0	0	0	0		
0	0	0	0	0	0	180.0	135.0

0	0	0	0	0	0		
0	0	0	0	0	0		
0	0	0	0	0	0		
0	0	0	0	0	0	160.0	60.0
0	0	0	0	0	0		
0	0	0	0	0	0		
0	0	0	0	0	0	169.0	54.0
0	0	0	0	1	0	163.0	58.0
0	0	0	1	0	0		
0	0	0	0	0	0	166.0	66.0
0	0	0	0	0	0		
0	0	0	1	0	0	156.0	72.0
0	0	0	0	0	0		
0	0	0	0	0	0		
0	0	0	0	0	0	169.0	81.0
0	0	0	0	0	0		
0	0	0	0	0	0		
0	0	0	0	0	0		
0	0	0	0	0	0		
0	0	0	0	0	0		
0	0	0	1	0	0		
0	0	0	0	0	0		
0	0	0	0	0	0	168.0	71.0
0	0	0	1	0	0		
0	0	0	0	0	0		
0	0	0	0	0	0		
0	0	0	1	0	0	165.0	70.0
0	0	0	0	0	0	168.0	70.0
0	0	0	1	0	0		
0	0	0	0	0	0		
0	0	0	0	0	0		
0	0	0	0	0	0		
0	0	0	0	0	0		
0	0	0	1	0	0		
0	0	0	0	0	0	171.0	63.0
0	0	0	0	0	0		
0	0	0	0	0	0	169.0	68.0
0	0	0	0	0	0		
0	0	0	0	0	0		
0	0	0	0	0	0		
0	0	0	1	0	0	####	68.0
0	0	0	1	0	0		

0	0	0	1	0	0		
0	0	0	0	0	0		
0	0	0	1	0	0		
0	0	0	0	0	0	####	67.0
0	0	0	1	0	0		
0	0	0	0	0	0		
0	0	0	0	0	0	####	80.0
0	0	0	1	0	0		
0	0	0	1	0	0		
0	0	0	0	0	0		
0	0	0	0	0	0		
0	0	0	0	0	0		
0	0	0	0	0	0	159.0	81.0
0	0	0	0	0	0		
0	0	0	0	0	0	165.0	63.0
0	0	0	0	0	0	152.3	48.7
0	0	0	0	0	0		
0	0	0	0	0	0		
0	0	0	0	0	0	####	77.0
0	0	0	0	0	0		
0	0	0	0	0	0	165.0	69.0
0	0	0	0	0	0	176.0	71.0
0	0	0	0	0	0	164.0	67.0
0	0	0	1	0	0		
0	0	0	1	0	0		
0	0	0	0	0	0		
0	0	0	0	0	0		
0	0	0	0	0	0		
0	0	0	1	0	0		
0	0	0	0	0	0		
0	0	0	1	0	0		
0	0	0	0	0	0		
0	0	0	1	0	0		
0	0	0	0	0	0	168.0	65.0
0	0	0	0	0	0		
0	0	0	1	0	0		
0	0	0	0	0	0		
0	0	0	0	0	0		
0	0	0	1	0	0	####	63.0
0	0	0	0	0	0		
0	0	0	0	0	0		

0	0	0	0	0	0		
0	0	0	0	0	0		
0	0	0	0	0	0	####	62.0
0	0	0	0	0	0		
0	0	0	0	0	0		
0	0	0	0	0	0		
0	0	0	1	0	0		
0	0	0	0	0	0		
0	0	0	0	0	0		
0	0	0	0	0	0		
0	0	0	0	0	0		
0	0	0	1	0	0	161.0	67.0
0	0	0	0	0	0	175.0	62.0
0	0	0	0	0	0		
0	0	0	0	0	0		
0	0	0	0	0	0		
0	0	0	0	0	0		
0	0	0	0	0	0		
0	0	0	0	0	0		
0	0	0	0	0	0		
0	0	0	0	0	0		
0	0	0	0	0	0	165.0	84.0
0	0	0	1	0	0		
0	0	0	0	0	0		
0	0	0	0	0	0		
0	0	0	0	0	0		
0	0	0	1	0	0		
0	0	0	0	0	0	168.0	92.0
0	0	0	0	0	0		
0	0	0	1	0	0		
0	0	0	0	0	0		
0	0	0	0	0	0		
0	0	0	0	0	0		
0	0	0	0	0	0	####	83.0
0	0	0	0	0	0		
0	0	0	0	0	0		
0	0	0	0	0	0		
0	0	0	0	0	0		
0	0	0	0	0	0		
0	0	0	1	0	0		
0	0	0	0	0	0		

0	0	0	0	0	0	####	48.0
0	0	0	1	0	0	180.0	118.0
0	0	0	0	0	0		
0	0	0	0	0	0		
0	0	0	0	0	0		
0	0	0	0	0	0		
0	0	0	0	0	0		
0	0	0	0	0	0		
0	0	0	0	0	0	172.0	82.0
0	0	0	0	0	0		
0	0	0	0	0	0		
0	0	0	1	0	0		
0	0	0	0	0	0		
0	0	0	0	0	0		
0	0	0	0	0	0	170.0	54.0
0	0	0	0	0	0		
0	0	0	0	0	0		
0	0	0	0	0	0		
0	0	0	0	0	0	168.0	85.0
0	0	0	1	0	0	163.5	67.0
0	0	0	0	0	0		
0	0	0	0	0	0		
0	0	0	0	0	0		
0	0	0	0	0	0		
0	0	0	0	0	0		
0	0	0	0	0	0		
0	0	0	0	0	0	169.0	61.0
0	0	0	0	0	0		
0	0	0	0	0	0		
0	0	0	0	0	0	163.0	59.0
0	0	0	0	0	0		
0	0	0	0	0	0		
0	0	0	0	0	0		
0	0	0	1	0	0		
0	0	0	0	0	0		
0	0	0	0	0	0		
0	0	0	0	0	0	####	70.0
0	0	0	0	0	0		
0	0	0	0	0	0		
0	0	0	0	0	0		
0	0	0	0	0	0		

0	0	0	1	0	0		
0	0	0	0	0	0	160.0	61.0
0	0	0	0	0	0		
0	0	0	0	1	0		
0	0	0	0	0	0	####	65.0
0	0	0	0	0	0		
0	0	0	0	0	0		
0	0	0	0	0	0	169.3	70.0
0	0	0	0	0	0		
0	0	0	0	0	0		
0	0	0	0	0	0		
0	0	0	0	0	0	169.0	80.0
0	0	0	0	0	0	####	58.0
0	0	0	0	0	0		
0	0	0	1	0	0		
0	0	0	0	0	0		
0	0	0	0	0	0		
0	0	0	0	0	0		
0	0	0	0	0	0		
0	0	0	0	0	0		
0	0	0	0	0	0	174.0	63.0
0	0	0	0	0	0		
0	0	0	0	0	0		
0	0	0	1	0	0		
0	0	0	0	0	0	170.0	72.5
0	0	0	0	0	0	####	67.1
0	0	0	0	0	0	155.0	70.0
0	0	0	0	0	0		
0	0	0	0	0	0		
0	0	0	0	0	0	169.0	60.0
0	0	0	0	0	0	166.0	62.0
0	0	0	0	0	0		
0	0	0	1	0	0		
0	0	0	0	0	0		
0	0	0	0	0	0		
0	0	0	0	0	0		
0	0	0	0	0	0		
0	0	0	0	0	0		
0	0	0	1	0	0		
0	0	0	0	0	0		
0	0	0	0	0	0		
0	0	0	0	0	0		

0	0	0	0	0	0		
0	0	0	1	0	0		
0	0	0	1	0	0	####	66.0
0	0	0	0	0	0		
0	0	0	0	0	0		
0	0	0	0	0	0		
0	0	0	0	0	0		
0	0	0	0	0	0	####	80.0
0	0	0	0	0	0		
0	0	0	1	0	0		
0	0	0	0	0	0		
0	0	0	0	0	0		
0	0	0	0	0	0	164.0	54.0
0	0	0	0	0	0	172.0	75.0
0	0	0	0	0	0		
0	0	0	1	0	0		
0	0	0	0	1	0	####	67.4
0	0	0	0	0	0		
0	0	0	0	0	0		
0	0	0	0	0	0		
0	0	0	0	0	0		
0	0	0	1	0	0	####	78.0
0	0	0	0	0	0		
0	0	0	1	0	0	171.0	65.0
0	0	0	0	0	0	####	82.2
0	0	0	0	0	0		
0	0	0	0	0	0		
0	0	0	0	0	0	170.0	73.0
0	0	0	0	0	0		
0	0	0	0	0	0	160.7	68.6
0	0	0	0	0	0		
0	0	0	0	0	0		
0	0	0	0	1	0	164.0	68.0
0	0	0	0	0	0		
0	0	0	0	0	0		
0	0	0	0	0	0		
0	0	0	0	0	0		
0	0	0	0	0	0		
0	0	0	0	0	0		
0	0	0	0	0	0		
0	0	0	0	0	0		

0	0	0	0	0	0		
0	0	0	0	0	0		
0	0	0	0	0	0		
0	0	0	0	0	0		
0	0	0	0	0	0		
0	0	0	0	0	0		
0	0	0	1	0	0		
0	0	0	0	0	0		
0	0	0	0	0	0		
0	0	0	1	0	0		
0	0	0	0	0	0		
0	0	0	0	0	0		
0	0	0	0	0	0		
0	0	0	1	1	0	####	76.0
0	0	0	0	0	0		
0	0	0	0	0	0		
0	0	0	0	0	0		
0	0	0	1	0	0		
0	0	0	0	0	0	####	53.0
0	0	0	0	0	0	####	59.1
0	0	0	0	0	0	####	58.5
0	0	0	0	0	0		
0	0	0	0	0	0		
0	0	0	0	0	0		
0	0	0	1	0	0		
0	0	0	0	0	0		
0	0	0	0	0	0		
0	0	0	0	0	0		
0	0	0	0	0	0		
0	0	0	0	0	0		
0	0	0	0	0	0		
0	0	0	0	0	0		
0	0	0	0	0	0		
0	0	0	0	0	0		
0	0	0	0	0	0		
0	0	0	0	0	0		
0	0	0	0	0	0		
0	0	0	0	0	0		
0	0	0	0	0	0	165.0	64.0

0	0	0	0	0		0		
0	0	0	0	0		0		
0	0	0	0	0		0		
0	0	0	1	0		0		
0	0	0	0	0		0		
0	0	0	0	0		0	162.0	85.0
0	0	0	0	0		0		
0	0	0	1	0		0		
0	0	0	0	0		0		
0	0	0	1	0		0		
0	0	0	0	0		0		
0	0	0	1	0		0		
0	0	0	1	0		0		
0	0	0	0	0		0		
0	0	0	0	0		0	160.0	33.7
0	0	0	1	0		0	####	63.5
0	0	0	0	0		0	170.0	73.0
0	0	0	0	0		1		
0	0	0	0	0		1	169.0	55.0
0	0	0	0	0		1	150.0	55.0
0	0	0	1	0		1	168.5	64.0
0	0	0	0	0		1	167.0	72.0
0	0	0	1	0	0	1	168.0	55.0
0	0	0	0	0		1		
1	0	0	0	0		0	####	59.0
1	0	0	0	0		0	150.0	56.0
1	0	0	0	0		0		
1	0	0	1	0		0	160.0	77.0
1	0	0	0	0		0	176.0	81.0
1	0	0	1	0		0		
1	0	0	0	0		0	####	92.0
1	0	0	0	0		0		
1	0	0	1	0		0	164.0	68.0
1	0	0	0	0		0		
1	0	0	1	0		0	164.0	72.0
1	0	0	0	0		0	155.0	61.0
1	0	0	1	0		0	157.0	62.0
1	0	0	1	0		0		
1	0	0	0	0		0		
1	0	0	1	0		0	170.0	54.0
1	0	0	0	0		0	176.0	56.0
1	0	0	0	0		0		

1	0	0	0	0		0	####	79.8
1	0	0	0	0		0	161.0	61.0
1	0	0	0	0		0	168.0	74.0
1	0	0	0	0		0	####	58.0
1	0	0	0	0		0	164.0	59.0
1	0	0	0	0		0	164.0	47.0
1	0	0	1	0		0	160.0	62.0
1	0	0	1	0		0	174.0	64.0
1	0	0	0	0	0	0	174.0	67.0
1	0	0	1	0		0	168.0	91.0
1	0	0	0	0		0	174.0	67.0
1	0	0	0	0		0	####	71.2
1	0	0	0	0		0	166.0	60.0
1	0	0	0	0		0		
0	0	1	0	0		0		
0	0	1	1	0		0		
0	0	1	0	0		0	160.0	65.0
0	0	1	0	0		0	168.0	73.0
0	0	1	0	0		0		
0	0	1	0	0		0		
0	0	1	0	0		0		
0	0	1	1	0		0	155.0	46.0
0	0	1	0	0		0	181.0	152.0
0	0	1	0	0		0	157.0	97.0
0	0	1	0	0		0		
0	0	1	0	0		0	161.5	52.0
0	0	1	0	0		0	####	51.0
0	0	1	0	0		0		
0	0	1	0	0		0	180.0	54.0
0	0	1	0	0		0		
0	0	1	0	0		0	170.0	79.0
0	0	1	0	0		0		
0	0	1	1	0		0		
0	0	1	1	0		0	167.4	66.0
0	0	1	0	0		0		
0	0	1	1	0		0		
0	0	1	0	0		0		
0	0	1	0	0		0	172.0	68.5
0	0	1	0	0		0		
0	0	1	0	0		0		
0	0	1	0	0		0		

0	0	1	0	0	0	165.6	62.2
0	0	1	0	0	0	171.0	96.0
0	0	1	0	0	0		
0	0	1	1	0	0	####	70.0
0	0	1	0	0	0	167.0	98.0
0	0	1	1	0	0	####	52.0
0	0	1	0	0	0		
0	0	1	0	0	0	168.0	62.0
0	0	1	0	0	0	162.0	48.0
0	0	1	0	0	0		
0	0	1	0	0	0	164.0	37.0
0	0	1	0	0	0	####	44.0
0	0	1	0	0	0	162.0	62.0
0	0	1	0	0	0	165.5	67.0
0	0	1	0	0	0		47.0
0	0	1	0	0	0		
0	0	1	0	0	0	162.0	50.0
0	0	1	0	0	0	158.0	52.0
0	0	1	0	0	0		
1	0	1	0	0	0		
1	0	1	1	0	0	173.0	80.0
1	0	1	0	0	0	167.0	47.0
1	0	1	1	0	0		
1	0	1	1	0	0	167.0	63.0
1	0	1	0	0	0	160.0	54.0
1	0	1	0	0	0	161.0	64.0
1	0	1	0	0	0	####	62.0
1	0	1	0	0	0	156.0	42.0
1	0	1	0	0	0	175.0	82.6
1	0	1	1	0	0	168.0	44.0
1	0	1	0	0	0	162.0	49.0
1	0	1	1	0	0	161.0	71.0
1	0	1	0	0	0		
1	0	1	0	0	0		
1	0	1	0	0	0		
1	0	1	0	0	0	####	68.6
1	0	1	0	0	0	####	76.0
1	0	1	1	0	0	165.0	73.0
1	0	1	0	0	0	165.0	48.0
1	0	1	0	0	0	162.0	63.0
1	0	1	0	0	0	165.0	48.0
1	0	1	0	0	0	####	48.0

1	0	1	0	0	0		
0	0	0	0	0	0	164.0	56.0
0	0	0	0	0	0		
0	0	0	1	0	0		
0	0	0	0	0	0	####	54.0
0	0	0	0	0	0		
0	0	0	0	0	0		
0	0	0	1	0	0		
0	0	0	0	0	0		
0	0	0	0	0	0		
0	0	0	1	0	0	165.0	71.0
0	0	0	0	0	0		
0	0	0	0	0	0		
0	0	0	1	0	0		
0	0	0	0	0	0		
0	0	0	1	0	0		
0	0	0	1	0	0		
0	0	0	0	0	0		
0	0	0	0	0	0		
0	0	0	0	0	0		
0	0	0	1	0	0	162.0	58.0
0	0	0	0	0	0	####	67.0
0	0	0	0	0	0		
0	0	0	1	0	0	160.0	40.0
0	0	0	1	0	0		
0	0	0	0	0	0		
0	0	0	0	0	0		
0	0	0	0	0	0	####	78.0
0	0	0	1	0	0		
0	0	0	0	0	0		
0	0	0	1	0	0		
0	0	0	1	0	0		
0	0	0	0	0	0		
0	0	0	0	0	0		
0	0	0	0	0	0		
0	0	0	0	0	0		
0	0	0	0	0	0		
0	0	0	0	0	0	####	74.4
0	0	0	0	0	0		
0	0	0	0	0	0		
0	0	0	0	0	0		
0	0	0	0	0	0		

0	0	0	0	0	0	####	51.0
0	0	0	0	0	0		
0	0	0	1	0	0	####	58.4
0	0	0	0	0	0		
0	0	0	0	0	0		
0	0	0	0	0	0		
0	0	0	1	0	0		
0	0	0	0	0	0		
0	0	0	0	0	0	####	68.0
0	0	0	0	0	0		
0	0	0	1	0	0		
0	0	0	1	0	0		
0	0	0	0	0	0	166.0	62.8
0	0	0	0	0	0		
0	0	0	1	0	0		
0	0	0	0	0	0		
0	0	0	0	0	0		
0	0	0	1	0	0		
0	0	0	0	0	0	####	61.0
0	0	0	0	0	0		
0	0	0	0	0	0		
0	0	0	0	0	0		
0	0	0	0	0	0		
0	0	0	1	0	0		
0	0	0	0	0	0		
0	0	0	1	0	0		
0	0	0	0	0	0		
0	0	0	0	0	0		
0	0	0	0	0	0		
0	0	0	0	0	0		
0	0	0	1	0	0		
0	0	0	0	0	0	####	36.0
0	0	0	0	0	0	####	59.0
0	0	0	0	0	0	####	58.0
0	0	0	0	0	0		
0	0	0	0	0	0		
0	0	0	1	0	0		54.0
0	0	0	1	0	0		
0	0	0	0	0	0		
0	0	0	1	0	0		

0	0	0	0	0	0		
0	0	0	0	0	0		
0	0	0	0	0	0		
0	0	0	0	0	0	170.0	65.0
0	0	0	0	0	0	158.0	51.0
0	0	0	1	0	0		
0	0	0	0	0	0		
0	0	0	0	0	0		
0	0	0	0	0	0		
0	0	0	0	0	0		
0	0	0	0	0	0		
0	0	0	0	0	0	171.0	68.0
0	0	0	0	1	0	157.0	61.0
0	0	0	1	0	0	169.0	69.0
0	0	0	1	0	0	167.0	46.0
0	0	0	1	0	0		
0	0	0	0	0	0	166.0	57.0
0	0	0	0	0	0		
0	0	0	1	0	0		
0	0	0	0	0	0		
0	0	0	1	0	0		
0	0	0	0	0	0		
0	0	0	1	0	0		
0	0	0	0	0	0	178.0	58.0
0	0	0	0	0	0	####	60.0
0	0	0	0	0	0		
0	0	0	0	0	0		
0	0	0	0	0	0	165.0	70.0
0	0	0	0	0	0	134.0	42.0
0	0	0	0	0	0		
0	0	0	0	0	0	168.0	69.0
0	0	0	0	0	0		
0	0	0	0	0	0		
0	0	0	0	0	0		
0	0	0	0	0	0		
0	0	0	0	0	0		
0	0	0	0	0	0		
0	0	0	1	0	0	173.0	73.0
0	0	0	1	0	0		
0	0	0	1	0	0		
0	0	0	0	0	0	157.0	40.0
0	0	0	0	0	0		
0	0	0	0	0	0		

0	0	0	0	0	0		
0	0	0	0	0	0	175.0	68.0
0	0	0	1	0	0	164.0	78.0
0	0	0	0	0	0		
0	0	0	0	0	0		
0	0	0	0	0	0	152.0	56.0
0	0	0	0	0	0		
0	0	0	0	0	0		
0	0	0	0	0	0	166.0	42.0
0	0	0	0	0	0		
0	0	0	1	0	0		
0	0	0	0	0	0	166.0	62.0
0	0	0	1	0	0	156.0	56.0
0	0	0	0	0	0	162.0	52.0
0	0	0	1	0	0		
0	0	0	0	0	0	167.6	60.0
0	0	0	0	0	0	172.0	67.0
0	0	0	0	0	0	159.0	66.0
0	0	0	1	0	0		
0	0	0	1	0	0		
0	0	0	0	0	0		
0	0	0	0	0	0	170.0	53.0
0	0	0	0	0	0		
0	0	0	0	0	0		
0	0	0	0	0	0		
0	0	0	0	0	0		
0	0	0	0	0	0	171.0	65.0
0	0	0	0	0	0		
0	0	0	0	0	0		
0	0	0	0	0	0	159.0	63.0
0	0	0	1	0	0		
0	0	0	0	0	0		
0	0	0	1	0	0	164.0	68.0
0	0	0	0	0	0		
0	0	0	0	0	0		
0	0	0	0	0	0	169.7	62.4
0	0	0	1	0	0		
0	0	0	0	0	0	158.0	44.0
0	0	0	0	0	0		
0	0	0	0	0	0	167.0	58.0
0	0	0	0	0	0		
0	0	0	0	0	0	174.0	68.0
0	0	0	1	0	0	170.0	56.0

0	0	0	1	0	0		
0	0	0	1	0	0		
0	0	0	0	0	0		
0	0	0	0	0	0		
0	0	0	0	0	0	168.0	67.0
0	0	0	1	0	0		
0	0	0	0	0	0		
0	0	0	1	0	0		
0	0	0	0	0	0		
0	0	0	1	0	0		
0	0	0	0	0	0		
0	0	0	0	0	0		
0	0	0	0	0	0		
0	0	0	0	0	0		
0	0	0	0	0	0	####	64.0
0	0	0	1	0	0		
0	0	0	0	0	0	161.0	61.0
0	0	0	0	0	0		
0	0	0	0	0	0		
0	0	0	0	0	0		
0	0	0	1	0	0		
0	0	0	0	0	0		
0	0	0	1	0	0		
0	0	0	1	0	0		
0	0	0	0	0	0		
0	0	0	0	0	0		
0	0	0	0	0	0		
0	0	0	0	0	0		
0	0	0	0	0	0		
0	0	0	1	0	0		
0	0	0	1	0	0	####	43.0
0	0	0	0	0	0		
0	0	0	0	0	0		
0	0	0	1	0	0		
0	0	0	0	0	0	160.0	52.0
0	0	0	0	0	0		
0	0	0	1	0	0		
0	0	0	0	0	0	158.0	60.0
0	0	0	0	0	0		
0	0	0	1	0	0	166.0	62.0
0	0	0	1	0	0		
0	0	0	0	0	0	162.0	61.0

0	0	0	0	0	0	168.0	69.0
0	0	0	0	0	0	160.0	53.0
0	0	0	0	0	0		
0	0	0	0	0	0		
0	0	0	0	0	0		
0	0	0	0	0	0		
0	0	0	0	0	0		
0	0	0	0	0	0	176.0	55.0
0	0	0	0	0	0	171.0	54.0
0	0	0	0	0	0		
0	0	0	1	0	0		
0	0	0	1	0	0		
0	0	0	0	0	0		
0	0	0	1	0	0	####	62.0
0	0	0	0	0	0		
0	0	0	0	0	0		
0	0	0	0	0	0		
0	0	0	1	0	0	165.0	60.0
0	0	0	0	0	0	167.0	61.0
0	0	0	1	0	0		
0	0	0	1	0	0		
0	0	0	0	0	0		
0	0	0	0	0	0		
0	0	0	1	0	0		
0	0	0	0	0	0		
0	0	0	1	0	0		
0	0	0	0	0	0		
0	0	0	0	0	0	160.0	41.0
0	0	0	0	0	0		
0	0	0	0	0	0		
0	0	0	0	0	0	159.0	71.0
0	0	0	0	0	0		
0	0	0	0	0	0		
0	0	0	1	0	0		
0	0	0	0	0	0	175.0	64.0
0	0	0	1	0	0		
0	0	0	0	0	0	176.0	68.0
0	0	0	0	0	0	####	65.0
0	0	0	0	0	0		
0	0	0	0	0	0		
0	0	0	1	0	0	167.0	75.0
0	0	0	1	0	0		

0	0	0	0	0	0		
0	0	0	0	0	0		
0	0	0	0	0	0	164.0	53.0
0	0	0	0	0	0		
0	0	0	0	0	0		
0	0	0	0	0	0		
0	0	0	0	0	0		
0	0	0	0	0	0		
0	0	0	0	0	0		
0	0	0	1	0	0		
0	0	0	0	0	0		
0	0	0	0	0	0		
0	0	0	0	0	0	165.0	42.0
0	0	0	0	0	0		
0	0	0	0	0	0	156.0	48.0
0	0	0	1	0	0	167.0	69.0
0	0	0	0	0	0		
0	0	0	0	0	0		
0	0	0	0	0	0		
0	0	0	1	0	0	166.0	52.0
0	0	0	0	0	0	165.0	68.0
0	0	0	0	0	0	167.0	73.0
0	0	0	0	0	0		
0	0	0	1	0	0	171.0	60.0
0	0	0	0	0	0		
0	0	0	0	0	0	161.0	65.0
0	0	0	1	0	0		
0	0	0	0	0	0		
0	0	0	1	0	0		
0	0	0	1	0	0		
0	0	0	1	0	0	####	54.0
0	0	0	1	0	0		
0	0	0	0	0	0		
0	0	0	1	0	0		
0	0	0	0	0	0		
0	0	0	0	0	0		
0	0	0	0	0	0	172.0	66.0
0	0	0	0	0	0	166.0	60.0
0	0	0	0	0	0	167.0	60.0
0	0	0	0	0	0		
0	0	0	1	0	0		
0	0	0	0	0	0		

0	0	0	0	0	0		
0	0	0	1	0	0		
0	0	0	0	0	0	167.0	55.0
0	0	0	1	0	0		
0	0	0	1	0	0		
0	0	0	0	0	0	####	66.0
0	0	0	0	0	0		
0	0	0	0	0	0	165.0	69.0
0	0	0	0	0	0		
0	0	0	0	0	0		
0	0	0	0	0	0		
0	0	0	0	0	0	####	48.0
0	0	0	0	0	0		
0	0	0	0	0	0	172.0	74.0
0	0	0	0	0	0		
0	0	0	0	1	0		
0	0	0	0	0	0	165.0	65.0
0	0	0	0	0	0		
0	0	0	0	0	0		
0	0	0	0	0	0		
0	0	0	0	0	0		
0	0	0	0	0	0		
0	0	0	0	0	0		
0	0	0	0	0	0		
0	0	0	1	0	0		
0	0	0	0	0	0		
0	0	0	1	0	0	154.0	53.0
0	0	0	0	0	0		
0	0	0	0	0	0		
0	0	0	0	0	0		
0	0	0	1	0	0		
0	0	0	0	0	0	####	68.0
0	0	0	1	0	0		
0	0	0	1	0	0		
0	0	0	0	0	0		
0	0	0	0	0	0		
0	0	0	1	0	0	161.0	64.0
0	0	0	0	0	0		
0	0	0	1	0	0		
0	0	0	1	0	0	159.0	66.5
0	0	0	0	0	0	168.0	57.0
0	0	0	1	0	0	168.0	56.0

0	0	0	0	0	0		
0	0	0	0	0	0		
0	0	0	1	0	0		
0	0	0	0	0	0		
0	0	0	0	0	0		
0	0	0	0	0	0		
0	0	0	1	0	0	161.0	55.0
0	0	0	0	0	0	####	67.0
0	0	0	0	0	0		
0	0	0	0	0	0		
0	0	0	0	0	0		
0	0	0	1	0	0	158.0	52.0
0	0	0	0	0	0	165.0	52.0
0	0	0	0	0	0		
0	0	0	0	0	0		
0	0	0	0	0	0	170.0	57.0
0	0	0	0	0	0		
0	0	0	0	0	0		
0	0	0	0	0	0		
0	0	0	1	0	0	166.0	79.0
0	0	0	0	0	0		
0	0	0	0	0	0		
0	0	0	0	0	0		
0	0	0	1	0	0		
0	0	0	1	0	0	156.0	54.0
0	0	0	1	0	0	155.0	50.0
0	0	0	1	0	0		
0	0	0	1	1	0	156.0	56.0
0	0	0	0	0	0		
0	0	0	0	0	0		
0	0	0	1	0	0		
0	0	0	0	0	0		
0	0	0	1	0	0		
0	0	0	0	0	0	####	59.0
0	0	0	0	0	0		
0	0	0	0	0	0	171.0	63.0
0	0	0	1	0	0		
0	0	0	1	0	0		
0	0	0	1	0	0	162.0	63.0
0	0	0	0	0	0		
0	0	0	1	0	0	160.0	80.0
0	0	0	0	0	0		

0	0	0	1	0	0		
0	0	0	0	0	0		
0	0	0	0	0	0		
0	0	0	1	0	0		
0	0	0	1	0	0		
0	0	0	1	0	0		
0	0	0	0	0	0		
0	0	0	0	0	0		
0	0	0	0	0	0		
0	0	0	0	0	0		
0	0	0	0	0	0		
0	0	0	1	0	0		
0	0	0	0	0	0		
0	0	0	1	0	0		
0	0	0	0	0	0		
0	0	0	1	0	0	158.0	57.0
0	0	0	0	0	0		
0	0	0	0	0	0		
0	0	0	0	0	0		
0	0	0	0	0	0	172.0	80.0
0	0	0	0	0	0	167.0	53.0
0	0	0	0	0	0		
0	0	0	1	0	0		
0	0	0	1	0	0	####	65.0
0	0	0	1	0	0		
0	0	0	0	0	0		
0	0	0	0	1	0		
0	0	0	0	0	0	####	65.0
0	0	0	1	0	0		
0	0	0	0	0	0		
0	0	0	0	0	0		
0	0	0	0	0	0		
0	0	0	0	0	0	####	58.0
0	0	0	1	0	0		
0	0	0	1	0	0		
0	0	0	0	0	0		
0	0	0	0	0	0		
0	0	0	0	0	0		
0	0	0	0	0	0		
0	0	0	0	0	0	177.0	71.0
0	0	0	0	0	0		

0	0	0	0	0	0	####	65.0
0	0	0	1	0	0		
0	0	0	0	0	0		
0	0	0	1	0	0	183.0	76.0
0	0	0	0	0	0		
0	0	0	0	0	0	165.0	56.0
0	0	0	1	0	0		
0	0	0	0	0	0		
0	0	0	1	0	0	####	63.0
0	0	0	1	0	0	####	50.0
0	0	0	0	0	0		
0	0	0	0	0	0	166.0	71.0
0	0	0	0	0	0	157.0	53.0
0	0	0	0	0	0		
0	0	0	1	0	0		
0	0	0	0	0	0		
0	0	0	0	0	0		
0	0	0	0	0	0		
0	0	0	0	0	0	####	63.0
0	0	0	0	0	0		
0	0	0	0	0	0		
0	0	0	0	0	0		
0	0	0	0	0	0		
0	0	0	0	0	0		
0	0	0	0	0	0	####	72.0
0	0	0	0	0	0	####	65.0
0	0	0	0	0	0		
0	0	0	0	0	0		
0	0	0	1	0	0		
0	0	0	0	0	0		
0	0	0	1	0	0		
0	0	0	1	0	0		
0	0	0	0	0	0		
0	0	0	1	0	0	172.0	75.0
0	0	0	0	0	0	168.0	71.0
0	0	0	1	0	0		
0	0	0	0	0	0		
0	0	0	0	0	0		
0	0	0	0	0	0		
0	0	0	0	0	0		
0	0	0	0	0	0	####	98.0

0	0	0	0	0	0	####	59.0
0	0	0	0	0	0		
0	0	0	0	0	0	177.0	94.0
0	0	0	1	0	0	163.0	80.0
0	0	0	0	0	0		
0	0	0	0	0	0	165.0	53.0
0	0	0	0	0	0		
0	0	0	0	0	0	####	73.0
0	0	0	1	0	0		
0	0	0	1	0	0		
0	0	0	0	0	0	159.0	73.0
0	0	0	1	0	0		
0	0	0	0	0	0		
0	0	0	0	0	0		
0	0	0	1	0	0		
0	0	0	1	0	0		
0	0	0	0	0	0	171.0	58.0
0	0	0	0	0	0		
0	0	0	1	0	0		
0	0	0	0	0	0	157.0	64.0
0	0	0	0	0	0		
0	0	0	0	0	0		
0	0	0	0	0	0	####	81.0
0	0	0	0	0	0		
0	0	0	0	0	0		
0	0	0	1	0	0	161.0	51.0
0	0	0	0	0	0	171.0	65.0
0	0	0	0	0	0		
0	0	0	0	0	0	162.0	61.0
0	0	0	0	0	0		
0	0	0	0	0	0		
0	0	0	0	0	0	####	70.0
0	0	0	0	0	0	165.0	61.0
0	0	0	0	0	0	158.0	66.0
0	0	0	0	0	0		
0	0	0	0	0	0	168.0	72.0
0	0	0	1	0	0		
0	0	0	0	0	0	174.0	75.0
0	0	0	0	0	0		
0	0	0	0	0	0		
0	0	0	1	0	0		

0	0	0	0	0	0	161.0	72.0
0	0	0	0	0	0		
0	0	0	0	0	0	165.0	45.0
0	0	0	1	0	0		
0	0	0	0	0	0		
0	0	0	0	0	0		
0	0	0	0	0	0		
0	0	0	0	0	0		
0	0	0	0	0	0		
0	0	0	0	0	0		
0	0	0	1	0	0		
0	0	0	0	0	0		
0	0	0	0	0	0		
0	0	0	0	0	0		
0	0	0	0	0	0		
0	0	0	1	0	0		
0	0	0	0	0	0		
0	0	0	0	0	0	160.0	67.0
0	0	0	0	0	0		
0	0	0	0	0	0		
0	0	0	0	0	0	####	66.0
0	0	0	0	0	0		
0	0	0	0	0	0	170.0	42.0
0	0	0	0	0	0		
0	0	0	0	0	0	####	46.0
0	0	0	0	0	0	177.0	67.0
0	0	0	0	0	0		
0	0	0	0	0	0		
0	0	0	1	0	0	163.0	61.0
0	0	0	0	0	0	167.0	70.0
0	0	0	0	0	0		
0	0	0	0	0	0		
0	0	0	0	0	0		
0	0	0	1	0	0	156.0	43.0
0	0	0	0	0	0		
0	0	0	0	0	0		
0	0	0	1	0	0		
0	0	0	1	0	0	####	51.0
0	0	0	1	0	0	####	58.0
0	0	0	0	0	0	####	51.6
0	0	0	0	0	0		

0	0	0	1	0	0		
0	0	0	0	0	0		
0	0	0	0	0	0		
0	0	0	0	0	0	161.0	57.0
0	0	0	0	0	0		
0	0	0	0	0	0		
0	0	0	1	0	0		
0	0	0	0	0	0		
0	0	0	1	0	0		
0	0	0	1	0	0		
0	0	0	0	0	0		
0	0	0	0	0	0		
0	0	0	0	0	0	174.0	80.0
0	0	0	0	0	0	####	54.0
0	0	0	0	0	0		
0	0	0	0	0	0		
0	0	0	0	0	0		
0	0	0	0	0	0		
0	0	0	1	0	0		
0	0	0	0	0	0		
0	0	0	0	0	0		
0	0	0	0	0	0	179.0	74.3
0	0	0	0	0	0		
0	0	0	0	0	0		
0	0	0	0	0	0		
0	0	0	0	0	0		
0	0	0	0	0	0		
0	0	0	0	0	0		
0	0	0	1	0	0		
0	0	0	0	0	0		
0	0	0	1	0	0		
0	0	0	0	0	0		
0	0	0	0	0	0		
0	0	0	0	0	0		
0	0	0	1	0	0		
0	0	0	0	0	0		
0	0	0	0	0	0	170.0	67.5
0	0	0	1	0	0		
0	0	0	0	0	0	160.0	67.0
0	0	0	0	0	0		
0	0	0	0	0	0		

0	0	0	0	0	0		
0	0	0	1	0	0		
0	0	0	0	0	0		
0	0	0	1	0	0		
0	0	0	0	0	0		
0	0	0	1	0	0		
0	0	0	0	0	0	169.0	65.0
0	0	0	0	0	0	####	54.0
0	0	0	0	0	0		
0	0	0	1	0	0		
0	0	0	0	0	0		
0	0	0	0	0	0		
0	0	0	0	0	0		
0	0	0	0	0	0		
0	0	0	1	0	0	####	70.0
0	0	0	0	0	0	161.0	47.0
0	0	0	1	0	0	####	66.0
0	0	0	1	0	0	####	73.0
0	0	0	0	0	0		
0	0	0	0	0	0	165.0	54.0
0	0	0	0	0	0		
0	0	0	0	0	0		
0	0	0	0	0	0		
0	0	0	0	0	0		
0	0	0	0	0	0		
0	0	0	0	0	0	172.0	69.0
0	0	0	1	0	0		
0	0	0	1	0	0		
0	0	0	0	0	0	####	71.0
0	0	0	0	0	0		
0	0	0	1	0	0	####	51.0
0	0	0	0	0	0	161.0	53.0
0	0	0	1	0	0		
0	0	0	1	0	0		
0	0	0	0	0	0		
0	0	0	0	0	0	####	72.4
0	0	0	0	0	0	167.3	56.3
0	0	0	0	0	0		
0	0	0	0	0	0		
0	0	0	1	0	0		
0	0	0	0	0	0		

0	0	0	0	0	0		
0	0	0	1	0	0		
0	0	0	0	0	0		
0	0	0	1	0	0		
0	0	0	0	0	0		
0	0	0	0	0	0		
0	0	0	0	0	0	161.0	63.0
0	0	0	1	0	0	173.9	60.0
0	0	0	0	0	0		
0	0	0	0	0	0	169.0	65.0
0	0	0	1	0	0		
0	0	0	1	0	0		
0	0	0	0	0	0		
0	0	0	0	0	0		
0	0	0	1	0	0		
0	0	0	0	0	0		
0	0	0	0	0	0		
0	0	0	0	1	0	####	61.0
0	0	0	0	0	0		
0	0	0	0	0	0		
0	0	0	0	0	0		
0	0	0	0	0	0		
0	0	0	1	0	0		
0	0	0	0	0	0		
0	0	0	1	0	0	####	65.0
0	0	0	0	0	0		
0	0	0	0	0	0	####	65.0
0	0	0	1	0	0		
0	0	0	0	0	0	####	53.4
0	0	0	1	0	0	####	70.0
0	0	0	0	0	0		
0	0	0	1	0	0		
0	0	0	1	0	0		
0	0	0	0	0	0		
0	0	0	0	0	0		
0	0	0	0	0	0	####	61.0
0	0	0	0	0	0	177.0	57.4
0	0	0	0	0	0	164.0	75.0
0	0	0	0	0	0		
0	0	0	0	0	0		
0	0	0	0	0	0		

0	0	0	1	0	0		
0	0	0	0	0	0		
0	0	0	0	0	0		
0	0	0	1	0	0	####	57.0
0	0	0	0	0	0		
0	0	0	0	0	0		
0	0	0	0	0	0	164.0	57.0
0	0	0	1	0	0		
0	0	0	0	0	0		
0	0	0	1	0	0	167.0	54.0
0	0	0	0	0	0		
0	0	0	1	0	0		
0	0	0	0	0	0		
0	0	0	0	0	0	177.0	72.6
0	0	0	0	0	0		
0	0	0	0	0	0		
0	0	0	0	0	0		
0	0	0	0	0	0		
0	0	0	0	0	0		
0	0	0	0	0	0		
0	0	0	0	0	0		
0	0	0	0	0	0	167.0	73.4
0	0	0	0	0	0	160.0	63.0
0	0	0	1	0	0	167.0	66.8
0	0	0	0	0	0		
0	0	0	1	0	0		
0	0	0	1	0	0		
0	0	0	0	0	0	####	80.0
0	0	0	1	0	0		
0	0	0	0	0	0		
0	0	0	1	0	0		
0	0	0	0	0	0	####	47.0
0	0	0	1	1	0		
0	0	0	0	0	0		
0	0	0	0	0	0	164.0	72.0
0	0	0	0	0	0		
0	0	0	0	0	0	####	67.1
0	0	0	1	0	0	170.0	60.0
0	0	0	0	0	0	168.8	63.2
0	0	0	0	0	0		
0	0	0	1	0	0		

0	0	0	0	0	0		
0	0	0	0	0	0		
0	0	0	0	0	0		
0	0	0	0	0	0		
0	0	0	1	0	0		
0	0	0	1	0	0		
0	0	0	0	0	0		
0	0	0	0	0	0		
0	0	0	0	0	0		
0	0	0	0	0	0		
0	0	0	0	0	0		
0	0	0	0	0	0	170.0	84.7
0	0	0	0	0	0		
0	0	0	0	0	0		
0	0	0	0	0	0	164.5	67.1
0	0	0	1	0	0	165.0	99.0
0	0	0	0	0	0		
0	0	0	1	0	0	170.0	74.0
0	0	0	0	0	0	162.0	65.0
0	0	0	0	0	0		
0	0	0	1	0	0		
0	0	0	1	0	0	####	56.0
0	0	0	0	0	0		
0	0	0	0	0	0	162.0	64.0
0	0	0	0	0	0		
0	0	0	1	1	0		
0	0	0	1	0	0	180.0	100.0
0	0	0	0	1	0		
0	0	0	0	0	0		
0	0	0	1	0	0		
0	0	0	0	0	0	160.0	67.0
0	0	0	0	0	0		
0	0	0	0	0	0		
0	0	0	0	0	0	####	53.8
0	0	0	0	0	0		
0	0	0	0	0	0		
0	0	0	0	0	0	167.0	65.0
0	0	0	0	0	0		
0	0	0	0	0	0	171.0	52.0
0	0	0	0	0	0	172.0	69.0
0	0	0	0	0	0		
0	0	0	0	0	0	168.0	63.0

0	0	0	0	0	0	####	50.0
0	0	0	0	0	0		
0	0	0	1	0	0		
0	0	0	0	0	0		
0	0	0	0	0	0		
0	0	0	0	0	0		
0	0	0	0	0	0	####	70.0
0	0	0	0	0	0		
0	0	0	0	0	0		
0	0	0	0	0	0		
0	0	0	0	0	0		
0	0	0	0	0	0		
0	0	0	0	0	0		
0	0	0	0	0	0		
0	0	0	1	0	0		
0	0	0	0	0	0	179.0	53.0
0	0	0	0	0	0	161.0	86.0
0	0	0	0	0	0		
0	0	0	0	0	0	####	85.2
0	0	0	0	0	0		
0	0	0	0	0	0	168.0	63.0
0	0	0	0	0	0		
0	0	0	0	0	0		
0	0	0	0	0	0		
0	0	0	0	0	0		
0	0	0	0	0	0		
0	0	0	0	0	0		
0	0	0	0	0	0		
0	0	0	0	0	0		
0	0	0	1	0	0		
0	0	0	0	0	0		
0	0	0	1	0	0		
0	0	0	0	0	0		
0	0	0	0	0	0		
0	0	0	1	0	0	163.0	63.0
0	0	0	0	0	0		
0	0	0	0	0	0		
0	0	0	0	0	0		
0	0	0	0	0	0		
0	0	0	0	0	0		
0	0	0	0	0	0		

0	0	0	1	0	0	####	68.0
0	0	0	0	0	0		
0	0	0	0	0	0		
0	0	0	0	0	0		
0	0	0	0	0	0		
0	0	0	0	0	0	####	34.0
0	0	0	0	0	0		
0	0	0	1	0	0		
0	0	0	0	0	0		
0	0	0	0	0	0		
0	0	0	0	0	0		
0	0	0	1	0	0		
0	0	0	0	0	0		
0	0	0	0	0	0		
0	0	0	0	0	0		
0	0	0	0	0	0	####	57.7
0	0	0	0	0	0		60.0
0	0	0	0	0	0	157.0	44.0
0	0	0	0	0	0		
0	0	0	1	0	0		
0	0	0	0	0	0		
0	0	0	0	0	0	####	63.0
0	0	0	0	0	0		
0	0	0	0	0	0		
0	0	0	0	0	0		
0	0	0	0	0	0	172.0	80.0
0	0	0	0	0	0	166.0	67.0
0	0	0	0	0	0	173.5	66.7
0	0	0	1	0	0		
0	0	0	1	0	0	169.0	57.0
0	0	0	0	0	0		
0	0	0	0	0	0	169.0	53.0
0	0	0	0	0	0		
0	0	0	0	0	0		
0	0	0	0	0	0		
0	0	0	0	0	0		
0	0	0	0	0	0		
0	0	0	1	0	0	168.0	74.0
0	0	0	0	0	0		
0	0	0	0	0	0		
0	0	0	1	0	0		

0	0	0	1	0	0		
0	0	0	0	0	0		
0	0	0	0	0	0		
0	0	0	0	0	0		
0	0	0	0	0	0		
0	0	0	1	0	0		
0	0	0	0	0	0		
0	0	0	0	0	0		
0	0	0	1	0	0		
0	0	0	0	0	0		
0	0	0	0	0	0		
0	0	0	0	0	0		
0	0	0	0	0	0		
0	0	0	0	0	0		
0	0	0	0	0	0		
0	0	0	1	0	0		
0	0	0	0	0	0	183.0	84.0
0	0	0	0	0	0	####	76.0
0	0	0	1	0	0		
0	0	0	0	0	0		
0	0	0	0	0	0		
0	0	0	0	0	0		
0	0	0	0	0	0		
0	0	0	0	0	0		
0	0	0	1	0	0		
0	0	0	0	0	0		
0	0	0	0	0	0		
0	0	0	1	0	0		
0	0	0	0	0	0		
0	0	0	0	0	0		
0	0	0	1	0	0		
0	0	0	0	0	0		
0	0	0	0	0	0		
0	0	0	0	0	0		
0	0	0	1	0	0		
0	0	0	0	0	0		
0	0	0	1	0	0		
0	0	0	0	0	0		
0	0	0	0	0	0		
0	0	0	1	0	0		
0	0	0	0	0	0		

0	0	0	1	0	0		
0	0	0	1	0	0	167.5	72.4
0	0	0	0	0	0		
0	0	0	0	0	0		
0	0	0	0	0	0		
0	0	0	0	0	0		
0	0	0	0	0	0	165.5	66.0
0	0	0	0	0	0	156.0	44.4
0	0	0	0	0	0	171.0	80.0
0	0	0	0	0	0		
0	0	0	0	0	0		
0	0	0	0	0	0		
0	0	0	1	0	0		
0	0	0	1	0	0		
0	0	0	0	0	0		
0	0	0	0	0	0		
0	0	0	1	0	0		
0	0	0	0	0	0		
0	0	0	1	0	0		
0	0	0	0	0	0		
0	0	0	0	0	0	162.0	57.0
0	0	0	0	0	0		
0	0	0	0	0	0		
0	0	0	0	0	0		
0	0	0	0	0	0		
0	0	0	0	0	0	175.0	69.0
0	0	0	0	0	0		
0	0	0	0	0	0		
0	0	0	0	0	0		
0	0	0	0	0	0		
0	0	0	1	0	0		
0	0	0	1	0	0		
0	0	0	0	0	0	154.0	54.0
0	0	0	1	0	0		
0	0	0	0	0	0		
0	0	0	0	0	0		
0	0	0	0	0	0		
0	0	0	1	0	0		
0	0	0	0	1	0		
0	0	0	0	0	0	####	75.0
0	0	0	0	0	0		
0	0	0	1	0	0		

0	0	0	0	0	0		
0	0	0	1	0	0	161.0	62.0
0	0	0	0	0	0		
0	0	0	0	0	0		
0	0	0	0	0	0		
0	0	0	0	0	0		
0	0	0	0	0	0		
0	0	0	0	0	0	184.0	92.0
0	0	0	0	0	0		
0	0	0	0	0	0		
0	0	0	0	0	0		
0	0	0	0	0	0		
0	0	0	0	0	0		
0	0	0	0	0	0		
0	0	0	0	0	0		
0	0	0	0	0	0		
0	0	0	0	0	0		
0	0	0	1	0	0		
0	0	0	0	0	0	166.4	53.4
0	0	0	1	0	0		
0	0	0	1	0	0		
0	0	0	1	0	0		
0	0	0	0	0	0		
0	0	0	1	0	0		
0	0	0	1	0	0		
0	0	0	0	0	0	####	49.5
0	0	0	0	0	0		
0	0	0	1	0	0		
0	0	0	0	0	0		
0	0	0	0	0	0		
0	0	0	0	0	0		
0	0	0	0	0	0		
0	0	0	0	1	0		
0	0	0	0	0	0		
0	0	0	1	0	0		
0	0	0	0	0	0		
0	0	0	1	0	0		
0	0	0	1	0	0		
0	0	0	0	0	0		
0	0	0	1	0	0		
0	0	0	0	0	0		
0	0	0	0	1	0		

0	0	0	0	0	0		
0	0	0	0	0	0		
0	0	0	0	0	0		
0	0	0	0	0	0		
0	0	0	0	0	0		
0	0	0	0	0	0		
0	0	0	0	0	0		
0	0	0	0	0	0		
0	0	0	0	0	0		
0	0	0	0	0	0		
0	0	0	0	0	0	####	66.0
0	0	0	0	0	0		
0	0	0	0	0	0		
0	0	0	0	0	0		
0	0	0	0	0	0		
0	0	0	0	0	0		
0	0	0	0	0	0		
0	0	0	0	0	0		
0	0	0	1	0	0	156.0	58.0
0	0	0	0	0	0		
0	0	0	1	0	0		
0	0	0	0	0	0		
0	0	0	0	0	0		
0	0	0	0	0	0	170.6	83.4
0	0	0	0	0	0		
0	0	0	1	0	0		
0	0	0	1	0	0		
0	0	0	0	0	0		
0	0	0	1	0	0		
0	0	0	0	0	0		
0	0	0	0	0	0		
0	0	0	0	0	0		
0	0	0	0	0	0		
0	0	0	0	0	0		
0	0	0	0	0	0		
0	0	0	0	0	0	170.0	66.0
0	0	0	1	0	0		
0	0	0	0	0	0		
0	0	0	1	0	0		
0	0	0	0	0	0		
0	0	0	0	0	0	165.0	68.0
0	0	0	1	0	0		

0	0	0	1	0	0		
0	0	0	0	0	0		
0	0	0	0	0	0		
0	0	0	0	0	0		
0	0	0	1	0	0		
0	0	0	0	0	0		
0	0	0	1	0	0	####	57.0
0	0	0	1	0	0		
0	0	0	0	0	0	####	62.0
0	0	0	0	0	0		
0	0	0	0	0	0	####	55.0
0	0	0	0	0	0		
0	0	0	0	0	0		
0	0	0	1	0	0		
0	0	0	1	0	0		
0	0	0	0	0	0		
0	0	0	0	0	0		
0	0	0	0	0	0		
0	0	0	0	0	0	165.3	75.0
0	0	0	0	0	0		
0	0	0	0	0	0		
0	0	0	0	0	0		
0	0	0	0	0	0		
0	0	0	1	0	0		
0	0	0	0	0	0		
0	0	0	0	0	0		
0	0	0	0	0	0		
0	0	0	1	0	0		
0	0	0	1	0	0		
0	0	0	0	0	0		
0	0	0	0	0	0		
0	0	0	0	0	0		
0	0	0	1	0	0		
0	0	0	0	0	0		
0	0	0	0	0	0		
0	0	0	0	0	0		
0	0	0	0	0	0		
0	0	0	0	0	0		
0	0	0	1	0	0		
0	0	0	0	0	0		
0	0	0	0	0	0		
0	0	0	0	0	0		

0	0	0	0	0	0		
0	0	0	0	0	0		
0	0	0	0	0	0	162.0	50.1
0	0	0	0	0	0		
0	0	0	0	0	0	167.0	60.0
0	0	0	0	0	0	167.3	72.8
0	0	0	0	0	0		
0	0	0	0	0	0		
0	0	0	0	0	0		
0	0	0	0	0	0		
0	0	0	0	0	0		
0	0	0	1	0	0		
0	0	0	0	0	0		
0	0	0	1	0	0		
0	0	0	0	0	0		
0	0	0	0	0	0		
0	0	0	0	0	0		
0	0	0	0	0	0		
0	0	0	0	0	0		
0	0	0	0	0	0		
0	0	0	0	0	0		
0	0	0	1	0	0		
0	0	0	1	0	0		
0	0	0	0	0	0		
0	0	0	1	0	0		
0	0	0	1	0	0		
0	0	0	0	0	0		
0	0	0	0	0	0		
0	0	0	0	1	0		
0	0	0	0	0	0		
0	0	0	0	0	0		
0	0	0	0	0	0		
0	0	0	1	0	0		
0	0	0	0	0	0		
0	0	0	1	0	0		
0	0	0	0	0	0		
0	0	0	0	0	0		
0	0	0	0	0	0		
0	0	0	1	0	0		
0	0	0	0	0	0		
0	0	0	0	0	0		
0	0	0	0	0	0		

0	0	0	0	0	0		
0	0	0	0	0	0		
0	0	0	0	0	0		
0	0	0	0	0	0	166.0	70.0
0	0	0	0	0	0		
0	0	0	0	0	0		
0	0	0	0	0	0		
0	0	0	0	0	0		
0	0	0	0	0	0		
0	0	0	0	0	0		
0	0	0	0	0	0		
0	0	0	0	0	0		
0	0	0	0	0	0	####	65.0
0	0	0	1	0	0	160.0	58.0
0	0	0	0	0	0		
0	0	0	0	0	0		
0	0	0	0	0	0		
0	0	0	0	0	0		
0	0	0	1	0	0		
0	0	0	0	0	0		
0	0	0	0	0	0		
0	0	0	0	0	0		
0	0	0	1	0	0		
0	0	0	1	0	0		
0	0	0	0	0	0		
0	0	0	0	0	0		
0	0	0	1	0	0		
0	0	0	0	0	0		
0	0	0	0	0	0		
0	0	0	0	0	0		
0	0	0	0	0	0		
0	0	0	0	0	0		
0	0	0	1	0	0		
0	0	0	1	0	0		
0	0	0	1	0	0		
0	0	0	1	0	0		
0	0	0	0	0	0		
0	0	0	0	0	0		
0	0	0	1	0	0		
0	0	0	1	0	0		
0	0	0	1	0	0		
0	0	0	0	0	0	170.8	88.3

0	0	0	0	1	0		
0	0	0	1	0	0		
0	0	0	0	1	0		
0	0	0	0	0	0		
0	0	0	0	0	0		
0	0	0	1	0	0		
0	0	0	1	0	0		
0	0	0	0	0	0		
0	0	0	0	0	0		
0	0	0	1	0	0		
0	0	0	0	0	0		
0	0	0	1	0	0	####	46.0
0	0	0	0	0	0		
0	0	0	1	0	0		
0	0	0	0	0	0		
0	0	0	0	0	0		
0	0	0	0	0	0	####	64.0
0	0	0	0	0	0		
0	0	0	0	0	0		
0	0	0	0	0	0		
0	0	0	0	0	0		
0	0	0	0	0	0		
0	0	0	0	0	0		
0	0	0	0	0	0		
0	0	0	0	0	0		
0	0	0	0	0	0		
0	0	0	0	0	0		
0	0	0	0	0	0		
0	0	0	0	0	0		
0	0	0	0	0	0		
0	0	0	0	0	0		
0	0	0	0	0	0		
0	0	0	0	0	0		
0	0	0	0	0	0		
0	0	0	0	0	0		
0	0	0	1	0	0		
0	0	0	1	0	0		
0	0	0	0	0	0		
0	0	0	1	0	0	176.0	67.0
0	0	0	0	0	0		
0	0	0	0	0	0		
0	0	0	1	0	0		

0	0	0	0	0	0		
0	0	0	0	0	0		
0	0	0	0	0	0		
0	0	0	0	0	0		
0	0	0	0	0	0		
0	0	0	0	0	0		
0	0	0	1	0	0		
0	0	0	0	0	0	169.0	71.0
0	0	0	0	0	0		
0	0	0	1	0	0		
0	0	0	1	0	0		
0	0	0	1	0	0		
0	0	0	0	0	0		
0	0	0	0	0	0		
0	0	0	0	0	0		
0	0	0	0	0	0	165.0	66.0
0	0	0	0	0	0		
0	0	0	0	0	0		
0	0	0	1	0	0		
0	0	0	0	0	0	####	57.0
0	0	0	0	0	0		
0	0	0	0	0	0		
0	0	0	0	0	0		
0	0	0	0	0	0		
0	0	0	1	0	0		
0	0	0	0	0	0		
0	0	0	0	0	0		
0	0	0	0	0	0		
0	0	0	0	0	0	174.0	73.0
0	0	0	0	0	0		
0	0	0	1	0	0		
0	0	0	0	0	0		
0	0	0	0	0	0		
0	0	0	0	0	0		
0	0	0	0	0	0		
0	0	0	0	0	0		
0	0	0	0	0	0		
0	0	0	1	0	0		
0	0	0	0	0	0		
0	0	0	0	0	0	####	80.0
0	0	0	0	0	0		
0	0	0	0	0	0		

0	0	0	0	0	0		
0	0	0	0	0	0		
0	0	0	1	0	0		
0	0	0	1	0	0		
0	0	0	0	0	0		
0	0	0	1	0	0		
0	0	0	0	0	0		
0	0	0	0	0	0		
0	0	0	0	0	0		
0	0	0	0	0	0		
0	0	0	0	0	0		
0	0	0	0	0	0		
0	0	0	0	0	0		
0	0	0	0	0	0		
0	0	0	0	0	0	165.0	65.0
0	0	0	0	0	0	169.0	62.0
0	0	0	0	0	0		
0	0	0	0	0	0		
0	0	0	0	0	0		
0	0	0	0	0	0		
0	0	0	0	0	0		
0	0	0	0	0	0		
0	0	0	0	0	0		
0	0	0	1	0	0		
0	0	0	0	0	0		
0	0	0	0	0	0		
0	0	0	0	0	0		
0	0	0	0	0	0		
0	0	0	0	0	0		
0	0	0	0	0	0		
0	0	0	0	0	0		
0	0	0	0	0	0	172.0	57.0
0	0	0	0	0	0	157.0	46.0
0	0	0	0	0	0		
0	0	0	0	0	0		
0	0	0	0	0	0		
0	0	0	0	0	0		
0	0	0	0	0	0		
0	0	0	0	0	0		
0	0	0	0	0	0	168.0	65.0
0	0	0	0	0	0		
0	0	0	1	0	0		

0	0	0	1	0	0		
0	0	0	0	0	0		
0	0	0	0	0	0		
0	0	0	0	0	0		
0	0	0	0	0	0		
0	0	0	0	0	0	170.0	76.0
0	0	0	0	0	0		
0	0	0	1	0	0	162.0	61.0
0	0	0	1	0	0		
0	0	0	0	0	0	165.5	51.5
0	0	0	1	0	0		
0	0	0	0	0	0		
0	0	0	0	0	0		
0	0	0	0	0	0		
0	0	0	0	0	0	####	56.6
0	0	0	0	0	0		
0	0	0	0	0	0		
0	0	0	0	0	0		
0	0	0	0	0	0		
0	0	0	0	1	0		
0	0	0	1	0	0	161.0	63.0
0	0	0	0	0	0	####	66.0
0	0	0	1	0	0	####	72.0
0	0	0	1	0	0		
0	0	0	1	0	0		
0	0	0	0	0	0	165.0	64.0
0	0	0	1	0	0		
0	0	0	0	0	0	163.0	78.0
0	0	0	0	0	0		
0	0	0	1	0	0		
0	0	0	0	0	0		
0	0	0	0	0	0	166.0	64.0
0	0	0	1	0	0		
0	0	0	0	0	0	174.0	68.0
0	0	0	0	0	0		
0	0	0	0	0	0		
0	0	0	0	0	0		
0	0	0	0	0	0	167.3	66.0
0	0	0	0	0	0		
0	0	0	0	0	0		
0	0	0	0	0	0		
0	0	0	1	0	0		

0	0	0	1	0		0		
0	0	0	0	0		0		
0	0	0	0	0		0	####	72.5
0	0	0	0	1		0		
0	0	0	0	0		0		
0	0	0	0	0		0		
0	0	0	0	0		0		
0	0	0	0	0		0		
0	0	0	0	0		0		
0	0	0	0	0		0		
0	0	0	0	0		0		
0	0	0	1	0		0		
0	0	0	1	0		0		
0	0	0	0	0		0		
0	0	0	0	0		0		
0	0	0	0	0		0		
0	0	0	0	0		0		
0	0	0	0	0		0		
0	0	0	0	0		0		
0	0	0	0	0		0		
0	0	0	1	0		0		
0	0	0	0	0		0		
0	0	0	0	0		0		
0	0	0	0	0		0		
0	0	0	0	0		0		
0	0	0	0	0		0		
0	0	0	0	0		0		
0	0	0	0	0		0	159.6	53.2
0	0	0	0	0		0	171.0	58.3
0	0	0	0	0		0		
0	0	0	0	0		0		
0	0	0	0	0		0		
0	0	0	0	0		0		
0	0	0	0	0		0		
0	0	0	0	0		0		
0	0	0	1	0		0		
0	0	0	0	0		0		
0	0	0	0	0		0	160.7	56.5
0	0	0	1	0		0		
0	0	0	0	0		0	####	74.0
0	0	0	0	0		0		
0	0	0	0	0		0		

0	0	0	0	0	0	168.0	56.0
0	0	0	0	0	0		
0	0	0	0	0	0		
0	0	0	1	0	0		
0	0	0	0	0	0		
0	0	0	0	0	0		
0	0	0	0	0	0		
0	0	0	1	0	0		
0	0	0	1	0	0	167.0	57.0
0	0	0	0	0	0	153.0	51.0
0	0	0	1	0	0		
0	0	0	0	0	0		
0	0	0	0	0	0		
0	0	0	0	0	0		
0	0	0	0	0	0		
0	0	0	1	0	0		
0	0	0	0	0	0		
0	0	0	1	0	0	161.0	60.0
0	0	0	0	0	0		
0	0	0	0	0	0	####	####
0	0	0	1	0	0	####	66.0
0	0	0	0	0	0		
0	0	0	0	0	0		
0	0	0	0	0	0		
0	0	0	0	0	0		
0	0	0	0	0	0		
0	0	0	0	0	0		
0	0	0	0	0	0	180.0	76.0
0	0	0	0	0	0		
0	0	0	0	0	0		
0	0	0	0	0	0		
0	0	0	0	0	0		
0	0	0	0	0	0	159.3	55.9
0	0	0	0	0	0		
0	0	0	0	0	0		
0	0	0	0	0	0		
0	0	0	0	0	0		
0	0	0	0	0	0		
0	0	0	0	0	0		
0	0	0	1	0	0		
0	0	0	0	0	0	157.0	56.0
0	0	0	1	0	0	####	52.2

0	0	0	0	0	0		
0	0	0	0	0	0		
0	0	0	0	0	0		
0	0	0	0	0	0		
0	0	0	1	0	0		
0	0	0	0	0	0		
0	0	0	0	0	0	163.0	56.0
0	0	0	1	0	0		
0	0	0	0	0	0		
0	0	0	0	0	0		
0	0	0	0	0	0		
0	0	0	0	0	0		
0	0	0	0	0	0	####	81.2
0	0	0	0	0	0	####	73.4
0	0	0	0	0	0		
0	0	0	0	0	0		
0	0	0	1	0	0		
0	0	0	0	0	0		
0	0	0	0	0	0		
0	0	0	0	0	0	165.0	57.2
0	0	0	0	0	0	168.0	60.6
0	0	0	0	0	0	171.7	76.7
0	0	0	0	0	0	180.7	72.4
0	0	0	0	0	0	####	83.0
0	0	0	0	0	0		
0	0	0	0	0	0		
0	0	0	0	0	0		
0	0	0	0	0	0		
0	0	0	0	0	0		
0	0	0	0	0	0		
0	0	0	0	0	0		
0	0	0	0	0	0		
0	0	0	0	0	0	168.0	70.0
0	0	0	0	0	0		
0	0	0	0	1	0	163.0	42.0
0	0	0	0	0	0	####	65.0
0	0	0	1	0	0	158.0	57.0
0	0	0	0	0	0		
0	0	0	1	0	0		
0	0	0	1	0	0		
0	0	0	0	0	0	168.3	57.7

0	0	0	0	0	0	160.0	53.0
0	0	0	0	0	0	169.0	66.3
0	0	0	0	0	0	177.8	77.0
0	0	0	0	0	0		
0	0	0	0	0	0	172.4	74.7
0	0	0	0	0	0	170.0	64.5
0	0	0	0	0	0		
0	0	0	0	0	0		
0	0	0	0	0	0		
0	0	0	1	0	0		
0	0	0	1	0	0		
0	0	0	0	0	0		
0	0	0	0	0	0		
0	0	0	1	0	0		
0	0	0	0	0	0	176.0	74.0
0	0	0	0	0	0		
0	0	0	0	0	0		
0	0	0	0	0	0		
0	0	0	0	0	0		
0	0	0	0	0	0		
0	0	0	0	0	0		
0	0	0	0	0	0	173.7	66.0
0	0	0	0	0	0	167.0	61.2
0	0	0	0	0	0	168.4	70.0
0	0	0	0	0	0		
0	0	0	0	0	0		
0	0	0	0	0	0		
0	0	0	0	0	0	166.0	69.0
0	0	0	0	0	0		
0	0	0	1	0	0		
0	0	0	0	0	0		
0	0	0	0	0	0		
0	0	0	0	0	0		
0	0	0	0	0	0		
0	0	0	0	1	0		
0	0	0	0	0	0	173.0	52.5
0	0	0	1	0	0		
0	0	0	0	0	0	176.0	66.0
0	0	0	0	0	0		
0	0	0	0	0	0		
0	0	0	0	0	0	170.3	68.8
0	0	0	0	0	0	166.6	75.6

0	0	0	0	0	0	173.1	75.0
0	0	0	0	0	0		
0	0	0	0	0	0		
0	0	0	0	0	0	168.0	67.7
0	0	0	0	0	0		
0	0	0	0	0	0		
0	0	0	1	0	0		
0	0	0	0	0	0		
0	0	0	0	0	0		
0	0	0	0	0	0		
0	0	0	0	0	0		
0	0	0	0	0	0		
0	0	0	1	1	0		
0	0	0	0	0	0		
0	0	0	1	0	0		
0	0	0	0	0	0		
0	0	0	0	0	0	166.0	54.0
0	0	0	0	0	0		
0	0	0	0	0	0	####	68.0
0	0	0	0	0	0	155.0	49.0
0	0	0	0	0	0		
0	0	0	0	0	0		
0	0	0	1	0	0		
0	0	0	0	0	0		
0	0	0	0	0	0		
0	0	0	1	0	0		
0	0	0	0	0	0		
0	0	0	0	0	0		
0	0	0	0	0	0		
0	0	0	0	0	0		
0	0	0	0	0	0	171.1	74.7
0	0	0	0	0	0	186.0	90.6
0	0	0	0	0	0		
0	0	0	1	0	0		
0	0	0	0	0	0		
0	0	0	0	0	0		
0	0	0	0	0	0		
0	0	0	0	0	0		
0	0	0	0	0	0		
0	0	0	0	0	0		
0	0	0	0	0	0		
0	0	0	0	0	0		

0	0	0	0	0		0	114.0	113.0
0	0	0	0	0		0		
0	0	0	0	0		0		
0	0	0	1	0		0	173.0	50.0
0	0	0	0	0		0		
0	0	0	0	0		0		
0	0	0	1	0		0		
0	0	0	1	0		0		
0	0	0	0	0		0		
0	0	0	0	0		0		
0	0	0	1	0		0		
0	0	0	1	0		0		
0	0	0	0	0		0		
0	0	0	1	0		0		
0	0	0	0	0		0		
0	0	0	0	0		0		
0	0	0	0	0		0		
0	0	0	1	0		0		
0	0	0	0	0		0		
0	0	0	0	0		0		
0	0	0	1	0		0		
0	0	0	0	0		0		
0	0	0	1	0		0		
0	0	0	0	0		0		
0	0	0	1	0		0		
0	0	0	1	0		0		
0	0	0	0	0		0		
0	0	0	0	0		0		
0	0	0	0	0		0		
0	0	0	0	0		0		
0	0	0	0	0		0		
0	0	0	0	0		0		
0	0	0	0	0		0		
0	0	0	0	0		0	159.4	58.4
0	0	0	0	0		0	181.0	55.2
0	0	0	0	0		0	165.8	65.0
0	0	0	0	0		0		
0	0	0	0	0		0		
0	0	0	0	0		0	172.6	83.4
0	0	0	0	0		0		
0	0	0	0	0		0		
0	0	0	0	0		0		

0	0	0	1	0	0		
0	0	0	0	0	0		
0	0	0	0	0	0		
0	0	0	0	0	0		
0	0	0	0	0	0	####	36.0
0	0	0	1	0	0		
0	0	0	1	0	0		
0	0	0	1	0	0		
0	0	0	1	0	0		
0	0	0	0	0	0		
0	0	0	1	0	0		
0	0	0	0	0	0		
0	0	0	1	0	0		
0	0	0	0	0	0		
0	0	0	0	0	0	168.0	72.0
0	0	0	1	0	0		
0	0	0	0	0	0		
0	0	0	0	0	0		
0	0	0	0	0	0		
0	0	0	0	0	0		
0	0	0	0	0	0		
0	0	0	0	0	0		
0	0	0	0	0	0		
0	0	0	0	0	0		
0	0	0	0	0	0		
0	0	0	1	0	0		
0	0	0	0	0	0	165.0	58.0
0	0	0	1	0	0		
0	0	0	0	0	0	163.0	77.0
0	0	0	0	0	0		
0	0	0	0	0	0	170.0	65.0
0	0	0	0	0	0		
0	0	0	0	0	0		
0	0	0	0	0	0	####	59.7
0	0	0	1	0	0		
0	0	0	0	0	0		
0	0	0	0	0	0	163.0	57.0
0	0	0	0	0	0		
0	0	0	1	0	0		
0	0	0	0	0	0		
0	0	0	0	0	0		
0	0	0	0	0	0		

0	0	0	0	0	0		
0	0	0	1	0	0		
0	0	0	0	0	0		
0	0	0	1	0	0		
0	0	0	0	0	0		
0	0	0	0	0	0		
0	0	0	1	0	0		
0	0	0	0	0	0		
0	0	0	0	0	0		
0	0	0	0	0	0		
0	0	0	1	0	0		
0	0	0	0	0	0		
0	0	0	1	0	0		
0	0	0	0	0	0		
0	0	0	0	0	0	####	86.0
0	0	0	0	0	0		
0	0	0	0	0	0		
0	0	0	0	0	0		
0	0	0	1	0	0		
0	0	0	0	0	0	180.0	105.0
0	0	0	1	0	0		
0	0	0	0	0	0		
0	0	0	0	0	0		
0	0	0	0	0	0		
0	0	0	1	0	0		
0	0	0	1	0	0		
0	0	0	0	0	0		
0	0	0	0	0	0		
0	0	0	0	0	0		
0	0	0	0	0	0		
0	0	0	0	0	0		
0	0	0	0	0	0		
0	0	0	0	0	0		
0	0	0	0	0	0		
0	0	0	0	0	0		
0	0	0	0	0	0	161.0	55.0
0	0	0	0	0	0		
0	0	0	1	0	0	155.0	55.0
0	0	0	0	0	0	160.0	53.0
0	0	0	0	0	0		
0	0	0	1	0	0	165.0	63.0
0	0	0	0	0	0		

0	0	0	0	0	0		
0	0	0	0	0	0		
0	0	0	0	0	0		
0	0	0	0	0	0		
0	0	0	0	0	0		
0	0	0	0	0	0		
0	0	0	0	0	0		
0	0	0	0	0	0		
0	0	0	1	0	0		
0	0	0	0	0	0		
0	0	0	0	0	0	155.0	47.0
0	0	0	0	0	0		
0	0	0	0	0	0		
0	0	0	1	0	0		
0	0	0	1	0	0		
0	0	0	0	0	0		
0	0	0	0	0	0		
0	0	0	1	0	0		
0	0	0	0	0	0	168.1	61.3
0	0	0	0	0	0	178.5	88.1
0	0	0	0	0	0		
0	0	0	0	0	0		
0	0	0	0	0	0		
0	0	0	0	0	0		
0	0	0	0	0	0	161.5	72.5
0	0	0	0	0	0		
0	0	0	0	0	0	####	60.0
0	0	0	1	0	0		
0	0	0	1	0	0		
0	0	0	0	0	0		
0	0	0	1	0	0		
0	0	0	1	0	0		
0	0	0	0	0	0		
0	0	0	0	0	0		
0	0	0	0	0	0		
0	0	0	0	0	0		
0	0	0	0	0	0		
0	0	0	0	0	0		
0	0	0	0	0	0		
0	0	0	0	0	0		
0	0	0	0	0	0		
0	0	0	0	0	0		
0	0	0	0	0	0	170.0	62.0

0	0	0	1	0	0		
0	0	0	0	0	0		
0	0	0	0	0	0		
0	0	0	0	0	0	163.0	50.0
0	0	0	0	0	0		
0	0	0	0	0	0		
0	0	0	0	0	0		
0	0	0	0	0	0		
0	0	0	0	0	0		
0	0	0	0	0	0		
0	0	0	0	0	0		
0	0	0	1	0	0		
0	0	0	0	0	0		
0	0	0	0	0	0		
0	0	0	0	0	0		
0	0	0	0	0	0		
0	0	0	0	0	0		
0	0	0	0	0	0		
0	0	0	1	0	0		
0	0	0	0	0	0		
0	0	0	0	0	0		
0	0	0	0	0	0		
0	0	0	0	0	0		
0	0	0	0	0	0	157.0	53.0
0	0	0	0	0	0		
0	0	0	0	0	0		
0	0	0	0	0	0		
0	0	0	0	0	0		
0	0	0	0	0	0		
0	0	0	0	0	0		
0	0	0	1	0	0		
0	0	0	0	0	0		
0	0	0	0	1	0		
0	0	0	0	0	0		
0	0	0	0	0	0		
0	0	0	0	0	0	####	47.0
0	0	0	0	0	0		
0	0	0	0	0	0	164.0	62.0
0	0	0	0	0	0		
0	0	0	0	0	0	150.0	45.0
0	0	0	0	0	0		
0	0	0	1	0	0		

0	0	0	0	0	0		
0	0	0	0	0	0		
0	0	0	0	0	0		
0	0	0	0	0	0		
0	0	0	0	0	0	####	73.0
0	0	0	0	0	0		
0	0	0	0	0	0		
0	0	0	0	0	0		
0	0	0	0	0	0	164.5	61.8
0	0	0	0	0	0		
0	0	0	0	1	0		
0	0	0	0	0	0		
0	0	0	0	0	0	174.0	65.5
0	0	0	1	0	0		
0	0	0	1	0	0		
0	0	0	0	0	0		
0	0	0	1	0	0		
0	0	0	0	0	0		
0	0	0	0	0	0		
0	0	0	1	0	0		
0	0	0	1	0	0		
0	0	0	0	0	0		
0	0	0	0	0	0		
0	0	0	0	0	0		
0	0	0	0	0	0		
0	0	0	0	0	0		
0	0	0	0	0	0		
0	0	0	0	0	0		
0	0	0	0	0	0		
0	0	0	0	0	0		
0	0	0	0	0	0		
0	0	0	0	0	0		
0	0	0	1	0	0		
0	0	0	0	0	0		
0	0	0	0	0	0	####	60.0
0	0	0	0	0	0		
0	0	0	0	0	0	####	59.0
0	0	0	0	0	0	####	53.0
0	0	0	0	0	0		
0	0	0	1	0	0	####	57.0

0	0	0	0	0	0		
0	0	0	0	0	0		
0	0	0	1	0	0		
0	0	0	0	0	0		
0	0	0	0	0	0	####	72.5
0	0	0	0	0	0		
0	0	0	0	0	0		
0	0	0	0	0	0		
0	0	0	0	0	0		
0	0	0	1	0	0		
0	0	0	1	0	0		
0	0	0	0	0	0		
0	0	0	0	0	0		
0	0	0	1	0	0		
0	0	0	0	0	0		
0	0	0	0	0	0		
0	0	0	0	0	0		
0	0	0	1	0	0		
0	0	0	0	1	0		
0	0	0	0	0	0	####	70.0
0	0	0	0	0	0		
0	0	0	0	0	0		
0	0	0	0	0	0		
0	0	0	0	0	0		
0	0	0	0	0	0		
0	0	0	0	0	0		
0	0	0	0	0	0		
0	0	0	1	0	0		
0	0	0	0	0	0		
0	0	0	0	0	0		
0	0	0	0	0	0		
0	0	0	0	0	0		
0	0	0	0	0	0		
0	0	0	0	0	0		
0	0	0	0	0	0		
0	0	0	0	0	0	####	62.0
0	0	0	1	0	0		
0	0	0	1	0	0		

0	0	0	1	0	0		
0	0	0	1	0	0		
0	0	0	1	0	0		
0	0	0	0	0	0		
0	0	0	1	0	0		
0	0	0	0	0	0		
0	0	0	0	0	0		
0	0	0	0	0	0		
0	0	0	0	0	0		
0	0	0	0	0	0		
0	0	0	0	0	0		
0	0	0	0	0	0		
0	0	0	1	0	0		
0	0	0	0	0	0	####	54.0
0	0	0	0	0	0		
0	0	0	0	0	0		
0	0	0	0	0	0	170.0	68.0
0	0	0	0	0	0	174.0	43.0
0	0	0	1	0	0		
0	0	0	0	0	0		
0	0	0	0	0	0		
0	0	0	0	0	0		
0	0	0	0	0	0		
0	0	0	0	0	0	168.0	78.0
0	0	0	0	0	0		
0	0	0	1	0	0		
0	0	0	0	0	0		
0	0	0	0	0	0		
0	0	0	1	0	0		
0	0	0	0	0	0		
0	0	0	0	0	0	171.0	77.0
0	0	0	1	0	0		
0	0	0	1	0	0	165.0	46.0
0	0	0	0	0	0	####	74.0
0	0	0	0	0	0		
0	0	0	0	0	0		
0	0	0	0	0	0		
0	0	0	0	0	0		
0	0	0	0	0	0		
0	0	0	0	0	0		
0	0	0	0	0	0		

0	0	0	0	0	0		
0	0	0	0	0	0	168.0	84.0
0	0	0	0	0	0		
0	0	0	0	0	0		
0	0	0	0	0	0		
0	0	0	1	0	0		
0	0	0	0	0	0		
0	0	0	0	0	0		
0	0	0	0	0	0		
0	0	0	0	0	0		
0	0	0	1	0	0		
0	0	0	0	0	0		
0	0	0	0	0	0		
0	0	0	0	0	0	165.0	65.0
0	0	0	0	0	0	172.5	85.5
0	0	0	0	0	0	171.0	68.6
0	0	0	0	0	0	169.0	60.0
0	0	0	0	0	0		
0	0	0	0	0	0		
0	0	0	0	0	0		
0	0	0	0	0	0		
0	0	0	0	0	0		
0	0	0	1	0	0		
0	0	0	1	0	0		
0	0	0	0	0	0		
0	0	0	0	0	0		
0	0	0	0	0	0	173.0	84.0
0	0	0	0	0	0		
0	0	0	0	0	0	####	79.0
0	0	0	0	0	0	157.0	60.0
0	0	0	0	0	0		
0	0	0	0	0	0		
0	0	0	0	0	0		
0	0	0	0	0	0		
0	0	0	0	0	0		
0	0	0	0	0	0		
0	0	0	0	0	0		
0	0	0	0	0	0		
0	0	0	0	0	0		
0	0	0	0	0	0		
0	0	0	0	1	0		
0	0	0	1	0	0		

0	0	0	0	0	0		
0	0	0	0	0	0		
0	0	0	0	0	0		
0	0	0	0	0	0		
0	0	0	1	0	0		
0	0	0	0	0	0		
0	0	0	0	0	0		
0	0	0	1	0	0		
0	0	0	0	0	0		
0	0	0	0	0	0		
0	0	0	0	0	0	####	67.0
0	0	0	0	0	0		
0	0	0	0	1	0		
0	0	0	0	0	0		
0	0	0	0	0	0		
0	0	0	0	0	0	####	58.9
0	0	0	0	0	0		
0	0	0	0	0	0		
0	0	0	0	0	0		
0	0	0	0	0	0		
0	0	0	0	0	0	169.0	51.0
0	0	0	1	0	0		
0	0	0	1	0	0		
0	0	0	0	0	0		
0	0	0	0	0	0	####	53.0
0	0	0	1	0	0		
0	0	0	0	0	0		
0	0	0	1	0	0		
0	0	0	1	0	0		
0	0	0	0	0	0		
0	0	0	0	0	0		
0	0	0	1	1	0	169.0	63.0
0	0	0	0	0	0	167.0	72.0
0	0	0	0	0	0		
0	0	0	0	0	0		
0	0	0	1	1	0		
0	0	0	0	0	0		
0	0	0	0	0	0		
0	0	0	0	0	0		
0	0	0	0	0	0		
0	0	0	0	0	0		

0	0	0	0	0	0	154.0	56.0
0	0	0	0	0	0		
0	0	0	0	0	0		
0	0	0	0	0	0		
0	0	0	0	0	0		
0	0	0	1	0	0		
0	0	0	0	0	0		
0	0	0	0	0	0	####	66.0
0	0	0	0	0	0		
0	0	0	0	0	0		
0	0	0	0	0	0	164.0	70.0
0	0	0	0	0	0		
0	0	0	0	0	0		
0	0	0	1	0	0	####	53.0
0	0	0	0	0	0		53.0
0	0	0	0	0	0		
0	0	0	0	0	0		
0	0	0	0	0	0		
0	0	0	0	0	0	153.0	45.0
0	0	0	0	0	0		
0	0	0	0	0	0		
0	0	0	0	0	0		
0	0	0	0	0	0		
0	0	0	0	0	0		
0	0	0	0	0	0		
0	0	0	0	0	0		
0	0	0	0	0	0		
0	0	0	0	0	0		
0	0	0	0	0	0		
0	0	0	0	0	0		
0	0	0	0	0	0		
0	0	0	0	0	0		
0	0	0	0	0	0		
0	0	0	0	0	0		
0	0	0	0	0	0		
0	0	0	0	0	0		
0	0	0	0	0	0		
0	0	0	0	0	0		
0	0	0	0	0	0		
0	0	0	0	0	0		
0	0	0	0	0	0	165.0	36.0

0	0	0	0	0	0		
0	0	0	0	0	0		
0	0	0	1	0	0		
0	0	0	0	1	0		
0	0	0	0	0	0		
0	0	0	0	0	0		
0	0	0	0	0	0		
0	0	0	0	0	0		
0	0	0	0	0	0		
0	0	0	1	0	0		
0	0	0	0	0	0		
0	0	0	0	0	0		
0	0	0	0	1	0		
0	0	0	0	0	0		
0	0	0	0	0	1		
0	0	0	0	0	1	173.0	75.0
0	0	0	0	0	1		
0	0	0	0	0	1		
0	0	0	0	0	1	160.0	75.0
0	0	0	0	0	1	165.0	77.0
0	0	0	0	0	1		
0	0	0	1	0	1		
0	0	0	0	0	1		
0	0	0	0	0	1		
0	0	0	0	0	1		
0	0	0	1	0	1		54.0
0	0	0	0	0	1		
0	0	0	0	0	1		
0	0	0	0	0	1	172.0	59.0
0	0	0	0	0	1		
0	0	0	1	0	1		
0	0	0	1	0	1		
0	0	0	0	0	1	169.0	78.0
0	0	0	0	0	1		
0	0	0	1	0	1	157.0	49.0
0	0	0	0	0	1		
0	0	0	0	0	1	167.0	65.0
0	0	0	1	0	1		
0	0	0	1	0	1		
0	0	0	0	0	1		
0	0	0	1	0	1		
0	0	0	0	0	1		

0	0	0	0	0	1		
0	0	0	0	1	1		
0	0	0	0	0	1		
0	0	0	1	0	1		
0	0	0	1	0	1		
0	0	0	0	0	1		
0	0	0	0	0	1		
0	0	0	1	0	1		
0	0	0	0	0	1		
0	0	0	0	0	1		
0	0	0	0	0	1		
0	0	0	0	0	1		
0	0	0	0	0	1		
0	0	0	0	0	1		
0	0	0	1	0	1		
0	0	0	0	0	1		
0	0	0	0	0	1		
0	0	0	0	0	1	####	64.0
0	0	0	1	0	1		
0	0	0	0	0	1		
0	0	0	0	0	1		
0	0	0	1	0	1		
0	0	0	0	0	1		
0	0	0	0	0	1		
0	0	0	0	0	1		
0	0	0	1	0	1		
0	0	0	0	0	1		
0	0	0	0	0	1		
0	0	0	0	0	1		
1	0	0	1	0	0	####	78.0
1	0	0	1	0	0		
1	0	0	1	0	0	####	56.0
1	0	0	0	0	0	160.0	56.0
1	0	0	1	0	0		
1	0	0	0	0	0		
1	0	0	0	0	0	172.0	71.0
1	0	0	0	0	0		
1	0	0	1	0	0	####	49.0
1	0	0	0	0	0	####	77.0
1	0	0	0	0	0	161.0	55.0
1	0	0	0	0	0		

1	0	0	1	0	0	161.0	50.0
1	0	0	0	0	0	####	58.0
1	0	0	1	0	0		
1	0	0	0	0	0	164.0	57.0
1	0	0	0	0	0		
1	0	0	0	0	0		
1	0	0	1	0	0	167.0	60.0
1	0	0	1	0	0	165.0	61.0
1	0	0	0	0	0	173.0	60.0
1	0	0	0	0	0		
1	0	0	0	0	0	165.0	66.0
1	0	0	0	0	0	####	60.0
1	0	0	1	0	0	175.0	68.0
1	0	0	0	0	0		
1	0	0	1	0	0	166.0	70.0
1	0	0	0	0	0		
1	0	0	1	0	0	193.0	82.0
1	0	0	0	0	0	167.0	64.0
1	0	0	0	0	0	173.0	74.0
1	0	0	0	0	0	176.0	71.0
1	0	0	0	0	0	165.0	78.0
1	0	0	0	0	0		
1	0	0	0	0	0	####	80.0
1	0	0	0	0	0	156.0	53.0
1	0	0	1	0	0		
1	0	0	0	0	0		
1	0	0	0	0	0	166.0	63.0
1	0	0	1	0	0		
1	0	0	0	0	0	167.0	40.0
1	0	0	1	0	0	157.0	52.0
1	0	0	0	0	0	176.0	86.0
1	0	0	0	0	0	170.0	73.0
1	0	0	0	0	0	####	42.0
1	0	0	0	0	0	171.0	63.0
1	0	0	0	0	0		
1	0	0	0	0	0	168.0	75.0
1	0	0	0	0	0	####	76.0
1	0	0	1	0	0		
1	0	0	0	0	0		
1	0	0	0	0	0		
1	0	0	0	0	0	####	79.0
1	0	0	1	0	0	####	70.0

1	0	0	0	0	0		
1	0	0	0	0	0		
1	0	0	0	0	0		
1	0	0	0	0	0	####	53.0
1	0	0	1	0	0	155.0	42.0
1	0	0	0	0	0	####	56.0
1	0	0	0	0	0	153.0	51.0
1	0	0	1	0	0		
1	0	0	1	0	0		
1	0	0	0	0	0		
1	0	0	0	0	0		
1	0	0	1	0	0		
1	0	0	0	0	0	####	77.0
1	0	0	0	0	0	####	73.0
1	0	0	1	0	0		
1	0	0	1	0	0		
1	0	0	1	0	0		
1	0	0	0	0	0	163.0	55.0
1	0	0	0	0	0	####	66.0
1	0	0	0	0	0		91.0
1	0	0	1	0	0		
1	0	0	0	0	0		
1	0	0	0	0	0	166.0	68.0
1	0	0	1	0	0		
1	0	0	0	0	0	160.0	74.0
1	0	0	0	0	0	175.0	76.0
1	0	0	0	0	0		
1	0	0	1	0	0		
1	0	0	0	0	0		
1	0	0	0	0	0	####	59.0
1	0	0	0	0	0		
1	0	0	0	0	0		
1	0	0	1	0	0	####	55.0
1	0	0	0	0	0		
1	0	0	0	0	0		
1	0	0	0	0	0		
1	0	0	0	0	0	176.8	82.0
1	0	0	0	0	0		
1	0	0	1	0	0		
1	0	0	0	0	0		
1	0	0	0	0	0		

1	0	0	1	0	0		
1	0	0	0	0	0		
1	0	0	0	0	0	169.0	69.0
1	0	0	0	0	0	173.0	80.0
1	0	0	1	0	0		
1	0	0	1	0	0		
1	0	0	1	0	0	####	79.3
1	0	0	0	1	0	166.0	69.0
1	0	0	1	0	0		
1	0	0	1	0	0	159.0	77.0
1	0	0	1	0	0		
1	0	0	0	0	0	164.4	56.0
1	0	0	0	0	0		
1	0	0	0	0	0		
1	0	0	0	0	0		
1	0	0	0	0	0	161.0	66.0
1	0	0	1	0	0	160.0	63.0
1	0	0	1	0	0		
1	0	0	0	0	0	####	84.0
1	0	0	0	0	0		
1	0	0	0	0	0		
1	0	0	0	0	0	####	35.0
1	0	0	0	0	0	171.0	102.0
1	0	0	0	0	0		
1	0	0	0	0	0		
1	0	0	1	0	0	160.0	60.0
1	0	0	0	0	0	165.0	57.0
1	0	0	0	0	0		
1	0	0	1	0	0		
1	0	0	0	0	0		
1	0	0	0	0	0		
1	0	0	0	0	0		
1	0	0	1	0	0		
1	0	0	0	0	0		
1	0	0	0	0	0	175.0	76.0
1	0	0	0	0	0		
1	0	0	0	0	0	176.0	103.0
1	0	0	0	0	0	161.0	60.0
1	0	0	0	0	0	171.0	72.5
1	0	0	1	0	0		
1	0	0	1	0	0		
1	0	0	0	0	0		

1	0	0	0	0	0		
1	0	0	0	0	0		
1	0	0	0	0	0	168.0	66.5
1	0	0	0	0	0	####	57.0
1	0	0	0	0	0		
1	0	0	0	0	0		
1	0	0	0	0	0		
1	0	0	1	0	0	####	63.0
1	0	0	1	0	0		
1	0	0	0	0	0		
1	0	0	0	0	0		
1	0	0	0	0	0		
1	0	0	0	0	0		
1	0	0	0	0	0		
1	0	0	0	0	0	160.0	66.0
1	0	0	0	0	0		
1	0	0	0	0	0		
1	0	0	0	0	0		
1	0	0	1	0	0		
1	0	0	0	0	0		
1	0	0	1	0	0	166.0	69.0
1	0	0	1	0	0	154.0	53.0
1	0	0	0	0	0		
1	0	0	1	0	0	161.0	64.0
1	0	0	0	0	0		
1	0	0	0	0	0	####	97.0
1	0	0	0	0	0		
1	0	0	0	0	0		
1	0	0	0	0	0		
1	0	0	1	0	0		
1	0	0	0	0	0		
1	0	0	0	0	0	150.0	48.2
1	0	0	0	0	0		
1	0	0	0	0	0	172.0	70.0
1	0	0	1	0	0		
1	0	0	0	0	0		
1	0	0	0	0	0		
1	0	0	0	0	0	168.0	82.0
1	0	0	1	0	0	164.0	50.0
1	0	0	0	0	0	162.0	81.0
1	0	0	1	0	0		
1	0	0	0	0	0	173.0	76.0

1	0	0	0	0	0		
1	0	0	0	0	0		
1	0	0	0	0	0		
1	0	0	0	0	0		
1	0	0	0	0	0	172.0	66.0
1	0	0	0	0	0	####	56.8
1	0	0	0	0	0		
1	0	0	0	0	0		
1	0	0	0	0	0		
1	0	0	0	0	0		
1	0	0	0	0	0		
1	0	0	0	0	0	####	40.5
1	0	0	1	0	0	####	58.0
1	0	0	1	0	0	163.0	62.0
1	0	0	0	0	0		
1	0	0	0	0	1	172.0	68.0
0	0	0	0	0	0		
0	0	1	1	0	0	165.0	58.0
0	0	1	1	0	0		
0	0	1	0	0	0	180.0	74.0
0	0	1	0	0	0	170.0	70.0
0	0	1	0	0	0		
0	0	1	0	0	0		
0	0	1	0	0	0		53.0
0	0	1	1	0	0		
0	0	1	0	0	0	158.0	63.0
0	0	1	0	0	0		
0	0	1	1	0	0	178.0	97.0
0	0	1	0	0	0		
0	0	1	0	0	0	####	62.0
0	0	1	0	0	0		
0	0	1	0	0	0	173.0	74.0
0	0	1	0	0	0	169.0	60.0
0	0	1	0	0	0		
0	0	1	0	0	0	####	66.0
0	0	1	0	0	0		
0	0	1	0	0	0		
0	0	1	0	0	0	####	62.0
0	0	1	0	0	0		
0	0	1	0	0	0		
0	0	1	0	0	0		

0	0	1	0	0	0	####	48.0
0	0	1	0	0	0		
0	0	1	0	0	0	167.0	71.0
0	0	1	0	0	0	172.0	67.0
0	0	1	0	0	0		
0	0	1	1	0	0	168.0	71.0
0	0	1	0	0	0		
0	0	1	1	0	0		
0	0	1	0	0	0		
0	0	1	0	0	0		
0	0	1	0	0	0		
0	0	1	0	0	0		
0	0	1	1	0	0		
0	0	1	1	0	0		58.0
0	0	1	0	0	0		
0	0	1	0	0	0		
0	0	1	0	0	0		
0	0	1	0	0	0		
0	0	1	0	0	0		
0	0	1	0	0	0	162.0	59.0
0	0	1	0	0	0		
0	0	1	0	0	0		
0	0	1	0	0	0		
0	0	1	1	0	0		
0	0	1	0	0	0		
0	0	1	0	0	0		
0	0	1	0	0	0		
0	0	1	0	0	0		
0	0	1	1	0	0	164.0	65.0
0	0	1	1	0	0		
0	0	1	0	0	0	####	83.0
0	0	1	0	0	0		
0	0	1	0	0	0	171.0	77.5
0	0	1	1	0	0		
0	0	1	0	0	0	165.0	48.0
0	0	1	0	0	0		
0	0	1	0	0	0	164.0	57.0
0	0	1	0	0	0		
0	0	1	0	0	0		
0	0	1	0	0	0		
0	0	1	0	0	0	162.0	94.0
0	0	1	0	0	0		

0	0	1	0	0		0		
0	0	1	0	0		0	176.0	95.0
0	0	1	0	0		0		70.0
0	0	1	0	0		0	####	85.0
0	0	1	0	0		0		
0	0	1	0	0		0	165.0	53.0
0	0	1	0	0		0		
0	0	1	1	0		0		
0	0	1	0	0		0		
0	0	1	0	0		0	166.7	91.0
0	0	1	0	0		0	165.0	54.0
0	0	1	0	0		0	167.0	57.0
0	0	1	0	0		0	####	72.0
0	0	1	0	0		0		
0	0	1	1	0		0		
0	0	1	0	0		0		
0	0	1	0	0		0		
0	0	1	0	0		0		
0	0	1	1	0		0		
0	0	1	0	0		0		
0	0	1	1	0		0	158.0	40.0
0	0	1	1	0		0		
0	0	1	0	0		0	153.0	53.0
0	0	1	0	0		0	161.0	51.0
0	0	1	0	0		0		
0	0	1	0	0		0		
0	0	1	1	0		0	171.0	58.0
0	0	1	0	0		0		
0	0	1	0	0		0		
0	0	1	0	0		0	####	73.0
0	0	1	0	0		0		
0	0	1	0	0		0		
0	0	1	0	0		0	####	46.5
0	0	1	0	1		0	181.0	39.0
0	0	1	1	0		0	####	68.0
0	0	1	0	0		0	####	69.0
0	0	1	0	0		0		
0	0	1	1	0		0	165.0	74.0
0	0	1	1	0		0	168.0	68.0
0	0	1	1	0		0	158.0	44.0
0	0	1	0	0		0	####	54.0

0	0	1	1	0	0	157.0	55.0
0	0	1	0	0	0		
0	0	1	0	0	0	170.0	61.0
0	0	1	0	0	0	157.0	58.0
0	0	1	0	1	0	####	72.0
0	0	1	0	0	0		
0	0	1	0	0	0		
0	0	1	0	0	0	168.0	68.0
0	0	1	0	0	0		
0	0	1	0	0	0		
0	0	1	0	0	0	159.0	47.0
0	0	1	0	0	0	####	53.0
0	0	1	0	0	0		
0	0	1	0	0	0		
0	0	1	0	0	0		
0	0	1	0	0	0	####	42.0
0	0	1	0	0	0		
0	0	1	0	0	0		
0	0	1	0	0	0		
0	0	1	0	0	0		
0	0	1	1	0	0		
0	0	1	0	0	0		
0	0	1	0	0	0		
0	0	1	0	0	0		
0	0	1	0	0	0		60.0
0	0	1	0	0	0	170.0	65.0
0	0	1	0	0	0		
0	0	1	0	0	0		
0	0	1	0	0	0	171.0	60.0
0	0	1	0	0	0		
0	0	1	1	0	0		
0	0	1	0	0	0	####	63.0
0	0	1	1	0	0		
0	0	1	0	0	0		
0	0	1	1	0	0		
0	0	1	0	0	0	152.0	51.0
0	0	1	0	0	0		
0	0	1	0	0	0	####	45.0
0	0	1	0	0	0		
0	0	1	0	0	0		
0	0	1	0	0	0		

0	0	1	0	0	0		
0	0	1	0	0	0	159.0	80.0
0	0	1	1	0	0	166.0	49.0
0	0	1	0	0	0	####	57.0
0	0	1	0	0	0		
0	0	1	0	0	0		
0	0	1	0	0	0		
0	0	1	0	0	0	####	46.2
0	0	1	0	0	0	####	72.0
0	0	1	1	0	0		
0	0	1	0	0	0	164.6	63.6
0	0	1	0	1	0	170.0	70.0
0	0	1	0	0	0	166.0	57.0
0	0	1	0	0	0		
0	0	1	0	0	0	161.0	57.0
0	0	1	0	0	0		
0	0	1	1	0	0		
0	0	1	0	0	0	166.0	59.0
0	0	1	0	0	0	####	49.2
0	0	1	0	0	0		
0	0	1	0	0	0		
0	0	1	1	0	0	167.0	61.0
0	0	1	0	0	0		
0	0	1	1	1	0		
0	0	1	0	0	0		
0	0	1	0	0	0		
0	0	1	1	0	0		
0	0	1	0	0	0		
0	0	1	1	0	0		
0	0	1	0	0	0	172.0	63.0
0	0	1	1	0	0	####	67.0
0	0	1	0	0	0		
0	0	1	0	0	0	165.0	50.0
0	0	1	0	0	0		
0	0	1	0	0	0		
0	0	1	0	0	0	170.0	63.0
0	0	1	1	0	0		
0	0	1	0	0	0		
0	0	1	0	0	0		
0	0	1	0	0	0		
0	0	1	0	0	0		
0	0	1	0	0	0		

0	0	1	1	0	0		
0	0	1	1	0	0		
0	0	1	0	0	0	159.0	54.0
0	0	1	0	0	0		
0	0	1	1	0	0		
0	0	1	0	0	0	174.0	76.0
0	0	1	0	0	0		
0	0	1	0	0	0		
0	0	1	1	0	0		
0	0	1	0	0	0		
0	0	1	0	0	0		
0	0	1	0	0	0		
0	0	1	0	0	0	154.0	48.0
0	0	1	0	0	0		
0	0	1	0	0	0		
0	0	1	0	0	0		
0	0	1	1	0	0		
0	0	1	0	0	0		
0	0	1	0	0	0	####	39.0
0	0	1	0	0	0	####	62.0
0	0	1	1	0	0		
0	0	1	1	0	0	165.0	68.0
0	0	1	0	0	0		
0	0	1	0	0	0		
0	0	1	0	0	0		
0	0	1	1	0	0		
0	0	1	1	0	0		
0	0	1	1	0	0	169.0	70.0
0	0	1	0	0	0	153.0	39.0
0	0	1	0	0	0		
0	0	1	0	0	0		
0	0	1	1	0	0		
0	0	1	0	0	0		
0	0	1	0	0	0		
0	0	1	0	0	0		
0	0	1	0	0	0	162.0	50.0
0	0	1	1	0	0		
0	0	1	0	0	0	####	59.0
0	0	1	0	0	0	####	51.0
0	0	1	0	0	0		
0	0	1	0	0	0		
0	0	1	0	0	0		

0	0	1	0	0	0		
0	0	1	1	1	0	162.0	58.9
0	0	1	0	0	0		
0	0	1	0	0	0	165.0	47.0
0	0	1	0	0	0		
0	0	1	0	0	0		
0	0	1	0	0	0		
0	0	1	0	0	0		
0	0	1	0	0	0		
0	0	1	0	0	0	175.9	60.9
0	0	1	0	0	0		
0	0	1	0	0	0		
0	0	1	0	0	0		
0	0	1	0	0	0		
0	0	1	0	0	0		
0	0	1	0	0	0		
0	0	1	0	0	0		
0	0	1	0	0	0		
0	0	1	0	0	0		
0	0	1	0	0	0	165.0	47.0
0	0	1	0	0	0	169.4	66.0
0	0	1	0	0	0	####	52.0
0	0	1	1	0	0		
0	0	1	0	0	0	177.0	64.0
0	0	1	0	0	0		
0	0	1	0	0	0	165.0	46.5
0	0	1	0	0	0	180.0	67.0
0	0	1	0	0	0		
0	0	1	0	1	0	171.0	57.0
0	0	1	0	0	0	167.0	66.0
0	0	1	0	0	0		
0	0	1	0	0	0		
0	0	1	0	0	0	168.0	94.0
0	0	1	0	0	0		
0	0	1	0	0	0	####	62.5
0	0	1	0	0	0	184.5	84.5
0	0	1	0	0	0	165.0	48.0
0	0	1	0	0	0		
0	0	1	1	0	0		
0	0	1	0	0	0		
0	0	1	0	0	0		
0	0	1	0	0	0	166.0	72.0

0	0	1	0	0	0		
0	0	1	0	0	0		
0	0	1	0	0	0	####	56.0
0	0	1	0	0	0		
0	0	1	0	0	0		
0	0	1	0	0	0		
0	0	1	0	0	0		
0	0	1	0	0	0		
0	0	1	0	0	0	171.0	60.0
0	0	1	0	0	0		
0	0	1	0	0	0		
0	0	1	0	0	0	162.0	42.0
0	0	1	0	1	0		
0	0	1	0	0	0		
0	0	1	1	0	0		
0	0	1	0	0	0		
0	0	1	0	0	0		
0	0	1	0	0	0		
0	0	1	0	0	0		
0	0	1	0	0	0		
0	0	1	0	0	0		
0	0	1	0	0	0		
0	0	1	0	0	0		
0	0	1	0	0	0		
0	0	1	0	0	0		
0	0	1	0	0	0	174.0	66.0
0	0	1	0	1	0		
0	0	1	0	0	0		
0	0	1	1	0	0	####	55.0
0	0	1	0	0	0	170.0	61.0
0	0	1	0	0	0		
0	0	1	0	0	0	####	54.0
0	0	1	0	0	0		
0	0	1	0	0	0		
0	0	1	0	0	0		
0	0	1	0	0	0	####	54.0
0	0	1	0	0	0		
0	0	1	0	0	0	####	72.0
0	0	1	0	0	0		
0	0	1	0	0	0		
0	0	1	1	0	0		

0	0	1	0	0	0		
0	0	1	1	0	0		
0	0	1	0	0	0	####	62.0
0	0	1	0	0	0		
0	0	1	0	1	0		
0	0	1	0	0	0		
0	0	1	0	0	0	163.0	56.0
0	0	1	0	0	0	166.0	50.0
0	0	1	0	0	0		
0	0	1	0	0	0		
0	0	1	0	0	0		
0	0	1	0	0	0		
0	0	1	0	0	0		
0	0	1	0	0	0	####	67.0
0	0	1	0	0	0	####	41.0
0	0	1	0	0	0		
0	0	1	0	0	0		
0	0	1	0	1	0		
0	0	1	0	1	0		
0	0	1	0	1	0		
0	0	1	0	0	0		
0	0	1	0	0	0	162.0	53.0
0	0	1	0	0	0		
0	0	1	0	0	0	173.0	46.0
0	0	1	0	0	0	####	44.1
0	0	1	0	1	0	174.0	58.0
0	0	1	0	0	0		
0	0	1	0	0	0	159.0	43.0
0	0	1	0	0	0		
0	0	1	0	0	0	172.0	56.0
0	0	1	0	0	0		
0	0	1	0	0	0		
0	0	1	0	0	0		
0	0	1	0	0	0		
0	0	1	0	0	0		
0	0	1	0	0	0		
0	0	1	0	0	0		
0	0	1	0	0	0		
0	0	1	0	0	0		
0	0	1	0	1	0		
0	0	1	0	0	0		

0	0	1	0	0	0		
0	0	1	0	0	0		
0	0	1	0	0	0		
0	0	1	0	0	1	162.0	70.0
0	0	1	0	0	1		
0	0	1	1	0	1		
0	0	1	0	0	1		
0	0	1	0	0	1		
0	0	1	0	0	1		
0	0	1	0	0	1		
0	0	1	0	0	1		
1	0	1	1	0	0	####	70.0
1	0	1	0	0	0	####	76.0
1	0	1	1	0	0	164.0	58.0
1	0	1	0	0	0		51.0
1	0	1	1	0	0		
1	0	1	0	0	0	145.0	57.0
1	0	1	0	0	0	161.0	70.0
1	0	1	0	0	0	170.0	66.0
1	0	1	0	0	0	155.0	52.0
1	0	1	0	0	0	161.0	57.0
1	0	1	1	0	0	152.0	73.0
1	0	1	0	0	0		
1	0	1	1	0	0		
1	0	1	1	0	0	161.0	59.0
1	0	1	0	0	0	158.0	55.0
1	0	1	0	0	0	166.5	57.1
1	0	1	0	0	0		
1	0	1	0	0	0	169.0	55.0
1	0	1	0	0	0	158.0	52.0
1	0	1	1	0	0		
1	0	1	0	0	0	165.0	67.0
1	0	1	0	0	0	####	67.0
1	0	1	1	0	0	####	70.0
1	0	1	0	0	0		
1	0	1	1	0	0		
1	0	1	1	0	0		
1	0	1	0	0	0	####	68.0
1	0	1	0	0	0	####	67.0
1	0	1	1	0	0	156.0	62.0
1	0	1	0	0	0	150.0	60.0

1	0	1	0	0	0		
1	0	1	0	0	0		
1	0	1	0	0	0	155.0	50.0
1	0	1	0	0	0	####	47.0
1	0	1	1	0	0		
1	0	1	1	0	0	####	72.0
1	0	1	0	0	0	####	62.0
1	0	1	0	0	0	####	54.0
1	0	1	1	0	0		
1	0	1	1	0	0		
1	0	1	0	0	0	####	66.0
1	0	1	0	0	0	179.0	96.0
1	0	1	1	0	0		
1	0	1	0	0	0		
1	0	1	0	0	0	158.0	73.0
1	0	1	1	0	0		
1	0	1	1	0	0	####	64.0
1	0	1	0	0	0	165.0	65.0
1	0	1	0	0	0		
1	0	1	1	0	0	####	47.0
1	0	1	1	0	0		
1	0	1	0	0	0		
1	0	1	0	0	0	####	52.0
1	0	1	0	0	0	170.0	61.0
1	0	1	0	0	0	160.0	47.7
1	0	1	0	0	0	171.0	39.0
1	0	1	1	0	0	####	55.0
1	0	1	0	0	0		
1	0	1	1	0	0	154.5	83.8
1	0	1	0	0	0	167.0	64.4
1	0	1	0	0	0		
1	0	1	0	0	0	168.0	63.6
1	0	1	0	0	0	176.0	80.0
1	0	1	1	0	0		
1	0	1	0	0	0	160.0	64.0
1	0	1	1	0	0		
1	0	1	0	0	0	172.0	55.0
1	0	1	0	0	0	####	57.0
1	0	1	0	0	0		
1	0	1	1	0	0	160.0	62.0
1	0	1	0	0	0		

1	0	1	1	0	0	156.0	47.0
1	0	1	0	0	0	162.0	61.0
1	0	1	0	1	0		
1	0	1	0	0	0		
1	0	1	1	0	0		
1	0	1	1	0	0	####	51.0
1	0	1	0	0	0	171.5	61.8
1	0	1	1	0	0	154.0	62.0
1	0	1	0	0	0		
1	0	1	0	0	0		
1	0	1	1	0	0	163.0	55.7
1	0	1	1	0	0		
1	0	1	1	0	0		
1	0	1	1	0	0		
1	0	1	1	0	0		
1	0	1	0	0	0		
1	0	1	0	0	0		
1	0	1	0	0	0		
1	0	1	0	0	0		
1	0	1	0	0	0		
1	0	1	0	0	0		
1	0	1	0	0	0	160.0	55.0
1	0	1	1	0	0	####	55.0
1	0	1	0	0	0	160.0	47.0
1	0	1	0	0	0	164.0	61.0
1	0	1	0	0	0	159.0	52.0
1	0	1	0	0	0		
1	0	1	0	0	0	170.0	70.0
1	0	1	0	0	0	150.0	36.0
1	0	1	1	0	0	####	57.0
1	0	1	1	0	0	####	57.0
1	0	1	0	0	0		
1	0	1	1	0	0		
1	0	1	0	0	0		
1	0	1	0	0	0	170.0	74.0
1	0	1	0	0	0		
1	0	1	0	0	0		
1	0	1	0	0	0		
1	0	1	1	0	0		
1	0	1	1	0	0		
1	0	1	0	0	0		
1	0	1	0	0	0		

1	0	1	0	0		0	164.0	48.0
1	0	1	0	0		0		
1	0	1	0	1		0		
1	0	1	0	0		0		
1	0	1	0	0		0		
1	0	1	0	0		0		
1	0	1	0	0		0	164.0	54.0
1	0	1	0	0		0	179.0	83.0
1	0	1	0	0		0		
1	0	1	1	0		0	####	66.0
1	0	1	0	0		0		
1	0	1	0	0		0	####	64.0
1	0	1	1	0		0		
1	0	1	0	0		0		
1	0	1	0	0		0	####	66.0
1	0	1	0	0		0		
1	0	1	1	0		0		
1	0	1	0	0		0	155.0	43.0
1	0	1	0	0		0	173.0	63.0
1	0	1	0	0		0		
1	0	1	0	0		1		
1	0	1	0	0		1		
1	0	1	1	0		1		
1	0	0	0	0		0	168.0	71.0
1	0	0	1	0		0	####	53.0
0	0	0	1	0		0		
0	0	0	1	0		0	165.0	47.0
0	0	0	0	0		0		
0	0	0	1	0		0		
0	0	0	0	0	0	0	####	53.0
0	0	0	1	0		0		
0	0	0	1	0		0	####	56.0
0	0	0	1	0		0		
0	0	0	0	0		0	####	68.0
0	0	0	0	0		0	####	74.0
0	0	0	0	0		0	####	63.0
0	0	0	0	0		0	171.0	72.0
0	0	0	0	0		0		
0	0	0	0	0	0	0	177.0	78.0
0	0	0	0	0		0		
0	0	0	0	0		0		

0	0	0	0	0		0		
0	0	0	0	0		0		
0	0	0	0	0		0	####	90.0
0	0	0	0	0		0		
0	0	0	0	0		1	166.2	81.5
1	0	0	0	0		0	####	73.0
1	0	0	1	0		0		65.0
0	0	1	0	0		0		
0	0	1	1	0		0		77.0
1	0	1	0	0	0	0	161.0	64.0
1	0	1	1	0		0	####	53.0
1	0	1	0	0		0		55.0
0	0	0	0	0		0		
0	0	0	1	0		0	####	67.0
0	0	0	1	0		0		
0	0	0	0	0		0		
0	0	0	0	0		0		
0	0	0	1	1		0		
0	0	0	1	0		0		
0	0	0	0	0		0	160.0	78.0
0	0	0	0	0		0		
0	0	0	0	0		0	####	40.0
0	0	0	0	0		0		
0	0	0	0	0		0	175.0	60.0
0	0	0	1	0		0		
0	0	0	1	0		0	165.0	48.0
0	0	0	1	0		0		
0	0	0	0	0		0		
0	0	0	0	0		0	160.0	59.0
0	0	0	0	0		0		
0	0	0	1	0		0		
0	0	0	0	0		0		
0	0	0	0	0		0	####	78.0
0	0	0	1	0		0		
0	0	0	0	0		0		
0	0	0	0	0		0		
0	0	0	0	1		0		
0	0	0	0	0		0		
0	0	0	0	0		0		
0	0	0	0	0		0		
0	0	0	1	0		0		

0	0	0	0	0	0		
0	0	0	1	0	0		
0	0	0	0	1	0		
0	0	0	0	0	0	176.0	72.5
0	0	0	0	0	0		
0	0	0	1	0	0		
0	0	0	1	0	0	####	55.0
0	0	0	1	0	0	####	65.0
0	0	0	1	0	0		
0	0	0	1	0	0		
0	0	0	0	0	0		
0	0	0	0	0	0		
0	0	0	0	0	0	####	66.0
0	0	0	1	0	0		
0	0	0	1	0	0		
0	0	0	0	0	0		
0	0	0	1	0	0		
0	0	0	1	0	0		
0	0	0	0	0	0		
0	0	0	1	0	0		
0	0	0	0	0	0		
0	0	0	0	0	0	####	46.0
0	0	0	0	0	0		
0	0	0	0	0	0	179.0	79.8
0	0	0	0	0	0		
0	0	0	0	0	0	171.8	70.3
0	0	0	0	0	0		
0	0	0	0	0	0		
0	0	0	0	0	0		
0	0	0	0	0	0		
0	0	0	0	0	0		
0	0	0	1	0	0		
0	0	0	1	0	0		
0	0	0	0	0	0		
0	0	0	1	0	0		
0	0	0	1	0	0		
0	0	0	1	0	0		
0	0	0	1	0	0		
0	0	0	0	0	0		
0	0	0	0	0	0		
0	0	0	0	0	0		

0	0	0	0	0	0	####	79.0
0	0	0	1	0	0		
0	0	0	0	0	0	166.0	71.0
0	0	0	1	0	0	166.0	63.0
0	0	0	0	0	0	####	49.0
0	0	0	0	0	0	166.0	77.0
0	0	0	1	0	0		
0	0	0	0	0	0		
0	0	0	0	0	0	####	53.0
0	0	0	0	1	0		
0	0	0	0	0	1		
0	0	0	0	0	1	173.0	62.0
0	0	0	0	0	1	160.0	59.0
0	0	0	0	0	1	168.0	64.0
0	0	0	1	0	1		
0	0	0	0	0	1		
0	0	0	1	0	1		
0	0	0	0	0	1		
0	0	0	0	0	1		
1	0	0	1	0	0	####	54.0
1	0	0	0	0	0		
1	0	0	1	0	0	165.0	65.0
1	0	0	0	0	0		
1	0	0	1	0	0	####	53.0
1	0	0	1	0	0	175.0	82.0
1	0	0	1	0	0		
1	0	0	0	0	0	171.0	87.0
1	0	0	0	0	0	172.0	67.0
1	0	0	0	0	0	####	60.0
1	0	0	0	0	1	162.0	63.0
0	0	1	1	0	0	####	55.0
0	0	1	0	0	0		
0	0	1	0	0	0		
0	0	1	0	0	0	165.6	45.6
0	0	1	0	0	0		
0	0	1	1	0	0	178.0	81.0
0	0	1	0	0	0	####	62.0
0	0	1	0	0	0		
0	0	1	1	0	0		
0	0	1	0	0	0		
0	0	1	0	0	0	171.0	70.0

0	0	1	1	0			0	####	60.0
0	0	1	0	0			0	####	66.0
0	0	1	0	0			0		
1	0	1	0	0			0	162.0	65.0
1	0	1	0	0			0	158.2	54.0
1	0	1	1	0			0	173.0	79.0
1	0	1	0	0			0		
1	0	1	1	0			0		
coronar	DM	PPI	α －bloc	SSRI	treat	DPP4	urethra	Height	Weight

BMI	duration	retino	smoking	alcho	sex	age	BS	HbA1c
#DIV/0!					1	62	158	7
23.3					1	71	147	6.3
#DIV/0!					1	67	201	6.7
22.9	20	0			1	72	123	6.3
23.8	7	0	0	0	男性	74	110	6
#DIV/0!					男性	79	129	6.4
#DIV/0!					1	75	117	6.7
25.3					1	60	131	7.9
#DIV/0!					男性	58	149	6.8
27.4	5		2	0	1	42	126	7.7
20.1	2	0	0	0	1	69	157	8.3
#DIV/0!					男性	68	223	8.2
25.3	3		1	1	1	71	174	6.4
#DIV/0!					男性	74	151	6.6
#DIV/0!					男性	47	130	7.5
20.0	12	0			男性	65	87	6.5
#DIV/0!					1	62	122	6.1
18.5	14	1	不明	不明	1	56	94	5.5
22.0	12		2	0	1	69	145	6.9
#DIV/0!					1	69	136	6.8
#DIV/0!					男性	66	123	6.5
#DIV/0!					男性	71	129	6.1
#DIV/0!					1	77	171	6.7
#DIV/0!					1	39	308	12.2
#DIV/0!					1	72	202	5.9
#DIV/0!					1	78	165	6.3
23.1	1	0			1	59	161	7.1
20.5	26	0			男性	72	131	6.3
19.4	0	0	1	0	男性	83	129	6.3
24.1	15	0			1	73	120	6.3
0.0	2				1	49	134	5.3
30.2	6	0			男性	39	116	9.5
#DIV/0!	3		2	1	男性	49	126	6.1
19.3	20	0	2	0	1	83	115	6.1
24.2	25	1			1	77	174	6.4
22.1	26	1	0	0	男性	76	161	6.7
#DIV/0!					1	66	164	5.7
#DIV/0!					1	64	141	5.8
#DIV/0!					男性	76	153	7.6
22.8					1	75	148	8.1
#DIV/0!					1	73	108	5.7

#DIV/0!						1	65	158	7
25.5	12	0				1	63	87	7.1
27.5	14	0	1	0		1	37	210	8.8
#DIV/0!						1	67	******	******
#DIV/0!						1	64	107	6.6
#DIV/0!						1	85	143	5.7
23.5					男性		88	139	7
#DIV/0!						1	82	145	7.4
#DIV/0!					男性		80	288	6.9
19.4	30	0				1	73	128	6.5
23.1					男性		75	193	7.3
#DIV/0!					男性		77	159	7.3
19.6	36	0			男性		70	117	5.6
22.9	35	1				1	82	148	7
#DIV/0!						1	78	165	10
#DIV/0!						1	60	202	7.5
#DIV/0!						1	74	167	7.6
#DIV/0!						1	92	******	******
#DIV/0!						1	60	152	6.5
#DIV/0!						1	79	131	6.2
24.4						1	75	89	7.4
#DIV/0!						1	40	160	7
#DIV/0!						1	68	171	6.4
#DIV/0!						1	84	191	6.7
#DIV/0!						1	75	162	8.1
#DIV/0!						1	65	177	8.1
21.1	30	0				1	80	156	5.3
49.9						1	75	150	6.4
#DIV/0!						1	57	176	7
22.0					男性		76	146	6.2
#DIV/0!						1	69	135	6.9
22.1	30	0				1	72	121	6.1
27.6	19	0				1	66	173	6.7
25.9	16	1	0	0		1	78	128	6.7
28.2	29	1	2	0		1	69	150	7.2
20.6						1	75	135	7.9
33.3	30	0				1	75	******	******
#DIV/0!						1	64	125	6.4
#DIV/0!					男性		63	160	7.1
22.6						1	78	111	6.8
#DIV/0!						1	66	******	******
#DIV/0!						1	67	161	6.4
#DIV/0!						1	35	160	7.2

#DIV/0!				1	74	171	8
26.2				1	71	117	6.7
#DIV/0!				1	82	162	7.4
#DIV/0!				1	63	132	6.9
27.5				1	93	169	6.6
#DIV/0!				1	78	168	6.5
25.9				1	81	133	5.7
#DIV/0!				男性	74	179	6
#DIV/0!				1	64	192	7.2
#DIV/0!				1	66	165	7.9
#DIV/0!				1	72	134	7
24.5	5	0		1	64	169	10.1
19.4				1	80	155	6.6
25.0	7	0		1	65	137	6.6
#DIV/0!				1	57	193	6.2
#DIV/0!				1	58	301	9
#DIV/0!				1	65	139	6.5
31.2				1	82	97	6.2
21.8	26	0		1	65	172	6.7
#DIV/0!				1	78	105	6
17.1	27	1		1	73	134	6.3
#DIV/0!				1	72	112	6.3
26.5	9	0		1	61	120	6.6
25.6				1	84	166	8.7
#DIV/0!				1	79	140	5.3
25.1				1	87	185	7.4
#DIV/0!				1	73	118	7.7
21.8	27	1		1	83	96	6.9
#DIV/0!				1	72	100	7.4
#DIV/0!				1	84	98	5.8
22.4	26	1		1	69	162	7.3
24.6	12	0		1	60	134	6.4
#DIV/0!				1	70	146	6.7
17.4				1	79	120	5.6
#DIV/0!				1	71	210	6.6
27.8				1	67	129	5.8
19.8				1	61	146	6.7
27.0				1	77	175	6.7
#DIV/0!				1	77	123	6.6
#DIV/0!				1	87	125	7.8
#DIV/0!				1	56	125	6.7
#DIV/0!				1	77	110	4.6
#DIV/0!				1	72	206	6.7

#DIV/0!			1	57	137	6.4
19.7	25	1	1	71	95	7.3
#DIV/0!			1	69	137	7
#DIV/0!			1	54	274	9.3
22.0	18	0	1	62	199	7.9
#DIV/0!			1	72	136	7.4
#DIV/0!			1	80	133	6.6
26.4			1	73	104	5.9
#DIV/0!			1	67	155	7.1
#DIV/0!			1	53	123	5.9
27.7	5		1	67	149	7.4
24.7			1	80	127	7.3
#DIV/0!			1	78	114	6.1
25.6	10	0	男性	72	216	7.3
#DIV/0!			1	70	173	7.2
#DIV/0!			男性	72	236	6.8
17.3			1	78	142	6.8
29.2	21	0	1	77	146	7
36.5			1	36	104	5.9
24.9			1	73	189	7.8
20.6			1	69	95	6.5
#DIV/0!			1	84	141	6.1
#DIV/0!			男性	65	183	7.4
23.6	20	0	1	75	127	8
#DIV/0!			男性	68	103	6.1
19.0			1	72	278	7.1
#DIV/0!			男性	85	154	6.3
18.4	17	0	1	83	136	7
19.8	17	1	1	73	99	8.5
28.0	10	1	男性	64	183	8.2
27.0	20	0	男性	81	135	7
#DIV/0!			1	72	158	7
#DIV/0!			1	57	184	8.8
#DIV/0!			男性	82	127	6.6
25.9			1	77	139	5.6
#DIV/0!			男性	45	229	9.2
#DIV/0!			1	74	163	8.1
#DIV/0!			男性	73	124	7.3
#DIV/0!			男性	66	129	5.9
#DIV/0!			1	64	132	6.4
#DIV/0!			男性	63	140	6.6
25.2	19	1	男性	77	166	7.6

29.1					男性	75		
21.0	30	0			男性	66	124	6.5
21.4	14	0			男性	66	117	5.8
23.9	19	0			1	77	147	7
#DIV/0!					1	67	174	7.5
#DIV/0!					1	75	118	6.1
28.0	12	1	1	1	男性	66	140	6.8
#DIV/0!					1	58	100	7.6
21.6					1	73	168	6.3
#DIV/0!					1	69	154	7.2
#DIV/0!					1	71	190	7.6
26.9					男性	79	331	9
19.5	18	1			1	64	194	7.2
25.1					1	52	112	6.8
24.2					1	78	207	******
19.9	2	0			男性	76	148	6.7
#DIV/0!					1	63	118	6.9
23.2					1	78	138	6.2
#DIV/0!					男性	71	142	6.6
#DIV/0!					1	71	205	8.7
19.3	13	0			男性	70	83	6.9
19.4					1	67	******	******
20.3					男性	71	140	6.8
27.8	8	0			1	82	136	7
#DIV/0!					1	66	132	6.7
25.0					男性	62	119	4.9
25.3					1	71	144	6.6
#DIV/0!					男性	67	136	6.7
#DIV/0!					1	53	112	8.3
25.9	16	0			男性	78	217	5.9
23.6					男性	65	153	7.2
#DIV/0!					1	62	130	6.9
26.7	17	1			男性	64	140	
27.9					1	80	175	7.8
28.3	13	1			男性	73	164	7.7
22.4	15	0			1	70	144	7.3
#DIV/0!					1	71	179	7.6
#DIV/0!					男性	69	90	7.5
#DIV/0!					1	84	193	6
#DIV/0!					1	56	193	8
21.3					男性	63	119	6.7
22.0	16	0			1	71	126	5.6

#DIV/0!					男性	61	121	7.8
22.9	16	0			1	85	124	6.5
25.4					1	76	156	7.1
24.0					1	64	157	7.5
#DIV/0!					男性	49	124	6.9
#DIV/0!					1	53	180	6.8
#DIV/0!					1	63	272	8.1
23.2					1	71	181	6.3
#DIV/0!					男性	48	139	6.5
#DIV/0!					男性	66	137	5.8
#DIV/0!					1	81	******	5.7
24.4					男性	81	144	6.6
24.2	16	0	0	0	1	43	142	6.9
#DIV/0!					男性	71	145	6.6
25.5	15	1	2	0	1	58	123	6.9
#DIV/0!					1	62	127	7.1
#DIV/0!					男性	70	152	6.5
#DIV/0!					男性	77	134	11.9
28.8					1	52	214	7.3
#DIV/0!					1	62	117	6.5
#DIV/0!					1	54	128	8
#DIV/0!					1	64	118	6.6
#DIV/0!					1	75	132	6.5
#DIV/0!					1	77	217	6.6
#DIV/0!					男性	68	158	7.4
24.0					男性	73	170	6.5
#DIV/0!					1	76	102	5.7
#DIV/0!					1	69	174	6.9
#DIV/0!					1	78	124	7.2
#DIV/0!					1	83	106	5.2
30.3					1	60	105	6.5
21.8	14	0			1	63	164	6.6
20.7	8	1			男性	68	162	6.7
#DIV/0!					男性	69	125	6.5
#DIV/0!					男性	62	179	7.8
#DIV/0!					1	42	182	9.2
#DIV/0!					男性	73	151	6.6
22.0					1	78	123	7
#DIV/0!					男性	61	138	6.7
#DIV/0!					男性	68	204	6.7
#DIV/0!					1	80	141	6.2
26.7	14	1			1	76	135	8

26.4	5	0			1	64	126	6.5
#DIV/0!					1	65	188	6
#DIV/0!					1	73	171	7.3
20.1					男性	60		
27.0					男性	60	148	6.9
19.0	14	0			1	61	135	6.8
22.8					1	64	126	6.6
#DIV/0!					1	76	124	6.2
18.8	13	0			1	62	119	6.9
24.7					1	69	159	7.3
32.4	13	0			1	62	142	7.3
#DIV/0!					男性	63	151	6.5
#DIV/0!					1	70	108	5.8
#DIV/0!					1	59	128	6.1
23.8	16	1			男性	66	154	6.9
#DIV/0!					1	60	212	7.4
24.5	13	0			1	70	138	6.4
21.4					1	76	174	8.9
23.7					1	56	171	6.8
23.5	13				1	72	136	7
#DIV/0!					男性	60	149	7.6
#DIV/0!					1	68	122	5.5
#DIV/0!					男性	74	134	6.7
28.7	13	1			1	52	131	6.7
#DIV/0!					1	68	91	5.8
19.8	24	1			男性	72	149	7.3
21.4	24	0			1	63	123	8.2
#DIV/0!					男性	65	186	6.7
#DIV/0!					1	70	143	6
#DIV/0!					男性	68	119	6.6
#DIV/0!					男性	78	212	6.2
18.6					1	80	234	6.5
17.1					1	59	150	6.6
#DIV/0!					1	77	150	7
#DIV/0!					1	81	102	6.8
20.7	12	0			1	63	133	6.2
#DIV/0!					1	71	137	6.8
#DIV/0!					1	49	136	6.7
#DIV/0!					男性	78	111	6.7
#DIV/0!					1	62	134	6.5
24.6	8	0	0	1	男性	75	262	10.1
23.5	12	1			男性	81	136	7

20.4	12	0	1	96	223	8.5
#DIV/0!			1	77	167	6.6
24.1			1	69	121	7
36.7			1	82	146	7.1
#DIV/0!			1	61	140	6.8
#DIV/0!			1	62	142	7.1
#DIV/0!			1	73	169	6.9
#DIV/0!			1	63	137	6.3
#DIV/0!			1	73	138	6.3
#DIV/0!			男性	52	147	6
#DIV/0!			1	69	185	7.1
26.8	6	0	男性	71	181	6.9
20.4	11	0	1	67	146	6.1
#DIV/0!			男性	60	157	6.9
21.2	11	1	1	63	114	5.3
#DIV/0!			男性	64	155	6.5
18.1	12	0	1	78	144	6.5
#DIV/0!			男性	48	182	7.2
34.5	22	1	男性	52	193	7.1
29.9			1	67	120	6.6
#DIV/0!			1	67	97	5.9
#DIV/0!			男性	69	146	6.7
#DIV/0!			男性	69	143	6.6
21.3	9	1	1	72	128	7.1
#DIV/0!			1	61	157	6.5
#DIV/0!			1	55	398	10.7
25.3	10	0	1	65	175	7.9
24.1			1	55	162	8.5
#DIV/0!			男性	74	151	6.9
#DIV/0!			男性	74	142	6.7
#DIV/0!			1	71	140	10.2
23.1			男性	62	138	5.3
#DIV/0!			1	71	145	7.8
#DIV/0!			男性	67	124	6.1
#DIV/0!			男性	88	240	6.1
#DIV/0!			1	66	******	6.2
41.4			1	52	155	8.7
#DIV/0!			男性	65	143	5.9
25.0			男性	63	176	6.4
#DIV/0!			1	78	166	8.5
#DIV/0!			1	61	160	6.5
#DIV/0!			男性	66	134	7.2

33.7			1	74	159	7.3
#DIV/0!			1	65	163	6.7
#DIV/0!			男性	53	125	7.2
#DIV/0!			1	85	277	6.1
#DIV/0!			1	68	125	7
33.0	9	0	男性	84	142	7.8
29.7			男性	65	143	6.6
#DIV/0!			男性	72	95	7
25.6	7	0	1	64	156	6.9
#DIV/0!			1	63	173	7.4
22.1	31	0	1	72	121	7.5
#DIV/0!			1	60	184	6.8
25.1	9	0	男性	75	150	6.6
28.1	9	0	男性	65	118	7.1
#DIV/0!			男性	61	190	7.1
19.7			1	67	105	6.5
24.3			1	58	125	6.1
#DIV/0!			1	49	170	6.8
26.5			男性	69	134	6.7
#DIV/0!			男性	70	127	6.5
#DIV/0!			1	65	142	6.6
24.5			1	59	179	7.7
#DIV/0!			1	75	154	6.8
#DIV/0!			1	67	157	6.8
24.4	11	0	1	68	183	7.7
26.3			男性	49	163	7.3
#DIV/0!			男性	44	128	8
24.5	12	0	1	69	268	7.1
34.7	8	1	1	41	123	7.6
#DIV/0!			男性	60	118	6.7
#DIV/0!			男性	74	152	6.5
#DIV/0!			1	65	101	5.7
25.0	8	0	1	61	110	7.4
#DIV/0!			男性	61	148	7.2
#DIV/0!			1	64	129	6.9
#DIV/0!			1	69	138	6.6
#DIV/0!			男性	77	271	6.6
#DIV/0!			男性	66	205	7.3
#DIV/0!			1	70	120	6
#DIV/0!			1	58	111	7
#DIV/0!			1	73	160	8.2
#DIV/0!			男性	64	121	6.6

#DIV/0!			1	69	******	******
#DIV/0!			1	44	143	6.8
#DIV/0!			1	73	129	6.1
27.0			1	86	149	7.3
#DIV/0!			1	67	151	6.9
#DIV/0!			1	69	164	6.7
#DIV/0!			1	60	266	7.6
#DIV/0!			1	79	159	6.8
#DIV/0!			1	59	152	6.6
#DIV/0!			1	62	136	7.3
#DIV/0!			男性	79	135	6.4
#DIV/0!			1	63	137	7.2
#DIV/0!			1	73	159	7.6
#DIV/0!			1	77	108	6.4
#DIV/0!			1	48	149	7.2
#DIV/0!			1	56	153	6.2
24.5	30	0	1	63	169	7.2
#DIV/0!			男性	54	160	7
#DIV/0!			1	75	182	7.6
19.9			1	54	143	6.2
26.8	5	0	1	64	168	7
#DIV/0!			1	51	128	6.4
#DIV/0!			男性	42	115	4.4
#DIV/0!			1	62	195	6.4
#DIV/0!			1	62	146	7.3
#DIV/0!			1	62	******	******
#DIV/0!			1	55	171	8.5
#DIV/0!			1	71	118	7
23.8			1	62	121	6.3
#DIV/0!			1	50	124	6.2
#DIV/0!			1	61	192	8
16.7			男性	77	134	6.1
#DIV/0!			1	63	179	7.5
20.7			男性	77	118	6.5
#DIV/0!			1	60	89	6.4
#DIV/0!			男性	69	128	6.5
#DIV/0!			1	63	******	******
32.8			1	39	209	9.8
16.4	6	0	1	67	116	6.3
#DIV/0!			1	54	128	5.6
#DIV/0!			1	51	171	7.2
#DIV/0!			1	52	136	8.6
#DIV/0!			1	43	204	6.8

#DIV/0!					1	65	166	7.7
22.9					男性	58	128	6.5
#DIV/0!					1	48	103	7.1
#DIV/0!					1	57	144	6.1
#DIV/0!					男性	64	123	6.5
24.3					男性	65	141	6.4
#DIV/0!					1	68	118	6.7
22.1					1	64	106	6.9
#DIV/0!					1	68	143	6.1
#DIV/0!					1	60	171	7.8
#DIV/0!					1	53	115	6.5
31.8	8	1			1	46	103	7.5
#DIV/0!					1	59	90	6.4
25.0	15	1			1	68	150	6.5
#DIV/0!					1	45	151	7
#DIV/0!					1	79	98	6.3
#DIV/0!					1	52	179	7.8
29.0	6				1	50	209	8.9
24.3	8	0			1	64	140	7.7
#DIV/0!					1	82	121	6.7
#DIV/0!					1	62	197	7
#DIV/0!					1	50	169	6.9
25.9					男性	71	203	7.9
#DIV/0!					1	60	160	7.3
24.8					1	77	113	6.9
#DIV/0!					1	40	171	8.2
26.6	4	0			1	61	109	5.7
22.1					男性	71	174	6.5
#DIV/0!					1	70	180	7
22.8	7	1	0	0	1	61	162	7.3
23.9					男性	45	147	6.7
17.3					1	48	162	6.8
20.4					1	37	114	5
26.0	5	0			1	72	172	6.9
#DIV/0!					1	70	111	6.6
#DIV/0!					1	57	139	7.2
#DIV/0!					1	69	107	5.8
35.8					1	58	128	5.6
#DIV/0!	3		1	0	1	58	136	5.8
#DIV/0!					男性	50	221	8.1
#DIV/0!					1	54	153	6.1
24.8					1	60	158	7.3

#DIV/0!					男性	51	182	6.5
#DIV/0!					1	68	136	7.1
#DIV/0!					1	77	******	******
#DIV/0!					1	72	121	6.6
22.9					男性	70	113	6.1
22.6					1	55	120	5.5
24.7					1	54	136	6
23.7	7	0			1	42	202	7.3
26.0	5	0			1	70	158	7.2
#DIV/0!					1	63	147	6.1
29.4	3	0			1	60	140	7.1
#DIV/0!					1	50	******	******
19.7					1	80	96	5.7
#DIV/0!					1	59	146	6.1
20.8					男性	67	******	6.5
23.2	6	0			男性	54	156	7
18.4	3				1	66	******	******
0.0					1	47	188	7.2
#DIV/0!					1	84	123	7.2
#DIV/0!					1	55	180	7.4
#DIV/0!					1	73	156	6.2
#DIV/0!					男性	36	177	11.6
#DIV/0!					1	64	124	6.4
27.7					1	82	******	6
22.4	4	0	2	0	1	53	193	7.2
21.5	10		2	1	1	54	92	5.8
#DIV/0!					男性	56	110	7.2
#DIV/0!					男性	62		
#DIV/0!					1	67	156	7
#DIV/0!					1	78	256	5.8
31.8					男性	33	101	5.7
#DIV/0!					1	69	******	******
#DIV/0!					1	61	139	7.8
40.6	0.5		0	0	1	31	113	8.2
25.4	2	0			1	60	129	10.9
18.3	10		0	0	1	62	208	6.6
#DIV/0!					男性	45	171	8
21.6	20		0	0	1	78	128	5.9
28.5					男性	47	127	6
#DIV/0!					1	54	129	8.3
34.6					男性	31	148	7.5
#DIV/0!					男性	47	140	9.9

20.0					男性	67	220	6.8
#DIV/0!					男性	80	118	6.4
#DIV/0!					男性	77	170	4.9
20.1	0	0	2	0	男性	61	139	10
32.7					男性	50		6.3
29.4					男性	55	65	5.5
22.8					男性	74	122	5.6
22.0	43	1			男性	85	147	5.8
#DIV/0!					男性	67	113	6.5
20.1	12	0			1	66	200	5.5
#DIV/0!					1	52	188	8.6
34.2	5	0			1	42	125	6.8
25.4	17	0			1	66	124	6.3
#DIV/0!					1	53	141	8
23.9	12	0			1	62	148	6.1
#DIV/0!					1	63	90	5.3
27.1	3	0			1	55	148	8
#DIV/0!					1	49	135	5.9
#DIV/0!					1	54	139	6.5
#DIV/0!					1	60	109	7.9
#DIV/0!					1	70	139	7
24.4					1	62	141	6.5
#DIV/0!					男性	56	184	10.1
24.4					男性	71	119	6.2
#DIV/0!					1	75	144	6.6
21.9					1	78	100	6
22.3	26	0			1	75	133	7.6
23.3					1	79	131	6.4
#DIV/0!					1	75	132	7.1
#DIV/0!					1	69	******	******
21.3					男性	77	194	8.2
#DIV/0!					1	72	******	******
#DIV/0!					1	79	109	6.1
21.5					1	76	134	6.1
21.8	8	0		1	男性	63	152	
20.1	13	1			男性	81	132	6.7
20.8					1	78	117	7.5
17.5					1	78	144	7
#DIV/0!					1	78	******	******
23.9					男性	80	97	6.6
23.7					1	72	302	8.1

25.8	25	0	1	78	152	6.5
#DIV/0!			1	73	107	6.5
18.3	26	0	1	77	170	7.2
21.5			1	85	298	7.7
#DIV/0!			男性	50	164	7.1
27.8			1	70	138	8
19.2	26	0	1	77	128	7.6
#DIV/0!			1	58	128	6.7
19.2	20	0	1	70	161	7.6
21.6			1	75	113	6.5
31.8			1	56	139	6.2
20.7	21	0	1	73	146	7.9
24.0			1	68	165	7.4
22.9			男性	70	133	6.4
25.9	22	0	男性	71	105	5.8
19.2	6	1	1	73	120	6.7
30.1	19	1	1	59	214	6.9
26.7			男性	72	145	6.5
25.6	19	1	1	75	115	6.5
23.0			1	72	147	6.9
21.5			1	69	149	5.4
16.7			1	86	127	6.1
#DIV/0!			1	78	178	7.6
24.4	17	1	1	61	133	7
#DIV/0!			1	72	279	9.9
21.8			1	72	172	8
#DIV/0!			1	72	123	5.9
19.2			男性	80	182	6.5
#DIV/0!			1	64	161	8.2
24.8			男性	82	171	7.3
21.5	30	0	男性	82	155	7
#DIV/0!			1	74	170	5.8
22.4			男性	72	113	8.1
26.1	3	0	1	70	143	6.5
23.6			男性	62	146	5.6
24.7			1	72	190	8.2
27.2	15	1	1	75	147	7
25.3			1	63	147	7.5
#DIV/0!			1	79	236	11.5
#DIV/0!			1	59	151	6.6
25.4			1	79	201	5.8
25.6	13	0	1	66	157	7.3

20.1					1	65	166	6.9
25.7					1	71	151	7.1
#DIV/0!					1	68	75	5.3
#DIV/0!					男性	48	174	7.2
#DIV/0!					男性	78	129	5.9
27.1					男性	56	88	8.4
21.5					1	76	112	5.8
20.7					男性	64	115	6.5
#DIV/0!					1	74	146	6.9
22.1					1	71	109	6.9
#DIV/0!					1	77	******	7.2
#DIV/0!					1	68	175	6.4
23.8	8	0			1	62	188	6.8
28.7					1	69	135	6.9
23.1					男性	87	131	6.8
25.8	15	1			1	71	145	6.5
29.0					男性	63	150	7
24.3					1	59	130	6.4
#DIV/0!					1	63	117	7.2
#DIV/0!					1	65	147	7.2
18.4					1	64	165	6.7
23.0	7	0			1	88	134	6.7
26.0	4	0			1	64	166	7.2
29.4	6	0			1	72	172	7.2
#DIV/0!					1	57	196	6.9
29.0	5	0			1	84	109	6.1
24.4	4	0			1	73	130	6.1
#DIV/0!					1	66	103	7.1
#DIV/0!					1	64	122	6.2
23.6					1	59	215	7.2
26.1	3	0			男性	66	262	7.1
22.2	20	0			1	82	102	6.5
#DIV/0!					1	62	119	5.8
17.9	2		0	0	1	75	141	6.7
22.3	1	0			1	67	120	6
#DIV/0!					1	57	114	7.1
29.4					男性	68	125	7.1
27.2					男性	44	229	******
23.6					1	63	146	5.9
#DIV/0!					男性	63	101	6.6
#DIV/0!					1	68	109	******
25.2	7	0			1	74	137	6.5
#DIV/0!					1	44	******	******

#DIV/0!			1	73	149	7.2
#DIV/0!			1	75	162	7.1
#DIV/0!			1	85	194	7.2
21.9			1	77	132	6.6
#DIV/0!			男性	57	139	7.9
#DIV/0!			男性	78	135	6.6
21.6			男性	78	116	6.9
25.1			1	79	198	7.8
22.8	16	0	1	79	123	5.8
26.2	35	0	1	84	186	7.1
21.3	26	0	1	66	94	6.7
24.0			1	66	166	6.8
22.4			1	63	136	5.9
24.4	19	0	1	70	225	6
23.4	12	0	1	64	131	6.8
22.8	19	0	1	69	110	6.7
#DIV/0!			1	79	133	7.7
20.4			1	71	151	7.4
27.5			1	66	149	7.1
#DIV/0!			1	64	129	6.2
#DIV/0!			男性	60	106	6.1
29.7	12	0	1	60	156	6.5
#DIV/0!			1	74	141	6.9
22.5			男性	67	164	7.4
28.5			1	73	116	6.2
#DIV/0!			1	86	105	6.2
27.0	5	0	1	63	126	8.6
24.3			1	63	217	8.5
27.9			男性	71	162	7.4
23.4	18	0	1	64	174	7.2
#DIV/0!			1	69	85	5.9
#DIV/0!			1	51	152	6.3
25.8			1	79	123	7.4
20.6			1	93	156	6.5
#DIV/0!			男性	64	144	6.9
18.9			1	63	154	6.7
25.2	5	0	1	65	186	7.3
#DIV/0!			男性	80	310	4.9
#DIV/0!			1	60	150	6.6
#DIV/0!			1	74	109	6.7
21.5			男性	73	130	6.3
#DIV/0!			1	64	167	6.7

#DIV/0!			1	59	166	6.6
24.1			1	63	144	6.8
#DIV/0!			男性	67	161	5
20.4			男性	77	101	6.7
23.6			1	66	199	6.9
#DIV/0!			1	70	177	6.7
#DIV/0!			1	82	125	6.4
19.2			1	74	******	6.2
23.4	14	1	1	77	165	8.7
23.3			1	78	155	6
26.3			男性	64	251	8
#DIV/0!			1	52	143	6
24.8	5	1	1	71	153	7
#DIV/0!			男性	46	139	7
#DIV/0!			男性	56	183	6.8
20.8			1	75	182	8
20.1			男性	71	231	7.3
21.6	3	0	1	60	105	5.5
#DIV/0!			1	68	226	9.6
28.7			1	75	136	6.5
#DIV/0!			1	59	143	7.1
#DIV/0!			1	79	194	6.8
#DIV/0!			1	66	132	7.4
#DIV/0!			1	58	176	7.4
#DIV/0!			男性	79	108	6
#DIV/0!			男性	60	113	6.1
#DIV/0!			男性	67	158	7.3
22.9	31	0	1	71	136	6.4
20.4			男性	80	97	6.9
#DIV/0!			1	84	******	5.5
19.3			男性	77	199	******
#DIV/0!			1	70	121	6.8
22.0			1	66	104	6.3
24.1	20	0	1	63	107	5.8
#DIV/0!			1	71	132	5.3
#DIV/0!			1	64	242	9.4
#DIV/0!			1	57	282	******
#DIV/0!			1	55	172	6.6
#DIV/0!			1	54	119	6.4
21.1	8	1	男性	68	101	6.3
26.1			1	62	87	6.5
#DIV/0!			1	65	113	5.4

#DIV/0!					1	67	130	6.2
#DIV/0!					1	65	102	5.6
20.3					男性	74	110	6
23.3	5	0			1	63	153	10
19.2	3	1	0	0	1	66	113	6
#DIV/0!					1	69	105	6
#DIV/0!					1	63	202	5.8
24.7			0	0	1	76	110	6.1
#DIV/0!					男性	64	127	5.5
22.0	3		2	1	1	67	157	7.9
27.4					男性	41	******	******
21.5	2	1			男性	64	290	9.6
28.6	1	0			男性	63	384	8.2
#DIV/0!					1	58	******	******
22.9					男性	73	219	6.6
#DIV/0!	3		0	0	1	65	135	6.4
#DIV/0!					1	75	151	6.5
22.2	35	1			1	76	114	7.7
22.0					1	81	138	5.8
24.7					男性	78	227	7.3
26.1	10	0			1	68	114	5.6
20.3					男性	77	135	6.7
19.8					1	74	91	******
#DIV/0!					1	59	110	5.2
17.6	26	0			1	73	130	6.9
21.3					1	83	142	6.3
18.1	12	0			1	65	90	6.5
18.9					男性	71	266	7.9
#DIV/0!					1	79	124	6.4
25.7	11	0			男性	75	123	6.8
#DIV/0!					1	76	137	5.5
31.2	6	1			1	67	114	6.8
20.1					1	80	311	8.4
#DIV/0!					男性	72	154	6.7
23.7					1	77	123	6.7
27.0					1	75	136	7.3
#DIV/0!					男性	81	98	5.1
#DIV/0!					1	54	124	6.4
27.4					1	70	255	7.4
26.6					男性	73	151	7
25.5					男性	78	112	6.5
24.0					1	78	133	7.3

22.2					男性	73	149	6.5
#DIV/0!					1	75	134	6.6
26.0	15				1	71	121	6.8
#DIV/0!					1	58	143	7.2
23.8	12	1			男性	88	124	7.2
21.2	7	0			1	86	207	6.8
#DIV/0!					1	59	151	6.9
25.1					男性	58	147	6.2
23.6					1	67	190	7.1
23.1					男性	70	250	7.5
20.6	5	0			1	76	115	4.9
#DIV/0!					男性	49	153	7.2
23.4	9	1			1	63	178	6.5
22.5					男性	77	145	6.1
#DIV/0!					男性	72	137	6.3
20.0					1	77	149	6
26.1					1	75	106	6.9
#DIV/0!					1	68	******	******
#DIV/0!					1	74	137	5.1
30.7					男性	47	145	6.8
#DIV/0!					1	57	152	6.8
#DIV/0!					1	75	137	5.8
20.7					男性	71	******	******
#DIV/0!					1	75	185	6.4
#DIV/0!					1	67	225	8.8
26.4	3	1	0	1	1	65	91	5.9
25.8	3		0	1	1	68	171	7.9
#DIV/0!					1	81	113	6.3
#DIV/0!					1	83	******	******
22.6	0				男性	81	153	14.1
#DIV/0!					男性	78	239	4.6
30.3					男性	86	236	6.1
#DIV/0!					男性	65	120	5.2
27.7	3	0			1	60	133	5.5
20.6					1	73	160	5.9
#DIV/0!					男性	78	139	6.3
27.5					男性	57	124	8.2
20.6					1	79	******	******
#DIV/0!					1	52	165	7.5
#DIV/0!					1	77	137	6.5
#DIV/0!					1	63	147	6.1
24.4					男性	88	******	******

21.5	1	65	141	8.6
20.0	1	69	206	8.3
#DIV/0!	1	80	269	8
#DIV/0!	男性	54	189	8.7
#DIV/0!	男性	47	123	6.5
#DIV/0!	1	79	138	6.4
#DIV/0!	1	85	141	7.2
#DIV/0!	1	68	162	4.7
25.4	男性	73	305	7
22.2	男性	75	273	6.1
22.3	1	72	213	7.6
20.6	1	84	142	6.4
#DIV/0!	男性	64	120	7.3
#DIV/0!	1	64	164	8.5
#DIV/0!	男性	57	147	5.9
#DIV/0!	1	61	121	6.8
#DIV/0!	1	77	172	8.9
#DIV/0!	1	71	211	6.3
#DIV/0!	男性	69	134	6.5
#DIV/0!	男性	71	137	7.1
#DIV/0!	1	87	165	7.2
#DIV/0!	1	67	128	6.2
#DIV/0!	1	48	172	7.1
#DIV/0!	男性	68	222	7.6
#DIV/0!	男性	68	142	5.4
#DIV/0!	男性	60	105	7.3
#DIV/0!	男性	56	149	7.8
#DIV/0!	男性	82	156	6.2
#DIV/0!	1	53	160	6
#DIV/0!	男性	41	147	8
#DIV/0!	男性	78	153	7.7
32.6	男性	52	142	6.9
#DIV/0!	1	76	155	6.4
#DIV/0!	男性	53	264	7.2
#DIV/0!	男性	80	120	6.2
#DIV/0!	男性	64	167	
#DIV/0!	男性	74	127	6.4
#DIV/0!	男性	77	160	7.6
#DIV/0!	男性	64	107	6.4
18.3	1	75	101	7
#DIV/0!	1	78	160	7.6

#DIV/0!	1	60	127	7.2
23.0	男性	67	134	7.2
18.8	男性	69	112	6
#DIV/0!	男性	71	106	6.2
22.0	男性	96	110	6.2
#DIV/0!	1	73	101	6.7
#DIV/0!	男性	59	214	7.5
#DIV/0!	男性	71	113	6.7
20.5	男性	64	200	9.5
#DIV/0!	男性	73	131	6
#DIV/0!	1	73	142	7
31.2	1	59	111	6.1
18.9	1	72	99	5.4
#DIV/0!	1	67	185	8.1
23.1	男性	67	163	7.1
#DIV/0!	男性	41	134	6.7
#DIV/0!	1	63	124	6.4
#DIV/0!	1	66	152	6.7
#DIV/0!	1	63	121	6.8
#DIV/0!	1	64	136	6.5
#DIV/0!	1	67	134	6.1
#DIV/0!	男性	58	143	5.9
#DIV/0!	1	64	104	6.6
#DIV/0!	1	57	223	7.5
#DIV/0!	1	53	196	8.3
21.6	男性	55	135	6.5
#DIV/0!	1	44	151	7.3
21.9	1	79	97	6.6
#DIV/0!	1	59	169	7.3
#DIV/0!	1	43	156	7.8
#DIV/0!	1	80	132	6.7
#DIV/0!	1	52	184	7.2
#DIV/0!	男性	63	148	7.3
24.0	1	66	144	6.3
#DIV/0!	1	71	139	5.7
23.0	1	68	136	6.3
24.3	1	71	218	6.3
#DIV/0!	1	72	182	6.5
#DIV/0!	男性	50	118	5.6
22.6	1	66	124	6.1
#DIV/0!	1	47	157	7.2
#DIV/0!	男性	75	203	7.4

24.2					1	78	180	7.4
18.3					男性	70	160	5.7
#DIV/0!					1	39	142	7.2
#DIV/0!					1	55	188	6
22.2					男性	47	136	5.8
24.2					1	63	128	6.2
26.8					男性	52	95	7.2
#DIV/0!					1	29	134	6.2
#DIV/0!					1	51	166	8.9
#DIV/0!					男性	57	120	5.8
23.5					男性	69	129	6.6
25.1	13	1			男性	49	243	8
#DIV/0!					1	38	180	7.4
#DIV/0!					1	58	183	6.6
#DIV/0!					男性	69	173	6.6
#DIV/0!					男性	66	188	9.3
37.3	10		2	0	1	47	148	7.3
#DIV/0!					男性	63	196	8
#DIV/0!					1	55	152	******
22.4					1	74	105	7.2
#DIV/0!					1	82	104	5.5
#DIV/0!					男性	84	176	6.8
#DIV/0!					男性	81	153	7.5
#DIV/0!					男性	78	161	7.4
24.8					1	72	145	7.7
#DIV/0!					1	69	124	6.2
21.5					1	63	100	6.1
#DIV/0!					男性	76	168	6.6
25.7					1	74	114	6.3
23.7					男性	64	172	6.6
#DIV/0!					1	79	126	7.5
23.9					1	78	105	7.5
27.6					1	69	161	7.3
19.2					1	74	130	6.2
#DIV/0!					1	74	159	6.5
#DIV/0!					1	78	103	5.6
18.8					男性	70	120	5.8
22.6					男性	60	243	6.9
#DIV/0!					男性	65	212	******
29.4					男性	50	444	11.1
23.9					1	66	125	6.9
27.6					1	79	153	6.8

22.9				1	72	179	6.3
25.8				1	79	116	5.9
#DIV/0!				1	59	240	8.5
#DIV/0!				1	70	121	6.4
24.7				1	66	63	5.8
#DIV/0!				男性	57	129	6
25.7				1	78	110	5.7
#DIV/0!				男性	66	124	5.7
18.9				男性	71	153	6.9
#DIV/0!				男性	72	161	6.6
#DIV/0!				1	79	215	8
#DIV/0!				男性	65	147	6.4
30.4				男性	53	160	7.3
#DIV/0!				男性	60	125	6.4
#DIV/0!				1	73	174	6.6
24.2				男性	57	143	7.4
22.8				男性	65	116	6.1
#DIV/0!				男性	64	122	5.8
#DIV/0!				男性	75	128	5.8
#DIV/0!				男性	70	183	6.6
21.2	12	0	1	1	75	203	7.6
#DIV/0!				1	63	148	6.1
37.6				男性	46	122	7.7
#DIV/0!				男性	30	124	4.5
#DIV/0!				男性	82	106	6
27.3				1	63	99	6.1
21.1				男性	85	161	6.5
#DIV/0!				1	79	132	5.5
31.9				1	74	150	6.3
26.0				1	78	121	5.8
23.1				1	65	134	5.8
#DIV/0!				1	69	148	7.3
#DIV/0!				1	76	193	7.5
#DIV/0!				1	73	141	6.8
#DIV/0!				男性	73	129	6.7
#DIV/0!				1	80	147	6.7
23.1				1	64	158	7.5
21.9				男性	79	112	6.3
16.6				1	73	156	6.9
23.0				男性	78	160	6.8
25.0				1	66	150	6.9

24.2	30		2	1	男性	72	151	6.2
18.6					男性	60	96	5.8
#DIV/0!					1	66	107	6.1
20.2					1	67	137	7.3
19.6					男性	67	104	
#DIV/0!					1	60	185	9.6
20.9					男性	63	102	6.3
29.1	2	0			男性	47	190	8.3
#DIV/0!					男性	64	150	7.4
#DIV/0!					男性	78	163	7.5
22.4	12	0			1	45	127	5.1
26.2	15	0			1	66	135	7
21.4	25	0			男性	62	118	6
21.8	10	0			1	64	134	6.3
19.6	22	1	0	0	男性	73	144	7.6
24.6	8	0			男性	70	119	6
23.3	17	0	0	0	1	54	147	6.8
25.4	17	0			男性	71	174	7.3
22.0	7	0			男性	73	205	6.4
#DIV/0!					男性	57	158	7.3
26.5	20	1			1	71	145	7.2
22.9	3				1	77	177	7
20.6	6	0			男性	71	121	6
26.5	16	0			男性	42	220	8.7
30.9	6	0			男性	65	195	7.7
21.1	12	1	2	0	男性	62	162	7.7
19.0	10	0			男性	69	130	6.1
24.1	17	1			男性	63	93	6.2
23.8	11	0			1	72	138	6
22.4	7	0			男性	64	157	6.9
41.3	12	1			男性	49	111	8.1
25.6	5	0	2	1	1	61	95	5.8
#DIV/0!					1	54	119	7.2
19.3	4	0	0	1	1	64	140	6.4
23.0					1	72	132	6.8
32.2	8	0	1	0	男性	57	174	7.3
#DIV/0!					1	51	144	5.8
#DIV/0!					男性	67	******	6.4
#DIV/0!					1	68	132	6
#DIV/0!					男性	78	129	6.4
#DIV/0!					男性	40	153	7.2

#DIV/0!					1	49	325	
#DIV/0!			0	0	男性	69	151	6.6
25.7					男性	46	153	6.4
18.6	20	1	2	0	1	44	329	7.5
25.3	15	1			男性	79	136	6.1
30.5	25	0			男性	75	135	7.2
20.7	18.0				1	72	111	6.5
24.8	22	1	0	1	1	70	157	7.2
#DIV/0!	3	0			男性	46	205	7.9
27.1	4	0	1	1	1	67	166	7.1
20.8					男性	69	117	5.8
0.0	3		1	0	男性	69	110	
20.4					1	81	206	7.9
18.9	18	0			男性	64	131	5.9
18.8	26	0			1	78	163	6.6
24.0	28	0			男性	72	184	7.6
18.5	17	0			1	76	152	7.2
19.8	13	1			男性	67	97	6.3
25.0	2	0			男性	55	113	5.9
24.8	20	0			1	60	141	5.5
#DIV/0!	2		1	0	男性	42	96	5.7
20.9					1	68	186	6
20.8	30	1			男性	78	202	8.8
21.4	14		0	0	男性	60	102	5.7
24.0	5		1	0	男性	64	195	8.3
28.0					1	66	158	6.5
18.1					1	67	125	5.7
#DIV/0!					男性	81	362	9.4
#DIV/0!					男性	84	106	7.4
#DIV/0!					男性	77	219	8
22.9					男性	73	212	6.6
19.4	40	1			男性	78	207	6.2
18.2					1	78	139	6.8
#DIV/0!					男性	62	145	6.5
20.6	13	1			1	52	449	12.1
#DIV/0!					1	77	217	6.5
19.8	40	1	0	0	1	75	154	6.6
23.7					男性	76	165	9.4
#DIV/0!					1	51	96	6.9
21.1	24	0			1	48	106	5.7
17.9	20	1			男性	46	177	7.1
35.2	18	1			1	41	168	8.6

21.9	18	0			1	47	238	7
24.9	15	1			1	65	109	8.3
26.2	9	0			男性	45	167	8.2
20.7	13	0			1	60	52	6.8
19.8	10	0			1	39	216	7.7
21.2	13	0			男性	40	220	8.3
16.6	9	0			1	43	123	7.3
21.6	6	0			1	47	89	6.1
21.3	7	0			1	54	80	7.4
26.6					1	36	281	9.7
21.5	5	0			1	43	165	6.7
24.2					1	75	144	9.8
21.8					男性	76	101	7.9
14.6	24	1			1	65	423	11.5
23.2	19	0			1	60	234	7
29.1					男性	79	147	7.2
23.1					1	54	243	6.8
19.6	12	0			1	42	173	6.3
19.8	35	1			男性	68	105	7.4
20.1					1	72	193	7.3
20.5					男性	75	135	7.9
23.6	20		0	0	1	57	245	8.3
22.5					1	82	******	******
31.7					1	62	273	9.2
#DIV/0!					男性	64	******	******
20.8	38	1	0	0	男性	50	137	7.7
22.5	35	1	0	0	1	79	147	7.5
20.7	16	1			1	70	285	9
22.1	19	1			男性	46	86	9.9
21.5	17	1			1	64	146	6.3
23.0	15	1	2	0	男性	73	109	7
17.2	25	0			1	74	116	6.5
16.9					1	77	345	6.6
#DIV/0!					1	67	165	6.9
20.9	36	1			男性	63	95	6.5
24.2	24	1			1	70	81	6.9
#DIV/0!					1	71	1398	6.5
#DIV/0!					1	68	290	7.3
#DIV/0!					1	76	116	6.8
20.2	37	1			1	68	157	8
#DIV/0!					1	82	114	7
22.2	12	0			1	75	116	5.9

#DIV/0!					1	70	307	8.6
14.6	30	0			男性	78	188	7.1
#DIV/0!					1	80	288	6.9
21.1	32		0	0	男性	63	253	7.1
28.6					1	77	90	7.9
25.2	26	1			1	74	179	7.8
#DIV/0!					1	56	84	6.5
22.0	26	1			1	69	146	6.5
27.8	22	1	2		1	79	108	6
26.4	35	1			1	75	142	8
29.4	36	0			1	85	95	5.9
29.8	28	1			1	53	204	9.1
25.2					1	87	173	9.1
#DIV/0!					1	70	317	9.3
21.7					1	78	95	6.6
27.3	36	1			1	73	191	7.6
31.7	6	0			1	61	127	7
23.8					1	71	******	******
29.4	22	1			1	62	150	9.5
25.3	13	1			1	62	173	7
19.0	25	1			1	55	182	8.1
#DIV/0!					1	76	213	6.3
21.8					1	92	******	******
22.5	18	0			1	57	251	7.5
#DIV/0!					1	63	162	6.8
#DIV/0!					1	70	77	6.5
20.3	24	0			1	69	131	7.2
29.1	35	0			1	62	273	10
#DIV/0!					1	73	100	6.5
22.9					1	70	192	7.6
31.0					男性	73	175	6.5
21.6					男性	74	163	8
19.4	2	0			1	82	201	6.2
25.7	25	1			1	75	111	8.3
#DIV/0!					1	82	196	7.5
#DIV/0!					1	53	211	6.2
#DIV/0!					1	68	151	6.5
24.8	20	1			1	64	229	7.5
#DIV/0!					1	64	126	6.5
#DIV/0!					男性	66	124	6.9
24.5					1	64	153	7.2
#DIV/0!					1	71	152	5.9
#DIV/0!					1	63	104	6.1

#DIV/0!					男性	62	277	7.2
21.6	9	0			男性	81	219	7.4
21.8					男性	74	162	8.2
#DIV/0!					1	50	181	6.4
19.3	0	0			男性	80	180	6.1
21.8	10	1			1	77	160	5.9
20.4					1	75	173	7.8
#DIV/0!					男性	63	337	6.9
26.9					男性	62	124	9
#DIV/0!					男性	54		
#DIV/0!					男性	66	122	6.6
#DIV/0!					男性	77	139	6.5
#DIV/0!					1	65	139	7.1
19.3	10	1			男性	62	118	7.2
31.0					男性	78	84	6.1
#DIV/0!					1	76	295	7.4
31.5	25	0			1	69	98	6.7
#DIV/0!					1	70	198	6.6
#DIV/0!					男性	80	83	6.8
22.8	21	0	0	0	1	79	133	6.5
26.0					1	77	167	6
19.8	19	1			1	81	157	7.1
20.5					男性	84	222	7.2
#DIV/0!					1	60	136	6
#DIV/0!					1	80	179	8.6
#DIV/0!					男性	53	145	7.8
27.2	12	1			男性	74	50	7.9
#DIV/0!					男性	60	146	6.7
40.7	17	1			男性	56	186	7.2
20.1					1	66	120	6.2
23.1					1	76	109	6.7
26.2					1	65	232	9.4
21.1	17	0			1	56	174	8.9
23.3					1	67	97	6.6
#DIV/0!					男性	66	131	7.1
#DIV/0!					男性	63	162	6.7
#DIV/0!					1	66	132	7.5
21.0					男性	69	135	6.6
17.6	17	1			1	76	185	9.2
#DIV/0!					1	65	136	7.7
18.8					男性	74	98	6.8
#DIV/0!					1	68	101	6.5

#DIV/0!					1	66	115	7.6
#DIV/0!					男性	52	108	8
24.6	16	1			男性	72	149	6.9
#DIV/0!					男性	67	132	8.7
24.2	13	1			1	66	110	7.4
#DIV/0!					男性	48		
#DIV/0!					男性	62		
18.2	30	1	0	0	1	83	235	7.4
22.8	7	0			1	57	180	7
23.7	16	0			男性	67	326	8.9
#DIV/0!			0	0	1	56	******	9.8
#DIV/0!					1	81	110	6.7
29.0	15	1			1	63	92	8.7
25.5	30	1			1	66	95	10.3
37.1					男性	41	265	11
32.4	15	1			1	51	191	8.8
23.0					男性	64	146	6.4
#DIV/0!					1	71	150	7.5
#DIV/0!					1	73	229	7.9
#DIV/0!					1	46	162	8.6
22.9					1	62	235	7.7
#DIV/0!					男性	49	70	6.9
#DIV/0!					男性	56	274	9
24.7					1	68	203	5.7
21.5	14	0			1	65	182	7.5
#DIV/0!					1	74	140	7.7
25.4					1	45	199	8.6
31.8	14	0			男性	57	211	10.9
#DIV/0!					1	52	153	7.8
#DIV/0!					1	55	113	6.8
26.8	14	1			男性	47	152	7.2
23.5					1	60	307	8.6
22.9	13	1			男性	64	256	8
31.9					1	60	232	8.2
32.8	13	1			1	48	346	9.1
#DIV/0!					男性	51	56	7.6
25.3	13	1			1	74	284	8.6
27.2					1	77	207	6.7
26.6					男性	58	186	6.7
#DIV/0!					1	73	210	6.5
20.2					男性	74	225	6.5
21.4					男性	50	183	8.6

26.1			1	81	152	8.6
#DIV/0!			1	43	236	10.6
22.8	20	1	1	64	74	7.1
24.5	12	1	1	55	164	7.7
23.7			1	73	173	7.2
#DIV/0!			男性	67	169	7.7
20.8			1	64	133	7.7
#DIV/0!			1	42	103	7.5
24.1	17	1	男性	57	421	9
#DIV/0!			1	68	143	6.8
28.6			1	58	185	6.5
#DIV/0!			男性	66	102	6.5
25.9			男性	66	193	8.3
#DIV/0!			男性	65	86	8.4
#DIV/0!			1	61	186	6.6
33.0	11	1	1	54	157	6.6
37.2	11	0	1	80	123	6.4
#DIV/0!			1	64	231	6.9
26.0			1	67	176	7
32.4	13	0	男性	62	131	7.7
29.4			1	53	186	******
#DIV/0!			1	59	186	7.1
#DIV/0!			男性	48	146	9.2
26.2			男性	62	351	9.8
#DIV/0!			1	64	177	8.4
18.2			1	75	115	7
#DIV/0!			1	44	106	8.3
19.4			男性	56	164	7.3
28.2	18	1	男性	64	213	7.7
21.3			1	83	161	6.4
#DIV/0!			1	56	124	6.9
32.4			男性	70	161	6.8
27.7	17	1	男性	44	114	11.9
#DIV/0!			男性	42	94	6.3
#DIV/0!			1	21	223	7.7
24.3	9	0	1	61	159	6.6
#DIV/0!			男性	60	151	8.7
25.6	18	0	1	61	210	8.7
#DIV/0!			男性	60	206	6.9
27.4			1	59	92	7.1
#DIV/0!			男性	66	194	7.5
#DIV/0!			1	46	183	11.2

#DIV/0!					1	41	263	7.4
26.3					男性	81	144	6
17.1					男性	71	108	5.7
#DIV/0!					男性	60	241	10.7
26.8					1	82	114	5.6
25.1					1	65	170	6.6
27.3					1	40	106	9.5
#DIV/0!					男性	42	203	6.8
#DIV/0!					1	55	194	7.6
#DIV/0!					1	49	210	7.8
#DIV/0!					男性	22	95	7.9
95.7			2	0	1	77	251	5.3
20.0					1	67	96	7.1
#DIV/0!					1	64	197	7.7
27.1					1	70	79	8.4
#DIV/0!					1	85	127	6.2
#DIV/0!					1	47	326	12.1
#DIV/0!					男性	73	341	8.5
31.3	7	0			1	39	134	7.3
21.4	17	0	1	0	1	77	161	7
#DIV/0!					1	58	95	6.3
24.7					1	73	157	6.7
16.4					男性	79	319	8
25.5					1	74	121	5.4
#DIV/0!	5		0	0	1	65	155	6.6
26.9					男性	44	401	15.5
#DIV/0!					1	50	134	6.9
33.1	9	1	0	0	1	34	256	9.3
18.1					男性	69	235	7.2
#DIV/0!					1	58	259	10.3
23.0					1	71	288	6.5
27.0	7	0	0	0	1	64	217	6.3
#DIV/0!					男性	57	248	8.8
23.4	6		0	0	1	29	295	9.6
#DIV/0!					1	34	59	7.6
#DIV/0!					1	62	158	8.9
24.9					1	63	134	5.7
24.5	20		0	0	1	63	119	5.5
24.1					1	56	******	******
29.0					男性	41	236	6.7
32.4					男性	26	227	11.4
23.5					男性	50	135	6.2

32.6	2	0			男性	50	143	9.6
23.9	4	0			1	62	104	7.6
#DIV/0!					男性	49	270	9.1
#DIV/0!					男性	54	111	7.5
23.5					1	36	161	7.1
28.1	20		1	0	1	44	100	7
#DIV/0!					1	52	236	******
#DIV/0!					男性	80	102	5.3
21.7	8	0			男性	81	194	7.5
20.1					1	79	121	5.8
26.1					1	78	115	8.1
24.2			1		1	72	210	7.9
#DIV/0!					1	90	191	6.4
#DIV/0!					1	74	173	6.9
23.0					1	92	88	6.4
13.3					1	82	46	6
21.0					1	74	105	6.3
26.2					男性	79	126	7.9
25.2					1	71	188	7.4
25.9					1	67	279	7.4
25.6					1	48	132	8.1
20.1	26	1			1	89	121	5.7
22.8					1	78	229	6.5
#DIV/0!					1	67	123	7.6
29.4	22	1			1	66	129	7.6
#DIV/0!					1	69	115	7.5
23.0	19	0			1	64	136	6.8
29.5	29	1	0	0	男性	64	196	9.6
26.9					1	80	180	6.5
29.4					1	80	54	7.3
42.2					1	60	136	7.4
#DIV/0!					1	80	137	7.2
24.1					男性	74		
#DIV/0!					男性	72	223	8.5
25.4	14	0			1	50	134	5.8
26.6	19	0			男性	53	121	8.6
19.5					男性	78	147	6.4
19.5					男性	70	139	6.5
#DIV/0!					男性	65	130	8
25.3	25	1			男性	70	174	8.3
20.7					1	67	89	7.1
23.3	13	1			1	64	154	8.1

21.6	19	1			1	71	94	5.7
#DIV/0!					男性	41	82	6.1
21.5					1	81	179	7.8
24.9					1	71	123	6.6
25.1					男性	75	138	7.2
#DIV/0!					1	78	******	******
21.2					1	65	105	7.2
22.2	16	0			男性	67	154	6.9
24.3					男性	63	184	7.7
24.4	14	0			男性	69	141	6.5
#DIV/0!					1	79	276	6.5
25.9					男性	68	190	6.6
25.2	8	1			男性	78	207	7.2
25.1					男性	67	227	7.6
27.3					1	69	210	7.2
#DIV/0!					1	59	186	8.1
25.0					1	72	270	6.2
23.3	15	0			1	85	104	6.3
23.4	25	1	0	0	1	77	100	7.8
23.6					1	74	134	7.3
19.6	3	1	0	1	男性	77	100	6.7
23.1	6		2	1	1	75	272	11.2
#DIV/0!					1	59	139	6
#DIV/0!					男性	85	180	6.6
20.8					男性	81	137	7
25.6					男性	79	140	5.9
24.5	37	1			男性	67	159	7.6
20.7					男性	75	129	7.3
19.8					1	64	93	6.5
34.6					1	59	134	6.7
20.9	30	0			1	77	******	******
18.8					1	76	118	6.5
23.3	8	0			1	73	231	7.4
34.5					1	55	248	10.2
#DIV/0!					1	66	165	9.1
#DIV/0!					1	58	120	6.3
37.9					1	74	99	6.5
29.3					1	69	193	9
22.8	25	1			1	60	166	6.2
22.4					男性	78	75	4.6
29.0	26	1			1	72	173	7.7
20.3	19	0	0	0	1	69	118	6.5

26.3	19	1			1	63	132	6.6
22.4	19	0			男性	80	273	7.6
26.3					1	78	198	8.4
21.2	17	0			男性	75	107	5.9
#DIV/0!					1	59	420	7.8
30.5					男性	60	156	7.3
25.8	16	1			1	78	292	6.5
19.1					男性	85	252	7.4
21.2	20				男性	71	122	6.9
#DIV/0!					1	53	230	7.9
17.2					1	57	133	6.4
22.1	42	1			男性	81	126	7.6
22.0	12	1	2	0	1	60	131	7.4
#DIV/0!					1	69	129	7.5
24.0					男性	68	183	7
#DIV/0!					1	66	120	5.9
23.9	9	0			男性	61	185	7.6
51.6					男性	51	185	5.9
#DIV/0!					男性	71	241	8.9
#DIV/0!					1	60	101	5.9
23.0	31	1			1	76	164	6.8
24.9					1	75	160	5.9
26.1					1	81	170	6.5
#DIV/0!					1	49	122	6.8
#DIV/0!					男性	76	121	6.8
#DIV/0!					男性	75		
32.7	30		0	0	1	67	319	8.9
25.2					1	61	197	9.2
23.9					男性	52	213	******
23.5					男性	67	321	11.8
23.6	2	0	0	0	1	82	191	8.3
#DIV/0!					1	74	124	7
23.0	30	0			1	85	196	7.2
21.3					1	76	161	7.1
26.2	14	1			1	81	180	7.5
#DIV/0!					1	78	238	7.8
24.4					1	86	103	6.5
23.5	8	0			男性	75	130	7
#DIV/0!					1	72	220	8.5
#DIV/0!					1	85	130	7.7
26.4					1	84	******	6.8
24.4					1	78	194	8.8

20.7					男性	67	174	5.5
24.0					1	88	******	******
35.9					1	65	259	8
19.1	8	0			1	73	158	6.9
22.9					男性	81	172	6.4
23.1					男性	79	141	7.4
#DIV/0!					1	77	201	7.6
25.5	24	0			1	76	208	5.6
22.5					1	75	127	5.5
30.4					1	88	188	6.8
#DIV/0!					1	51	201	7.7
28.3					男性	54	280	9.8
#DIV/0!					男性	84	150	7
25.4					1	60	155	9.3
24.0					1	74	244	8.3
23.4	0	0			男性	71	139	5.8
23.5					1	79	215	7.3
32.4	7		2	1	1	48	212	6.3
#DIV/0!					男性	65	181	4.9
20.1					1	76	157	7.5
25.4					男性	74	230	7.1
30.5					1	71	181	7.1
24.0	20		1	0	1	75	164	7.5
#DIV/0!					1	70	122	6.7
#DIV/0!					1	63	112	6.4
#DIV/0!					男性	77	189	7.8
#DIV/0!					1	84	352	7.8
24.4					男性	75	259	8.7
21.4					男性	68	118	6.8
24.8					1	70	162	9.5
21.8					男性	57	171	7.9
21.1					1	62	242	12.6
21.2					1	67	96	6.5
#DIV/0!					1	84	172	8.1
#DIV/0!					1	70	207	6.4
#DIV/0!					1	64	240	8
#DIV/0!					1	63	314	6.6
#DIV/0!					1	69	110	7.3
19.4					男性	57	145	14.5
#DIV/0!					1	60	171	9
#DIV/0!					1	81	251	8.2
17.6					1	79	194	6.1

#DIV/0!	1	64	160	7.4
21.5	男性	62		
22.6	1	75	169	7.8
20.7	男性	67	162	8.7
#DIV/0!	1	31	156	8.8
#DIV/0!	1	63	87	7.9
28.1	1	72	208	7.6
24.4	男性	57	******	******
#DIV/0!	1	75	111	6.6
#DIV/0!	1	72	148	8
#DIV/0!	1	82	149	7.6
18.9	男性	66	******	******
20.3	1	77	135	7.7
22.0	1	74	181	7
23.9	男性	68	151	7.2
21.4	男性	71	144	7.2
#DIV/0!	男性	51	283	10.3
#DIV/0!	1	63	152	5.3
#DIV/0!	男性	82	179	7.4
#DIV/0!	1	62	267	9.7
24.5	男性	72	161	7.3
#DIV/0!	男性	74	115	6.9
#DIV/0!	1	55	304	8.8
#DIV/0!	1	56	195	6.8
24.1	1	56	244	9.3
23.9	男性	72	185	7.2
20.3	男性	59	210	9.3
23.6	男性	78	199	7.1
31.2	男性	87	174	8.3
20.6	男性	63	177	7.9
20.7	1	70	303	7.2
#DIV/0!	男性	54	169	8.7
#DIV/0!	男性	63	229	6.3
#DIV/0!	1	57	239	8.7
20.6	男性	53	157	7.4
#DIV/0!	1	61	232	10.2
#DIV/0!	男性	66	******	******
#DIV/0!	男性	56	277	8.3
#DIV/0!	1	66	347	7.7
24.6	男性	80	187	6.9
23.9	男性	50	247	8.5
19.8	1	78	142	7.6

22.7					男性	83	231	7
#DIV/0!					1	58	140	5.6
#DIV/0!					1	74	109	7.7
#DIV/0!					男性	73	277	7.4
#DIV/0!					男性	56	325	10.4
#DIV/0!					1	44	115	5.7
#DIV/0!					男性	77	93	6.7
20.3					男性	85	139	6.2
#DIV/0!					1	60	190	7.7
28.4					1	33	144	7.8
#DIV/0!					男性	64	113	6.2
#DIV/0!					1	62	178	7.4
#DIV/0!					1	53	246	8
23.2					1	59	285	9.8
20.0					1	72	116	6.6
#DIV/0!					1	77	202	7
32.0					男性	66	178	8.7
#DIV/0!					1	46	211	8.6
#DIV/0!					1	61	170	7.7
#DIV/0!					男性	45	166	8.5
#DIV/0!					1	32	216	6.9
#DIV/0!					男性	57	162	7.5
21.9					1	67	127	6.8
#DIV/0!					1	45	148	8
#DIV/0!					1	64	118	6.3
21.9					1	81	104	6.3
#DIV/0!					1	69	192	8.3
29.4	12	0			1	50	120	5.8
#DIV/0!					男性	59	208	9
27.2					男性	60	142	5.4
#DIV/0!					男性	44	149	5.3
#DIV/0!					男性	71	72	6.7
18.6	10		0	0	1	52	148	6.5
#DIV/0!					男性	75	154	6.6
28.3					男性	53	212	8.7
#DIV/0!					1	64	199	7.3
#DIV/0!					1	67	138	6.5
#DIV/0!					1	59	176	8.1
22.2					男性	64	156	6.3
20.6					男性	78	257	6.6
28.1					1	64	144	6.5
23.7					1	67	173	10.6

#DIV/0!				1	84	140	5
22.1				1	87	258	7.1
#DIV/0!				1	56	129	8.1
#DIV/0!				1	49	211	9.1
26.4				男性	77	180	9.8
27.7				男性	74	175	8.2
#DIV/0!				男性	64	169	7.3
23.1				男性	76	210	7.2
18.1				男性	81	******	6.8
#DIV/0!				1	75	284	8.8
#DIV/0!				1	79	148	8.5
19.3				1	74	277	6.8
23.3				男性	59	125	6.7
18.9				男性	80	129	6.3
#DIV/0!				男性	84	158	7.5
#DIV/0!				男性	58	258	11.2
23.0				1	65	108	6.7
35.7				1	46	220	10.5
24.2				1	55	122	6.4
23.5				1	64	159	6
23.9				1	68	76	9.7
18.2				1	74	122	9.4
#DIV/0!				男性	73	145	6.5
22.6				1	68	173	7.3
#DIV/0!				1	77	129	4.8
#DIV/0!				1	87	143	6.7
#DIV/0!				男性	54	261	8.4
25.9				男性	64	127	7
43.1				1	35	184	7.9
22.6				男性	71	165	7
18.6				1	82	193	8.4
#DIV/0!				1	73	157	7.3
28.3				1	47	177	8.2
#DIV/0!				1	59	200	8.5
28.1	4	2	0	1	49	201	6.9
#DIV/0!	0	1	1	1	62	277	******
25.4				男性	63	220	5.8
#DIV/0!	12	2	1	1	59	231	5.8
16.9				男性	79	204	6.9
#DIV/0!				男性	79	106	******
#DIV/0!				1	71	121	7
#DIV/0!				1	68	144	6.8

21.3					1	77	99	6.1
22.8					1	76	231	6.8
21.1					男性	69	253	8.2
19.7					1	79	170	7.4
23.1					男性	86	150	6.9
#DIV/0!					1	69	166	11.1
21.9					1	69	146	5.9
31.1					1	64	141	9.5
25.5					1	63	166	7.1
31.8					1	68	110	7
31.5	31	1			男性	62	148	7.7
23.7	17	1			男性	69	217	7.4
21.3	35	1			男性	69	240	7.9
22.9	12	1	1	1	男性	60	108	6.3
35.0	17	1			男性	61	126	9.8
21.1	37	1	1	0	男性	75	146	6.9
34.9	14	1			男性	53	200	10.7
25.7	14	1			1	50	124	6.8
#DIV/0!					1	65	65	7.7
20.8	22	1			1	71	97	5.6
22.1	16	1	0	1	男性	66	306	8.5
23.1	15	1			1	63	124	6.4
34.3	15	1	0	0	男性	49	132	9.2
21.7	14	1			1	63	153	7.1
25.1	15	0	2		男性	53	172	6.4
31.8	12	1			男性	62	162	7.2
23.5	16	1	2	0	男性	61	258	10.2
25.1	17	1	2	0	男性	51	228	8.3
26.9	12	0	2	1	1	62	137	6.5
24.5	10				男性	63	115	7.1
27.4	7	0			男性	43	111	5.8
25.2	18	1			1	52	133	7.6
28.7	6	1			1	42	236	9.4
#DIV/0!					1	62	122	7.8
23.2	20	1	2	0	1	55	150	6.5
#DIV/0!					1	48	137	5.6
#DIV/0!					男性	63	120	5.9
#DIV/0!					男性	60	307	5.8
27.8					男性	50	118	6.5
#DIV/0!					男性	48	108	7.5
27.1	18	1			1	71	116	7.1

27.8	34		0	0	男性	47	120	9.9
18.2	30	1			1	89	184	8.2
22.6	13	1	1	0	男性	64	64	7.2
#DIV/0!					1	64	190	8
25.6	12	1			1	58	265	9.3
33.6	30	0	0	0	1	47	195	9.6
20.7	20	1	1	1	男性	63	284	8.5
20.2	22		0	0	男性	68	143	6.6
25.2	3	1	2	0	男性	56	131	5.7
26.4	35	1	0	1	1	76	166	7.6
19.6	43	1	0	1	男性	76	59	6.9
20.3	18	1	0	0	男性	69	108	6.5
25.5	17	1			男性	50	267	7.9
27.5	45	1	0	1	男性	71	123	7.6
18.8	17	1	2	0	1	70	138	7.1
35.6	15	0	1	1	男性	60	139	6.4
23.7	23	1			男性	60	170	6.8
35.4	26	1	2	0	男性	60	108	9.2
27.9	36	1			男性	80	129	7.7
31.2	30	1	0	1	男性	81	214	8.8
30.3	14	1	1	0	1	37	163	11.4
27.0	31	1	0	0	1	61	126	7.5
27.8	38	1	1	0	1	71	139	8.2
21.6	20	1			1	63	157	8.9
22.2	17	0			1	67	177	8.1
23.8	3	0	1	0	1	60	199	6.1
18.8					1	64	97	7.2
#DIV/0!					1	41	******	******
#DIV/0!					男性	51		
#DIV/0!					1	60	******	******
#DIV/0!					男性	48		
#DIV/0!					男性	72	175	7
#DIV/0!					男性	38		
#DIV/0!					男性	56		
#DIV/0!					男性	34	******	******
#DIV/0!					1	38	******	******
#DIV/0!					男性	38	******	******
#DIV/0!					男性	24		
#DIV/0!					男性	64		
#DIV/0!					男性	88	127	6.7
#DIV/0!					男性	30		

#DIV/0!			男性	40		
#DIV/0!			男性	32		
#DIV/0!			男性	33		
#DIV/0!			1	86	140	6.7
#DIV/0!			男性	79	125	7
#DIV/0!			男性	77	146	6.2
18.0			男性	82	******	******
#DIV/0!			1	81		******
15.9			1	74	******	******
#DIV/0!			1	70	129	6.3
#DIV/0!			1	70	******	******
24.3			1	87	163	6.4
26.2	0		男性	62	146	6.4
#DIV/0!			1	80	******	******
#DIV/0!			男性	57		
#DIV/0!			男性	86	******	******
#DIV/0!			1	67	******	7.4
#DIV/0!			1	39	******	******
#DIV/0!			1	86	205	5.5
22.8			男性	79	250	6.7
15.2	30	0	男性	86	237	6.9
#DIV/0!			1	77	112	5.8
22.4			1	85	151	6.6
15.5			1	75	151	6.4
#DIV/0!			1	70	135	7.5
#DIV/0!			1	29	******	******
#DIV/0!			1	82	114	6.7
#DIV/0!			男性	55	******	******
#DIV/0!			1	78	98	6.5
#DIV/0!			1	68	201	7.1
#DIV/0!			男性	63	******	******
#DIV/0!			1	81	127	5.9
#DIV/0!			1	68	98	6.6
#DIV/0!			1	60	127	6.2
22.6			1	81	154	8.4
32.8			1	62	202	8
#DIV/0!			1	51	******	******
#DIV/0!			1	64	149	6.5
#DIV/0!			1	60	******	******
#DIV/0!			1	84	******	******
#DIV/0!			1	68	******	******
41.7	1	0	男性	39	164	7

#DIV/0!			1	69	******	******
#DIV/0!			1	80	******	******
#DIV/0!			男性	80	******	******
23.4	6	0	1	77	145	6.5
#DIV/0!			1	49	******	******
#DIV/0!			1	82	128	6.1
18.9			1	76	114	6.2
21.8			1	73	******	******
#DIV/0!			男性	75		
24.0			1	74	111	6.3
#DIV/0!			男性	60	******	******
29.6			1	78	136	7.2
#DIV/0!			1	66	156	6.4
#DIV/0!			男性	90		
28.4			1	76	119	6.8
#DIV/0!			1	73	130	6
#DIV/0!			1	65	149	6.8
#DIV/0!			1	70	******	******
#DIV/0!			男性	25		
#DIV/0!			男性	46	******	******
#DIV/0!			1	68	135	6.3
#DIV/0!			1	78	125	6.4
25.2			1	79	******	******
#DIV/0!			男性	66	141	7
#DIV/0!			1	69	136	6.2
#DIV/0!			1	61	126	6.4
25.7			1	64	162	7.5
24.8			男性	81	116	5.9
#DIV/0!			男性	87	******	******
#DIV/0!			1	82	233	6.8
#DIV/0!			1	78	211	******
#DIV/0!			男性	71	******	******
#DIV/0!			1	65	317	******
#DIV/0!			1	88	******	******
21.5			1	80	108	6.6
#DIV/0!			1	85	******	******
23.8			男性	76	******	******
#DIV/0!			1	78	125	6.6
#DIV/0!			男性	66		
#DIV/0!			男性	70	******	******
24.1			男性	78	234	
#DIV/0!			1	62	******	5.9

#DIV/0!				1	69	124	6.8
#DIV/0!				男性	82		
#DIV/0!				男性	83	145	6.6
24.9				男性	84	123	5.9
#DIV/0!				男性	82		
#DIV/0!				1	64	122	6.6
27.7	2	0		男性	54	145	6.2
#DIV/0!				男性	87	139	7.2
#DIV/0!				男性	80		
#DIV/0!				1	97	******	******
#DIV/0!				男性	76		
#DIV/0!				男性	86		
32.0				1	73	130	5.8
#DIV/0!				1	74	152	7.1
23.1	18	0		1	90	122	5.5
21.0	17	0		男性	83	139	6.2
#DIV/0!				1	23	******	******
#DIV/0!				男性	88		
27.0				男性	74	69	6.5
#DIV/0!				男性	94	******	******
25.3				1	66	181	7.3
22.9				男性	73	******	******
24.9				男性	64	209	7.3
#DIV/0!				1	60	******	******
#DIV/0!				男性	69	******	******
#DIV/0!				男性	55		
#DIV/0!				男性	80	154	6.7
#DIV/0!				1	71	******	******
#DIV/0!				男性	78	146	
#DIV/0!				男性	62		
#DIV/0!				男性	73		
#DIV/0!				男性	60	******	******
#DIV/0!				1	81	139	6.1
23.0	18	0		1	68	101	6
#DIV/0!				男性	70	******	******
#DIV/0!				男性	67		
#DIV/0!				1	77	******	******
#DIV/0!				男性	75		
23.1				男性	84		8.8
#DIV/0!				男性	58		
#DIV/0!				男性	80	169	7.9

#DIV/0!			男性	84		
#DIV/0!			1	69	125	5.9
23.3			男性	77	124	6.4
#DIV/0!			男性	73	125	6.5
#DIV/0!			男性	87	147	5.9
#DIV/0!			男性	86	139	6.6
#DIV/0!			男性	61		
#DIV/0!			1	67	114	6.2
#DIV/0!			1	52	******	******
#DIV/0!			男性	34	******	******
#DIV/0!			1	73	******	******
25.8	6	0	1	63	165	7.1
20.2			1	73	160	7
#DIV/0!			1	62	146	6.2
#DIV/0!			男性	75	******	******
#DIV/0!			男性	59	163	6.8
#DIV/0!			男性	63	157	6.1
#DIV/0!			男性	78	******	******
#DIV/0!			男性	80		
#DIV/0!			1	85	126	9
#DIV/0!			男性	73		
30.9			男性	57	127	7.6
#DIV/0!			男性	67		
#DIV/0!			男性	58	131	6.4
#DIV/0!			1	76	******	******
#DIV/0!			1	61	97	6.4
#DIV/0!			1	77	******	******
32.6	14	1	男性	37	230	7.1
#DIV/0!			男性	84		
#DIV/0!			男性	80		
#DIV/0!			1	84	141	6.4
#DIV/0!			男性	83	119	6.5
#DIV/0!			1	78	138	7
29.4			男性	52	138	
#DIV/0!			男性	59		
#DIV/0!			1	71	106	6.3
#DIV/0!			1	73	******	******
#DIV/0!			男性	61	147	6.8
#DIV/0!			男性	70		
#DIV/0!			1	89	******	******
#DIV/0!			1	71	******	******

18.5	男性	81	230	
36.4	1	69	130	6.2
#DIV/0!	男性	72	161	6.5
#DIV/0!	男性	67	142	6.9
#DIV/0!	男性	67		
#DIV/0!	1	58	143	6
#DIV/0!	1	47	******	******
#DIV/0!	1	53	115	6.4
27.7	1	66	134	6.6
#DIV/0!	男性	74	154	6.8
#DIV/0!	1	69	133	6.3
#DIV/0!	1	75	******	******
#DIV/0!	男性	52	******	******
#DIV/0!	1	61	240	7
18.7	男性	59	191	6.5
#DIV/0!	男性	53	112	8.3
#DIV/0!	男性	62	130	6.4
#DIV/0!	男性	86	188	6.7
30.1	1	56	147	6.7
25.1	1	77	******	******
#DIV/0!	男性	40		
#DIV/0!	男性	48	******	******
#DIV/0!	男性	60	149	7.2
#DIV/0!	男性	40	******	******
#DIV/0!	1	74	123	6.6
#DIV/0!	1	80	148	6.8
21.4	1	60	279	9.4
#DIV/0!	1	80	******	******
#DIV/0!	1	72	133	6.7
22.2	男性	73	126	6.8
#DIV/0!	男性	69		
#DIV/0!	1	70	213	6.2
#DIV/0!	男性	56	118	6
#DIV/0!	男性	63	76	6.6
#DIV/0!	男性	68		
#DIV/0!	1	57	******	******
27.0	男性	53		
#DIV/0!	男性	62	******	******
#DIV/0!	男性	71	******	******
#DIV/0!	1	65	******	******
#DIV/0!	1	76	******	******

#DIV/0!			1	47	******	6.6
23.8			男性	70	142	6.3
#DIV/0!			1	51	164	7.2
#DIV/0!			男性	69	105	6.9
25.4			男性	87	289	7
#DIV/0!			1	72	******	******
#DIV/0!			男性	77	******	******
24.4			1	63	217	6
#DIV/0!			1	42	******	******
#DIV/0!			1	54	******	******
#DIV/0!			男性	64	125	6.7
28.0			男性	73	166	7.1
21.6			男性	78	243	7
#DIV/0!			1	47	******	******
#DIV/0!			男性	76	******	******
#DIV/0!			男性	53	144	7.1
#DIV/0!			1	71	155	6.3
#DIV/0!			1	75	121	6.4
#DIV/0!			1	86	205	5.5
#DIV/0!			1	66	145	6.6
20.8			1	63	148	6.7
#DIV/0!			1	41	153	6.3
#DIV/0!			1	76	210	******
#DIV/0!			男性	78	165	6.7
25.1			1	58	162	6.5
22.5			男性	53	177	7.2
29.1			男性	52	109	6.3
#DIV/0!			男性	65	******	******
#DIV/0!			1	68	108	5.8
21.0	6		男性	70	160	7.1
22.5	9	0	1	75	120	7
#DIV/0!			1	34	171	6.4
#DIV/0!			男性	57	******	******
#DIV/0!			1	74	69	6.3
#DIV/0!			男性	76	214	
#DIV/0!			1	63	157	6.7
#DIV/0!			1	69	121	6.7
#DIV/0!			男性	67	144	7
#DIV/0!			男性	75	******	******
#DIV/0!			1	70	130	6.3
#DIV/0!			男性	70	******	******
#DIV/0!			1	55	119	6.7

#DIV/0!			男性	73	150	6.8
#DIV/0!			男性	61	******	******
23.4			男性	81	152	5.7
#DIV/0!			男性	86	******	******
#DIV/0!			1	64	164	7.2
#DIV/0!			1	61	191	8.2
#DIV/0!			男性	69	121	5.9
27.4			男性	61	99	5.2
#DIV/0!			1	49	157	7.2
#DIV/0!			男性	73	270	******
#DIV/0!			1	45	137	5.7
#DIV/0!			男性	57		
20.1			男性	60	125	6.5
25.4			1	40	121	6.7
#DIV/0!			男性	43	143	6.2
#DIV/0!			男性	52		
22.0			男性	58	124	6.8
#DIV/0!			1	79	112	6.1
#DIV/0!			男性	50	******	******
#DIV/0!			男性	70	191	6.8
#DIV/0!			1	82	******	******
26.1			男性	77	208	
#DIV/0!			男性	43	201	6
22.2			男性	75	125	6.7
32.8			男性	63	126	6.8
#DIV/0!			男性	30		
#DIV/0!			1	65	142	7.5
25.3	10	0	1	64	71	6.5
#DIV/0!			男性	67	114	6.5
26.6			男性	41	195	7.4
#DIV/0!			男性	62	136	6.1
#DIV/0!			男性	66	139	6.5
25.3			1	68	******	******
#DIV/0!			1	62	******	******
#DIV/0!			1	36	135	6.8
#DIV/0!			男性	39	******	******
#DIV/0!			男性	42	******	******
#DIV/0!			男性	23	******	******
#DIV/0!			男性	26	******	******
#DIV/0!			男性	37	171	9
#DIV/0!			男性	51	******	******

#DIV/0!	男性	64	******	******
#DIV/0!	男性	31	******	******
#DIV/0!	男性	74		6.3
#DIV/0!	男性	56	******	******
#DIV/0!	男性	66	181	5.1
#DIV/0!	男性	37	******	******
#DIV/0!	男性	76		
#DIV/0!	男性	56		
#DIV/0!	男性	67	218	7.8
#DIV/0!	男性	47		
#DIV/0!	男性	60	194	7.1
#DIV/0!	男性	54		
#DIV/0!	男性	25		
21.3	男性	54	145	8.3
#DIV/0!	男性	31		
#DIV/0!	男性	22		
#DIV/0!	男性	59	206	
#DIV/0!	男性	61		
19.2	男性	75		6.4
21.8	男性	81	115	7.2
22.0	男性	62		6.6
#DIV/0!	男性	41		
#DIV/0!	男性	27		
#DIV/0!	男性	31		
#DIV/0!	男性	25		
#DIV/0!	男性	29		
#DIV/0!	男性	56		
#DIV/0!	男性	75		
#DIV/0!	男性	56		
#DIV/0!	男性	50		
#DIV/0!	男性	57		
#DIV/0!	男性	50		
#DIV/0!	男性	46		
#DIV/0!	男性	27		
#DIV/0!	男性	45		
#DIV/0!	男性	32		
#DIV/0!	男性	39		
#DIV/0!	男性	51	******	******
23.5	1	76	114	7.6

#DIV/0!					1	35	126	6.8
#DIV/0!					男性	56	169	6.7
#DIV/0!					男性	61	147	6.8
#DIV/0!					1	67	******	******
#DIV/0!					1	51	135	6.5
32.4					1	66	103	
#DIV/0!					1	64	133	7.1
#DIV/0!	1				1	56	******	7
#DIV/0!					1	82	185	6.3
#DIV/0!					1	62	******	******
#DIV/0!					男性	37		
#DIV/0!					男性	69		
#DIV/0!					男性	56		
#DIV/0!					男性	76		
13.2					男性	66	145	6.8
25.8					男性	63	141	6.7
25.3	12	1			1	75	155	6.2
#DIV/0!					男性	65	171	
19.3	12	0			1	76	143	6.5
24.4					1	70	******	******
22.5	6	0	1	0	1	64	******	******
25.8					男性	77	125	6.8
19.5					1	69	333	8.1
#DIV/0!					男性	66	******	******
21.4					男性	69	191	
24.9					1	86	138	6
#DIV/0!					1	94	111	6.5
30.1					1	79	125	6.6
26.1					1	74	109	6.5
#DIV/0!					男性	75	117	5.9
30.7					男性	59	251	8.5
#DIV/0!					1	59	130	6.4
25.3					男性	86	142	7.3
#DIV/0!					1	75	130	7.3
26.8					1	62	146	6.5
25.4					1	71	138	6.7
25.2					男性	74	139	6.5
#DIV/0!					男性	88	140	6.4
#DIV/0!					1	63	125	5.9
18.7	1	0			1	70	158	6.5
18.1					1	76	93	5.8
#DIV/0!					1	57	209	5.9

29.1					男性	74	141	7.3
23.5					男性	63	88	6.5
26.2					1	74	******	9.3
20.8					男性	51	97	4.8
21.9					1	80	231	8.4
17.5	14	1	0	0	1	75	236	8.4
24.2					1	71	******	******
21.1					男性	74	148	6.8
22.1	6	0			男性	63	113	6
32.2	15	1	0	0	1	55	125	8
22.1					1	54	128	6.5
26.2					男性	68	210	
21.8					男性	76	198	6.7
#DIV/0!					男性	70	124	7
#DIV/0!					男性	31		
#DIV/0!					男性	60		
25.4					1	76	123	6.6
25.9					1	64	125	6.2
#DIV/0!					男性	34	******	******
#DIV/0!					1	70	142	6.6
#DIV/0!					1	76	175	6.5
19.1	29	0			男性	85	153	7.5
46.4					男性	50	291	10.5
39.4					1	75	142	6.6
#DIV/0!					男性	84	88	5.5
19.9	12	0			1	64	******	******
22.4					男性	86	128	7
#DIV/0!					1	64	******	******
16.7					1	60	263	******
#DIV/0!					1	81	138	6.4
27.3					1	80	152	6.5
#DIV/0!					男性	74		
#DIV/0!					1	82	139	7.3
23.6					1	78	210	6.2
#DIV/0!					男性	74		7.3
#DIV/0!					男性	75	151	7.1
#DIV/0!					男性	84		
23.2					1	67	145	7.2
#DIV/0!					男性	84	******	******
#DIV/0!					男性	71	******	******
#DIV/0!					1	43	******	******

22.7					1	71	127	6.4
32.8					1	60	545	10.5
#DIV/0!					1	72	146	6.8
25.1					男性	77	117	5.5
35.1					1	62	169	7.6
18.4					男性	75	256	8.2
#DIV/0!					1	43	141	7
22.0	7	0			1	62	150	6.5
18.3					1	47	99	5
#DIV/0!					男性	63	141	6.6
13.8					1	76	158	7
15.8					男性	76	229	
23.6	20		2	1	1	58	78	5.2
24.5					男性	66	165	7.7
#DIV/0!	0		2	0	1	63	132	7.6
#DIV/0!					男性	56	207	5.1
19.1					男性	78	486	14.2
20.8	6		1	0	1	67	100	6.6
#DIV/0!	0		1	1	1	73	198	10.7
#DIV/0!					男性	81	130	6.5
26.7					1	68	140	6.9
16.9					男性	80	******	******
#DIV/0!					1	76	******	******
22.6	18	0			1	91	108	6.4
21.1					1	73	126	7.4
24.7	14	1			男性	72	118	6.5
21.2					男性	66	134	6.5
17.3					1	82	206	5.9
27.0					男性	57	144	6.7
15.6					1	80	241	6.4
18.7					1	79	183	6.6
27.4	9	0			1	83	159	7.4
#DIV/0!					男性	61	118	5.7
#DIV/0!					1	52	128	6.8
#DIV/0!					1	63	276	7.5
24.0					男性	67	148	6.3
31.2					男性	59	84	6.8
26.8					男性	57	82	4.8
17.6					1	80	147	4.9
24.0					男性	76	169	6.6
17.6	1		0	0	1	56	137	7
18.8					男性	73	247	7.3

#DIV/0!			男性	63	139	6.4
20.8	26	0	1	73	129	5.8
#DIV/0!			男性	75		
#DIV/0!			1	84	******	******
20.3			男性	79		
#DIV/0!			1	84	100	5.2
#DIV/0!			男性	68		
#DIV/0!			1	86	81	******
#DIV/0!			男性	51		
#DIV/0!			1	23	******	******
26.1			1	61	******	******
#DIV/0!			1	55	108	5.2
#DIV/0!			1	64	103	******
#DIV/0!			1	41	100	******
#DIV/0!			男性	70	107	
#DIV/0!			男性	77	119	
#DIV/0!			1	69	******	5.4
#DIV/0!			1	26	******	******
#DIV/0!			男性	57	108	
#DIV/0!			男性	41		
22.1			1	83	97	5.4
22.6			男性	45	105	5
#DIV/0!			1	48	******	******
15.6			1	88	******	******
#DIV/0!			1	75	******	******
#DIV/0!			1	53	107	5.1
#DIV/0!			男性	48	115	******
22.8			男性	49	119	5
#DIV/0!			1	61	******	******
#DIV/0!			1	64	******	******
#DIV/0!			男性	60		
#DIV/0!			男性	63	117	5.8
#DIV/0!			1	36	******	******
#DIV/0!			男性	56		
#DIV/0!			1	63	******	******
#DIV/0!			1	67	******	******
#DIV/0!			1	66	129	6
26.7			男性	75	97	4.7
#DIV/0!			男性	41	105	******
#DIV/0!			男性	45	******	******
#DIV/0!			男性	54		5.4
#DIV/0!			男性	83	149	******

19.9	男性	69	144	6
#DIV/0!	男性	71	118	
22.2	男性	76		5.4
#DIV/0!	男性	41	96	
#DIV/0!	男性	37		
#DIV/0!	男性	40		
#DIV/0!	男性	55	126	
#DIV/0!	男性	80	142	5.1
26.2	男性	71	100	
#DIV/0!	男性	26		
#DIV/0!	男性	60	77	******
#DIV/0!	男性	51	106	
22.8	1	76	******	******
#DIV/0!	1	75	******	******
#DIV/0!	1	62	97	5.5
#DIV/0!	男性	83	122	
#DIV/0!	1	81	106	6
#DIV/0!	1	68	******	******
22.7	男性	74		
#DIV/0!	男性	65	112	
#DIV/0!	男性	56	******	5.9
#DIV/0!	男性	71		
#DIV/0!	男性	72	141	6.1
#DIV/0!	男性	62		
#DIV/0!	男性	63	102	
#DIV/0!	1	72	******	******
#DIV/0!	1	78	118	5.8
#DIV/0!	1	48	******	******
#DIV/0!	男性	77		
#DIV/0!	男性	66		
#DIV/0!	男性	82		
15.0	男性	41		
21.9	男性	73	100	
21.8	男性	84	109	5.7
#DIV/0!	1	82	103	5.3
#DIV/0!	1	60	******	******
#DIV/0!	1	97	******	******
#DIV/0!	1	64	******	******
#DIV/0!	1	66	103	5.7
#DIV/0!	1	82	******	******

#DIV/0!	1	82	138	5.5
#DIV/0!	1	79	101	5.1
#DIV/0!	男性	70	118	5.6
22.5	1	65	******	******
20.4	1	80	******	******
#DIV/0!	1	83	138	******
#DIV/0!	1	70	******	******
#DIV/0!	1	77	******	******
#DIV/0!	1	36	******	******
#DIV/0!	男性	61	******	******
#DIV/0!	1	41	103	5
23.3	1	39	******	******
24.7	1	94	112	******
24.2	1	66	90	4.9
16.5	1	81	103	4.7
#DIV/0!	1	80	******	******
20.7	男性	75	******	5.4
#DIV/0!	男性	78	124	5.3
#DIV/0!	男性	68	******	******
#DIV/0!	1	60	106	5.8
#DIV/0!	1	60	112	5.2
#DIV/0!	男性	64	******	******
#DIV/0!	1	62	******	******
18.3	1	70	******	******
20.5	男性	58	113	
#DIV/0!	1	81	109	5.8
#DIV/0!	1	63	******	******
25.7	1	75	******	******
23.4	1	84	118	5.1
#DIV/0!	1	75	******	******
24.4	男性	81	******	******
#DIV/0!	1	69	******	******
#DIV/0!	1	52	97	5.3
#DIV/0!	1	55	138	4.6
#DIV/0!	男性	73	119	5.7
#DIV/0!	1	74	94	4.7
#DIV/0!	1	65	137	5
24.4	1	73	99	******
#DIV/0!	1	68	98	******
#DIV/0!	1	77	98	******
16.2	1	74	92	******
#DIV/0!	男性	62	99	5.4
#DIV/0!	男性	80	******	4

#DIV/0!	1	87	92	5.3
22.2	1	69	106	******
29.0	1	70	******	5.1
#DIV/0!	1	75	******	******
#DIV/0!	男性	77	154	5.5
24.2	1	81	******	5.6
#DIV/0!	1	63	92	5
#DIV/0!	1	81	151	5.3
15.2	1	77	124	5.8
#DIV/0!	男性	86	140	5.4
#DIV/0!	1	67	******	******
22.5	1	76	97	******
23.0	1	91	111	5
19.8	1	83	******	******
#DIV/0!	1	61	109	5.5
21.4	男性	60	105	5.2
22.6	男性	65	101	5.2
26.1	1	59	104	5.4
#DIV/0!	1	70	127	******
#DIV/0!	1	84	******	******
#DIV/0!	1	90	109	5.3
18.3	1	69	******	******
#DIV/0!	1	64	******	******
#DIV/0!	1	74	******	5.1
#DIV/0!	1	83	106	5.2
#DIV/0!	男性	76	94	******
22.2	1	71	96	5.3
#DIV/0!	男性	81	******	******
#DIV/0!	1	78	105	5.3
24.9	1	74	138	5.8
#DIV/0!	男性	73	108	4.8
#DIV/0!	1	72	******	******
25.3	1	82	128	8.1
#DIV/0!	1	62	102	5.7
#DIV/0!	1	73	******	******
21.7	1	77	******	******
#DIV/0!	1	80	******	******
17.6	1	85	130	******
#DIV/0!	1	69	105	5.5
20.8	1	71	******	******
#DIV/0!	1	52	98	5.5
22.5	1	75	******	5.3
19.4	1	90	******	******

#DIV/0!	男性	77			
#DIV/0!	1	63	******	******
#DIV/0!	男性	54	******	******
#DIV/0!	1	79	******	******
23.7	1	67	115	5.6
#DIV/0!	1	89	******	******
#DIV/0!	1	67	******	******
#DIV/0!	1	77	******	******
#DIV/0!	男性	40	100	5.1
#DIV/0!	男性	71		
#DIV/0!	男性	64	121	5.7
#DIV/0!	男性	73		
#DIV/0!	1	71	******	******
#DIV/0!	1	72	******	******
23.9	男性	66		
#DIV/0!	男性	61		
23.5	1	78	******	******
#DIV/0!	1	62	127	5.6
#DIV/0!	1	78	107	5.2
#DIV/0!	男性	58		
#DIV/0!	1	66	******	******
#DIV/0!	男性	56	106	******
#DIV/0!	1	76	******	******
#DIV/0!	1	76	******	******
#DIV/0!	1	89	110	4.9
#DIV/0!	1	84	******	******
#DIV/0!	1	73	96	******
#DIV/0!	1	63	96	4.8
#DIV/0!	1	71	107	5.3
#DIV/0!	1	83	******	******
18.4	男性	89		
#DIV/0!	男性	60	******	******
#DIV/0!	1	73	109	5.8
#DIV/0!	1	67	106	5.1
20.3	1	81	147	6
#DIV/0!	1	65	100	5.5
#DIV/0!	1	63	109	******
24.0	1	84	76	5.7
#DIV/0!	男性	67	114	5.6
22.5	1	78	104	5.3
#DIV/0!	1	63	167	6.1
23.2	1	75	113	5.9

24.4	1	62	94	******
20.7	1	80	97	******
#DIV/0!	男性	64	74	
#DIV/0!	男性	72	******	******
#DIV/0!	1	70	******	******
#DIV/0!	男性	64	103	5.2
#DIV/0!	1	57	105	4.9
17.8	男性	85	140	5.5
18.5	1	35	101	******
#DIV/0!	1	76	106	5.3
#DIV/0!	1	72	110	5.2
#DIV/0!	1	81	******	******
#DIV/0!	1	83	******	******
23.9	男性	81	90	5.2
#DIV/0!	男性	33		
#DIV/0!	1	74	******	5.3
#DIV/0!	1	67	******	******
22.0	1	78	111	5.1
21.9	1	77	******	******
#DIV/0!	1	80	104	5.1
#DIV/0!	男性	79	93	******
#DIV/0!	1	61	******	******
#DIV/0!	1	62	94	******
#DIV/0!	1	67	102	******
#DIV/0!	男性	54	115	5.8
#DIV/0!	男性	58		
#DIV/0!	1	79	101	6.1
16.0	男性	80	99	4.9
#DIV/0!	1	74	123	5.9
#DIV/0!	男性	61	123	5.6
28.1	1	70	105	******
#DIV/0!	1	78	102	5.6
#DIV/0!	1	71	97	******
#DIV/0!	1	63	118	5
20.9	1	62	75	5.4
#DIV/0!	1	75	168	5.8
22.0	男性	67	105	5.6
23.9	男性	70	146	
#DIV/0!	1	66	97	4.5
#DIV/0!	1	51	116	5.6
26.9	1	68	95	******
#DIV/0!	1	58	******	******

#DIV/0!	1	64	******	5.2
#DIV/0!	1	72	126	5.7
19.7	1	75	103	******
#DIV/0!	男性	51		
#DIV/0!	1	82	100	5.1
#DIV/0!	男性	80	******	******
#DIV/0!	1	69	******	******
#DIV/0!	1	50	107	******
#DIV/0!	1	87	109	5.7
#DIV/0!	男性	58	******	******
#DIV/0!	男性	99	108	4.9
#DIV/0!	1	76	123	5.9
15.4	1	90	******	******
#DIV/0!	1	77	******	******
19.7	1	74	******	******
24.7	1	65	182	5
#DIV/0!	1	74	125	5.9
#DIV/0!	男性	83	109	5.9
#DIV/0!	1	79	100	5.4
18.9	1	84	91	5
25.0	1	77	******	******
26.2	1	77	112	5.9
#DIV/0!	1	89	108	5.2
20.5	1	80	107	******
#DIV/0!	1	73	108	******
25.1	1	75	******	******
#DIV/0!	1	86	******	******
#DIV/0!	男性	78	100	5.6
#DIV/0!	1	55	111	******
#DIV/0!	1	69	112	4.7
19.8	男性	68	103	
#DIV/0!	男性	68	******	******
#DIV/0!	1	82	******	******
#DIV/0!	1	67	******	5.3
#DIV/0!	男性	64	108	4.9
#DIV/0!	1	75	117	5.6
22.3	1	64	111	5.4
21.8	男性	82	******	******
21.5	1	86	86	******
#DIV/0!	男性	63		
#DIV/0!	1	81	******	******
#DIV/0!	1	62	129	6

#DIV/0!	男性		60	111	5.9
#DIV/0!		1	95	******	******
19.7	男性		81	112	5.4
#DIV/0!		1	101	******	******
#DIV/0!		1	81	******	******
24.2	男性		80	102	
#DIV/0!		1	50	104	4.6
25.3		1	76	******	******
#DIV/0!	男性		76	105	******
#DIV/0!		1	50	116	6
#DIV/0!		1	80	108	******
18.8	男性		76	109	5.3
#DIV/0!		1	70	127	5.8
25.0		1	55	******	5.7
#DIV/0!	男性		68	******	******
#DIV/0!		1	76	109	5.7
23.9		1	78	104	5.5
#DIV/0!	男性		73		
#DIV/0!	男性		75	******	******
#DIV/0!	男性		55	93	5
#DIV/0!		1	69	101	******
#DIV/0!		1	70	94	4.9
#DIV/0!		1	60	******	******
#DIV/0!		1	41	******	******
#DIV/0!		1	80	******	******
#DIV/0!		1	81	98	******
22.3		1	81	******	******
#DIV/0!		1	80	102	5.3
#DIV/0!		1	71	108	5.2
#DIV/0!	男性		66	******	******
#DIV/0!		1	62	******	******
24.1	男性		73	92	5.1
#DIV/0!		1	77	95	5.4
#DIV/0!	男性		80	******	******
#DIV/0!		1	72	125	5.5
#DIV/0!		1	73	115	5.2
24.7		1	78	******	******
#DIV/0!		1	86	114	5.4
#DIV/0!		1	70	******	******
26.3		1	68	******	******
20.2		1	69	84	5.5
19.8		1	71	98	5.3

#DIV/0!	1	66	******	******
#DIV/0!	1	59	151	5.6
#DIV/0!	1	79	124	5.7
#DIV/0!	1	87	******	******
#DIV/0!	1	74	103	4.7
#DIV/0!	1	52	135	5.3
21.2	1	76	******	******
22.6	男性	64	102	
#DIV/0!	男性	67	******	******
#DIV/0!	1	82	105	5.4
#DIV/0!	1	68	124	6.1
20.8	1	69	114	4.9
19.1	1	67	120	5.8
#DIV/0!	1	62	******	******
#DIV/0!	男性	78	134	5.7
19.7	1	88	******	******
#DIV/0!	1	47	102	4.9
#DIV/0!	1	73	******	******
#DIV/0!	1	63	102	5.1
28.7	1	70	116	5.7
#DIV/0!	男性	79	108	5.4
#DIV/0!	男性	45	99	
#DIV/0!	男性	52	95	4.9
#DIV/0!	1	69	******	******
22.2	1	83	157	5.4
20.8	1	89	102	5.6
#DIV/0!	1	69	93	******
23.0	1	86	131	5.5
#DIV/0!	1	88	100	******
#DIV/0!	1	86	******	******
#DIV/0!	男性	76		5.5
#DIV/0!	1	83	******	******
#DIV/0!	1	74	******	******
18.6	男性	78		
#DIV/0!	1	60	166	6
21.5	男性	53	111	5.3
#DIV/0!	1	61	115	5.3
#DIV/0!	1	78	******	6
24.0	1	76	93	5.6
#DIV/0!	1	80	******	******
31.3	1	79	88	5
#DIV/0!	男性	57	******	******

#DIV/0!	1	59	******	******
#DIV/0!	男性	55	99	5.2
#DIV/0!	1	59	156	5.1
#DIV/0!	1	71	103	******
#DIV/0!	1	82	******	******
#DIV/0!	男性	78	******	******
#DIV/0!	男性	62		
#DIV/0!	男性	51	120	******
#DIV/0!	1	88	******	******
#DIV/0!	男性	43	126	5.7
#DIV/0!	1	70	107	6.1
#DIV/0!	1	70	******	******
#DIV/0!	男性	78	122	5.3
#DIV/0!	男性	57	******	******
#DIV/0!	1	72	82	5.6
22.8	1	72	******	******
#DIV/0!	男性	86		
#DIV/0!	1	25	******	******
#DIV/0!	男性	58	96	5.4
27.0	男性	76	149	5.1
19.0	1	78	92	5.2
#DIV/0!	1	57	109	5
#DIV/0!	1	60	95	5.4
19.8	男性	57	127	5.5
#DIV/0!	1	80	110	******
#DIV/0!	1	63	93	4.6
#DIV/0!	男性	63		
19.4	男性	23	94	
#DIV/0!	1	72	******	******
#DIV/0!	1	57	109	5.5
#DIV/0!	男性	55	95	4.9
#DIV/0!	1	56	99	5.7
24.1	男性	68		
#DIV/0!	1	67	97	5.2
#DIV/0!	1	74	120	5.2
#DIV/0!	男性	74		
#DIV/0!	男性	89		
#DIV/0!	男性	45	117	5.5
#DIV/0!	男性	76	99	
22.7	1	60	117	4.8
#DIV/0!	男性	75	151	

22.5	男性	50	99	
#DIV/0!	1	60	94	4.9
#DIV/0!	男性	49	94	5.5
22.7	1	61	******	******
#DIV/0!	1	77	117	5.6
20.6	男性	66	91	******
#DIV/0!	1	89	******	******
#DIV/0!	1	84	******	******
23.7	男性	81	97	
18.4	男性	86		
#DIV/0!	男性	76	113	
25.8	1	76	162	5.6
21.5	1	87	******	******
#DIV/0!	男性	74	103	
#DIV/0!	1	84	66	4.5
#DIV/0!	男性	53		
#DIV/0!	1	61	116	5.2
#DIV/0!	男性	69	129	5.6
22.9	男性	69	110	5.6
#DIV/0!	1	61	******	5.3
#DIV/0!	男性	83	132	5.5
#DIV/0!	1	71	******	******
#DIV/0!	男性	67	111	5.6
#DIV/0!	男性	83	******	******
22.2	男性	63	91	4.9
23.0	男性	72	113	5.2
#DIV/0!	男性	60		5.7
#DIV/0!	男性	49	96	******
#DIV/0!	男性	80		
#DIV/0!	男性	71	******	******
#DIV/0!	男性	76		
#DIV/0!	1	75	******	******
#DIV/0!	1	78	******	******
25.4	1	62	118	5.3
25.2	1	74	******	******
#DIV/0!	1	79	94	******
#DIV/0!	1	81	******	******
#DIV/0!	男性	60		
#DIV/0!	男性	75	104	
#DIV/0!	1	54	99	******
39.3	男性	86		

22.2	男性	64	95	4.9
#DIV/0!	男性	66	98	5.5
30.0	1	57	101	5
30.1	1	67	******	******
#DIV/0!	男性	79		
19.5	1	77	124	5.3
#DIV/0!	1	80	******	******
27.8	男性	71	102	5.6
#DIV/0!	男性	72		
#DIV/0!	1	61	******	******
28.9	1	63	******	5
#DIV/0!	男性	83	102	4.8
#DIV/0!	1	54	******	******
#DIV/0!	1	70	120	5.8
#DIV/0!	1	62	117	******
#DIV/0!	1	64	******	******
19.8	1	70	108	5.1
#DIV/0!	1	86	93	******
#DIV/0!	男性	78	104	5.5
26.0	男性	80	110	******
#DIV/0!	男性	64		
#DIV/0!	1	52	******	******
27.4	男性	57	118	5.5
#DIV/0!	1	70	102	5
#DIV/0!	男性	89	******	5.2
19.7	1	59	98	5.8
22.2	1	58	129	5
#DIV/0!	1	67	90	******
23.2	1	85	133	5.6
#DIV/0!	男性	82		
#DIV/0!	1	49	******	******
22.1	男性	52	91	
22.4	1	84	126	4.1
26.4	1	76	104	******
#DIV/0!	男性	74		
25.5	1	60	******	******
#DIV/0!	1	84	89	5.7
24.8	1	67	******	******
#DIV/0!	男性	71		
#DIV/0!	1	70	105	4.8
#DIV/0!	1	80	88	5

27.8	1	68	107	******
#DIV/0!	1	56	******	******
16.5	1	74	******	******
#DIV/0!	1	77	******	******
#DIV/0!	1	57	102	5.4
#DIV/0!	1	78	******	******
#DIV/0!	1	63	******	******
#DIV/0!	男性	75		
#DIV/0!	1	68	101	5.9
#DIV/0!	1	61	105	5.1
#DIV/0!	男性	57	108	
#DIV/0!	男性	51	92	4.5
#DIV/0!	1	75	108	5.5
#DIV/0!	男性	85	100	5.3
#DIV/0!	男性	76	******	******
#DIV/0!	1	79	******	******
#DIV/0!	1	79	93	4.7
26.2	1	66	98	5.6
#DIV/0!	1	69	******	******
#DIV/0!	1	54	******	******
24.0	男性	85	104	5.4
#DIV/0!	男性	64		
14.5	1	69	96	5.1
#DIV/0!	男性	45	93	4.9
19.4	男性	80		
21.4	1	54	104	5.1
#DIV/0!	男性	85	171	5.6
#DIV/0!	男性	65		5.2
23.0	1	92	95	******
25.1	1	71	107	5.1
#DIV/0!	男性	58		
#DIV/0!	1	51	122	5.9
#DIV/0!	男性	59	99	******
17.7	男性	90	80	******
#DIV/0!	1	45	******	5.7
#DIV/0!	男性	60	108	6
#DIV/0!	男性	76		
18.7	男性	74		
22.9	男性	78	92	
19.9	男性	69	92	4.9
#DIV/0!	男性	70		

#DIV/0!	1	75	******	5.7
#DIV/0!	男性	76	92	5.1
#DIV/0!	1	41	97	******
22.0	1	73	113	4.2
#DIV/0!	男性	68	97	
#DIV/0!	1	75	******	******
#DIV/0!	男性	71		5
#DIV/0!	1	73	98	5.4
#DIV/0!	1	77	******	******
#DIV/0!	1	90	******	******
#DIV/0!	男性	59	135	5.9
#DIV/0!	男性	69		5.3
26.4	男性	71	104	5.5
18.7	男性	62	96	
#DIV/0!	男性	75	114	5.4
#DIV/0!	男性	66	93	6
#DIV/0!	1	71	88	******
#DIV/0!	1	73	120	5.8
#DIV/0!	男性	68	100	
#DIV/0!	1	80	******	******
#DIV/0!	男性	61	110	5.8
23.2	男性	43	94	4.9
#DIV/0!	1	69	******	5.3
#DIV/0!	男性	79		
#DIV/0!	男性	58		
#DIV/0!	男性	79	105	5.6
#DIV/0!	男性	66	******	******
#DIV/0!	1	72	******	******
#DIV/0!	男性	65	******	******
#DIV/0!	男性	64	95	5.4
#DIV/0!	1	68	******	******
#DIV/0!	1	78	******	******
#DIV/0!	1	71	******	******
#DIV/0!	男性	67	******	******
#DIV/0!	1	76	******	******
#DIV/0!	1	73	98	******
23.4	1	44	124	5
#DIV/0!	1	77	******	******
26.2	1	42	******	5.8
#DIV/0!	1	80	116	5.7
#DIV/0!	男性	47		

#DIV/0!	男性	68	113		
#DIV/0!	男性	75	97		
#DIV/0!	1	73	99	5.2	
#DIV/0!	1	84	******	******	
#DIV/0!	男性	71	******	******	
#DIV/0!	1	77	******	******	
22.8	1	85	******	******	
21.1	男性	76		5.1	
#DIV/0!	男性	64		5.9	
#DIV/0!	1	62	93	5.2	
#DIV/0!	男性	57	133	5.7	
#DIV/0!	1	61	******	******	
#DIV/0!	男性	78	98	******	
#DIV/0!	1	51	97	5.2	
25.1	男性	75		5.5	
18.1	男性	79	******	5.3	
23.4	男性	76	111		
27.6	男性	62	110		
#DIV/0!	男性	64	108	5.9	
19.8	1	73	97	5	
#DIV/0!	1	75	99	******	
#DIV/0!	1	60	107	5.7	
#DIV/0!	男性	50	89	******	
#DIV/0!	1	82	116	4.6	
#DIV/0!	男性	62	93	******	
23.3	1	85	103	******	
#DIV/0!	1	77	102	5.4	
#DIV/0!	男性	72	94	5.4	
25.2	男性	67	106		
#DIV/0!	男性	78	******	5.9	
20.2	男性	63	150	4.8	
20.4	1	67	******	5.1	
#DIV/0!	男性	81	91		
#DIV/0!	1	87	94	5.4	
#DIV/0!	1	62	******	******	
26.6	男性	89	111		
20.1	1	54	99	4.8	
#DIV/0!	男性	63	98	5.2	
#DIV/0!	1	68	151	******	
#DIV/0!	男性	74	115	6.1	
#DIV/0!	男性	75	97	5.4	

#DIV/0!	1	61	110	******
#DIV/0!	男性	64	******	******
#DIV/0!	男性	47		
#DIV/0!	1	70	******	******
#DIV/0!	1	79	109	5.9
#DIV/0!	男性	51	******	******
24.3	男性	67	******	******
19.8	1	71	******	******
#DIV/0!	1	75	118	6.2
22.8	1	63	******	******
#DIV/0!	1	85	******	******
#DIV/0!	男性	84	******	******
#DIV/0!	1	81	******	******
#DIV/0!	1	68	******	******
#DIV/0!	1	85	******	******
#DIV/0!	1	86	******	******
#DIV/0!	1	54	119	5.7
22.7	男性	63	112	
#DIV/0!	男性	66		
#DIV/0!	1	61	******	******
#DIV/0!	男性	39	103	5.1
#DIV/0!	1	76	87	5.5
#DIV/0!	1	60	******	******
#DIV/0!	1	73	103	5.3
26.7	男性	81	113	
#DIV/0!	男性	51	101	
21.2	男性	55	95	4.8
#DIV/0!	男性	49	118	
23.4	男性	77	103	5.7
25.1	男性	75	111	4.9
#DIV/0!	1	71	100	5.1
#DIV/0!	1	68	116	******
#DIV/0!	男性	74		
#DIV/0!	男性	67	101	5.4
#DIV/0!	1	81	167	******
21.4	男性	83	101	5.5
18.3	1	75	******	******
27.9	1	71	104	5.5
#DIV/0!	男性	78	104	5.3
#DIV/0!	男性	48	99	
#DIV/0!	男性	59	******	******

#DIV/0!	男性	61		
#DIV/0!	男性	71		5.6
#DIV/0!	男性	81		
19.7	男性	75		
#DIV/0!	1	62	139	5.6
#DIV/0!	男性	73	96	
21.2	1	89	******	******
#DIV/0!	男性	74	104	5.3
#DIV/0!	男性	66	132	5.8
19.4	1	74	130	5.3
#DIV/0!	1	72	94	5.3
#DIV/0!	1	62	******	******
#DIV/0!	1	75	123	******
23.2	1	57	101	5.2
#DIV/0!	1	67	128	6.1
#DIV/0!	1	63	97	5.5
#DIV/0!	男性	64	104	******
#DIV/0!	男性	47	98	5.2
#DIV/0!	男性	69		
#DIV/0!	1	62	******	5.9
#DIV/0!	1	59	122	******
26.3	1	79	153	5
24.6	1	70	103	5.2
24.0	1	72	93	5.1
#DIV/0!	男性	74	102	
#DIV/0!	男性	64	64	6.9
#DIV/0!	1	72	98	5.3
25.5	男性	52	94	5.3
#DIV/0!	1	79	116	6
#DIV/0!	男性	61	131	4.4
#DIV/0!	男性	76	92	******
20.6	男性	88	114	5.3
#DIV/0!	1	62	98	******
#DIV/0!	1	69	******	******
26.8	1	77	127	5.2
#DIV/0!	1	65	132	******
22.4	男性	60	104	5.8
20.8	男性	66	92	5.4
22.2	男性	65	******	5.6
#DIV/0!	1	69	104	5
#DIV/0!	男性	81	103	5.7

#DIV/0!	1	58	104	******
#DIV/0!	1	60	104	4.9
#DIV/0!	男性	76		
#DIV/0!	男性	55	135	6
#DIV/0!	男性	71	93	6.3
#DIV/0!	男性	68	******	******
#DIV/0!	1	54	93	5.3
#DIV/0!	1	68	103	5.4
#DIV/0!	男性	49	109	4.9
#DIV/0!	1	68	109	******
#DIV/0!	男性	27	93	4.8
29.3	1	65	108	5
#DIV/0!	1	37	105	4.9
#DIV/0!	男性	77	101	4.6
24.8	1	55	115	5.3
36.4	1	63	94	******
#DIV/0!	1	60	******	5.4
25.6	1	63	106	5.7
24.8	1	71	108	******
#DIV/0!	1	60	101	******
#DIV/0!	1	89	98	5.3
22.4	男性	68	91	5.3
#DIV/0!	1	67	120	5.3
24.4	男性	82	95	4.7
#DIV/0!	男性	62	146	5.9
#DIV/0!	1	36	103	******
30.9	1	73	121	5.6
#DIV/0!	1	63	141	5.2
#DIV/0!	男性	52	109	
#DIV/0!	男性	85		
26.2	1	79	138	******
#DIV/0!	1	61	101	******
#DIV/0!	男性	85	139	4.9
20.6	男性	72	104	
#DIV/0!	男性	72	105	4.8
#DIV/0!	1	65	105	5.1
23.3	男性	52	******	5.5
#DIV/0!	1	92	******	5.6
17.8	1	69	122	******
23.3	男性	63	113	******
#DIV/0!	男性	69	110	5
22.3	男性	78	116	******

18.8	男性	83	130	
#DIV/0!	1	55	89	5.4
#DIV/0!	男性	85	111	
#DIV/0!	1	73	97	******
#DIV/0!	男性	55	150	******
#DIV/0!	1	59	150	******
23.7	男性	62	123	5.9
#DIV/0!	1	68	138	6.1
#DIV/0!	男性	78	110	******
#DIV/0!	1	77	101	******
#DIV/0!	1	59	113	5.5
#DIV/0!	男性	64	109	
#DIV/0!	男性	53	92	******
#DIV/0!	男性	63	91	5
#DIV/0!	1	65	80	5.3
16.5	1	81	122	******
33.2	男性	51	******	5.4
#DIV/0!	男性	60	90	5.6
28.8	男性	77	106	
#DIV/0!	男性	67	97	
22.3	1	76	90	******
#DIV/0!	1	59	95	5.3
#DIV/0!	男性	48	102	
#DIV/0!	1	52	76	5
#DIV/0!	男性	67	132	5.7
#DIV/0!	1	74	173	5.7
#DIV/0!	1	64	102	5.3
#DIV/0!	1	62	115	5.8
#DIV/0!	1	62	96	5.3
#DIV/0!	1	69	109	******
#DIV/0!	1	65	113	6
#DIV/0!	男性	60	104	
#DIV/0!	1	67	110	5.6
#DIV/0!	男性	63	111	
23.7	1	77	102	5.6
#DIV/0!	1	86	******	******
#DIV/0!	男性	72	100	
#DIV/0!	1	81	******	5.7
#DIV/0!	1	70	104	******
#DIV/0!	1	67	105	5.6
#DIV/0!	男性	79	97	5.6

23.0	男性	64	145	3.4
#DIV/0!	1	63	******	******
#DIV/0!	1	62	107	******
#DIV/0!	男性	78	95	4.7
#DIV/0!	男性	62	145	5.6
14.2	男性	98	102	5.1
#DIV/0!	男性	76	100	5.2
#DIV/0!	1	63	135	******
#DIV/0!	1	64	******	******
#DIV/0!	1	62	103	
#DIV/0!	1	55	127	5.8
#DIV/0!	男性	75	112	5.1
#DIV/0!	男性	70	94	
#DIV/0!	1	62	******	******
#DIV/0!	男性	49	178	5.2
21.5	男性	55	114	5.7
#DIV/0!	男性	58		
17.9	男性	65	99	5.8
#DIV/0!	1	45	******	******
#DIV/0!	1	82	83	5.9
#DIV/0!	男性	72	117	
22.9	男性	72	95	
#DIV/0!	男性	67	106	5.4
#DIV/0!	男性	83	110	******
#DIV/0!	男性	72	122	5.2
27.0	1	67	116	******
24.3	1	65	102	6
22.2	1	73	109	5.5
#DIV/0!	男性	87	118	
20.0	1	66	101	5
#DIV/0!	男性	83	******	******
18.6	1	74	104	5.9
#DIV/0!	1	75	127	******
#DIV/0!	男性	70	99	4.9
#DIV/0!	男性	68	96	
#DIV/0!	1	61	142	******
#DIV/0!	1	75	******	6.3
26.2	1	71	138	5.8
#DIV/0!	男性	64	88	5
#DIV/0!	1	69	******	******
#DIV/0!	1	78	126	******

#DIV/0!	1	80	153	******
#DIV/0!	男性	76		
#DIV/0!	1	82	110	******
#DIV/0!	男性	76		
#DIV/0!	1	51	91	4.9
#DIV/0!	男性	73	113	5.7
#DIV/0!	1	75	115	******
#DIV/0!	男性	51	96	5.1
#DIV/0!	男性	77	92	
#DIV/0!	男性	56	88	
#DIV/0!	男性	77	118	******
#DIV/0!	男性	80		
#DIV/0!	1	80	******	******
#DIV/0!	1	63	108	5.1
#DIV/0!	1	61	93	******
#DIV/0!	1	47	97	******
25.1	男性	61	104	******
26.9	男性	62	114	
#DIV/0!	男性	69	******	******
#DIV/0!	1	73	******	******
#DIV/0!	1	54	******	******
#DIV/0!	1	64	******	******
#DIV/0!	1	76	******	******
#DIV/0!	1	79	******	******
#DIV/0!	1	71	93	******
#DIV/0!	1	67	******	******
#DIV/0!	男性	63		
#DIV/0!	男性	84		
#DIV/0!	男性	72	118	
#DIV/0!	男性	67	107	5.1
#DIV/0!	男性	60	87	
#DIV/0!	男性	70	109	5.4
#DIV/0!	1	67	96	5
#DIV/0!	男性	85	109	5.6
#DIV/0!	男性	71		
#DIV/0!	男性	62	111	
#DIV/0!	1	86	******	******
#DIV/0!	1	70	******	******
#DIV/0!	1	82	******	******
#DIV/0!	1	56	******	******
#DIV/0!	1	67	******	******

#DIV/0!	1	56	******	******
25.8	1	67	******	******
#DIV/0!	1	31	******	******
#DIV/0!	1	61	******	******
#DIV/0!	1	73	******	******
#DIV/0!	1	62	92	******
24.1	男性	62	106	******
18.2	男性	79	85	******
27.4	1	57	94	5.1
#DIV/0!	1	86	******	******
#DIV/0!	1	44	******	******
#DIV/0!	1	63	109	4.9
#DIV/0!	1	65	97	5.3
#DIV/0!	1	63	105	******
#DIV/0!	1	70	99	******
#DIV/0!	1	68	******	******
#DIV/0!	1	86	******	******
#DIV/0!	1	57	******	******
#DIV/0!	1	64	******	******
#DIV/0!	1	63	106	5.4
21.7	1	60	******	******
#DIV/0!	男性	71		
#DIV/0!	男性	101	114	
#DIV/0!	1	68	106	5.2
#DIV/0!	1	64	117	4.9
22.5	1	61	******	******
#DIV/0!	男性	49		
#DIV/0!	男性	55		
#DIV/0!	1	78	******	******
#DIV/0!	男性	23	106	5.5
#DIV/0!	1	62	******	******
#DIV/0!	男性	81	98	
22.8	男性	87	******	******
#DIV/0!	1	68	******	******
#DIV/0!	1	63	125	5.7
#DIV/0!	1	81	******	******
#DIV/0!	1	77	******	******
#DIV/0!	1	71	******	******
#DIV/0!	男性	69	93	5
25.1	男性	62	124	5.4
#DIV/0!	男性	61	******	******
#DIV/0!	1	72	******	******

#DIV/0!	男性	71	******	******
23.9	1	76	******	******
#DIV/0!	男性	78	111	******
#DIV/0!	1	79	******	******
#DIV/0!	男性	65	108	5.7
#DIV/0!	1	67	******	******
#DIV/0!	1	70	******	******
27.2	男性	51	******	******
#DIV/0!	1	67	130	6
#DIV/0!	1	68	******	******
#DIV/0!	1	71	******	******
#DIV/0!	1	78	******	******
#DIV/0!	1	34	******	******
#DIV/0!	男性	66		5.4
#DIV/0!	男性	67	109	5.6
#DIV/0!	1	73	******	******
#DIV/0!	1	64	88	5.8
#DIV/0!	男性	68	101	4.9
19.3	1	49	101	5.4
#DIV/0!	1	66	******	******
#DIV/0!	1	75	******	5.2
#DIV/0!	男性	53	******	******
#DIV/0!	1	88	******	******
#DIV/0!	1	79	******	******
#DIV/0!	男性	49	******	******
17.7	男性	54	108	
#DIV/0!	1	56	118	5.2
#DIV/0!	1	80	******	******
#DIV/0!	1	35	******	******
#DIV/0!	1	46	114	5.2
#DIV/0!	1	64	105	5.2
#DIV/0!	1	45	******	******
#DIV/0!	男性	38	119	5.4
#DIV/0!	1	77	******	******
#DIV/0!	1	76	******	******
#DIV/0!	1	67	111	5.4
#DIV/0!	男性	59	122	5.8
#DIV/0!	男性	80	101	5.4
#DIV/0!	1	73	******	******
#DIV/0!	1	76	******	******
#DIV/0!	1	71	******	******
#DIV/0!	1	52	111	5.4

#DIV/0!	1	64	******	******
#DIV/0!	男性	60	103	5.2
#DIV/0!	男性	80	150	6.2
#DIV/0!	1	67	******	******
#DIV/0!	男性	71	******	******
#DIV/0!	1	67	******	******
#DIV/0!	1	71	******	******
#DIV/0!	1	57	106	******
#DIV/0!	男性	68	******	******
#DIV/0!	1	93	123	6.1
25.1	男性	51	103	5.3
#DIV/0!	1	65	112	5.6
#DIV/0!	1	84	169	******
#DIV/0!	男性	63	104	5.2
#DIV/0!	男性	59	102	******
#DIV/0!	男性	66	******	5.4
#DIV/0!	1	70	******	******
#DIV/0!	1	46	109	4.8
23.8	1	81	******	******
#DIV/0!	1	58	101	5.9
#DIV/0!	1	67	******	
#DIV/0!	1	62	******	******
#DIV/0!	1	83	106	5.3
28.7	男性	52	96	5.5
#DIV/0!	男性	50	******	******
#DIV/0!	1	74	******	******
#DIV/0!	1	66	94	5.6
#DIV/0!	男性	61	111	4.9
#DIV/0!	男性	39	112	
#DIV/0!	1	74	137	5.9
#DIV/0!	1	69	117	5.4
#DIV/0!	1	42	94	******
#DIV/0!	1	73	101	5.4
#DIV/0!	男性	50	110	
#DIV/0!	1	66	******	******
22.8	男性	68	114	4.5
#DIV/0!	1	59	******	******
#DIV/0!	1	49	102	4.6
#DIV/0!	1	78	******	******
#DIV/0!	1	48	******	******
25.0	1	74	******	4.6
#DIV/0!	男性	64		5.2

#DIV/0!	1	80	******	******
#DIV/0!	1	71	******	******
#DIV/0!	1	48	104	5.3
#DIV/0!	1	78	******	******
#DIV/0!	男性	81	******	******
#DIV/0!	1	73	******	******
22.5	男性	74	99	
#DIV/0!	1	77	******	******
23.9	男性	69	99	4.9
#DIV/0!	男性	66		
21.5	男性	74	110	
#DIV/0!	1	66	128	5.9
#DIV/0!	1	68	125	5.8
#DIV/0!	男性	61	91	5
#DIV/0!	1	85	******	******
#DIV/0!	男性	67	******	******
#DIV/0!	1	79	100	6.3
#DIV/0!	1	66	******	******
27.4	1	39	******	******
#DIV/0!	1	74	******	******
#DIV/0!	男性	30		
#DIV/0!	男性	53	103	******
#DIV/0!	男性	82	97	******
#DIV/0!	1	71	******	******
#DIV/0!	1	63	127	5.6
#DIV/0!	1	75	******	******
#DIV/0!	1	48	******	******
#DIV/0!	1	56	******	******
#DIV/0!	男性	75	******	******
#DIV/0!	1	93	******	******
#DIV/0!	1	78	******	******
#DIV/0!	1	73	******	******
#DIV/0!	男性	63	100	5.3
#DIV/0!	1	61	******	******
#DIV/0!	1	59	******	******
#DIV/0!	1	83	******	******
#DIV/0!	1	61	******	******
#DIV/0!	1	79	******	******
#DIV/0!	1	62	******	******
#DIV/0!	1	66	99	******
#DIV/0!	1	56	******	******
#DIV/0!	1	68	******	******

#DIV/0!	男性	43	111	5.5
#DIV/0!	1	43	******	******
19.1	1	78	******	******
#DIV/0!	1	78	******	******
21.5	1	69	******	******
26.0	1	53	******	******
#DIV/0!	1	60	******	******
#DIV/0!	1	77	******	******
#DIV/0!	1	45	105	******
#DIV/0!	男性	41	94	******
#DIV/0!	男性	80	******	******
#DIV/0!	1	76	******	******
#DIV/0!	1	64	******	******
#DIV/0!	1	61	******	******
#DIV/0!	1	44	114	******
#DIV/0!	1	74	******	******
#DIV/0!	1	54	******	5
#DIV/0!	男性	75	95	5.4
#DIV/0!	1	73	******	******
#DIV/0!	男性	72	114	5
#DIV/0!	1	50	******	******
#DIV/0!	1	62	******	******
#DIV/0!	男性	79		
#DIV/0!	男性	58	95	5.5
#DIV/0!	男性	64	89	
#DIV/0!	1	52	******	******
#DIV/0!	男性	40	103	4.9
#DIV/0!	男性	63	******	5
#DIV/0!	1	61	******	******
#DIV/0!	1	79	******	******
#DIV/0!	男性	70	124	5.1
#DIV/0!	1	78	******	******
#DIV/0!	1	68	******	******
#DIV/0!	男性	61	104	******
#DIV/0!	1	84	******	******
#DIV/0!	男性	63	******	******
#DIV/0!	1	45	105	5.7
#DIV/0!	男性	63	130	
#DIV/0!	1	78	******	******
#DIV/0!	男性	34	101	
#DIV/0!	1	49	******	******
#DIV/0!	1	87	******	******

#DIV/0!	1	77	100	5.3
#DIV/0!	1	73	******	******
#DIV/0!	1	64	******	******
25.4	1	67	87	******
#DIV/0!	男性	61		
#DIV/0!	1	44	******	******
#DIV/0!	男性	78	112	******
#DIV/0!	1	36	******	******
#DIV/0!	1	37	96	5.2
#DIV/0!	男性	60		
#DIV/0!	1	81	108	******
#DIV/0!	男性	71	104	5.9
23.6	男性	58	104	
22.7	男性	79	102	5.6
#DIV/0!	男性	59	120	5.4
#DIV/0!	男性	79	110	5.7
#DIV/0!	1	79	******	******
#DIV/0!	1	70	89	5
#DIV/0!	1	65	108	******
#DIV/0!	男性	51		5.8
#DIV/0!	1	69	******	******
#DIV/0!	1	49	******	******
#DIV/0!	1	58	******	******
#DIV/0!	1	58	******	******
#DIV/0!	1	74	******	******
#DIV/0!	1	60	******	******
#DIV/0!	1	77	******	******
#DIV/0!	1	52	106	5.6
#DIV/0!	1	53	102	5.3
#DIV/0!	1	56	******	******
#DIV/0!	男性	43	105	4.9
#DIV/0!	1	56	107	******
#DIV/0!	1	65	******	******
#DIV/0!	男性	57	109	
#DIV/0!	1	62	******	******
#DIV/0!	1	70	******	******
#DIV/0!	男性	60	92	******
#DIV/0!	1	70	******	******
#DIV/0!	1	77	******	******
#DIV/0!	男性	67	108	******
#DIV/0!	1	62	******	******
30.3	1	58	101	5.2

#DIV/0!	1	54	128	6
#DIV/0!	1	79	******	******
#DIV/0!	1	64	******	******
#DIV/0!	1	63	******	******
#DIV/0!	1	50	******	******
#DIV/0!	1	72	******	******
#DIV/0!	1	56	******	******
#DIV/0!	男性	70	******	******
#DIV/0!	1	52	******	******
#DIV/0!	男性	56	111	5.3
#DIV/0!	男性	59	122	5.2
15.5	男性	62	155	5.5
#DIV/0!	男性	65	103	
#DIV/0!	1	62	101	******
#DIV/0!	1	74	******	******
#DIV/0!	1	68	******	******
20.9	男性	50	101	
#DIV/0!	1	71	******	******
#DIV/0!	1	55	96	4.8
#DIV/0!	1	75	******	******
#DIV/0!	男性	65	105	5.6
#DIV/0!	1	57	144	6.1
#DIV/0!	1	89	******	******
#DIV/0!	男性	77		
#DIV/0!	1	80	******	******
#DIV/0!	1	62	115	5.6
#DIV/0!	1	62	120	5.4
#DIV/0!	1	48	******	******
#DIV/0!	1	34	******	******
#DIV/0!	1	52	******	******
#DIV/0!	1	68	******	******
#DIV/0!	1	61	******	******
#DIV/0!	1	44	******	******
#DIV/0!	男性	80		
#DIV/0!	1	82	******	******
#DIV/0!	1	76	******	******
#DIV/0!	1	75	******	******
#DIV/0!	男性	73	104	******
21.6	1	79	98	5.1
#DIV/0!	男性	67	99	5.3
#DIV/0!	男性	56	******	******
#DIV/0!	男性	69	109	5.4

#DIV/0!	男性	51	98	******
#DIV/0!	男性	52	84	
#DIV/0!	1	58	******	******
#DIV/0!	1	60	******	******
#DIV/0!	1	57	129	5.6
#DIV/0!	1	72	119	******
#DIV/0!	男性	78	106	******
24.9	1	49	******	******
#DIV/0!	男性	37		
#DIV/0!	1	54	******	******
#DIV/0!	1	68	******	******
#DIV/0!	1	73	******	******
#DIV/0!	1	28	******	******
#DIV/0!	1	30	******	******
#DIV/0!	男性	62		
24.2	男性	74	102	5.5
#DIV/0!	1	68	100	5.7
#DIV/0!	1	57	******	******
#DIV/0!	1	66	******	******
22.5	男性	81		
#DIV/0!	1	58	******	******
#DIV/0!	1	77	******	******
#DIV/0!	1	80	******	******
#DIV/0!	男性	88	132	
#DIV/0!	1	83	******	******
#DIV/0!	男性	69	114	5.7
#DIV/0!	1	76	******	******
#DIV/0!	1	69	******	******
24.1	1	71	******	******
#DIV/0!	男性	73		
#DIV/0!	1	82	******	******
#DIV/0!	1	27	******	******
#DIV/0!	1	72	******	******
#DIV/0!	1	63	******	******
#DIV/0!	1	61	******	******
#DIV/0!	1	39	******	******
#DIV/0!	1	47	******	******
#DIV/0!	男性	63		
#DIV/0!	1	71	******	******
26.1	男性	54		
#DIV/0!	1	65	122	5.1
#DIV/0!	1	49	85	4.8

#DIV/0!	1	64	******	******
#DIV/0!	1	78	152	******
#DIV/0!	1	71	******	******
#DIV/0!	1	70	******	******
#DIV/0!	1	57	******	******
#DIV/0!	男性	63	100	
#DIV/0!	1	75	106	4.9
#DIV/0!	男性	44	119	******
#DIV/0!	1	83	******	******
#DIV/0!	1	77	106	6
#DIV/0!	男性	70	89	
#DIV/0!	1	72	110	6
#DIV/0!	1	62	******	******
#DIV/0!	男性	51	******	******
23.9	1	72	******	******
21.7	1	69	104	5.7
#DIV/0!	1	67	******	******
#DIV/0!	1	78	******	******
#DIV/0!	1	44	139	5.8
#DIV/0!	男性	56	106	******
#DIV/0!	1	51	******	******
#DIV/0!	男性	27		
#DIV/0!	男性	27	******	******
#DIV/0!	1	69	******	******
#DIV/0!	1	73	******	******
#DIV/0!	男性	72	102	5.3
#DIV/0!	男性	62	97	5.1
#DIV/0!	男性	49		
#DIV/0!	1	67	******	******
#DIV/0!	1	74	******	******
#DIV/0!	1	53	******	******
19.3	男性	88	129	5.1
18.7	男性	71	103	5.4
#DIV/0!	1	77	******	******
#DIV/0!	男性	44	99	5.2
#DIV/0!	1	58	106	4.8
#DIV/0!	男性	33	******	******
#DIV/0!	1	53	******	******
#DIV/0!	1	48	91	5.3
23.0	1	28	106	4.9
#DIV/0!	1	79	******	******
#DIV/0!	男性	60	108	******

#DIV/0!	1	76	******	******
#DIV/0!	男性	83		
#DIV/0!	1	43	******	******
#DIV/0!	1	60	******	******
#DIV/0!	1	79	108	6
26.3	男性	63	******	5.7
#DIV/0!	1	57	119	5.5
23.2	男性	75	******	5.5
#DIV/0!	男性	54	115	6.1
18.8	男性	62	76	******
#DIV/0!	1	55	******	******
#DIV/0!	1	36	113	5.7
#DIV/0!	男性	69	******	******
#DIV/0!	男性	58	121	6.1
20.1	男性	85	89	
#DIV/0!	1	83	******	******
#DIV/0!	男性	63	105	4.9
#DIV/0!	1	47	******	******
#DIV/0!	男性	63	108	
#DIV/0!	男性	41	98	
24.3	1	76	******	******
20.8	男性	63		
25.5	男性	56		
#DIV/0!	1	62	******	******
#DIV/0!	1	85	******	******
23.5	1	76	******	******
#DIV/0!	男性	54		
29.4	1	66	******	6.2
#DIV/0!	1	74	117	5.6
#DIV/0!	1	76	******	******
#DIV/0!	1	77	******	******
23.2	1	61	******	******
#DIV/0!	1	84	******	******
22.5	男性	70	******	******
#DIV/0!	1	56	******	******
#DIV/0!	男性	68	107	******
#DIV/0!	1	49	******	******
23.6	1	62	******	******
#DIV/0!	男性	40	******	4.8
#DIV/0!	男性	46		5.4
#DIV/0!	1	55	******	******
#DIV/0!	1	85	108	5.2

#DIV/0!	1	70	******	******
#DIV/0!	1	37	108	4.5
25.7	男性	60	120	6.2
#DIV/0!	男性	76	******	******
#DIV/0!	1	59	******	******
#DIV/0!	1	47	******	******
#DIV/0!	1	64	******	******
#DIV/0!	1	50	89	4.8
#DIV/0!	1	37	******	******
#DIV/0!	1	37	******	******
#DIV/0!	1	54	******	******
#DIV/0!	1	68	******	******
#DIV/0!	1	64	******	******
#DIV/0!	男性	51	******	******
#DIV/0!	1	50	******	******
#DIV/0!	1	62	******	******
#DIV/0!	男性	53	97	******
#DIV/0!	1	59	112	5.7
#DIV/0!	1	36	88	4.8
#DIV/0!	1	71	******	******
#DIV/0!	1	93	******	******
#DIV/0!	1	47	87	4.9
#DIV/0!	1	49	92	4.9
#DIV/0!	1	40	******	******
#DIV/0!	1	50	******	******
#DIV/0!	男性	46		
#DIV/0!	男性	61		
20.9	男性	42	******	******
19.9	1	60	******	******
#DIV/0!	男性	72	90	6.2
#DIV/0!	1	39	******	******
#DIV/0!	1	45	******	******
#DIV/0!	1	66	******	******
#DIV/0!	1	69	******	******
#DIV/0!	1	50	******	******
#DIV/0!	1	76	******	******
#DIV/0!	1	45	******	******
21.9	1	75	******	******
#DIV/0!	1	68	******	******
28.9	男性	68	101	
#DIV/0!	男性	79	131	
#DIV/0!	男性	68	118	******

19.8	1	83	******	******
#DIV/0!	1	61	103	5.5
#DIV/0!	1	52	124	5
#DIV/0!	男性	47		
#DIV/0!	男性	67	******	******
#DIV/0!	1	58	******	******
#DIV/0!	1	62	******	******
#DIV/0!	1	86	******	******
20.4	1	57	******	******
21.8	男性	62	105	5.5
#DIV/0!	1	68	******	******
#DIV/0!	1	52	92	5.3
#DIV/0!	1	74	******	******
#DIV/0!	1	28	******	******
#DIV/0!	1	44	112	5.2
#DIV/0!	1	62	******	******
#DIV/0!	1	61	******	******
23.1	1	88	******	******
#DIV/0!	1	27	******	******
33.3	男性	66	129	
24.2	男性	58	107	
#DIV/0!	男性	69	111	******
#DIV/0!	1	55	******	******
#DIV/0!	1	67	******	******
#DIV/0!	1	80	******	******
#DIV/0!	男性	53	126	
#DIV/0!	1	49	107	5.4
23.5	男性	69	97	5.3
#DIV/0!	男性	51	108	5.4
#DIV/0!	1	65	******	******
#DIV/0!	男性	60	96	5.5
#DIV/0!	1	74	******	******
22.0	1	66	******	******
#DIV/0!	男性	35	89	4.9
#DIV/0!	男性	63	105	5.1
#DIV/0!	男性	60	111	
#DIV/0!	男性	77	******	******
#DIV/0!	1	52	******	******
#DIV/0!	1	64	******	******
#DIV/0!	1	72	******	******
22.7	1	67	******	******
19.2	男性	63	103	5

#DIV/0!	1	58	97	******
#DIV/0!	1	76	123	******
#DIV/0!	1	57	******	******
#DIV/0!	1	64	******	5.4
#DIV/0!	男性	67	96	
#DIV/0!	男性	75	104	
21.1	1	58	100	******
#DIV/0!	男性	49	106	
#DIV/0!	男性	33	104	5.2
#DIV/0!	男性	52	103	5
#DIV/0!	男性	56	93	4.9
#DIV/0!	1	51	******	******
23.6	男性	32		
23.7	男性	31	98	4.9
#DIV/0!	男性	81	104	
#DIV/0!	1	62	98	5.5
#DIV/0!	1	38	******	******
#DIV/0!	1	63	******	******
#DIV/0!	男性	41	99	5.2
21.0	男性	57	100	6.1
21.5	1	71	95	5.2
26.0	男性	65	103	4.9
22.2	男性	45	103	5.1
26.3	男性	41	102	5.5
#DIV/0!	男性	50		
#DIV/0!	1	58	98	5.6
#DIV/0!	1	55	112	5.4
#DIV/0!	1	62	******	5.1
#DIV/0!	男性	59		6
#DIV/0!	1	39	******	******
#DIV/0!	1	37	******	******
#DIV/0!	男性	74		
24.8	1	54	93	5.3
#DIV/0!	1	55	******	******
15.8	1	69	******	******
26.7	男性	73		
22.8	1	60	100	******
#DIV/0!	1	56	102	******
#DIV/0!	1	73	******	******
#DIV/0!	1	66	******	******
20.4	1	41	******	******

20.7	1	67	******	******
23.2	1	63	******	******
24.4	1	41	111	5.5
#DIV/0!	男性	63	95	5.1
25.1	男性	47	106	5.1
22.3	男性	56	96	5.2
#DIV/0!	1	60	******	******
#DIV/0!	1	62	******	******
#DIV/0!	1	76	******	******
#DIV/0!	1	78	******	******
#DIV/0!	男性	69		
#DIV/0!	1	63	******	******
#DIV/0!	1	69	106	5.1
#DIV/0!	1	63	******	******
23.9	1	57	******	******
#DIV/0!	1	67	108	5.6
#DIV/0!	1	75	******	******
#DIV/0!	男性	63	76	5.9
#DIV/0!	男性	77	121	******
#DIV/0!	男性	68	105	5.8
#DIV/0!	1	46	99	4.8
21.9	男性	58	104	5.2
21.9	男性	44	78	4.8
24.7	1	37	102	4.9
#DIV/0!	1	57	******	******
#DIV/0!	1	38	100	4.7
#DIV/0!	1	67	117	5.1
25.0	1	70	******	******
#DIV/0!	1	64	******	******
#DIV/0!	男性	43	100	5.2
#DIV/0!	1	36	95	4.8
#DIV/0!	1	67	******	******
#DIV/0!	男性	81		
#DIV/0!	1	44	******	5
#DIV/0!	男性	61	117	5.4
17.5	1	64	91	******
#DIV/0!	1	42	******	******
21.3	1	58	120	6.1
#DIV/0!	1	41	******	******
#DIV/0!	1	62	87	5
23.7	男性	69	104	5.3
27.2	1	75	92	5.6

25.0	1	59	103	5.4
#DIV/0!	1	56	113	******
#DIV/0!	1	47	80	5
24.0	1	49	******	******
#DIV/0!	男性	65	108	5.1
#DIV/0!	男性	62	107	5.1
#DIV/0!	男性	58	92	5.2
#DIV/0!	1	55	114	5.3
#DIV/0!	男性	75	143	
#DIV/0!	男性	61	109	5.4
#DIV/0!	男性	46		
#DIV/0!	1	66	******	7.6
#DIV/0!	1	76	******	******
#DIV/0!	1	45	******	******
#DIV/0!	1	78	******	******
#DIV/0!	1	66	******	******
19.6	1	86	******	******
#DIV/0!	1	57	******	******
22.5	男性	77		
20.4	1	62	264	6.2
#DIV/0!	男性	57		
#DIV/0!	1	79	******	******
#DIV/0!	男性	63		
#DIV/0!	1	75	102	******
#DIV/0!	1	63	******	******
#DIV/0!	1	58	******	******
#DIV/0!	1	38	******	******
#DIV/0!	1	67	******	******
#DIV/0!	1	52	******	******
#DIV/0!	男性	61	119	5.4
25.5	男性	38	87	4.7
26.2	1	50	94	5.4
#DIV/0!	1	52	95	5
#DIV/0!	1	49	******	******
#DIV/0!	1	49	******	******
#DIV/0!	男性	39	92	5
#DIV/0!	1	39	94	5.1
#DIV/0!	1	41	100	4.6
#DIV/0!	1	61	119	5.6
#DIV/0!	1	62	******	******
#DIV/0!	男性	45	116	
#DIV/0!	1	77	******	******

86.9	1	46	118	5
#DIV/0!	1	51	106	******
#DIV/0!	1	72	98	******
16.7	1	76	******	******
#DIV/0!	1	59	110	5.4
#DIV/0!	1	81	******	******
#DIV/0!	1	52	******	******
#DIV/0!	1	67	******	******
#DIV/0!	1	35	******	******
#DIV/0!	1	42	******	******
#DIV/0!	男性	61		
#DIV/0!	1	73	******	******
#DIV/0!	男性	61	82	******
#DIV/0!	1	64	******	******
#DIV/0!	1	54	125	5.7
#DIV/0!	1	79	******	******
#DIV/0!	1	63	100	5.2
#DIV/0!	1	53	******	******
#DIV/0!	1	67	******	******
#DIV/0!	1	80	105	******
#DIV/0!	1	62	129	******
#DIV/0!	1	60	125	5.3
#DIV/0!	1	64	******	******
#DIV/0!	1	82	******	******
#DIV/0!	1	58	******	******
#DIV/0!	1	81	******	******
#DIV/0!	1	51	******	******
#DIV/0!	1	71	******	******
#DIV/0!	男性	34	******	******
#DIV/0!	男性	41		
#DIV/0!	1	46	******	******
#DIV/0!	1	63	******	******
#DIV/0!	1	57	******	4.9
23.0	男性	57	103	5.4
16.8	1	59	******	******
23.6	男性	74	97	5.3
#DIV/0!	1	68	******	******
#DIV/0!	1	59	******	******
28.0	男性	46	107	5.1
#DIV/0!	1	40	******	5.1
#DIV/0!	1	61	******	******
#DIV/0!	男性	47	99	5.2

#DIV/0!	1	87	******	******
#DIV/0!	男性	51	131	5.7
#DIV/0!	男性	35	85	
#DIV/0!	1	40	99	******
14.6	男性	74	99	
#DIV/0!	1	64	******	******
#DIV/0!	男性	82	92	6.3
#DIV/0!	1	63	******	******
#DIV/0!	1	72	******	******
#DIV/0!	男性	77	101	5.1
#DIV/0!	男性	37	******	******
#DIV/0!	1	51	112	******
#DIV/0!	1	85	83	5.3
#DIV/0!	男性	79	143	5.2
25.5	1	59	109	6.4
#DIV/0!	1	71	******	******
#DIV/0!	1	64	******	******
#DIV/0!	男性	46	89	******
#DIV/0!	1	64	******	******
#DIV/0!	1	76	******	******
#DIV/0!	1	62	******	******
#DIV/0!	1	72	126	******
#DIV/0!	男性	56	******	5.5
#DIV/0!	1	35	92	5
#DIV/0!	1	50	109	5.1
#DIV/0!	1	76	******	******
21.3	男性	44	83	******
#DIV/0!	1	72	******	******
29.0	1	76	******	******
#DIV/0!	1	55	******	******
22.5	1	56	102	******
#DIV/0!	1	70	79	******
#DIV/0!	1	84	******	******
22.3	男性	69	90	4.9
#DIV/0!	男性	68	102	
#DIV/0!	1	62	******	******
21.5	1	65	******	******
#DIV/0!	1	29	******	******
#DIV/0!	男性	70	******	******
#DIV/0!	男性	36	176	5.8
#DIV/0!	1	92	******	******
#DIV/0!	1	61	******	******

#DIV/0!	1	33	******	5
#DIV/0!	1	80	******	******
#DIV/0!	1	57	******	******
#DIV/0!	1	79	******	******
#DIV/0!	男性	69	95	******
#DIV/0!	1	47	117	******
#DIV/0!	1	63	******	******
#DIV/0!	1	51	******	5.3
#DIV/0!	1	71	******	******
#DIV/0!	男性	34		
#DIV/0!	1	76	******	******
#DIV/0!	男性	42		
#DIV/0!	1	59	******	******
#DIV/0!	1	71	******	******
28.7	男性	32	130	
#DIV/0!	1	39	******	******
#DIV/0!	1	54	112	******
#DIV/0!	1	61	******	******
#DIV/0!	1	44	******	******
32.4	1	25	******	******
#DIV/0!	1	79	******	******
#DIV/0!	1	85	******	4.9
#DIV/0!	男性	54	94	******
#DIV/0!	男性	53	100	
#DIV/0!	1	53	******	******
#DIV/0!	1	59	******	******
#DIV/0!	男性	63	131	******
#DIV/0!	1	70	******	******
#DIV/0!	1	87	******	******
#DIV/0!	1	58	******	******
#DIV/0!	1	37	******	******
#DIV/0!	1	75	107	4.8
#DIV/0!	1	64	******	******
#DIV/0!	1	25	116	4.8
#DIV/0!	1	49	******	******
21.2	1	77	******	******
#DIV/0!	1	22	******	******
22.9	1	84	******	******
20.7	1	74	150	5.9
#DIV/0!	1	64	******	******
23.1	1	77	******	******
#DIV/0!	男性	55	111	

#DIV/0!	1	60	******	******
#DIV/0!	1	60	******	******
#DIV/0!	1	84	******	5.6
#DIV/0!	1	37	******	******
#DIV/0!	男性	62	******	******
#DIV/0!	1	32	******	******
#DIV/0!	男性	62	78	******
#DIV/0!	1	64	113	******
#DIV/0!	1	47	******	******
#DIV/0!	1	57	******	******
19.6	1	97	******	******
#DIV/0!	1	43	273	******
#DIV/0!	男性	35	102	******
#DIV/0!	1	63	******	******
#DIV/0!	1	63	91	5.1
#DIV/0!	1	35	89	4.7
#DIV/0!	1	53	******	******
#DIV/0!	1	67	******	******
21.7	1	50	******	******
27.7	1	52	******	******
#DIV/0!	1	54	******	******
#DIV/0!	1	31	95	5
#DIV/0!	1	44	******	******
#DIV/0!	1	49	******	******
27.8	男性	59	106	5.1
#DIV/0!	男性	39	100	5.3
21.8	男性	68	115	5.5
#DIV/0!	1	60	******	******
#DIV/0!	1	60	96	******
#DIV/0!	1	28	******	******
#DIV/0!	1	65	******	******
#DIV/0!	1	37	******	******
#DIV/0!	1	59	130	6
#DIV/0!	1	34	129	6
#DIV/0!	1	26	******	******
#DIV/0!	男性	52	******	******
#DIV/0!	1	50	122	5.2
#DIV/0!	1	37	******	******
#DIV/0!	1	29	******	******
#DIV/0!	1	58	******	******
#DIV/0!	1	47	******	******
#DIV/0!	1	72	******	******
21.5	1	51	******	******

#DIV/0!	1	53	******	******
#DIV/0!	男性	46	98	4.6
#DIV/0!	1	53	******	******
18.8	1	79	125	5.3
#DIV/0!	1	36	******	******
#DIV/0!	1	67	******	******
#DIV/0!	1	25	******	******
#DIV/0!	1	43	******	******
#DIV/0!	1	80	******	******
#DIV/0!	男性	36	184	5.4
#DIV/0!	1	38	******	******
#DIV/0!	1	73	******	******
#DIV/0!	1	36	******	******
#DIV/0!	1	62	******	******
#DIV/0!	1	50	******	******
#DIV/0!	1	58	******	******
#DIV/0!	1	34	101	4.9
#DIV/0!	1	56	******	******
#DIV/0!	1	80	******	******
#DIV/0!	1	42	******	******
#DIV/0!	1	64	******	******
#DIV/0!	1	63	110	******
#DIV/0!	1	62	78	5.4
21.5	男性	61	******	******
#DIV/0!	1	72	******	5.5
#DIV/0!	1	58	******	******
#DIV/0!	1	57	******	******
#DIV/0!	1	65	107	5.5
#DIV/0!	1	27	******	******
#DIV/0!	1	56	******	******
#DIV/0!	1	67	******	******
#DIV/0!	男性	81	******	******
#DIV/0!	1	49	******	******
#DIV/0!	1	66	99	******
#DIV/0!	男性	76		
17.7	男性	73		5.1
#DIV/0!	男性	48	113	******
23.1	男性	81	116	******
#DIV/0!	男性	59	******	******
20.0	男性	79	94	5.3
#DIV/0!	男性	35	******	******
#DIV/0!	男性	40	******	******

#DIV/0!	男性	38	108	******
#DIV/0!	男性	67	126	******
#DIV/0!	男性	70		
#DIV/0!	男性	63		
25.3	男性	54		
#DIV/0!	男性	68		
#DIV/0!	男性	64	126	******
#DIV/0!	男性	23	109	******
22.8	男性	64	116	5.3
#DIV/0!	男性	42	******	******
#DIV/0!	男性	53	103	5.2
#DIV/0!	男性	32	101	******
21.6	男性	39	94	4.8
#DIV/0!	男性	81		
#DIV/0!	男性	61	115	
#DIV/0!	男性	56	165	******
#DIV/0!	男性	62	121	5.9
#DIV/0!	男性	54	85	******
#DIV/0!	男性	79	137	
#DIV/0!	男性	29	106	
#DIV/0!	男性	73		
#DIV/0!	男性	71	102	******
#DIV/0!	男性	61	162	
#DIV/0!	男性	70	116	4.9
#DIV/0!	男性	48	91	5.1
#DIV/0!	男性	42	94	5.9
#DIV/0!	男性	24	91	4.9
#DIV/0!	男性	53	******	******
#DIV/0!	男性	67	98	******
#DIV/0!	男性	60	135	5.1
#DIV/0!	男性	71	113	
#DIV/0!	男性	47	******	******
#DIV/0!	男性	70	101	
#DIV/0!	男性	65		6.3
21.8	男性	73	95	
#DIV/0!	男性	57	99	
19.7	男性	79		5.6
23.2	男性	85	99	4.2
#DIV/0!	男性	62		
21.5	男性	72	108	4.8

#DIV/0!	男性	66	93	
#DIV/0!	男性	26	127	
#DIV/0!	男性	66	85	
#DIV/0!	男性	78	105	5.5
21.2	男性	59	90	4.6
#DIV/0!	男性	58	95	
#DIV/0!	男性	46		5.1
#DIV/0!	男性	46	100	
#DIV/0!	男性	47	105	
#DIV/0!	男性	45	102	
#DIV/0!	男性	69	161	5.9
#DIV/0!	男性	45	99	5
#DIV/0!	男性	65	82	4.3
#DIV/0!	男性	70	112	5
#DIV/0!	男性	68		5
#DIV/0!	男性	71	135	4.3
#DIV/0!	男性	59	103	
#DIV/0!	男性	55	98	
#DIV/0!	男性	44	104	
20.9	男性	52	100	4.7
#DIV/0!	男性	56	95	5.4
#DIV/0!	男性	53	113	
#DIV/0!	男性	73	112	
#DIV/0!	男性	36		
#DIV/0!	男性	19	127	
#DIV/0!	男性	74	83	4.8
#DIV/0!	男性	86	81	
#DIV/0!	男性	43	175	
#DIV/0!	男性	70	115	6.3
#DIV/0!	男性	65	130	5.7
#DIV/0!	男性	37		
#DIV/0!	男性	31	99	
#DIV/0!	男性	47	102	
#DIV/0!	男性	48	97	5
#DIV/0!	男性	71	88	5.2
22.5	男性	64	170	5.5
#DIV/0!	男性	62	112	
#DIV/0!	男性	86	116	5.4

#DIV/0!	男性	43	101		
#DIV/0!	男性	70			
#DIV/0!	男性	48	108		
#DIV/0!	男性	28	127		
#DIV/0!	男性	84	114	5	
#DIV/0!	男性	35	99		
#DIV/0!	男性	67	126		
#DIV/0!	男性	48	116		
#DIV/0!	男性	28	78		
#DIV/0!	男性	30	184		
#DIV/0!	男性	56			
#DIV/0!	男性	78	121	6	
#DIV/0!	1	69	******	******	
20.1	男性	65	125	5.1	
#DIV/0!	1	48	117	5.4	
#DIV/0!	男性	75	94	******	
23.5	1	74	******	******	
14.2	1	91	******	4.1	
#DIV/0!	1	71	******	******	
#DIV/0!	男性	79	113		
#DIV/0!	男性	70		5.1	
#DIV/0!	1	59	******	******	
#DIV/0!	1	78	106	5.2	
27.6	1	44	******	5.1	
#DIV/0!	男性	62			
#DIV/0!	1	62	******	******	
#DIV/0!	1	48	******	4.8	
#DIV/0!	1	32	98	******	
#DIV/0!	1	67	******	******	
#DIV/0!	1	42	******	******	
26.3	1	66	******	******	
#DIV/0!	1	63	******	******	
16.9	男性	90	113	5.5	
25.3	男性	62	139	5.7	
#DIV/0!	男性	60	112	5.7	
#DIV/0!	1	59	******	******	
#DIV/0!	1	51	93	5.1	
#DIV/0!	1	62	******	5.4	
#DIV/0!	1	64	******	******	
#DIV/0!	男性	54	89	******	
#DIV/0!	男性	67	92	4.5	

#DIV/0!	男性	54	98	******
29.8	男性	58	******	5.6
#DIV/0!	1	53	******	******
#DIV/0!	男性	46	114	
#DIV/0!	1	76	******	******
#DIV/0!	1	51	******	******
#DIV/0!	1	46	******	******
#DIV/0!	1	71	******	******
#DIV/0!	男性	51	99	******
#DIV/0!	1	56	116	5.4
#DIV/0!	1	69	122	5.6
#DIV/0!	1	55	101	4.7
#DIV/0!	1	76	******	******
23.9	1	59	98	******
28.7	男性	62	103	******
23.5	男性	66	99	******
21.0	1	64	162	6.3
#DIV/0!	男性	76		
#DIV/0!	1	75	132	6.2
#DIV/0!	1	73	******	******
#DIV/0!	男性	66		
#DIV/0!	1	33	******	******
#DIV/0!	1	84	******	******
#DIV/0!	1	76	******	******
#DIV/0!	1	43	******	******
#DIV/0!	1	81	98	5.3
28.1	1	65	100	******
#DIV/0!	男性	65	165	
26.1	男性	51	120	5.4
24.3	1	85	******	5.3
#DIV/0!	1	28	******	******
#DIV/0!	男性	48	104	4.9
#DIV/0!	1	49	******	******
#DIV/0!	男性	51	102	5.7
#DIV/0!	1	78	114	******
#DIV/0!	1	60	******	******
#DIV/0!	1	73	91	******
#DIV/0!	1	49	******	******
#DIV/0!	1	64	96	******
#DIV/0!	1	69	120	******
#DIV/0!	1	42	101	******
#DIV/0!	1	67	102	5.1

#DIV/0!	1	56	105	5.3
#DIV/0!	男性	77		5.3
#DIV/0!	男性	58	99	******
#DIV/0!	男性	54	111	5.9
#DIV/0!	男性	78		
#DIV/0!	男性	71	182	
#DIV/0!	男性	47	112	5.4
#DIV/0!	男性	85	115	
#DIV/0!	男性	66	123	
#DIV/0!	男性	57	113	
22.0	男性	62	93	
#DIV/0!	男性	65	137	
#DIV/0!	男性	53	116	4.9
#DIV/0!	男性	76	98	
#DIV/0!	男性	72	105	4.7
20.0	男性	60	112	5.2
#DIV/0!	1	62	******	******
#DIV/0!	1	68	******	******
#DIV/0!	男性	73	108	4.1
#DIV/0!	男性	66	99	******
17.9	男性	69	159	******
#DIV/0!	1	73	******	******
#DIV/0!	1	56	******	******
#DIV/0!	1	62	137	******
18.8	男性	70	139	
#DIV/0!	1	58	******	******
#DIV/0!	男性	76	105	4.9
#DIV/0!	1	67	******	******
#DIV/0!	男性	68	93	
#DIV/0!	1	64	114	5.4
#DIV/0!	男性	54	116	******
22.1	1	43	******	******
25.8	1	47	112	5.1
#DIV/0!	1	59	******	******
#DIV/0!	1	54	96	5
#DIV/0!	1	63	******	******
#DIV/0!	1	70	144	5.5
#DIV/0!	1	64	118	5
#DIV/0!	男性	65	84	5.3
#DIV/0!	1	56	******	******
#DIV/0!	1	60	106	5.1

23.6	男性	73	143	4.7
#DIV/0!	1	63	******	******
#DIV/0!	男性	64	101	
#DIV/0!	1	60	******	******
#DIV/0!	男性	41	118	5.7
#DIV/0!	1	61	******	******
#DIV/0!	1	63	******	******
26.4	男性	72		
#DIV/0!	男性	49	88	
#DIV/0!	男性	58	131	4.1
26.0	1	64	******	******
#DIV/0!	男性	63	148	4.9
#DIV/0!	1	64	******	******
22.3	男性	57	76	5
#DIV/0!	1	82	******	******
#DIV/0!	1	82	80	4.6
#DIV/0!	男性	76	******	******
#DIV/0!	男性	76		
19.2	1	71	105	5.5
#DIV/0!	男性	67	107	5.9
#DIV/0!	男性	56	******	******
#DIV/0!	男性	58	149	5.7
#DIV/0!	男性	60	104	5.1
#DIV/0!	男性	75	101	5.3
#DIV/0!	男性	69		
#DIV/0!	男性	63	107	5.2
#DIV/0!	男性	65	112	4.8
#DIV/0!	男性	41	85	4.2
#DIV/0!	男性	58	******	******
#DIV/0!	男性	60	******	******
#DIV/0!	男性	76	83	5
#DIV/0!	1	57	81	5
#DIV/0!	1	31	******	******
#DIV/0!	男性	54	******	******
#DIV/0!	男性	71	******	******
#DIV/0!	1	63	******	******
#DIV/0!	男性	69	******	******
#DIV/0!	1	41	******	******
#DIV/0!	1	62	48	******
#DIV/0!	男性	50	******	******
13.2	1	60	******	******

#DIV/0!	1	52	97	******
#DIV/0!	男性	86	120	5.3
#DIV/0!	1	59	180	4.8
#DIV/0!	男性	69		
#DIV/0!	1	69	141	5.2
#DIV/0!	男性	59	101	5
#DIV/0!	男性	59	******	******
#DIV/0!	男性	60	110	4.9
#DIV/0!	男性	73		
#DIV/0!	男性	71		
#DIV/0!	男性	50		
#DIV/0!	男性	61	109	
#DIV/0!	男性	70	93	5
#DIV/0!	男性	47		
#DIV/0!	1	73	88	6.1
25.1	男性	67	102	******
#DIV/0!	男性	66	111	5.2
#DIV/0!	1	74	******	******
29.3	1	68	******	5.1
28.3	1	62	******	******
#DIV/0!	1	78	81	******
#DIV/0!	1	76	******	******
#DIV/0!	1	75	******	5.3
#DIV/0!	1	84	******	******
#DIV/0!	1	68	******	******
#DIV/0!	1	69	******	******
#DIV/0!	男性	72	107	5.9
#DIV/0!	1	64	******	******
19.9	1	82	******	******
#DIV/0!	1	68	111	******
#DIV/0!	男性	64	107	******
#DIV/0!	1	60	******	******
27.3	1	69	******	******
#DIV/0!	1	67	124	******
19.9	1	83	98	4.9
#DIV/0!	1	63	******	******
23.3	男性	71	91	5.3
#DIV/0!	1	57	******	******
#DIV/0!	1	66	******	******
#DIV/0!	男性	68	112	******
#DIV/0!	1	64	******	******
#DIV/0!	男性	70	******	4.7

#DIV/0!	1	79	******	******
#DIV/0!	1	60	******	******
#DIV/0!	1	63	96	******
#DIV/0!	1	78	******	******
#DIV/0!	1	62	******	******
#DIV/0!	男性	76	115	******
#DIV/0!	1	77	******	******
#DIV/0!	1	75	******	******
#DIV/0!	1	73	******	******
#DIV/0!	1	80	******	******
#DIV/0!	1	65	******	******
#DIV/0!	男性	70	99	
#DIV/0!	男性	79	93	
#DIV/0!	男性	60	70	
#DIV/0!	男性	60	97	4.8
#DIV/0!	1	73	******	******
#DIV/0!	1	61	******	******
26.0	男性	70		5.7
#DIV/0!	1	73	******	******
#DIV/0!	男性	72		
#DIV/0!	1	63	******	******
#DIV/0!	1	59	******	******
#DIV/0!	1	63	******	******
#DIV/0!	1	74	******	******
#DIV/0!	1	57	******	******
#DIV/0!	1	69	******	******
#DIV/0!	1	47	******	******
#DIV/0!	1	61	******	******
#DIV/0!	1	42	******	******
31.6	男性	78	137	
#DIV/0!	男性	77		
19.8	男性	79	88	5.5
21.9	1	82	90	5.4
#DIV/0!	1	81	******	******
#DIV/0!	1	79	******	******
24.0	男性	53	96	5.5
#DIV/0!	男性	76		5.5
21.8	男性	88		
29.0	男性	77	137	5.1
21.2	男性	78	******	******
#DIV/0!	1	60	98	5.2

19.3	1	84	******	******
20.8	男性	79	88	5.7
#DIV/0!	1	70	******	******
21.2	男性	79	******	******
#DIV/0!	1	65	******	******
#DIV/0!	1	84	******	******
21.5	1	86	******	******
22.4	1	86	******	******
20.0	1	78	90	5.2
#DIV/0!	1	70	103	5.7
24.2	1	75	122	5.7
22.0	男性	81	99	5.6
22.2	1	70	******	5.7
#DIV/0!	1	79	98	5.3
25.4	1	84	******	******
#DIV/0!	1	86	175	61
22.0	1	73	105	5.4
22.9	1	84	98	5.5
24.7	1	79	******	******
22.9	1	72	74	******
28.7	1	71	102	5.9
#DIV/0!	1	79	121	******
26.1	男性	73	106	
21.8	男性	83	107	******
#DIV/0!	1	77	128	5
#DIV/0!	男性	78	82	5.4
22.9	1	80	83	4.7
#DIV/0!	1	73	******	******
14.3	1	81	******	******
21.1	1	76	98	5.1
27.8	男性	63	******	******
25.3	1	80	101	******
17.7	男性	77	133	
21.5	1	82	123	5.8
#DIV/0!	男性	85	86	
26.6	男性	76	******	******
26.6	男性	61		5
#DIV/0!	男性	71	115	
#DIV/0!	1	64	106	5.8
#DIV/0!	男性	80	97	5.4
30.5	男性	76	124	5.5
24.2	男性	80	95	

#DIV/0!	1	77	109	5.9
#DIV/0!	1	73	103	4.9
#DIV/0!	男性	65	148	6
20.7	男性	85		4.4
17.5	男性	73	144	5.4
19.4	男性	76	112	5.5
21.8	1	79	130	5.5
#DIV/0!	1	71	108	5.8
#DIV/0!	1	79	141	5.5
#DIV/0!	1	60	119	5.5
#DIV/0!	1	71	******	******
#DIV/0!	1	76	109	5.4
28.3	男性	61	110	5.1
25.6	男性	77	100	5.4
#DIV/0!	1	54	******	5.1
#DIV/0!	男性	78	102	6
#DIV/0!	1	89	******	******
20.7	1	81	******	******
24.0	男性	67	105	6.1
#DIV/0!	男性	52		
#DIV/0!	男性	80	89	
#DIV/0!	男性	68	96	
24.7	1	67	******	******
#DIV/0!	1	84	******	******
28.9	1	59	******	******
24.8	男性	59	126	5.6
#DIV/0!	男性	76	97	5
#DIV/0!	1	60	95	4.9
#DIV/0!	男性	80	99	5.2
24.6	男性	76		
#DIV/0!	男性	64	92	5.4
#DIV/0!	1	56	129	5.5
21.0	男性	79	129	5.7
#DIV/0!	1	77	113	5.7
#DIV/0!	男性	61	103	5.6
#DIV/0!	1	76	89	******
26.2	男性	73	114	5.9
#DIV/0!	1	70	118	5.6
#DIV/0!	1	88	******	******
#DIV/0!	1	81	114	5.4
#DIV/0!	男性	60	131	6

#DIV/0!	1	69	136	4.9
#DIV/0!	男性	65	108	5.6
24.2	男性	77	108	5.8
26.7	1	71	132	5.5
#DIV/0!	1	78	96	5.1
#DIV/0!	1	76	******	******
28.4	男性	83	120	5.2
25.0	1	63	90	5.1
#DIV/0!	男性	60	78	******
30.5	1	76	110	6
#DIV/0!	1	68	98	******
20.7	1	73	99	5.7
#DIV/0!	1	84	159	6.1
#DIV/0!	1	78	97	******
#DIV/0!	1	85	118	5.9
25.5	1	78	100	5.4
24.6	1	84	118	6.1
#DIV/0!	1	76	109	5.5
30.5	男性	65	121	5.5
#DIV/0!	1	68	120	5
#DIV/0!	男性	62	96	5
12.9	男性	80		
34.9	1	73	109	5.6
#DIV/0!	1	77	134	6
#DIV/0!	1	72	******	******
23.4	1	81	******	******
20.9	1	71	129	6.4
#DIV/0!	1	61	******	******
#DIV/0!	1	69	92	5.9
#DIV/0!	1	72	******	******
#DIV/0!	1	65	109	5.9
#DIV/0!	1	68	******	******
#DIV/0!	1	63	******	******
#DIV/0!	1	80	166	6.3
24.8	男性	63	125	5.4
#DIV/0!	1	65	95	5.5
33.3	男性	53	117	5.2
23.1	1	76	141	5.4
24.8	男性	71	******	5.6
#DIV/0!	1	78	******	******
#DIV/0!	1	67	******	******
#DIV/0!	男性	75	116	5.7

#DIV/0!	1	61	******	******
#DIV/0!	1	74	******	******
23.6	男性	57	132	4.9
22.8	男性	75		5.5
#DIV/0!	男性	73	83	5.4
#DIV/0!	男性	66	80	5.2
#DIV/0!	男性	63	107	5.2
23.7	男性	77	82	
#DIV/0!	1	72	******	******
#DIV/0!	1	86	******	******
#DIV/0!	男性	56		
#DIV/0!	1	78	******	******
#DIV/0!	1	57	98	5.4
#DIV/0!	1	83	******	******
25.8	1	67	******	******
#DIV/0!	男性	51		
#DIV/0!	男性	43	99	4.9
#DIV/0!	男性	77	109	
#DIV/0!	1	54	******	******
#DIV/0!	男性	85	80	4.8
25.0	1	82	107	5.7
22.3	男性	70	93	6.2
#DIV/0!	1	80	******	******
24.7	1	79	189	5.6
#DIV/0!	男性	66	148	5.9
31.0	男性	70	137	6
#DIV/0!	1	72	******	******
#DIV/0!	1	75	******	******
#DIV/0!	1	43	111	5.1
#DIV/0!	1	76	******	******
#DIV/0!	1	50	******	******
21.4	男性	60	94	5.4
#DIV/0!	1	82	******	******
23.7	男性	46	111	5.1
#DIV/0!	1	74	******	******
#DIV/0!	1	67	160	******
#DIV/0!	男性	52		
29.1	1	38	133	5.7
18.6	男性	88	95	5.5
30.9	1	63	121	5.3
#DIV/0!	1	52	******	******
25.4	1	60	******	******

#DIV/0!	1	38	106	5
#DIV/0!	男性	29	******	5.5
#DIV/0!	男性	54	121	6
#DIV/0!	男性	55	164	5.4
22.3	男性	69	170	5.6
19.4	男性	88	101	5.5
#DIV/0!	男性	62	151	5.8
#DIV/0!	男性	56	117	4.9
#DIV/0!	男性	76	102	5.1
#DIV/0!	1	67	******	******
#DIV/0!	男性	48		
18.8	男性	75	87	
24.8	男性	78		
23.3	1	70	******	******
#DIV/0!	1	55	******	******
23.0	1	82	112	******
#DIV/0!	男性	68		
21.3	1	80	******	******
#DIV/0!	1	54	******	******
22.8	1	63	196	5.1
24.2	1	40	104	4.9
#DIV/0!	男性	41	98	5.7
#DIV/0!	1	83	******	5.6
#DIV/0!	男性	76		
#DIV/0!	1	74	93	5.1
25.2	1	81	110	5.9
#DIV/0!	1	89	******	******
30.6	1	84	******	******
#DIV/0!	1	79	******	******
20.7	男性	54	107	
#DIV/0!	1	67	******	******
24.7	男性	84	******	******
21.0	男性	58	******	******
#DIV/0!	1	63	******	******
23.4	男性	38	92	4.9
#DIV/0!	1	85	131	4.9
#DIV/0!	1	68	100	5.3
22.8	男性	86	107	
#DIV/0!	1	65	94	5.2
#DIV/0!	1	82	96	5.4
#DIV/0!	1	54	100	4.9

18.1	男性	85	98	4.6
#DIV/0!	1	77	******	******
25.5	1	76	102	******
22.6	1	72	130	5.5
#DIV/0!	1	69	123	5
25.2	1	62	******	******
#DIV/0!	男性	68	******	******
#DIV/0!	1	82	******	******
#DIV/0!	1	69	******	******
#DIV/0!	男性	78	******	******
#DIV/0!	1	85	110	5.2
#DIV/0!	1	60	88	5.2
#DIV/0!	男性	72		
#DIV/0!	1	68	******	******
#DIV/0!	1	68	97	5.1
#DIV/0!	男性	68	******	******
#DIV/0!	男性	90	******	******
#DIV/0!	1	70	151	6
#DIV/0!	1	83	******	******
22.5	1	68	102	5
#DIV/0!	男性	55	******	******
#DIV/0!	1	69	104	5.4
#DIV/0!	男性	68	140	5.3
#DIV/0!	1	69	******	5.3
#DIV/0!	1	55	******	******
#DIV/0!	1	80	104	5.2
#DIV/0!	男性	61		5.2
#DIV/0!	男性	26	102	4.9
24.2	男性	75	******	******
#DIV/0!	男性	64	******	******
28.7	男性	40	98	5.4
#DIV/0!	男性	69	111	******
26.5	男性	71	98	5.1
#DIV/0!	男性	71	125	4.9
17.6	1	84	******	5
#DIV/0!	1	60	94	5.2
21.2	1	64	115	5.2
#DIV/0!	男性	59	120	5.5
#DIV/0!	1	49	121	4.8
#DIV/0!	男性	60	93	5.4
35.8	1	75	139	5.6
#DIV/0!	1	84	116	5

#DIV/0!	1	61	99	5.1
30.7	1	64	103	5.2
#DIV/0!	1	44	******	******
26.5	男性	72	115	4.9
#DIV/0!	1	70	108	5.4
19.5	1	77	110	******
#DIV/0!	1	84	******	******
#DIV/0!	1	76	******	******
#DIV/0!	1	78	108	5.3
32.7	1	58	125	5.9
19.8	1	66	121	5.6
20.4	1	85	******	******
24.1	男性	79		
#DIV/0!	1	79	******	5.4
#DIV/0!	男性	82		
#DIV/0!	男性	80	116	******
#DIV/0!	男性	72		
#DIV/0!	男性	65	110	5.2
#DIV/0!	1	79	107	5.2
#DIV/0!	男性	58	89	4.9
16.0	男性	87	97	5.3
#DIV/0!	1	76	105	******
22.6	1	84	90	******
19.7	男性	82	******	******
#DIV/0!	男性	55	96	4.6
#DIV/0!	男性	76	102	5.4
19.8	1	72	******	******
#DIV/0!	1	43	97	5.3
#DIV/0!	男性	75	******	******
28.5	男性	75	127	5.4
#DIV/0!	1	51	******	******
#DIV/0!	1	73	******	5.5
17.8	男性	62	102	5.4
11.9	1	60	******	******
22.0	男性	78	96	5.2
23.7	男性	63	83	5.4
#DIV/0!	1	82	******	******
27.2	1	74	106	5.5
24.1	1	83	95	5.1
17.6	男性	86	******	4.7
18.7	男性	80	129	5

22.3	1	82	******	******
#DIV/0!	男性	69	137	
21.1	1	83	96	******
23.5	1	68	116	5.5
24.6	男性	71		
#DIV/0!	男性	73	92	
#DIV/0!	1	76	134	5.6
24.1	1	65	119	4.9
#DIV/0!	1	77	******	******
#DIV/0!	男性	68	115	6
18.6	1	80	******	******
19.2	男性	72	96	5.1
#DIV/0!	男性	87	95	
#DIV/0!	1	70	108	******
#DIV/0!	1	64	107	5.4
19.2	男性	86	121	5.4
#DIV/0!	男性	69		
#DIV/0!	男性	52	******	******
#DIV/0!	男性	83	******	******
#DIV/0!	男性	56	102	5.4
#DIV/0!	1	61	102	5.1
#DIV/0!	1	69	118	6.1
#DIV/0!	1	60	105	5.2
#DIV/0!	男性	71		
#DIV/0!	1	45	******	******
22.5	1	62	******	4.6
#DIV/0!	男性	64	******	******
#DIV/0!	1	75	126	******
20.5	1	75	131	******
#DIV/0!	男性	58	111	5.2
#DIV/0!	1	56	******	******
23.4	男性	76		
#DIV/0!	男性	67	117	5.5
#DIV/0!	男性	54	108	5.4
#DIV/0!	男性	78	116	6
22.1	1	83	102	4.9
#DIV/0!	1	51	106	5.6
16.1	男性	52	92	
#DIV/0!	1	62	157	4.6
#DIV/0!	男性	65	84	******
#DIV/0!	1	52	109	5.3

#DIV/0!	1	61	92	5
31.6	男性	59	123	6.2
17.8	1	79	101	5.2
20.7	男性	71	92	5.1
#DIV/0!	男性	56	92	5.1
#DIV/0!	1	58	******	******
#DIV/0!	男性	74		5.6
18.5	男性	82	145	5.2
24.6	男性	65	185	5.8
#DIV/0!	1	74	******	******
23.5	1	73	128	5.7
24.2	1	68	132	4.9
20.7	1	67	119	6.3
#DIV/0!	男性	73	92	5.3
22.0	1	72	89	5.3
#DIV/0!	1	69	100	******
#DIV/0!	1	59	******	******
21.4	1	66	97	5.5
18.4	男性	75	101	5.3
#DIV/0!	1	70	******	5.1
#DIV/0!	1	73	99	5.4
21.9	1	63	137	4.9
#DIV/0!	1	76	95	4.8
#DIV/0!	1	66	89	******
#DIV/0!	1	75	******	******
#DIV/0!	1	65	89	6
#DIV/0!	男性	70		
#DIV/0!	1	74	******	******
#DIV/0!	1	84	87	4.8
21.3	1	57	96	******
24.0	男性	58	102	5.1
#DIV/0!	1	64	87	4.8
18.4	1	77	92	5.7
#DIV/0!	1	73	116	5.5
#DIV/0!	1	68	******	5.7
21.8	1	67	******	******
#DIV/0!	男性	84	143	5.2
#DIV/0!	1	87	******	******
#DIV/0!	1	62	******	******
#DIV/0!	男性	64	******	******
#DIV/0!	1	38	******	******
#DIV/0!	男性	65	103	5.4

#DIV/0!	1	76	******	******
#DIV/0!	男性	68	93	
21.4	1	88	******	******
#DIV/0!	男性	59	105	
#DIV/0!	1	79	119	5.1
25.1	男性	75	126	******
#DIV/0!	1	77	93	5.7
#DIV/0!	1	48	92	5.4
#DIV/0!	1	63	******	******
#DIV/0!	1	68	134	5.8
#DIV/0!	1	68	******	******
#DIV/0!	男性	61	120	4.8
20.2	1	85	108	5.1
#DIV/0!	1	86	******	******
#DIV/0!	1	85	******	******
#DIV/0!	1	70	******	******
#DIV/0!	男性	62	142	4.4
#DIV/0!	1	65	153	5.9
16.7	男性	82	111	5.5
23.8	男性	79	102	5.3
#DIV/0!	男性	69	******	5.7
25.0	1	82	******	******
#DIV/0!	1	89	******	******
#DIV/0!	男性	58	******	4.3
#DIV/0!	1	70	******	******
#DIV/0!	1	73	******	5.1
#DIV/0!	1	81	******	******
24.5	1	62	129	5.8
16.7	1	61	******	******
#DIV/0!	1	48	96	5.2
#DIV/0!	1	67	******	******
#DIV/0!	1	78	95	5.4
#DIV/0!	1	34	91	4.6
#DIV/0!	1	78	******	******
#DIV/0!	1	70	******	******
19.1	男性	64	******	******
#DIV/0!	男性	66	94	5.9
21.7	男性	62	125	
19.0	男性	80	132	5
#DIV/0!	1	68	******	******
#DIV/0!	1	62	******	******
#DIV/0!	1	51	120	5.2

#DIV/0!	男性	55	108	4.9
22.4	男性	72	101	******
#DIV/0!	1	90	******	******
17.3	1	83	******	******
#DIV/0!	男性	68	102	
#DIV/0!	1	64	******	******
#DIV/0!	男性	55	102	5.1
#DIV/0!	男性	77	102	4.6
#DIV/0!	1	76	******	******
19.7	男性	35	83	4.6
#DIV/0!	男性	37	100	******
#DIV/0!	1	57	179	5
#DIV/0!	男性	69	113	5.1
#DIV/0!	1	57	116	5.3
#DIV/0!	1	53	******	******
#DIV/0!	1	58	******	******
#DIV/0!	1	76	******	******
#DIV/0!	1	58	119	5.7
#DIV/0!	1	77	******	******
17.3	男性	59	93	******
23.0	1	61	92	4.7
20.3	男性	64	253	
#DIV/0!	男性	72		5.2
20.4	男性	58	123	4.7
#DIV/0!	男性	82	108	4.7
17.1	男性	72	127	5.6
20.7	1	51	113	5.3
#DIV/0!	1	83	94	5.1
19.5	1	60	******	******
23.7	1	71	89	4.9
#DIV/0!	1	58	99	******
#DIV/0!	男性	78	146	5.3
33.3	1	60	162	5.4
#DIV/0!	1	61	100	4.9
21.8	男性	55	173	5.4
24.8	男性	42	111	5
17.6	男性	58	85	4.4
#DIV/0!	1	70	******	******
#DIV/0!	1	59	******	******
#DIV/0!	1	48	107	5.8
#DIV/0!	1	75	******	******
26.1	男性	47	******	5.6

#DIV/0!	男性	56	108	5.7
#DIV/0!	男性	48	110	******
20.3	男性	72	137	5.5
#DIV/0!	1	68	89	******
#DIV/0!	1	22	******	4.9
#DIV/0!	1	71	******	******
#DIV/0!	1	67	******	******
#DIV/0!	1	29	******	******
20.5	1	62	******	******
#DIV/0!	男性	62	150	5.4
#DIV/0!	1	48	******	******
16.0	1	72	110	5.9
#DIV/0!	1	62	******	******
#DIV/0!	男性	41	94	5.1
#DIV/0!	男性	79	103	
#DIV/0!	1	59	94	4.3
#DIV/0!	1	38	******	******
#DIV/0!	男性	71	130	5.6
#DIV/0!	1	35	93	4.5
#DIV/0!	1	90	******	******
#DIV/0!	男性	81	126	5.7
#DIV/0!	1	48	******	******
#DIV/0!	男性	56	169	******
#DIV/0!	1	42	******	******
#DIV/0!	男性	51	108	******
21.8	男性	59	87	5.2
#DIV/0!	男性	34	80	4.6
#DIV/0!	男性	26	102	5.1
21.5	男性	79	134	5.9
21.1	男性	31	89	******
#DIV/0!	男性	55	160	8.7
20.1	男性	62	108	5.3
#DIV/0!	男性	58		4
#DIV/0!	男性	72	125	4.9
#DIV/0!	男性	89	110	
19.8	男性	26	101	5
#DIV/0!	男性	77	110	
24.6	男性	60	159	
#DIV/0!	男性	67	110	
#DIV/0!	男性	84	105	
#DIV/0!	1	69	******	******

#DIV/0!	1	55	******	5.1
#DIV/0!	1	67	101	5.2
22.5	男性	67	115	3.8
#DIV/0!	1	67	******	******
#DIV/0!	男性	62	125	
#DIV/0!	1	60	106	******
21.1	男性	64	148	6.2
18.1	1	68	******	******
#DIV/0!	1	70	143	5.5
#DIV/0!	1	42	89	5
#DIV/0!	男性	60	82	
#DIV/0!	男性	56	95	4.9
#DIV/0!	1	53	******	******
24.3	男性	49	122	5.3
20.9	男性	71	100	5
#DIV/0!	男性	56	******	******
#DIV/0!	1	85	******	******
#DIV/0!	1	65	155	******
#DIV/0!	1	50	******	5.2
#DIV/0!	1	64	******	5.6
#DIV/0!	男性	77	108	4.5
20.2	男性	79	146	5.3
#DIV/0!	1	64	84	5
15.4	1	66	******	******
18.5	男性	69	110	5
19.2	1	54	******	******
#DIV/0!	1	61	98	4.8
17.0	1	66	120	5.2
#DIV/0!	男性	67	93	4.7
18.9	1	75	******	******
#DIV/0!	男性	72	67	
#DIV/0!	男性	47	******	******
#DIV/0!	男性	74		
#DIV/0!	男性	64	86	4.4
#DIV/0!	男性	40	******	******
#DIV/0!	男性	65	105	5
#DIV/0!	男性	44	******	******
#DIV/0!	男性	68	******	******
#DIV/0!	男性	68		
#DIV/0!	男性	64	62	3.8
#DIV/0!	男性	71		

#DIV/0!	男性	61	******	******
#DIV/0!	男性	79		
#DIV/0!	男性	41	81	4.9
26.7	1	73	96	******
#DIV/0!	1	74	148	5.4
#DIV/0!	1	70	******	******
#DIV/0!	1	76	103	******
#DIV/0!	男性	78	114	5.5
#DIV/0!	1	74	******	******
#DIV/0!	1	67	******	******
#DIV/0!	1	62	******	******
24.5	男性	77	114	5.1
25.1	男性	84		
21.6	1	71	******	******
#DIV/0!	男性	83	98	
#DIV/0!	1	83	95	5
27.1	1	86	******	******
27.0	1	70	105	******
22.8	1	74	******	******
21.6	1	83	104	5.6
22.0	1	78	******	5.5
31.6	1	85	******	******
#DIV/0!	男性	79		
#DIV/0!	1	78	******	******
22.8	1	84	96	******
22.0	1	83	73	4.9
20.6	男性	73	114	4.8
#DIV/0!	1	83	******	******
19.3	男性	75	107	5.3
20.8	1	90	98	******
#DIV/0!	1	83	******	******
24.6	男性	78	108	5.4
25.5	男性	86		
28.0	男性	78	89	5
#DIV/0!	男性	80	92	
#DIV/0!	男性	79	77	5.2
#DIV/0!	男性	87	112	
24.7	男性	77	141	6.1
24.6	男性	82	106	
25.5	1	83	87	4.7
26.7	1	78	******	******

#DIV/0!	1	74	******	******
#DIV/0!	男性	78	117	******
20.8	1	84	******	******
18.6	男性	80	88	5
#DIV/0!	1	92	******	******
23.5	男性	74		
24.2	男性	67	135	5.2
20.8	男性	65	165	4.8
#DIV/0!	1	81	******	******
#DIV/0!	男性	83	108	******
24.2	男性	78		5.7
30.0	1	56	108	5.4
#DIV/0!	1	76	******	******
#DIV/0!	男性	88		
29.2	男性	69	******	5.3
#DIV/0!	男性	86	92	5.3
27.0	男性	66		5.7
23.9	1	60	112	6.3
#DIV/0!	男性	72	128	4.8
19.6	男性	69	117	6
#DIV/0!	男性	77	92	4.9
#DIV/0!	1	72	112	5.6
17.5	男性	75	102	5.4
21.1	1	85	107	5.9
18.6	1	77	103	4.7
13.3	1	81	120	5.7
19.7	男性	70	178	5.6
#DIV/0!	1	77	104	5.8
35.1	1	76	109	5.6
23.1	1	78	107	******
#DIV/0!	1	84	130	5.4
22.5	1	60	113	******
25.8	男性	58	110	5.5
#DIV/0!	男性	72	******	5.2
25.0	1	64	******	******
#DIV/0!	1	74	107	5.8
18.6	1	66	123	5.8
21.7	男性	79	153	5.5
#DIV/0!	1	84	88	5.7
24.2	1	76	108	******
#DIV/0!	男性	66	100	5.6

19.3	1	85	113	5.6
23.2	1	74	139	5.8
#DIV/0!	男性	51	97	
#DIV/0!	1	74	******	******
#DIV/0!	男性	72	105	
18.7	男性	83	108	
21.0	1	62	123	5.7
26.1	1	81	******	******
#DIV/0!	1	78	134	6.1
#DIV/0!	1	67	******	******
21.0	男性	76	99	5.2
#DIV/0!	1	72	******	******
#DIV/0!	1	66	******	******
#DIV/0!	1	79	******	******
#DIV/0!	1	86	******	******
#DIV/0!	1	55	******	******
#DIV/0!	1	49	121	5.2
#DIV/0!	1	68	******	******
#DIV/0!	1	66	******	******
#DIV/0!	1	79	110	5.3
#DIV/0!	1	80	******	******
21.5	1	85	******	******
22.0	男性	90	118	
18.4	男性	66	98	******
22.7	男性	77	105	4.8
20.6	男性	73	116	5.1
#DIV/0!	1	71	******	******
24.2	1	63	******	******
16.0	男性	86	99	6
21.2	男性	66	109	4.9
21.2	男性	66	109	4.9
#DIV/0!	男性	96	119	5
#DIV/0!	1	79	******	******
#DIV/0!	男性	81	88	4.9
25.6	男性	61	130	5.6
#DIV/0!	1	66	******	******
#DIV/0!	1	61	******	******
#DIV/0!	1	66	101	5.2
#DIV/0!	1	80	******	******
#DIV/0!	1	86	******	******
#DIV/0!	男性	72	151	4.4
#DIV/0!	1	66	******	******

17.8					1	79	******	******
#DIV/0!					1	44	166	6
#DIV/0!					1	71	******	******
#DIV/0!					1	76	******	******
#DIV/0!					男性	81	100	5.4
#DIV/0!					1	76	******	******
20.1					男性	74	133	5.7
25.9					男性	51	91	4.6
#DIV/0!					男性	40	114	5
22.8					男性	80	142	5.5
#DIV/0!					男性	85	126	5.5
22.9					男性	64	134	5.7
#DIV/0!					男性	71	104	
#DIV/0!					男性	91	83	
24.8					男性	72	114	5.8
#DIV/0!					1	91	******	******
#DIV/0!					1	60	******	******
17.9					1	82	******	******
21.0					1	61	******	******
#DIV/0!					1	78	******	******
#DIV/0!					男性	79	98	5.7
#DIV/0!					1	62	******	******
#DIV/0!					1	65	******	******
25.2					1	71	97	5.5
22.6					男性	75	99	
#DIV/0!					男性	81	141	6.2
17.3	30		0	0	男性	82	193	6.8
#DIV/0!					男性	66	107	5.4
#DIV/0!	0	0			1	75	129	6.6
21.0	24	0	0	0	男性	72	160	7.9
#DIV/0!					男性	71	******	******
20.1	14	1			男性	77	150	6.6
#DIV/0!					男性	67	******	******
24.4	20		2	0	男性	53	213	7.9
23.9					男性	53	90	5.5
21.5	20		1	1	男性	78	193	6.4
24.6	7	0			1	36	127	6.2
#DIV/0!					1	69	125	6.6
24.9	13	1			男性	50	144	6
#DIV/0!					1	55	151	6.8
#DIV/0!					1	53	148	7.1

#DIV/0!					男性	48	126	6.3
#DIV/0!					男性	71	132	******
31.9	0		0	0	男性	40	120	6.3
#DIV/0!					1	61		
29.5					男性	68	112	6.3
28.5	30		1	0	男性	60	66	5.2
#DIV/0!					1	84	113	6.9
#DIV/0!					男性	71	132	6.8
#DIV/0!					男性	64	152	6.4
24.7	2	0			1	75	120	5.9
22.6					男性	79	173	5.9
#DIV/0!					男性	61	114	5.7
#DIV/0!					男性	74		
24.6					男性	73		
#DIV/0!					男性	71	139	
#DIV/0!					男性	63	******	******
#DIV/0!					男性	30	******	******
#DIV/0!					男性	63	******	******
#DIV/0!					男性	87	106	6
30.5			0	1	男性	44	106	5
#DIV/0!					1	77	128	
16.6					男性	78	107	6.1
#DIV/0!					男性	63		
19.6					1	66	******	******
#DIV/0!					1	62	106	******
17.6					1	74	123	5
#DIV/0!					男性	81		
#DIV/0!					1	64	106	
23.0					男性	69	******	******
#DIV/0!					男性	82	******	******
#DIV/0!					男性	83		
#DIV/0!					1	65	125	5
25.2					男性	53		
#DIV/0!					男性	81	******	******
#DIV/0!					1	44		
#DIV/0!					男性	79		
#DIV/0!					1	75	90	5.2
#DIV/0!					男性	57		
#DIV/0!					1	79		
#DIV/0!					1	75		
#DIV/0!					男性	68	******	******

#DIV/0!			男性	71	110	5.5
#DIV/0!			1	64	87	5.2
#DIV/0!			1	51	108	5.3
23.4			1	52	100	4.7
#DIV/0!			1	76		5.7
#DIV/0!			1	63		
21.0			男性	76		
23.3			男性	68		
#DIV/0!			男性	64	******	******
#DIV/0!			1	69		
#DIV/0!			男性	55	******	******
#DIV/0!			男性	65	******	******
23.7	0	0	男性	67	103	5.2
#DIV/0!			男性	70		
#DIV/0!			男性	59		
#DIV/0!			男性	61		
#DIV/0!			1	76		
#DIV/0!			1	54		
#DIV/0!			男性	66	******	******
#DIV/0!			男性	65	93	4.6
#DIV/0!			男性	30		
17.3	1	0	男性	69		
#DIV/0!			1	68		
24.9			男性	51	******	******
#DIV/0!			1	63	104	
23.8			1	44	83	4.7
#DIV/0!			1	56	89	4.8
#DIV/0!			男性	38	94	
#DIV/0!			1	55	100	4.8
#DIV/0!			1	62	98	5.3
#DIV/0!			男性	43		5.4
#DIV/0!			1	66		
#DIV/0!			1	44		
#DIV/0!			1	31		
#DIV/0!			1	61		
#DIV/0!			1	53	90	5
#DIV/0!			1	77	******	******
#DIV/0!			1	42	******	******
#DIV/0!			男性	68		
#DIV/0!			男性	62	******	******
#DIV/0!			男性	61	******	******

27.0				男性	43		
#DIV/0!				男性	69		
25.8				男性	59	127	5
22.9				1	64	114	
19.1				男性	70	123	5
27.9				1	57	91	5.2
#DIV/0!				1	59	92	5.3
#DIV/0!				1	69		5.1
19.9				男性	67	128	5.4
#DIV/0!				男性	40	94	5.2
#DIV/0!				男性	77		
20.7				1	70	111	5.3
23.0				男性	76	******	******
22.7				男性	70	******	******
#DIV/0!				1	59	******	******
#DIV/0!				男性	61	100	5
#DIV/0!				男性	62		5.3
#DIV/0!				男性	70		6.2
#DIV/0!		1	1	男性	68		
18.0				男性	62		
#DIV/0!				男性	77	******	******
23.9				1	84	111	5.3
#DIV/0!				男性	76	107	5.2
20.2				男性	85		
26.8				1	64		
#DIV/0!				1	67		
29.8				1	67	87	5.2
22.6				1	61	******	******
24.3				男性	73	81	5.7
24.0				男性	77	******	******
21.2	0			男性	78	128	6.5
#DIV/0!				男性	68	112	5.5
#DIV/0!				男性	66	116	4.3
16.6				男性	84	125	5.3
#DIV/0!				男性	61	101	4.8
25.6				1	60	122	6.1
23.9		0	0	男性	68		
#DIV/0!				男性	65	107	
#DIV/0!				男性	63	******	******
#DIV/0!				男性	46	111	5
23.9				男性	68	******	******

20.8					男性	73		
22.8					男性	48	107	5.6
#DIV/0!					男性	71		
24.8					1	74	122	5.2
21.6					男性	83	152	5.3
26.4					1	65	107	******
#DIV/0!					男性	65	77	4.4
#DIV/0!					1	62	109	5.1
BMI	duration	retino	smoking	alcho	sex	age	BS	HbA1c

prostatitis		
0	1	
0	2	
0	3	
0	4	
0	5	
0	6	
0	7	
0	8	
0	9	
0	10	
0	11	
0	12	
0	13	
0	14	
0	15	
0	16	
0	17	
0	18	
0	19	
0	20	
0	21	
0	22	
0	23	
0	24	
0	25	
0	26	
0	27	
0	28	
0	29	
0	30	
0	31	
0	32	
0	33	
0	34	
0	35	
0	36	
1	37	
1	38	
1	39	
1	40	
1	41	

1	42
1	43
1	44
1	45
1	46
0	47
0	48
0	49
0	50
0	51
0	52
0	53
0	54
0	55
0	56
0	57
0	58
0	59
0	60
0	61
0	62
0	63
0	64
0	65
0	66
0	67
0	68
0	69
0	70
0	71
0	72
0	73
0	74
0	75
0	76
0	77
0	78
0	79
0	80
0	81
0	82
0	83
0	84

0	85
0	86
0	87
0	88
0	89
0	90
0	91
0	92
0	93
0	94
0	95
0	96
0	97
0	98
0	99
0	100
0	101
0	102
0	103
0	104
0	105
0	106
0	107
0	108
0	109
0	110
0	111
0	112
0	113
0	114
0	115
0	116
0	117
0	118
0	119
0	120
0	121
0	122
0	123
0	124
0	125
0	126
0	127

0	128
0	129
0	130
0	131
0	132
0	133
0	134
0	135
0	136
0	137
0	138
0	139
0	140
0	141
0	142
0	143
0	144
0	145
0	146
0	147
0	148
0	149
0	150
0	151
0	152
0	153
0	154
0	155
0	156
0	157
0	158
0	159
0	160
0	161
0	162
0	163
0	164
0	165
0	166
0	167
0	168
0	169

0	170
0	171
0	172
0	173
0	174
0	175
0	176
0	177
0	178
0	179
0	180
0	181
0	182
0	183
0	184
0	185
0	186
0	187
0	188
0	189
0	190
0	191
0	192
0	193
0	194
0	195
0	196
0	197
0	198
0	199
0	200
0	201
0	202
0	203
0	204
0	205
0	206
0	207
0	208
0	209
0	210
0	211

0	212
0	213
0	214
0	215
0	216
0	217
0	218
0	219
0	220
0	221
0	222
0	223
0	224
0	225
0	226
0	227
0	228
0	229
0	230
0	231
0	232
0	233
0	234
0	235
0	236
0	237
0	238
0	239
0	240
0	241
0	242
0	243
0	244
0	245
0	246
0	247
0	248
0	249
0	250
0	251
0	252
0	253

0	254
0	255
0	256
0	257
0	258
0	259
0	260
0	261
0	262
0	263
0	264
0	265
0	266
0	267
0	268
0	269
0	270
0	271
0	272
0	273
0	274
0	275
0	276
0	277
0	278
0	279
0	280
0	281
0	282
0	283
0	284
0	285
0	286
0	287
0	288
0	289
0	290
0	291
0	292
0	293
0	294
0	295

0	296
0	297
0	298
0	299
0	300
0	301
0	302
0	303
0	304
0	305
0	306
0	307
0	308
0	309
0	310
0	311
0	312
0	313
0	314
0	315
0	316
0	317
0	318
0	319
0	320
0	321
0	322
0	323
0	324
0	325
0	326
0	327
0	328
0	329
0	330
0	331
0	332
0	333
0	334
0	335
0	336
0	337

0	338
0	339
0	340
0	341
0	342
0	343
0	344
0	345
0	346
0	347
0	348
0	349
0	350
0	351
0	352
0	353
0	354
0	355
0	356
0	357
0	358
0	359
0	360
0	361
0	362
0	363
0	364
0	365
0	366
0	367
0	368
0	369
0	370
0	371
0	372
0	373
0	374
0	375
0	376
0	377
0	378
0	379

0	380
0	381
0	382
0	383
0	384
0	385
0	386
0	387
0	388
0	389
0	390
0	391
0	392
0	393
0	394
0	395
0	396
0	397
0	398
0	399
0	400
0	401
0	402
0	403
0	404
0	405
0	406
0	407
0	408
0	409
0	410
0	411
0	412
0	413
0	414
0	415
0	416
0	417
0	418
0	419
0	420
0	421
0	422

0	423
0	424
0	425
0	426
0	427
0	428
0	429
0	430
0	431
0	432
0	433
0	434
0	435
0	436
0	437
0	438
0	439
0	440
0	441
0	442
0	443
0	444
0	445
0	446
0	447
0	448
0	449
0	450
0	451
0	452
0	453
0	454
0	455
0	456
0	457
0	458
0	459
0	460
0	461
0	462
0	463
0	464

0	465
0	466
0	467
0	468
0	469
0	470
0	471
0	472
0	473
0	474
0	475
0	476
0	477
0	478
0	479
0	480
0	481
0	482
0	483
0	484
0	485
0	486
0	487
0	488
0	489
0	490
0	491
0	492
0	493
0	494
0	495
0	496
0	497
0	498
0	499
0	500
0	501
0	502
0	503
0	504
0	505
0	506

0	507
0	508
0	509
0	510
0	511
0	512
0	513
0	514
0	515
0	516
0	517
0	518
0	519
0	520
0	521
0	522
0	523
0	524
0	525
0	526
0	527
0	528
0	529
1	530
0	531
0	532
0	533
0	534
0	535
0	536
0	537
0	538
0	539
0	540
0	541
0	542
0	543
0	544
0	545
0	546
0	547

0	548
0	549
0	550
0	551
0	552
0	553
0	554
0	555
0	556
0	557
0	558
0	559
0	560
0	561
0	562
0	563
0	564
0	565
0	566
0	567
0	568
0	569
0	570
0	571
0	572
0	573
0	574
0	575
0	576
0	577
0	578
0	579
0	580
0	581
0	582
0	583
0	584
0	585
0	586
0	587
0	588
0	589

0	590
0	591
0	592
0	593
0	594
0	595
0	596
0	597
0	598
0	599
0	600
0	601
0	602
0	603
0	604
0	605
0	606
0	607
0	608
0	609
0	610
0	611
0	612
0	613
0	614
0	615
0	616
0	617
0	618
0	619
0	620
0	621
0	622
0	623
0	624
0	625
0	626
0	627
0	628
0	629
0	630
0	631
1	632

0	633
0	634
0	635
0	636
0	637
0	638
0	639
0	640
0	641
0	642
0	643
0	644
0	645
0	646
0	647
0	648
0	649
0	650
0	651
0	652
0	653
0	654
0	655
0	656
0	657
0	658
0	659
0	660
0	661
0	662
0	663
0	664
0	665
0	666
0	667
0	668
0	669
0	670
0	671
0	672
0	673
0	674

0	675
0	676
0	677
0	678
0	679
0	680
0	681
0	682
0	683
0	684
0	685
0	686
0	687
0	688
0	689
0	690
0	691
0	692
0	693
0	694
0	695
0	696
0	697
0	698
0	699
0	700
0	701
0	702
0	703
0	704
0	705
0	706
0	707
0	708
0	709
0	710
0	711
0	712
0	713
0	714
0	715
0	716

0	717
0	718
0	719
0	720
0	721
0	722
0	723
0	724
0	725
0	726
0	727
0	728
0	729
0	730
0	731
0	732
0	733
0	734
0	735
0	736
0	737
0	738
0	739
0	740
0	741
0	742
0	743
0	744
0	745
0	746
0	747
0	748
0	749
0	750
0	751
0	752
0	753
0	754
0	755
0	756
0	757
0	758

0	759
0	760
0	761
0	762
0	763
0	764
0	765
0	766
0	767
0	768
0	769
0	770
0	771
0	772
0	773
0	774
0	775
0	776
0	777
0	778
0	779
0	780
0	781
0	782
0	783
0	784
0	785
0	786
0	787
0	788
0	789
0	790
0	791
0	792
0	793
1	794
1	795
0	796
0	797
0	798
0	799
0	800

0	801
0	802
0	803
0	804
0	805
0	806
0	807
0	808
0	809
0	810
0	811
0	812
0	813
0	814
0	815
0	816
0	817
0	818
0	819
0	820
0	821
0	822
0	823
0	824
0	825
0	826
0	827
0	828
0	829
0	830
0	831
0	832
0	833
0	834
0	835
0	836
0	837
0	838
0	839
0	840
0	841

0	842
0	843
0	844
0	845
0	846
0	847
0	848
0	849
0	850
0	851
0	852
0	853
0	854
0	855
0	856
0	857
0	858
0	859
0	860
0	861
0	862
0	863
0	864
0	865
0	866
0	867
0	868
0	869
0	870
0	871
0	872
0	873
0	874
0	875
0	876
0	877
0	878
0	879
0	880
0	881
0	882
0	883

0	884
0	885
0	886
0	887
0	888
0	889
0	890
0	891
0	892
0	893
0	894
0	895
0	896
0	897
0	898
0	899
0	900
0	901
0	902
0	903
0	904
0	905
0	906
0	907
0	908
0	909
0	910
0	911
0	912
0	913
0	914
0	915
0	916
0	917
0	918
0	919
0	920
0	921
0	922
0	923
0	924
0	925

0	926
0	927
0	928
0	929
0	930
0	931
0	932
0	933
0	934
0	935
0	936
0	937
0	938
0	939
0	940
0	941
0	942
0	943
0	944
0	945
0	946
0	947
0	948
0	949
0	950
0	951
0	952
0	953
0	954
0	955
0	956
0	957
0	958
0	959
0	960
0	961
0	962
0	963
0	964
0	965
0	966

0	967
0	968
0	969
0	970
0	971
0	972
0	973
0	974
0	975
0	976
0	977
0	978
0	979
0	980
0	981
0	982
0	983
0	984
0	985
0	986
0	987
0	988
0	989
0	990
0	991
0	992
0	993
0	994
0	995
0	996
0	997
0	998
0	999
0	1000
0	1001
0	1002
0	1003
0	1004
0	1005
0	1006
0	1007

0	1008
0	1009
0	1010
0	1011
0	1012
0	1013
0	1014
0	1015
0	1016
0	1017
0	1018
0	1019
0	1020
0	1021
0	1022
0	1023
0	1024
0	1025
0	1026
0	1027
0	1028
0	1029
0	1030
0	1031
0	1032
0	1033
0	1034
0	1035
0	1036
0	1037
0	1038
0	1039
0	1040
0	1041
0	1042
0	1043
0	1044
0	1045
0	1046
0	1047
0	1048
0	1049

0	1050	
0	1051	
0	1052	
0	1053	
0	1054	
0	1055	
0	1056	
0	1057	
0	1058	
0	1059	
0	1060	
0	1061	
0	1062	
0	1063	
0	1064	
0	1065	
0	1066	
0	1067	
0	1068	
0	1069	
0	1070	
0	1071	
0	1072	
0	1073	
0	1074	
0	1075	************
0	1076	
0	1077	
0	1078	
0	1079	
0	1080	
0	1081	
0	1082	
0	1083	
0	1084	
0	1085	
0	1086	
0	1087	
0	1088	
0	1089	
0	1090	
0	1091	

0	1092
0	1093
0	1094
0	1095
0	1096
0	1097
0	1098
0	1099
0	1100
0	1101
0	1102
0	1103
0	1104
0	1105
0	1106
0	1107
0	1108
0	1109
0	1110
0	1111
0	1112
0	1113
0	1114
0	1115
0	1116
0	1117
0	1118
0	1119
0	1120
0	1121
0	1122
0	1123
0	1124
0	1125
0	1126
0	1127
0	1128
0	1129
0	1130
0	1131
0	1132
0	1133
0	1134

0	1135
0	1136
0	1137
0	1138
0	1139
0	1140
0	1141
0	1142
0	1143
0	1144
0	1145
0	1146
0	1147
0	1148
0	1149
0	1150
0	1151
0	1152
0	1153
0	1154
0	1155
0	1156
0	1157
0	1158
0	1159
0	1160
0	1161
0	1162
0	1163
0	1164
0	1165
0	1166
0	1167
0	1168
0	1169
0	1170
0	1171
0	1172
0	1173
0	1174
0	1175
0	1176

0	1177
0	1178
0	1179
0	1180
0	1181
0	1182
0	1183
0	1184
0	1185
0	1186
0	1187
0	1188
0	1189
0	1190
0	1191
0	1192
0	1193
0	1194
0	1195
0	1196
0	1197
0	1198
0	1199
0	1200
0	1201
0	1202
0	1203
0	1204
0	1205
0	1206
0	1207
0	1208
0	1209
0	1210
0	1211
0	1212
0	1213
0	1214
0	1215
0	1216
0	1217
0	1218

0	1219
0	1220
0	1221
0	1222
0	1223
0	1224
0	1225
0	1226
0	1227
0	1228
0	1229
0	1230
0	1231
0	1232
0	1233
0	1234
0	1235
0	1236
0	1237
0	1238
0	1239
0	1240
0	1241
0	1242
0	1243
0	1244
0	1245
0	1246
0	1247
0	1248
0	1249
0	1250
0	1251
0	1252
0	1253
0	1254
0	1255
0	1256
0	1257
0	1258
0	1259
0	1260

0	1261
0	1262
0	1263
0	1264
0	1265
0	1266
0	1267
0	1268
0	1269
0	1270
0	1271
0	1272
0	1273
0	1274
0	1275
0	1276
0	1277
0	1278
0	1279
0	1280
0	1281
0	1282
0	1283
0	1284
0	1285
0	1286
0	1287
0	1288
0	1289
0	1290
0	1291
0	1292
0	1293
0	1294
0	1295
0	1296
0	1297
0	1298
0	1299
0	1300
0	1301
0	1302

0	1303
0	1304
0	1305
0	1306
0	1307
0	1308
0	1309
0	1310
0	1311
0	1312
0	1313
0	1314
0	1315
0	1316
0	1317
0	1318
0	1319
0	1320
0	1321
0	1322
0	1323
0	1324
0	1325
0	1326
0	1327
0	1328
0	1329
0	1330
0	1331
0	1332
0	1333
0	1334
0	1335
0	1336
0	1337
0	1338
0	1339
0	1340
0	1341
0	1342
0	1343
0	1344

0	1345
0	1346
0	1347
0	1348
0	1349
0	1350
0	1351
0	1352
0	1353
0	1354
0	1355
0	1356
0	1357
0	1358
0	1359
0	1360
0	1361
0	1362
0	1363
0	1364
0	1365
0	1366
0	1367
0	1368
0	1369
0	1370
0	1371
0	1372
0	1373
0	1374
0	1375
0	1376
0	1377
0	1378
0	1379
0	1380
0	1381
0	1382
0	1383
0	1384
0	1385
0	1386

0	1387
0	1388
0	1389
0	1390
0	1391
0	1392
0	1393
0	1394
0	1395
0	1396
0	1397
0	1398
0	1399
0	1400
0	1401
0	1402
0	1403
0	1404
0	1405
0	1406
0	1407
0	1408
0	1409
0	1410
0	1411
0	1412
0	1413
0	1414
0	1415
0	1416
0	1417
0	1418
0	1419
0	1420
0	1421
0	1422
0	1423
0	1424
0	1425
0	1426
0	1427
0	1428

0	1429
0	1430
0	1431
0	1432
0	1433
0	1434
0	1435
0	1436
0	1437
0	1438
0	1439
0	1440
0	1441
0	1442
0	1443
0	1444
0	1445
0	1446
0	1447
0	1448
0	1449
0	1450
0	1451
0	1452
0	1453
0	1454
0	1455
0	1456
0	1457
0	1458
0	1459
0	1460
0	1461
0	1462
0	1463
0	1464
0	1465
0	1466
0	1467
0	1468
0	1469
0	1470

0	1471
0	1472
0	1473
0	1474
0	1475
0	1476
0	1477
0	1478
0	1479
0	1480
0	1481
0	1482
0	1483
0	1484
0	1485
0	1486
0	1487
0	1488
0	1489
0	1490
0	1491
0	1492
0	1493
0	1494
0	1495
0	1496
0	1497
0	1498
0	1499
0	1500
0	1501
0	1502
0	1503
0	1504
0	1505
0	1506
0	1507
0	1508
0	1509
0	1510
0	1511
0	1512

0	1513
0	1514
0	1515
0	1516
0	1517
0	1518
0	1519
0	1520
0	1521
0	1522
0	1523
0	1524
0	1525
0	1526
0	1527
0	1528
0	1529
0	1530
0	1531
0	1532
0	1533
0	1534
0	1535
0	1536
0	1537
0	1538
0	1539
0	1540
0	1541
0	1542
0	1543
0	1544
0	1545
0	1546
0	1547
0	1548
0	1549
0	1550
0	1551
0	1552
0	1553
0	1554

0	1555
0	1556
0	1557
0	1558
0	1559
0	1560
0	1561
0	1562
0	1563
0	1564
0	1565
0	1566
0	1567
0	1568
0	1569
0	1570
0	1571
0	1572
0	1573
0	1574
0	1575
0	1576
0	1577
0	1578
0	1579
0	1580
0	1581
0	1582
0	1583
0	1584
0	1585
0	1586
0	1587
0	1588
0	1589
0	1590
0	1591
0	1592
0	1593
0	1594
0	1595
0	1596

0	1597
0	1598
0	1599
0	1600
0	1601
0	1602
0	1603
0	1604
0	1605
0	1606
0	1607
0	1608
0	1609
0	1610
0	1611
0	1612
0	1613
0	1614
0	1615
0	1616
0	1617
0	1618
0	1619
0	1620
0	1621
0	1622
0	1623
0	1624
0	1625
0	1626
0	1627
0	1628
0	1629
0	1630
0	1631
0	1632
0	1633
0	1634
0	1635
0	1636
0	1637

0	1638
0	1639
0	1640
0	1641
0	1642
0	1643
0	1644
0	1645
0	1646
0	1647
0	1648
0	1649
0	1650
0	1651
0	1652
0	1653
0	1654
0	1655
0	1656
0	1657
0	1658
0	1659
0	1660
0	1661
0	1662
0	1663
1	1664
1	1665
1	1666
1	1667
1	1668
1	1669
1	1670
1	1671
1	1672
1	1673
1	1674
1	1675
1	1676
1	1677
1	1678

1	1679
1	1680
1	1681
0	1682
0	1683
0	1684
0	1685
0	1686
0	1687
0	1688
0	1689
0	1690
0	1691
0	1692
0	1693
0	1694
0	1695
0	1696
0	1697
0	1698
0	1699
0	1700
0	1701
0	1702
0	1703
0	1704
0	1705
0	1706
0	1707
0	1708
0	1709
0	1710
0	1711
0	1712
0	1713
0	1714
0	1715
0	1716
0	1717
0	1718
0	1719
0	1720

0	1721
0	1722
0	1723
0	1724
0	1725
0	1726
0	1727
0	1728
0	1729
0	1730
0	1731
0	1732
0	1733
0	1734
0	1735
0	1736
0	1737
0	1738
0	1739
0	1740
0	1741
0	1742
0	1743
0	1744
0	1745
0	1746
0	1747
0	1748
0	1749
0	1750
0	1751
0	1752
0	1753
0	1754
0	1755
0	1756
0	1757
0	1758
0	1759
0	1760
0	1761
0	1762

0	1763
0	1764
0	1765
0	1766
0	1767
0	1768
0	1769
0	1770
0	1771
0	1772
0	1773
0	1774
0	1775
0	1776
0	1777
0	1778
0	1779
0	1780
0	1781
0	1782
0	1783
0	1784
0	1785
0	1786
0	1787
0	1788
0	1789
0	1790
0	1791
0	1792
0	1793
0	1794
0	1795
0	1796
0	1797
0	1798
0	1799
0	1800
0	1801
0	1802
0	1803

0	1804
0	1805
0	1806
0	1807
0	1808
0	1809
0	1810
0	1811
0	1812
0	1813
0	1814
0	1815
0	1816
0	1817
0	1818
0	1819
0	1820
0	1821
0	1822
0	1823
0	1824
0	1825
0	1826
0	1827
0	1828
0	1829
0	1830
0	1831
0	1832
0	1833
0	1834
0	1835
0	1836
0	1837
0	1838
0	1839
0	1840
0	1841
0	1842
0	1843
0	1844

0	1845
0	1846
0	1847
0	1848
0	1849
0	1850
0	1851
0	1852
0	1853
0	1854
0	1855
0	1856
0	1857
0	1858
0	1859
0	1860
0	1861
0	1862
0	1863
0	1864
0	1865
0	1866
0	1867
0	1868
0	1869
0	1870
0	1871
0	1872
0	1873
0	1874
0	1875
0	1876
0	1877
0	1878
0	1879
0	1880
0	1881
0	1882
0	1883
0	1884
0	1885

0	1886
0	1887
0	1888
0	1889
0	1890
0	1891
0	1892
0	1893
0	1894
0	1895
0	1896
0	1897
0	1898
0	1899
0	1900
0	1901
0	1902
0	1903
0	1904
0	1905
0	1906
0	1907
0	1908
0	1909
0	1910
0	1911
0	1912
0	1913
0	1914
0	1915
0	1916
0	1917
0	1918
0	1919
0	1920
0	1921
0	1922
0	1923
0	1924
0	1925
0	1926
0	1927

0	1928
0	1929
0	1930
0	1931
0	1932
0	1933
0	1934
0	1935
0	1936
0	1937
0	1938
0	1939
0	1940
0	1941
0	1942
0	1943
0	1944
0	1945
0	1946
0	1947
0	1948
0	1949
0	1950
0	1951
0	1952
0	1953
0	1954
0	1955
0	1956
0	1957
0	1958
0	1959
0	1960
0	1961
0	1962
0	1963
0	1964
0	1965
0	1966
0	1967
0	1968

0	1969
0	1970
0	1971
0	1972
0	1973
0	1974
0	1975
0	1976
0	1977
0	1978
0	1979
0	1980
0	1981
0	1982
0	1983
0	1984
0	1985
0	1986
0	1987
0	1988
0	1989
0	1990
0	1991
0	1992
0	1993
0	1994
0	1995
0	1996
0	1997
0	1998
0	1999
0	2000
0	2001
0	2002
0	2003
0	2004
0	2005
0	2006
0	2007

0	2008
0	2009
0	2010
0	2011
0	2012
0	2013
0	2014
0	2015
0	2016
0	2017
0	2018
0	2019
0	2020
0	2021
0	2022
0	2023
0	2024
0	2025
0	2026
0	2027
0	2028
0	2029
0	2030
0	2031
1	2032
0	2033
0	2034
0	2035
0	2036
0	2037
0	2038
0	2039
0	2040
0	2041
0	2042
0	2043
0	2044
0	2045
0	2046
0	2047
0	2048
0	2049

0	2050
0	2051
0	2052
0	2053
0	2054
0	2055
0	2056
0	2057
0	2058
0	2059
0	2060
0	2061
0	2062
0	2063
1	2064
1	2065
0	2066
0	2067
0	2068
0	2069
0	2070
0	2071
0	2072
0	2073
0	2074
0	2075
0	2076
0	2077
0	2078
0	2079
0	2080
0	2081
0	2082
0	2083
0	2084
0	2085
0	2086
0	2087
0	2088
0	2089
0	2090

0	2091
0	2092
0	2093
0	2094
0	2095
0	2096
0	2097
0	2098
0	2099
0	2100
0	2101
0	2102
0	2103
0	2104
0	2105
0	2106
0	2107
0	2108
0	2109
0	2110
0	2111
0	2112
0	2113
0	2114
0	2115
0	2116
0	2117
0	2118
0	2119
0	2120
0	2121
0	2122
0	2123
0	2124
0	2125
0	2126
0	2127
0	2128
0	2129
0	2130
0	2131
0	2132

0	2133
0	2134
0	2135
1	2136
1	2137
1	2138
1	2139
1	2140
1	2141
1	2142
1	2143
1	2144
1	2145
1	2146
1	2147
1	2148
1	2149
1	2150
1	2151
1	2152
1	2153
1	2154
1	2155
1	2156
1	2157
1	2158
1	2159
1	2160
1	2161
1	2162
1	2163
1	2164
1	2165
1	2166
1	2167
1	2168
1	2169
1	2170
1	2171
1	2172
1	2173
1	2174

1	2175
1	2176
1	2177
1	2178
1	2179
1	2180
1	2181
1	2182
1	2183
1	2184
1	2185
1	2186
0	2187
0	2188
0	2189
0	2190
0	2191
0	2192
0	2193
0	2194
0	2195
0	2196
0	2197
0	2198
0	2199
0	2200
0	2201
0	2202
0	2203
0	2204
0	2205
0	2206
0	2207
0	2208
0	2209
0	2210
0	2211
0	2212
0	2213
0	2214

0	2215
0	2216
0	2217
0	2218
0	2219
0	2220
0	2221
0	2222
0	2223
0	2224
0	2225
0	2226
0	2227
0	2228
0	2229
0	2230
0	2231
0	2232
0	2233
0	2234
0	2235
0	2236
0	2237
0	2238
0	2239
0	2240
0	2241
0	2242
0	2243
0	2244
0	2245
0	2246
0	2247
0	2248
0	2249
0	2250
0	2251
0	2252
0	2253
0	2254
0	2255
0	2256
0	2257

0	2258
0	2259
0	2260
0	2261
0	2262
0	2263
0	2264
0	2265
0	2266
0	2267
0	2268
0	2269
0	2270
0	2271
0	2272
0	2273
0	2274
0	2275
0	2276
0	2277
0	2278
0	2279
0	2280
0	2281
0	2282
0	2283
0	2284
0	2285
0	2286
0	2287
0	2288
0	2289
0	2290
0	2291
0	2292
0	2293
0	2294
0	2295
0	2296
0	2297
0	2298
0	2299
0	2300

0	2301
0	2302
0	2303
0	2304
0	2305
0	2306
0	2307
0	2308
0	2309
0	2310
0	2311
0	2312
0	2313
0	2314
0	2315
0	2316
0	2317
0	2318
0	2319
0	2320
0	2321
0	2322
0	2323
0	2324
0	2325
0	2326
0	2327
0	2328
0	2329
0	2330
0	2331
0	2332
0	2333
0	2334
0	2335
0	2336
0	2337
0	2338
0	2339
0	2340
0	2341
0	2342

0	2343
0	2344
0	2345
0	2346
0	2347
0	2348
0	2349
0	2350
0	2351
0	2352
0	2353
0	2354
0	2355
0	2356
0	2357
0	2358
0	2359
0	2360
0	2361
0	2362
0	2363
0	2364
0	2365
0	2366
0	2367
0	2368
0	2369
0	2370
0	2371
0	2372
0	2373
0	2374
0	2375
0	2376
0	2377
0	2378
0	2379
0	2380
0	2381
0	2382
0	2383
0	2384

0	2385
0	2386
0	2387
0	2388
0	2389
0	2390
0	2391
0	2392
0	2393
0	2394
0	2395
0	2396
0	2397
0	2398
0	2399
0	2400
0	2401
0	2402
0	2403
0	2404
0	2405
0	2406
0	2407
0	2408
0	2409
0	2410
0	2411
0	2412
0	2413
0	2414
0	2415
0	2416
0	2417
0	2418
0	2419
0	2420
0	2421
0	2422
0	2423
0	2424
0	2425
0	2426

0	2427
0	2428
0	2429
0	2430
0	2431
0	2432
0	2433
0	2434
0	2435
0	2436
0	2437
0	2438
0	2439
0	2440
0	2441
0	2442
0	2443
0	2444
0	2445
0	2446
0	2447
0	2448
0	2449
0	2450
0	2451
0	2452
0	2453
0	2454
0	2455
0	2456
0	2457
0	2458
0	2459
0	2460
0	2461
0	2462
0	2463
0	2464
0	2465
0	2466
0	2467
0	2468

0	2469
0	2470
0	2471
0	2472
0	2473
0	2474
0	2475
0	2476
0	2477
0	2478
0	2479
0	2480
0	2481
0	2482
0	2483
0	2484
0	2485
0	2486
0	2487
0	2488
0	2489
0	2490
0	2491
0	2492
0	2493
0	2494
0	2495
0	2496
0	2497
0	2498
0	2499
0	2500
0	2501
0	2502
0	2503
0	2504
0	2505
0	2506
0	2507
0	2508
0	2509
0	2510

0	2511
0	2512
0	2513
0	2514
0	2515
0	2516
0	2517
0	2518
0	2519
0	2520
0	2521
0	2522
0	2523
0	2524
0	2525
0	2526
0	2527
0	2528
0	2529
0	2530
0	2531
0	2532
0	2533
0	2534
0	2535
0	2536
0	2537
0	2538
0	2539
0	2540
0	2541
0	2542
0	2543
0	2544
0	2545
0	2546
0	2547
0	2548
0	2549
0	2550
0	2551

0	2552
0	2553
0	2554
0	2555
0	2556
0	2557
0	2558
0	2559
0	2560
0	2561
0	2562
0	2563
0	2564
0	2565
0	2566
0	2567
0	2568
0	2569
0	2570
0	2571
0	2572
0	2573
0	2574
0	2575
0	2576
0	2577
0	2578
0	2579
0	2580
0	2581
0	2582
0	2583
0	2584
0	2585
0	2586
0	2587
0	2588
0	2589
0	2590
0	2591
0	2592

0	2593
0	2594
0	2595
0	2596
0	2597
0	2598
0	2599
0	2600
0	2601
0	2602
0	2603
0	2604
0	2605
0	2606
0	2607
0	2608
0	2609
0	2610
0	2611
0	2612
0	2613
0	2614
0	2615
0	2616
0	2617
0	2618
0	2619
0	2620
0	2621
0	2622
0	2623
0	2624
0	2625
0	2626
0	2627
0	2628
0	2629
0	2630
0	2631
0	2632
0	2633

0	2634
0	2635
0	2636
0	2637
0	2638
0	2639
0	2640
0	2641
0	2642
0	2643
0	2644
0	2645
0	2646
0	2647
0	2648
0	2649
0	2650
0	2651
0	2652
0	2653
0	2654
0	2655
0	2656
0	2657
0	2658
0	2659
0	2660
0	2661
0	2662
0	2663
0	2664
0	2665
0	2666
0	2667
0	2668
0	2669
0	2670
0	2671
0	2672
0	2673
0	2674

0	2675
0	2676
0	2677
0	2678
0	2679
0	2680
0	2681
0	2682
0	2683
0	2684
0	2685
0	2686
0	2687
0	2688
0	2689
0	2690
0	2691
0	2692
0	2693
0	2694
0	2695
0	2696
0	2697
0	2698
0	2699
0	2700
0	2701
0	2702
0	2703
0	2704
0	2705
0	2706
0	2707
0	2708
0	2709
0	2710
0	2711
0	2712
0	2713
0	2714
0	2715

0	2716
0	2717
0	2718
0	2719
0	2720
0	2721
0	2722
0	2723
0	2724
0	2725
0	2726
0	2727
0	2728
0	2729
0	2730
0	2731
0	2732
0	2733
0	2734
0	2735
0	2736
0	2737
0	2738
0	2739
0	2740
0	2741
0	2742
0	2743
0	2744
0	2745
0	2746
0	2747
0	2748
0	2749
0	2750
0	2751
0	2752
0	2753
0	2754
0	2755
0	2756

0	2757
0	2758
0	2759
0	2760
0	2761
0	2762
0	2763
0	2764
0	2765
0	2766
0	2767
0	2768
0	2769
0	2770
0	2771
0	2772
0	2773
0	2774
0	2775
0	2776
0	2777
0	2778
0	2779
0	2780
0	2781
0	2782
0	2783
0	2784
0	2785
0	2786
0	2787
0	2788
0	2789
0	2790
0	2791
0	2792
0	2793
0	2794
0	2795
0	2796
0	2797

0	2798
0	2799
0	2800
0	2801
0	2802
0	2803
0	2804
0	2805
0	2806
0	2807
0	2808
0	2809
0	2810
0	2811
0	2812
0	2813
0	2814
0	2815
0	2816
0	2817
0	2818
0	2819
0	2820
0	2821
0	2822
0	2823
0	2824
0	2825
0	2826
0	2827
0	2828
0	2829
0	2830
0	2831
0	2832
0	2833
0	2834
0	2835
0	2836
0	2837
0	2838

0	2839
0	2840
0	2841
0	2842
0	2843
0	2844
0	2845
0	2846
0	2847
0	2848
0	2849
0	2850
0	2851
0	2852
0	2853
0	2854
0	2855
0	2856
0	2857
0	2858
0	2859
0	2860
0	2861
0	2862
0	2863
0	2864
0	2865
0	2866
0	2867
0	2868
0	2869
0	2870
0	2871
0	2872
0	2873
0	2874
0	2875
0	2876
0	2877
0	2878
0	2879
0	2880

0	2881
0	2882
0	2883
0	2884
0	2885
0	2886
0	2887
0	2888
0	2889
0	2890
0	2891
0	2892
0	2893
0	2894
0	2895
0	2896
0	2897
0	2898
0	2899
0	2900
0	2901
0	2902
0	2903
0	2904
0	2905
0	2906
0	2907
0	2908
0	2909
0	2910
0	2911
0	2912
0	2913
0	2914
0	2915
0	2916
0	2917
0	2918
0	2919
0	2920
0	2921

0	2922
0	2923
0	2924
0	2925
0	2926
0	2927
0	2928
0	2929
0	2930
0	2931
0	2932
0	2933
0	2934
0	2935
0	2936
0	2937
0	2938
0	2939
0	2940
0	2941
0	2942
0	2943
0	2944
0	2945
0	2946
0	2947
0	2948
0	2949
0	2950
0	2951
0	2952
0	2953
0	2954
0	2955
0	2956
0	2957
0	2958
0	2959
0	2960
0	2961
0	2962

0	2963
0	2964
0	2965
0	2966
0	2967
0	2968
0	2969
0	2970
0	2971
0	2972
0	2973
0	2974
0	2975
0	2976
0	2977
0	2978
0	2979
0	2980
0	2981
0	2982
0	2983
0	2984
0	2985
0	2986
0	2987
0	2988
0	2989
0	2990
0	2991
0	2992
0	2993
0	2994
0	2995
0	2996
0	2997
0	2998
0	2999
0	3000
0	3001
0	3002
0	3003

0	3004
0	3005
0	3006
0	3007
0	3008
0	3009
0	3010
0	3011
0	3012
0	3013
0	3014
0	3015
0	3016
0	3017
0	3018
0	3019
0	3020
0	3021
0	3022
0	3023
0	3024
0	3025
0	3026
0	3027
0	3028
0	3029
0	3030
0	3031
0	3032
0	3033
0	3034
0	3035
0	3036
0	3037
0	3038
0	3039
0	3040
0	3041
0	3042
0	3043
0	3044
0	3045

0	3046
0	3047
0	3048
0	3049
0	3050
0	3051
0	3052
0	3053
0	3054
0	3055
0	3056
0	3057
0	3058
0	3059
0	3060
0	3061
0	3062
0	3063
0	3064
0	3065
0	3066
0	3067
0	3068
0	3069
0	3070
0	3071
0	3072
0	3073
0	3074
0	3075
0	3076
0	3077
0	3078
0	3079
0	3080
0	3081
0	3082
0	3083
0	3084
0	3085
0	3086
0	3087

0	3088
0	3089
0	3090
0	3091
0	3092
0	3093
0	3094
0	3095
0	3096
0	3097
0	3098
0	3099
0	3100
0	3101
0	3102
0	3103
0	3104
0	3105
0	3106
0	3107
0	3108
0	3109
0	3110
0	3111
0	3112
0	3113
0	3114
0	3115
0	3116
0	3117
0	3118
0	3119
0	3120
0	3121
0	3122
0	3123
0	3124
0	3125
0	3126
0	3127
0	3128
0	3129

0	3130
0	3131
0	3132
0	3133
0	3134
0	3135
0	3136
0	3137
0	3138
0	3139
0	3140
0	3141
0	3142
0	3143
0	3144
0	3145
0	3146
0	3147
0	3148
0	3149
0	3150
0	3151
0	3152
0	3153
0	3154
0	3155
0	3156
0	3157
0	3158
0	3159
0	3160
0	3161
0	3162
0	3163
0	3164
0	3165
0	3166
0	3167
0	3168
0	3169
0	3170
0	3171

0	3172
0	3173
0	3174
0	3175
0	3176
0	3177
0	3178
0	3179
0	3180
0	3181
0	3182
0	3183
0	3184
0	3185
0	3186
0	3187
0	3188
0	3189
0	3190
0	3191
0	3192
0	3193
0	3194
0	3195
0	3196
0	3197
0	3198
0	3199
0	3200
0	3201
0	3202
0	3203
0	3204
0	3205
0	3206
0	3207
0	3208
0	3209
0	3210
0	3211
0	3212
0	3213

0	3214
0	3215
0	3216
0	3217
0	3218
0	3219
0	3220
0	3221
0	3222
0	3223
0	3224
0	3225
0	3226
0	3227
0	3228
0	3229
0	3230
0	3231
0	3232
0	3233
0	3234
0	3235
0	3236
0	3237
0	3238
0	3239
0	3240
0	3241
0	3242
0	3243
0	3244
0	3245
0	3246
0	3247
0	3248
0	3249
0	3250
0	3251
0	3252
0	3253
0	3254
0	3255

0	3256
0	3257
0	3258
0	3259
0	3260
0	3261
0	3262
0	3263
0	3264
0	3265
0	3266
0	3267
0	3268
0	3269
0	3270
0	3271
0	3272
0	3273
0	3274
0	3275
0	3276
0	3277
0	3278
0	3279
0	3280
0	3281
0	3282
0	3283
0	3284
0	3285
0	3286
0	3287
0	3288
0	3289
0	3290
0	3291
0	3292
0	3293
0	3294
0	3295
0	3296
0	3297

0	3298
0	3299
0	3300
0	3301
0	3302
0	3303
0	3304
0	3305
0	3306
0	3307
0	3308
0	3309
0	3310
0	3311
0	3312
0	3313
0	3314
0	3315
0	3316
0	3317
0	3318
0	3319
0	3320
0	3321
0	3322
0	3323
0	3324
0	3325
0	3326
0	3327
0	3328
0	3329
0	3330
0	3331
0	3332
0	3333
0	3334
0	3335
0	3336
0	3337
0	3338
0	3339

0	3340
0	3341
0	3342
0	3343
0	3344
0	3345
0	3346
0	3347
0	3348
0	3349
0	3350
0	3351
0	3352
0	3353
0	3354
0	3355
0	3356
0	3357
0	3358
0	3359
0	3360
0	3361
0	3362
0	3363
0	3364
0	3365
0	3366
0	3367
0	3368
0	3369
0	3370
0	3371
0	3372
0	3373
0	3374
0	3375
0	3376
0	3377
0	3378
0	3379
0	3380
0	3381

0	3382
0	3383
0	3384
0	3385
0	3386
0	3387
0	3388
0	3389
0	3390
0	3391
0	3392
0	3393
0	3394
0	3395
0	3396
0	3397
0	3398
0	3399
0	3400
0	3401
0	3402
0	3403
0	3404
0	3405
0	3406
0	3407
0	3408
0	3409
0	3410
0	3411
0	3412
0	3413
0	3414
0	3415
0	3416
0	3417
0	3418
0	3419
0	3420
0	3421
0	3422
0	3423

0	3424
0	3425
0	3426
0	3427
0	3428
0	3429
0	3430
0	3431
0	3432
0	3433
0	3434
0	3435
0	3436
0	3437
0	3438
0	3439
0	3440
0	3441
0	3442
0	3443
0	3444
0	3445
0	3446
0	3447
0	3448
0	3449
0	3450
0	3451
0	3452
0	3453
0	3454
0	3455
0	3456
0	3457
0	3458
0	3459
0	3460
0	3461
0	3462
0	3463
0	3464
0	3465

0	3466
0	3467
0	3468
0	3469
0	3470
0	3471
0	3472
0	3473
0	3474
0	3475
0	3476
0	3477
0	3478
0	3479
0	3480
0	3481
0	3482
0	3483
0	3484
0	3485
0	3486
0	3487
0	3488
0	3489
0	3490
0	3491
0	3492
0	3493
0	3494
0	3495
0	3496
0	3497
0	3498
0	3499
0	3500
0	3501
0	3502
0	3503
0	3504
0	3505
0	3506
0	3507

0	3508
0	3509
0	3510
0	3511
0	3512
0	3513
0	3514
0	3515
0	3516
0	3517
0	3518
0	3519
0	3520
0	3521
0	3522
0	3523
0	3524
0	3525
0	3526
0	3527
0	3528
0	3529
0	3530
0	3531
0	3532
0	3533
0	3534
0	3535
0	3536
0	3537
0	3538
0	3539
0	3540
0	3541
0	3542
0	3543
0	3544
0	3545
0	3546
0	3547
0	3548

0	3549
0	3550
0	3551
0	3552
0	3553
0	3554
0	3555
0	3556
0	3557
0	3558
0	3559
0	3560
0	3561
0	3562
0	3563
0	3564
0	3565
0	3566
0	3567
0	3568
0	3569
0	3570
0	3571
0	3572
0	3573
0	3574
0	3575
0	3576
0	3577
0	3578
0	3579
0	3580
0	3581
0	3582
0	3583
0	3584
0	3585
0	3586
0	3587
0	3588
0	3589
0	3590

0	3591
0	3592
0	3593
0	3594
0	3595
0	3596
0	3597
0	3598
0	3599
0	3600
0	3601
0	3602
0	3603
0	3604
0	3605
0	3606
0	3607
0	3608
0	3609
0	3610
0	3611
0	3612
0	3613
0	3614
0	3615
0	3616
0	3617
0	3618
0	3619
0	3620
0	3621
0	3622
0	3623
0	3624
0	3625
0	3626
0	3627
0	3628
0	3629
0	3630
0	3631
0	3632

0	3633
0	3634
0	3635
0	3636
0	3637
0	3638
0	3639
0	3640
0	3641
0	3642
0	3643
0	3644
0	3645
0	3646
0	3647
0	3648
0	3649
0	3650
0	3651
0	3652
0	3653
0	3654
0	3655
0	3656
0	3657
0	3658
0	3659
0	3660
0	3661
0	3662
0	3663
0	3664
0	3665
0	3666
0	3667
0	3668
0	3669
0	3670
0	3671
0	3672
0	3673
0	3674

0	3675
0	3676
0	3677
0	3678
0	3679
0	3680
0	3681
0	3682
0	3683
0	3684
0	3685
0	3686
0	3687
0	3688
0	3689
0	3690
0	3691
0	3692
0	3693
0	3694
0	3695
0	3696
0	3697
0	3698
0	3699
0	3700
0	3701
0	3702
0	3703
0	3704
0	3705
0	3706
0	3707
0	3708
0	3709
0	3710
0	3711
0	3712
0	3713
0	3714
0	3715
0	3716

0	3717
0	3718
0	3719
0	3720
0	3721
0	3722
0	3723
0	3724
0	3725
0	3726
0	3727
0	3728
0	3729
0	3730
0	3731
0	3732
0	3733
0	3734
0	3735
0	3736
0	3737
0	3738
0	3739
0	3740
0	3741
0	3742
0	3743
0	3744
0	3745
0	3746
0	3747
0	3748
0	3749
0	3750
0	3751
0	3752
0	3753
0	3754
0	3755
0	3756
0	3757
0	3758

0	3759
0	3760
0	3761
0	3762
0	3763
0	3764
0	3765
0	3766
0	3767
0	3768
0	3769
0	3770
0	3771
0	3772
0	3773
0	3774
0	3775
0	3776
0	3777
0	3778
0	3779
0	3780
0	3781
0	3782
0	3783
0	3784
0	3785
0	3786
0	3787
0	3788
0	3789
0	3790
0	3791
0	3792
0	3793
0	3794
0	3795
0	3796
0	3797
0	3798
0	3799
0	3800
0	3801

0	3802
0	3803
0	3804
0	3805
0	3806
0	3807
0	3808
0	3809
0	3810
0	3811
0	3812
0	3813
0	3814
0	3815
0	3816
0	3817
0	3818
0	3819
0	3820
0	3821
0	3822
0	3823
0	3824
0	3825
0	3826
0	3827
0	3828
0	3829
0	3830
0	3831
0	3832
0	3833
0	3834
0	3835
0	3836
0	3837
0	3838
0	3839
0	3840
0	3841
0	3842
0	3843

0	3844
0	3845
0	3846
0	3847
0	3848
0	3849
0	3850
0	3851
0	3852
0	3853
0	3854
0	3855
0	3856
0	3857
0	3858
0	3859
0	3860
0	3861
0	3862
0	3863
0	3864
0	3865
0	3866
0	3867
0	3868
0	3869
0	3870
0	3871
0	3872
0	3873
0	3874
0	3875
0	3876
0	3877
0	3878
0	3879
0	3880
0	3881
0	3882
0	3883

0	3884
0	3885
0	3886
0	3887
0	3888
0	3889
0	3890
0	3891
0	3892
0	3893
0	3894
0	3895
0	3896
0	3897
0	3898
0	3899
0	3900
0	3901
0	3902
0	3903
0	3904
0	3905
0	3906
0	3907
0	3908
0	3909
0	3910
0	3911
0	3912
0	3913
0	3914
0	3915
0	3916
0	3917
0	3918
0	3919
0	3920
0	3921

0	3922
0	3923
0	3924
0	3925
0	3926
0	3927
0	3928
0	3929
0	3930
0	3931
0	3932
0	3933
0	3934
0	3935
0	3936
0	3937
0	3938
0	3939
0	3940
0	3941
0	3942
0	3943
0	3944
0	3945
0	3946
0	3947
0	3948
0	3949
0	3950
0	3951
0	3952
0	3953
0	3954
0	3955
0	3956
0	3957
0	3958
0	3959
0	3960
0	3961
0	3962

0	3963
0	3964
0	3965
0	3966
0	3967
0	3968
0	3969
0	3970
0	3971
0	3972
0	3973
0	3974
0	3975
0	3976
0	3977
0	3978
0	3979
0	3980
0	3981
0	3982
0	3983
0	3984
0	3985
0	3986
0	3987
0	3988
0	3989
0	3990
0	3991
0	3992
0	3993
0	3994
0	3995
0	3996
0	3997
0	3998
0	3999
0	4000
0	4001
0	4002
0	4003
0	4004

0	4005
0	4006
0	4007
0	4008
0	4009
0	4010
0	4011
0	4012
0	4013
0	4014
0	4015
0	4016
0	4017
0	4018
0	4019
0	4020
0	4021
0	4022
0	4023
0	4024
0	4025
0	4026
0	4027
0	4028
0	4029
0	4030
0	4031
0	4032
0	4033
0	4034
0	4035
0	4036
0	4037
0	4038
0	4039
0	4040
0	4041
0	4042
0	4043
0	4044
0	4045

0	4046
0	4047
0	4048
0	4049
0	4050
0	4051
0	4052
0	4053
0	4054
0	4055
0	4056
0	4057
0	4058
0	4059
0	4060
0	4061
0	4062
0	4063
0	4064
0	4065
0	4066
0	4067
0	4068
0	4069
0	4070
0	4071
0	4072
0	4073
0	4074
0	4075
0	4076
0	4077
0	4078
0	4079
0	4080
0	4081
0	4082
0	4083
0	4084
0	4085
0	4086

0	4087
0	4088
0	4089
0	4090
0	4091
0	4092
0	4093
0	4094
0	4095
0	4096
0	4097
0	4098
0	4099
0	4100
1	4101
1	4102
1	4103
0	4104
0	4105
0	4106
0	4107
0	4108
0	4109
0	4110
0	4111
0	4112
0	4113
0	4114
0	4115
0	4116
0	4117
0	4118
0	4119
0	4120
0	4121
0	4122
0	4123
0	4124
0	4125
0	4126
0	4127
0	4128

0	4129
0	4130
0	4131
0	4132
0	4133
0	4134
0	4135
0	4136
0	4137
0	4138
0	4139
0	4140
0	4141
0	4142
0	4143
0	4144
0	4145
0	4146
0	4147
0	4148
0	4149
0	4150
0	4151
0	4152
0	4153
0	4154
0	4155
0	4156
0	4157
1	4158
1	4159
1	4160
1	4161
1	4162
1	4163
1	4164
0	4165
0	4166
0	4167
0	4168
0	4169

0	4170
0	4171
0	4172
0	4173
0	4174
0	4175
0	4176
0	4177
0	4178
0	4179
0	4180
0	4181
0	4182
0	4183
0	4184
0	4185
0	4186
0	4187
0	4188
0	4189
0	4190
0	4191
0	4192
0	4193
0	4194
0	4195
0	4196
0	4197
0	4198
0	4199
0	4200
0	4201
0	4202
0	4203
0	4204
0	4205
0	4206
0	4207
0	4208
0	4209
0	4210
0	4211

0	4212
0	4213
0	4214
0	4215
0	4216
0	4217
0	4218
0	4219
0	4220
0	4221
0	4222
0	4223
0	4224
0	4225
0	4226
0	4227
0	4228
0	4229
0	4230
0	4231
0	4232
0	4233
0	4234
0	4235
0	4236
0	4237
0	4238
0	4239
0	4240
0	4241
0	4242
0	4243
0	4244
0	4245
0	4246
0	4247
0	4248
0	4249
0	4250
0	4251
0	4252

0	4253
0	4254
0	4255
0	4256
0	4257
0	4258
0	4259
0	4260
0	4261
0	4262
0	4263
0	4264
0	4265
0	4266
0	4267
0	4268
0	4269
0	4270
0	4271
0	4272
0	4273
0	4274
0	4275
0	4276
0	4277
0	4278
0	4279
0	4280
0	4281
0	4282
0	4283
0	4284
0	4285
0	4286
0	4287
0	4288
0	4289
0	4290
0	4291
0	4292
0	4293
0	4294

0	4295
0	4296
0	4297
0	4298
0	4299
0	4300
0	4301
0	4302
0	4303
0	4304
0	4305
0	4306
0	4307
0	4308
0	4309
0	4310
0	4311
0	4312
0	4313
0	4314
0	4315
0	4316
0	4317
0	4318
0	4319
0	4320
0	4321
0	4322
0	4323
0	4324
0	4325
0	4326
0	4327
0	4328
0	4329
0	4330
0	4331
0	4332
0	4333
0	4334
0	4335
0	4336

0	4337
0	4338
0	4339
0	4340
0	4341
0	4342
0	4343
0	4344
0	4345
0	4346
0	4347
0	4348
0	4349
0	4350
0	4351
0	4352
0	4353
1	4354
1	4355
1	4356
1	4357
1	4358
0	4359
0	4360
0	4361
0	4362
0	4363
0	4364
0	4365
0	4366
0	4367
0	4368
0	4369
0	4370
0	4371
0	4372
0	4373
0	4374
0	4375
0	4376
0	4377

0	4378
0	4379
0	4380
0	4381
0	4382
0	4383
0	4384
0	4385
0	4386
0	4387
0	4388
0	4389
0	4390
0	4391
0	4392
0	4393
0	4394
0	4395
0	4396
0	4397
0	4398
0	4399
0	4400
0	4401
0	4402
0	4403
0	4404
0	4405
0	4406
0	4407
0	4408
0	4409
0	4410
0	4411
0	4412
0	4413
0	4414
0	4415
0	4416
0	4417
0	4418
0	4419

0	4420
0	4421
0	4422
0	4423
0	4424
0	4425
0	4426
0	4427
0	4428
0	4429
0	4430
0	4431
0	4432
0	4433
0	4434
0	4435
0	4436
0	4437
0	4438
0	4439
0	4440
0	4441
0	4442
0	4443
0	4444
0	4445
0	4446
0	4447
0	4448
0	4449
0	4450
0	4451
0	4452
0	4453
0	4454
0	4455
0	4456
0	4457
0	4458
0	4459
0	4460

0	4461
0	4462
0	4463
0	4464
0	4465
0	4466
0	4467
0	4468
0	4469
0	4470
0	4471
0	4472
0	4473
0	4474
0	4475
0	4476
0	4477
0	4478
0	4479
0	4480
0	4481
0	4482
0	4483
0	4484
0	4485
0	4486
0	4487
0	4488
0	4489
0	4490
0	4491
0	4492
0	4493
0	4494
0	4495
0	4496
0	4497
0	4498
0	4499
0	4500
0	4501

0	4502
0	4503
0	4504
0	4505
0	4506
0	4507
0	4508
0	4509
0	4510
0	4511
0	4512
0	4513
0	4514
0	4515
0	4516
0	4517
0	4518
0	4519
0	4520
0	4521
0	4522
0	4523
0	4524
0	4525
0	4526
0	4527
0	4528
0	4529
0	4530
0	4531
0	4532
0	4533
0	4534
0	4535
0	4536
0	4537
0	4538
0	4539
0	4540
0	4541
0	4542
0	4543

0	4544
0	4545
0	4546
0	4547
0	4548
0	4549
0	4550
0	4551
0	4552
0	4553
0	4554
0	4555
0	4556
0	4557
0	4558
0	4559
0	4560
0	4561
0	4562
0	4563
0	4564
0	4565
0	4566
0	4567
0	4568
0	4569
0	4570
0	4571
0	4572
0	4573
0	4574
0	4575
0	4576
0	4577
0	4578
0	4579
0	4580
0	4581
0	4582
0	4583
0	4584
0	4585

0	4586
0	4587
0	4588
0	4589
0	4590
0	4591
0	4592
0	4593
0	4594
0	4595
0	4596
0	4597
0	4598
0	4599
0	4600
0	4601
0	4602
0	4603
0	4604
0	4605
0	4606
0	4607
0	4608
0	4609
0	4610
0	4611
0	4612
0	4613
0	4614
0	4615
0	4616
0	4617
0	4618
0	4619
0	4620
0	4621
0	4622
0	4623
0	4624
0	4625
0	4626
0	4627

0	4628
0	4629
0	4630
0	4631
0	4632
0	4633
0	4634
0	4635
0	4636
0	4637
0	4638
0	4639
0	4640
0	4641
0	4642
0	4643
0	4644
0	4645
0	4646
0	4647
0	4648
0	4649
0	4650
0	4651
0	4652
0	4653
0	4654
0	4655
0	4656
0	4657
0	4658
0	4659
0	4660
0	4661
0	4662
0	4663
0	4664
0	4665
0	4666
0	4667
0	4668

0	4669
0	4670
0	4671
0	4672
0	4673
0	4674
0	4675
0	4676
0	4677
0	4678
0	4679
0	4680
0	4681
0	4682
0	4683
0	4684
0	4685
0	4686
0	4687
0	4688
0	4689
0	4690
0	4691
0	4692
0	4693
0	4694
0	4695
0	4696
0	4697
0	4698
0	4699
0	4700
0	4701
0	4702
0	4703
0	4704
0	4705
0	4706
0	4707
0	4708
0	4709

0	4710
0	4711
0	4712
1	4713
0	4714
0	4715
0	4716
0	4717
0	4718
0	4719
0	4720
1	4721
0	4722
0	4723
0	4724
0	4725
0	4726
0	4727
0	4728
0	4729
0	4730
0	4731
0	4732
0	4733
0	4734
0	4735
0	4736
0	4737
0	4738
0	4739
0	4740
0	4741
0	4742
0	4743
0	4744
0	4745
0	4746
0	4747
0	4748
0	4749
0	4750

0	4751
0	4752
0	4753
0	4754
0	4755
0	4756
0	4757
0	4758
0	4759
0	4760
0	4761
0	4762
0	4763
0	4764
0	4765
0	4766
0	4767
0	4768
0	4769
0	4770
0	4771
0	4772
0	4773
0	4774
0	4775
0	4776
0	4777
0	4778
0	4779
0	4780
0	4781
0	4782
0	4783
0	4784
0	4785
0	4786
0	4787
0	4788
0	4789
0	4790
0	4791

0	4792
0	4793
0	4794
0	4795
0	4796
0	4797
0	4798
0	4799
0	4800
0	4801
0	4802
0	4803
0	4804
0	4805
0	4806
0	4807
0	4808
0	4809
0	4810
0	4811
0	4812
0	4813
0	4814
0	4815
0	4816
0	4817
0	4818
0	4819
0	4820
0	4821
0	4822
0	4823
0	4824
0	4825
0	4826
0	4827
0	4828
0	4829
0	4830
0	4831
0	4832
0	4833

0	4834	
0	4835	
0	4836	
0	4837	
0	4838	
0	4839	
0	4840	
0	4841	
0	4842	
0	4843	
0	4844	
0	4845	
0	4846	
0	4847	
0	4848	
0	4849	
0	4850	
0	4851	
0	4852	
0	4853	
0	4854	
0	4855	
0	4856	
0	4857	
0	4858	
1	4859	
0	4860	****** ******
0	4861	
0	4862	
0	4863	
0	4864	
0	4865	
0	4866	
0	4867	
0	4868	
0	4869	
0	4870	
0	4871	
0	4872	
0	4873	
0	4874	

0	4875
0	4876
0	4877
0	4878
0	4879
0	4880
0	4881
0	4882
0	4883
0	4884
0	4885
0	4886
1	4887
1	4888
1	4889
0	4890
0	4891
0	4892
0	4893
0	4894
0	4895
0	4896
0	4897
0	4898
0	4899
0	4900
0	4901
0	4902
0	4903
0	4904
0	4905
0	4906
0	4907
0	4908
0	4909
0	4910
0	4911
0	4912
0	4913
0	4914
0	4915

0	4916
0	4917
0	4918
0	4919
0	4920
0	4921
0	4922
0	4923
0	4924
0	4925
0	4926
0	4927
0	4928
0	4929
0	4930
0	4931
0	4932
0	4933
0	4934
0	4935
0	4936
0	4937
0	4938
0	4939
0	4940
0	4941
0	4942
0	4943
0	4944
0	4945
0	4946
0	4947
0	4948
0	4949
0	4950
0	4951
0	4952
0	4953
0	4954
0	4955
0	4956

0	4957
0	4958
0	4959
0	4960
0	4961
0	4962
0	4963
0	4964
0	4965
0	4966
0	4967
0	4968
0	4969
0	4970
0	4971
0	4972
0	4973
0	4974
0	4975
1	4976
0	4977
0	4978
0	4979
0	4980
0	4981
0	4982
0	4983
0	4984
0	4985
0	4986
0	4987
0	4988
0	4989
0	4990
0	4991
0	4992
0	4993
0	4994
0	4995
0	4996
0	4997

0	4998
0	4999
0	5000
0	5001
0	5002
0	5003
0	5004
0	5005
prostatitis	
